# Microvesicles from brain-extract—treated mesenchymal stem cells improve neurological functions in a rat model of ischemic stroke

**DOI:** 10.1038/srep33038

**Published:** 2016-09-09

**Authors:** Ji Yong Lee, Eiru Kim, Seong-Mi Choi, Dong-Wook Kim, Kwang Pyo Kim, Insuk Lee, Han-Soo Kim

**Affiliations:** 1 Institute for BioMedical Convergence, Catholic Kwandong University-International St. Mary’s Hospital, Incheon 22711, Republic of Korea; 2 Department of Biotechnology, College of Life Science and Biotechnology, Yonsei University, Seoul 03722, Republic of Korea; 3 Department of Physiology, Yonsei University College of Medicine, Seoul 03722, Republic of Korea; 4 Department of Applied Chemistry, College of Applied Science, Kyung Hee University, Yongin, 17104, Republic of Korea; 5 Department of Biomedical Sciences, Catholic Kwandong University College of Medicine, Gangneung-si, Gangwon-do 25601, Republic of Korea

## Abstract

Transplantation of mesenchymal stem cells (MSCs) was reported to improve functional outcomes in a rat model of ischemic stroke, and subsequent studies suggest that MSC-derived microvesicles (MVs) can replace the beneficial effects of MSCs. Here, we evaluated three different MSC-derived MVs, including MVs from untreated MSCs (MSC-MVs), MVs from MSCs treated with normal rat brain extract (NBE-MSC-MVs), and MVs from MSCs treated with stroke-injured rat brain extract (SBE-MSC-MVs), and tested their effects on ischemic brain injury induced by permanent middle cerebral artery occlusion (pMCAO) in rats. NBE-MSC-MVs and SBE-MSC-MVs had significantly greater efficacy than MSC-MVs for ameliorating ischemic brain injury with improved functional recovery. We found similar profiles of key signalling proteins in NBE-MSC-MVs and SBE-MSC-MVs, which account for their similar therapeutic efficacies. Immunohistochemical analyses suggest that brain-extract—treated MSC-MVs reduce inflammation, enhance angiogenesis, and increase endogenous neurogenesis in the rat brain. We performed mass spectrometry proteomic analyses and found that the total proteomes of brain-extract—treated MSC-MVs are highly enriched for known vesicular proteins. Notably, MSC-MV proteins upregulated by brain extracts tend to be modular for tissue repair pathways. We suggest that MSC-MV proteins stimulated by the brain microenvironment are paracrine effectors that enhance MSC therapy for stroke injury.

Cell transplantation may become a therapeutic option for patients with central nervous system (CNS) diseases. There is increasing evidence that mesenchymal stem cells (MSCs) ameliorate functional deficits in a variety of CNS disease models, such as cerebral ischemia, spinal cord injury, Parkinson’s disease, and demyelinating diseases^1^,^2^,^3^. We and other groups have shown that in animal models, MSCs can improve functional deficits induced by ischemic stroke^4^,^5^,^6^. Therapeutic benefits are evident within days of cellular injection. By contrast, the rare event of cellular transdifferentiation of MSCs into neural cells, followed by integration into complex neural connections, may take weeks or months^6^,^7^. These results suggest that paracrine factors from MSCs could provide a promising treatment strategy for various CNS diseases.

Recent studies on MSCs have shown that the secretome, exosome, and microvesicle (MV) fractions of conditioned media mimic the beneficial effects of MSCs^8^,^9^. We reported an MV proteome derived from human bone marrow MSCs that may be highly relevant for tissue regeneration^10^. Although the nature of the essential components responsible for the therapeutic effects of MSCs remains to be determined, MVs and exosomes derived from MSCs are potential candidates. In various experimental disease models, such as acute kidney injury^11^, myocardial infarction^12^, liver cirrhosis^13^, endotoxin-induced lung injury^14^, and ischemic stroke^15^, MVs and exosomes have exhibited regenerative capacities comparable to those exerted by MSCs.

MVs are submicron-sized membranous vesicles that are either actively released from cells via secretory compartments or shed from cell surface membranes. MVs are generated by many cell types and serve as vehicles that transfer biological information (e.g., protein, mRNA, and miRNA) to distant cells, thereby affecting their gene expression, proliferation, differentiation, and function^16^. An increase in MSC-derived MVs can be observed in the blood of stroke patients^17^. The transcript and protein profiles of MSCs change in response to the microenvironment^18^. Thus, we hypothesised that culturing MSCs with brain extract *in vitro* would elicit cell responses and changes in the composition of their secretomes, including changes in soluble factors and MVs known to regulate cell proliferation, differentiation, inflammation, tissue regeneration, and angiogenesis.

In this study, we investigated whether MSC-MVs and MVs isolated from MSCs treated with normal (NBE-MSC-MVs) and stroke (SBE-MSC-MVs) rat brain extract would improve functional recovery and tissue regeneration after the administration into a rat model of stroke induced by permanent middle cerebral artery occlusion (pMCAO).

## Results

### MVs from MSCs treated with normal and stroke-injured brain extracts ameliorate functional deficits in rat pMCAO with similar efficacy

**Figure 1:**
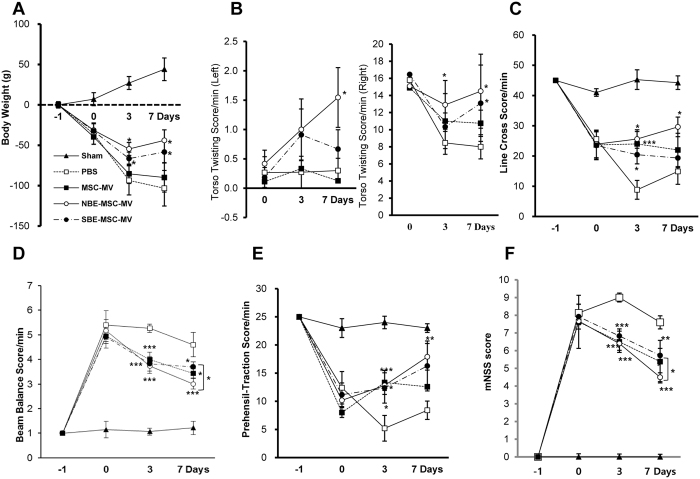
Transplantation of NBE-MSC-MVs and SBE-MSC-MVs improves neurological outcomes. (**A**) Analysis of body weight changes after injection of MSC-MVs, NBE-MSC-MVs, or SBE-MSC-MVs. Loss of body weight was significantly attenuated in rat groups treated with NBE-MSC-MVs (*P* < 0.011, *P* < 0.02) or SBE-MSC-MVs (*P* < 0.025, *P* < 0.05) compared with a group treated with PBS at day 3 and 7, respectively. (**B**) Torso-twisting test (left and right side). Score change from PBS group for right side was significant for NBE-MSC-MVs (*P* < 0.04) at day 3, and for both NBE-MSC-MVs (*P* < 0.025) and SBE-MSC-MVs (*P* < 0.03) at day 7. (**C**) Open field test (line cross score). Score change from PBS group was significant for NBE-MSC-MVs (*P* < 0.025) and SBE-MSC-MVs (*P* < 0.03) at day 3. (**D**) Beam balance test. All injection groups (MSC-MVs, NBE-MSC-MVs, and SBE-MSC-MVs) showed significant improvement compared with the PBS group at day 3 (*P* < 0.01, *P* < 0.001, *P* < 0.001, respectively) and day 7 (*P* < 0.012, *P* < 0.0001, *P* < 0.04, respectively). (**E**) Prehensile traction test. The score was significantly increased in animals treated with MSC-MVs (*P* < 0.0002) and NBE-MSC-MVs (*P* < 0.05) compared with that of the PBS control group at day 3, and in NBE-MSC-MVs (*P* < 0.005) at day 7. (**F**) Modified neurological severity scores (mNSS) indicate that animals treated with NBE-MSC-MVs and SBE-MSC-MVs displayed significant functional enhancement at day 3 (*P* < 0.0001 and *P* < 0.002, respectively) compared with the PBS control group, but animals treated with MSC-MVs did not. At 7 days after injection, animals treated with MSC-MVs, NBE-MSC-MVs, and SBE-MSC-MVs showed significant functional enhancement compared with that of the PBS group (*P* < 0.002, *P* < 0.0001, and *P* < 0.01, respectively). *P < 0.05; **P < 0.01, ***P < 0.001; *n* = 10 per group; Day −1, time of pMCAO surgery; Day 0, time of MV injection; Day 3 and 7, 3 and 7 days after MV injection, respectively. Sample sizes are *n* = 5 for sham-operated, *n* = 10 for NBE-MSC-MV and SBE-MSC-MV treatment groups, and *n* = 7 for MSC-MV treatment group.

In response to tissue injury, tissue repair and regeneration signals may be delivered to and sensed by resident stem cells. These stem cells then generate MVs, which are paracrine mediators that can reprogram the fate of other local stem cells^19^. Systemic or local administration of MSCs provides functional benefits in a number of animal stroke models. We hypothesised that MVs from MSCs significantly contribute to the observed benefits of MSC administration, and signalling molecules secreted from a damaged microenvironment could enhance the therapeutic effect of MSC-MVs. To test these hypotheses, we analysed the therapeutic effect of NBE-MSC-MVs, SBE-MSC-MVs, and untreated MSC-MVs. To examine the therapeutic effects of these three MV preparations, we induced a pMCAO model of the ischemic brain in rats. A single injection of MVs (0.2 mg/kg rat body weight) of NBE-MSC-MVs, SBE-MSC-MVs, and MSC-MVs was administered 48 h after the induction of pMCAO via the right common carotid artery. After induction of ischemic stroke, body weight change was observed in all the rats. Loss of body weight was significantly attenuated in groups treated with NBE-MSC-MVs or SBE-MSC-MVs compared with that of a control group treated with phosphate buffered saline (PBS) on days 3 and 7, but was not significantly different than that of rats treated with MSC-MVs (Fig. 1A).

Behavioural performance after ischemic stroke was assessed using the following tests: open field, foot fault, beam balance, prehensile traction, and torso-twisting. All rats exhibited severe functional deficits at postoperative day 1, whereas sham controls showed no evidence of functional deficits. However, starting at 3 days after treatment, rat groups treated with NBE-MSC-MVs or SBE-MSC-MVs exhibited significant functional enhancement for all parameters (Fig. 1B–F) compared with that of the PBS-treated control group. We observed that untreated MSC-MVs provided no significant improvement in the torso-twisting and line cross tests (Fig. 1B,C), and less significant improvement than that of NBE-MSC-MVs in the beam balance test and the modified neurological severity score (mNSS) (Fig. 1D,F). Notably, the administration of SBE-MSC-MVs did not show better behavioural outcome compared with that of NBE-MSC-MVs in all parameters. Conversely, the analyses revealed that rats treated with NBE-MSC-MVs tended to achieve slightly better scores in the beam balance test and mNSS. These combined results suggest that the brain extract microenvironment facilitates the therapeutic activity of MSC-MVs, and brain extract derived from a pathological (stroke) brain does not provide additional benefits in promoting the therapeutic effect of MSC-MVs and improving neurological outcome.

### MVs from MSCs treated with normal and stroke-injured brain extracts reduce brain infarction area with similar efficacy

**Figure 2:**
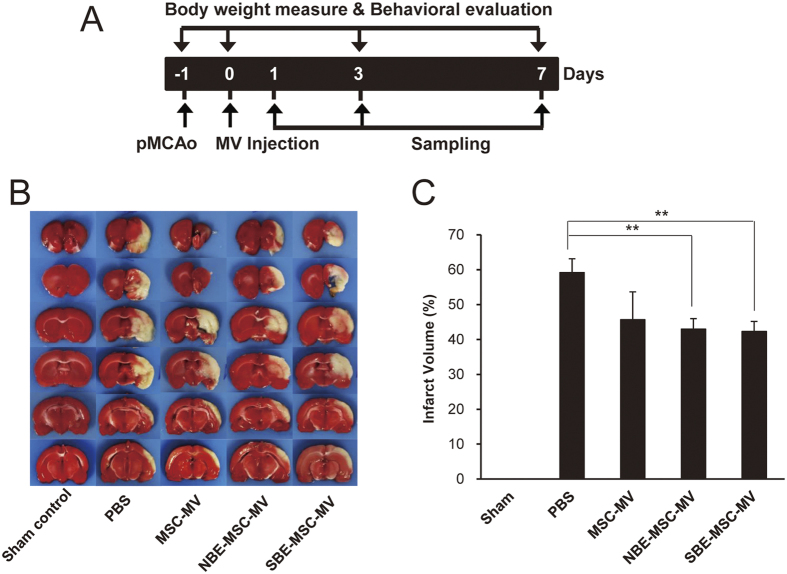
NBE-MSC-MV and SBE-MSC-MV injection reduces infarct size in a rat stroke model. (**A**) Schematic diagram depicting the experimental design of this study. Numerals in the black bar represent the days of experiment. Day −1, time of pMCAO surgery; Day 0, time of MV injection; Day 3 and 7, 3 and 7 days after MV injection. Downward arrows indicate the days for behavioural tests and weight measurement. Upward arrows indicate the days for treatments and brain tissue sampling. (**B**) Representative TTC-stained brain sections from rats treated with PBS (control), MSC-MVs, NBE-MSC-MVs, and SBE-MSC-MVs show ischemic lesions on day 7 after MV treatment. (**C**) Bar graph depicting infarct size in all animals. The infarct area in the ipsilateral hemisphere is expressed as a percentage of the contralateral hemisphere area. Values are indicated as means ± SEM. **P < 0.01 compared with controls. Sample size *n* = 5 for sham control, *n* = 10 for NBE-MSC-MV and SBE-MSC-MV treatment groups, and *n* = 7 for MSC-MV treatment group.

Brain infarction area (the ratio of the damaged ipsilateral area to the contralateral hemisphere area) was measured via 2,3,5,-Triphenyl tetrazolium chloride (TTC) staining of the brain on post-treatment day 7. Infarct lesions primarily appeared in the cortex and striatum. The infarction area was demonstrably larger in ischemic brains treated with PBS than in those treated with NBE-MSC-MVs and SBE-MSC-MVs (Fig. 2). The infarction area of PBS-treated (control) rats was 59.19 ± 3.95%. By contrast, the infarction area was significantly reduced in rats treated with NBE-MSC-MVs (42.31 ± 2.80%) or SBE-MSC-MVs (43.00 ± 2.98%) (*P* < 0.01). A rat group treated with MSC-MVs showed a tendency of reduction in infarction area (45.70 ± 7.95%), but this reduction was not statistically significant for the given sample size. There was no significant difference in lesion volume between groups treated with NBE-MSC-MVs or SBE-MSC-MVs, indicating that there is no significant difference between normal and stroke-injured brain extracts for MV stimulation. These combined results suggest that both normal and injured brain extracts can enhance the therapeutic effects of MSC-MVs by reducing infarction area, and extract from injured brains does not confer greater efficacy than extract from normal brains in promoting the therapeutic effect of MSC-MVs.

### Extracts of normal and stroke-injured brains have similar profiles of key signalling molecules

MSCs are known to respond to microenvironmental changes and, conversely, function to maintain microenvironmental homeostasis. Therefore, it is reasonable to hypothesise that signalling molecules secreted in response to local tissue damage can further promote the therapeutic effect of MSCs. However, we observed no significant enhancement of therapeutic effect by MSCs preconditioned with stroke-injured brain extract compared with that of MSCs preconditioned with normal brain extract. We investigated whether the functional effects induced by treatment with brain extracts were due to molecules in the extracts that facilitated the secretion and/or enhanced the therapeutic effects of MSC-MVs. This experiment was performed by analysing the expression levels of 90 key signalling proteins (such as cytokines) in each brain extract using a rat cytokine array (see Materials and Methods). Consistent with the observed similarity in the therapeutic efficacy conferred by normal and stroke-injured brain extracts, the measured quantities of 90 key signalling proteins showed high positive linear correlation (*r*^2^ = 0.88) in the two extracts, which suggested that they contained similar signalling-protein profiles. This can explain why extract of the stroke-injured brain does not confer additional enhancement of the therapeutic benefit conferred by extract of the normal brain.

### MVs from MSCs treated with brain extract induce neurogenesis and modulate inflammation in ischemic tissue

**Figure 3:**
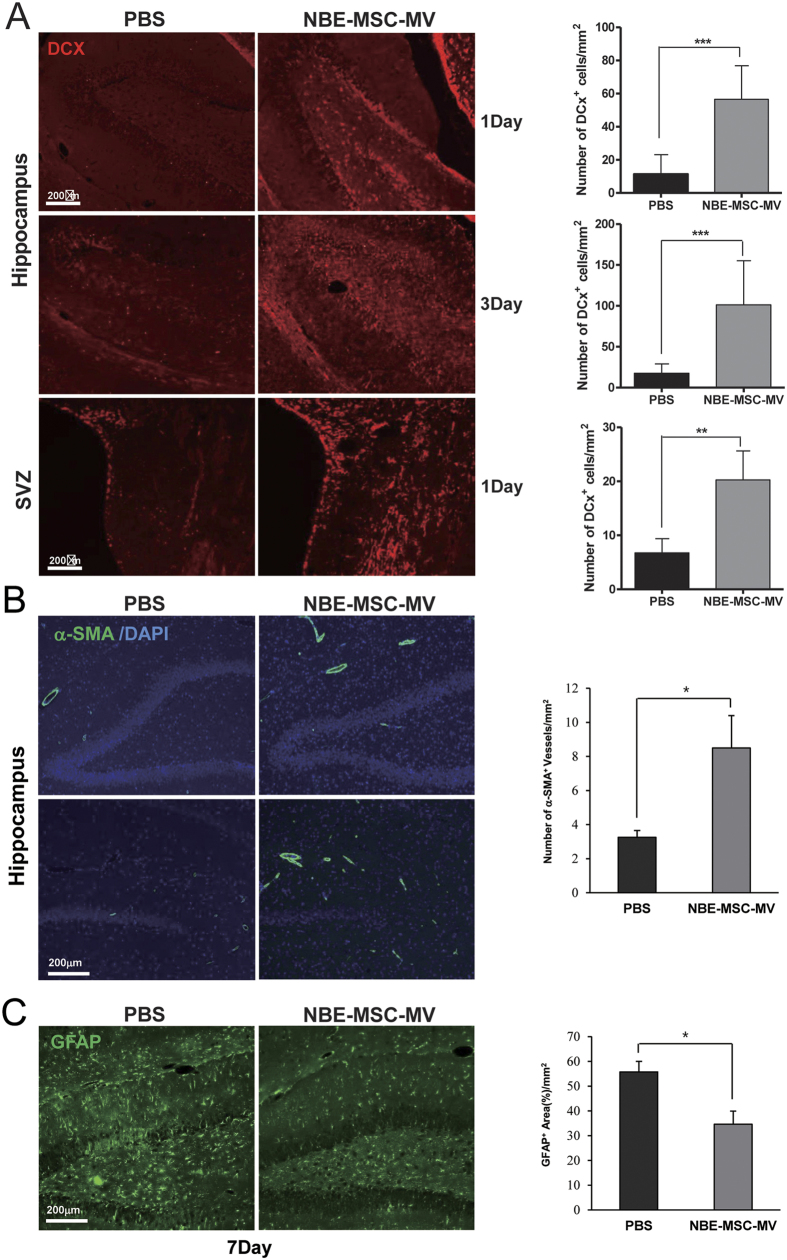
NBE-MSC-MV administration increases neurogenesis and reduces reactive astrogliosis at the ischemic boundary in the rat brain. (**A**) Compared with PBS control treatment, the number of DCX-positive cells at the ischemic boundary significantly increased immediately after NBE-MSC-MV treatment. *P < 0.05, **P < 0.01, ***P < 0.001, *n* = 5 per group. (**B**) Representative micrographs of α-SMA-positive vessels in ipsilateral hippocampal vessels at day 7. Compared with PBS control treatment, the number of α-SMA-positive vessels increased in the ipsilateral hippocampal area after NBE-MSC-MV treatment. Data are presented as mean numbers of α-SMA-positive vessels per group. *P < 0.05, *n* = 5 per group. (**C**) The number of GFAP-positive cells significantly decreased in response to NBE-MSC-MV transplantation compared with that of PBS control injection at day 7. Data are presented as percent of GFAP-positive area/field. *P < 0.05, *n* = 5 per group. Scale bar = 200 μm. Values are indicated as means ± SD.

Previous studies show that ischemic stroke promotes spontaneous neurogenesis in the subventricular zone (SVZ) and subgranular zone of the hippocampus and induces migration of neuroblasts to the ischemic boundary^20^,^21^. To examine the effect of MSC-MVs treated with brain extract on the proliferation of neural progenitor cells and immature neurons in the ischemic region, sagittal sections of brain tissue were stained for doublecortin (DCX), a marker of migrating neuroblast progenitor cells, 7 days after MV injection. The therapeutic efficacy and key signalling-protein profiles did not significantly differ between NBE-MSC-MVs and SBE-MSC-MVs (see Fig. 1). Therefore, we used NBE-MSC-MVs to perform immunohistochemical analyses and investigate the underlying mechanisms of brain tissue regeneration. The number of DCX-positive migrating neuroblasts in the lateral ventricle area and hippocampal region of stroke-affected hemispheres significantly increased after treatment with NBE-MSC-MVs compared to that of PBS (Fig. 3A), suggesting that NBE-MSC-MVs potentially promote neurogenesis by neural stem cells in the hippocampus and SVZ, and these cells migrate toward the ischemic boundary after stroke. By contrast, only a few DCX-positive cells confined to the SVZ were detected contralateral to the ischemic lesion in untreated rats (data not shown).

We also investigated the effect of NBE-MSC-MVs on angiogenesis in the ischemic region 7 days after MV injection using α-smooth muscle actin (α-SMA) as a prominent marker of vascular smooth muscle cells (Fig. 3B). The α-SMA-positive cells were identified in the ipsilateral hippocampal area. The number of α-SMA-positive cells significantly increased in the NBE-MSC-MV-treated group (8.5 ± 1.94) compared with the PBS-treated control group (3.25 ± 0.49). These results suggest that administration of NBE-MSC-MV promotes neovascularisation in the ischemic area after a stroke. We demonstrated previously that pMCAO in the rat and subsequent MSC transplantation reduced glial fibrillary acidic protein (GFAP)^6^, which is a marker of astrogliosis. A similar immunosuppressive effect was observed in the pMCAO rats treated with NBE-MSC-MVs (Fig. 3C). Immunostaining revealed that the number of GFAP-positive astrocytes was significantly reduced in the ischemic striatal region of NBE-MSC-MV-treated rats compared with that of the control group. These results indicate that NBE-MSC-MV treatment can reduce astrocyte activation, suggesting anti-inflammatory and anti-astrogliosis effects during ischemic stroke.

**Figure 4:**
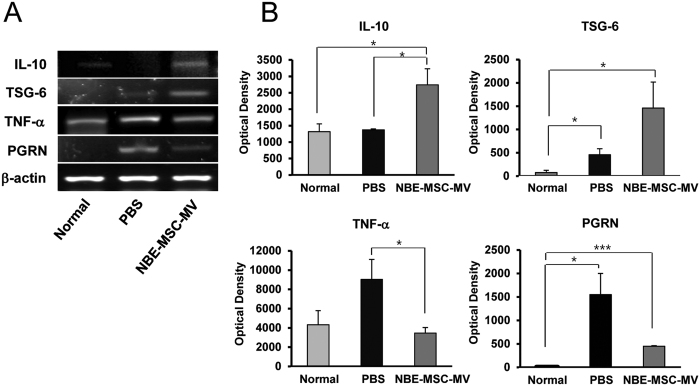
Anti-inflammatory effects of NBE-MSC-MVs in ischemic rat brain. (**A**) Representative RT-PCR results. (**B**) Quantification of inflammatory and anti-inflammatory gene expression. NBE-MSC-MVs reduced inflammatory factors and enhanced anti-inflammatory factors in ischemic rat brain tissue. Values are indicated as means ± SEM. *P < 0.05, **P < 0.01, *n* = 7 per group.

Increased expression of pro-inflammatory cytokines is considered as a hallmark of inflammation after stroke in the ischemic hemisphere^22^. We found that TNF-α and progranulin (PGRN) levels increased in the lesions of PBS-treated control rats (Fig. 4A,B). Tumour necrosis factor-stimulated gene (*TSG*)-6, which is induced by inflammatory cytokines such as TNF-α and IL-1 as a part of the compensatory mechanism of tissue repair^23^, is slightly upregulated in the lesions of PBS-treated groups. Treatment with NBE-MSC-MVs greatly upregulated expression of the anti-inflammatory cytokines IL-10 (1.9-fold increase) and TSG-6 (3.2-fold increase) in the rat stroke model. Conversely, expression of the pro-inflammatory factors tumour necrosis factor (*TNF*) and progranulin (*PGRN*) was significantly attenuated in brain tissue treated with NBE-MSC-MV compared with that of PBS-treated brain tissue.

### The proteomes of MSC-MVs treated with brain extract are highly enriched for known vesicular proteins

To identify the therapeutic factors associated with the observed behavioural recovery, neurogenesis, and immunosuppression conferred by MSC-MVs treated with brain extract, we analysed the NBE-MSC-MV and SBE-MSC-MV proteomes using mass spectrometry. Peptide spectra derived from mass spectrometry-based proteomic analysis were searched against the Human Ensembl GRCh37.75^24^ protein database. Several database search algorithms have complementary sensitivity and specificity. MSblender^25^ software increases the number of peptides identified by statistically combining search scores from multiple algorithms. We integrated the database search scores from three algorithms (Comet-2014.01_rev.0^26^, MSGFDB-v7780^27^, and Myrimatch-2.1.138^28^) in our proteome pipeline. An identical analysis was performed for spectral data from three batches of MSC-MV samples treated with brain extract and for previously published spectral data of control untreated MSC-MVs^29^.

**Figure 5:**
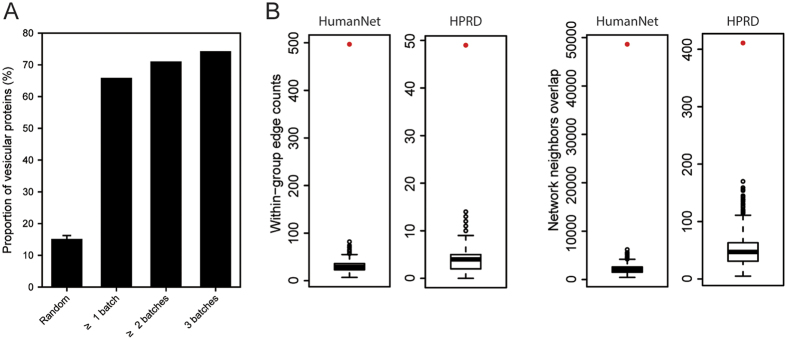
Functional analysis of the NBE-MSC-MV proteome. (**A**) The proportions of vesicular proteins in the NBE-MSC-MV proteome supported by more than 1, 2, or 3 experimental batches and random proteomes (e.g., 10 random samplings of 1,000 proteins), indicating that the NBE-MSC-MV proteome is enriched for vesicular proteins annotated in the Vesiclepedia database. The degree of enrichment for vesicular proteins was higher for proteins identified in multiple NBE-MSC-MV batches. (**B**) Plots from ‘within-group connectivity’ and ‘sharing network neighbour’ analysis of the NBE-MSC-MV proteome for a functional network (HumanNet) and a protein-protein interaction network (HPRD). Box-and-whisker plots represent ‘within-group edge count’ and ‘network neighbours overlap’ scores for 1,000 random gene sets of equal size, and red dots represent scores for the NBE-MSC-MV proteome.

Statistical integration of the search results enabled the identification of 1,523 human proteins from 775 genes that appeared in at least one batch, 921 proteins from 474 genes that appeared in at least two batches, and 591 proteins from 320 genes that appeared in all three batches of NBE-MSC-MVs. Similar analysis identified 1,349 human proteins from 669 genes that appeared in at least one batch, 807 proteins from 465 genes that appeared in at least two batches, and 521 proteins from 317 genes that appeared in all three batches of SBE-MSC-MVs (Supplementary Table S1). We found 1,183 proteins (70% of the union proteome set) that were present in both proteomes. Consistent with our previous report, we detected signatures of the endosome and a typical set of proteins associated with the exosome/MV, such as glyceraldehyde 3-phosphate dehydrogenase (GAPDH), 14-3-3 proteins, Gα proteins, clathrin, annexins (ANXs), Rab proteins, actin, tubulin, HSP70, and HSP90. The proteome also included proteins associated with MV/exosome biogenesis, vesicle trafficking, Rab-related GTPases, anti-apoptosis/programmed cell death, and neuron differentiation/neurogenesis. We searched the NBE-MSC-MV and SBE-MSC-MV proteomes using Vesiclepedia^30^, a database of vesicular proteins created from curation of the literature (Fig. 5A and Supplementary Fig. S1A). Both NBE-MSC-MV and SBE-MSC-MV proteomes contained proteins that were identified in at least one batch and were highly enriched for MV, exosome, and microparticle proteins annotated in the Vesiclepedia database, suggesting that our proteomic study reliably identified the MV proteome. The same proteomes identified in at least two batches showed even higher enrichment for vesicular proteins in the Vesiclepedia database, indicating that a protein assigned to the proteome of brain-extract—treated MSC-MVs has a relatively strong probability of being a vesicular protein.

### Upregulated proteins in NBE-MSC-MVs are functionally coherent

MSC-MVs treated with brain extract have enhanced therapeutic effects in a rat stroke model compared with that of untreated MSC-MVs. We hypothesised that upregulated proteins in these MVs had enhanced therapeutic effects. To test this hypothesis, we identified MV proteins that were upregulated in response to brain extract. A total of 286 proteins from 160 genes were upregulated in NBE-MSC-MVs compared with untreated MSC-MVs (Supplementary Table S2). We used the functional gene network HumanNet^31^, and a protein-protein interaction network derived from Human Protein Reference Database (HPRD)^32^, and found that the 160 upregulated genes were significantly more connected to one another than would be expected by random chance (Fig. 5B). The high modularity of the upregulated NBE-MSC-MV proteins in co-functional or protein-protein interaction networks supports the idea that brain extract contains factors that activate MSC-MV proteins, which collaboratively participate in the process of tissue regeneration. We found similarly high network modularity of the upregulated SBE-MSC-MV proteins. A total of 286 proteins from 156 genes were upregulated in SBE-MSC-MVs compared with untreated MSC-MVs (Supplementary Table S3), and the unregulated genes identified in SBE-MSC-MV also were significantly more connected to one another than would be observed through random chance (Supplementary Fig. S1B).

### Construction of a network of NBE-MSC-MV proteins involved in tissue repair

**Figure 6:**
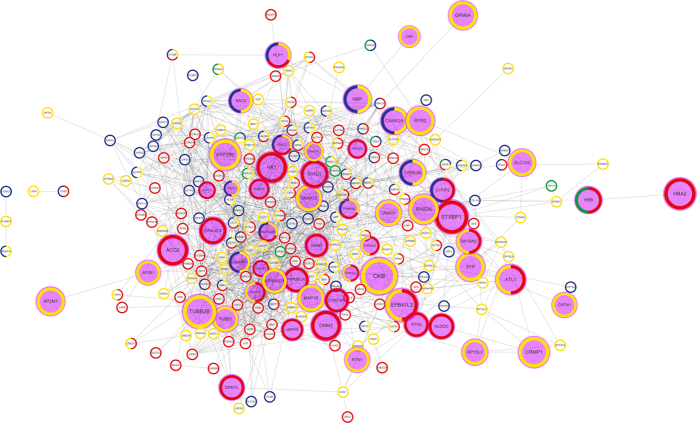
Proposed network of 236 NBE-MSC-MV proteins belonging to four functional categories involved in tissue repair. Each node and edge of the network represents a human gene and a functional link, respectively. Functional links were derived from a co-functional network of human genes (HumanNet). We found that 236 of 272 genes associated with GO terms for angiogenesis, neurogenesis, anti-inflammation, and apoptosis are interconnected by functional links in the largest component of the network. The circumference of each node represents the proportion of proteins from each of the four functional categories: green for angiogenesis, blue for anti-inflammation, yellow for neurogenesis, and red for apoptosis. Upregulated NBE-MSC-MV proteins are shown as purple nodes. The size of the purple node represents the fold change.

To gain a holistic view of therapeutic modules in NBE-MSC-MVs, we employed a systems biology approach. The 1,523 identified proteins (from 775 genes) in the human NBE-MSC-MV proteome represent a wide variety of functional categories. To reduce the complexity of functional analysis, we focused on four well-characterised functional categories involved in tissue repair: angiogenesis, neurogenesis, anti-inflammation, and apoptosis. We identified 272 genes associated with GO terms related to these four functional categories, with significant enrichment for each category: 11 of 89 angiogenesis-related genes, 76 of 1,475 anti-inflammatory genes, 156 of 1,620 neurogenesis-related genes, and 122 of 1,697 apoptosis-related genes (*P* = 3.24 × 10^−4^, 4.46 × 10^−4^, 9.87  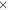 10^−30^, and 2.70  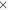 10^−13^, respectively, by Fisher’s exact test) were represented (Supplementary Table S4). We then constructed a network of the 272 therapeutic genes by projecting them on the HumanNet functional network (Fig. 6). HumanNet connected 247 of the 272 genes with 1,439 links. This densely wired network suggests a high degree of functional association among NBE-MSC-MV proteins belonging to these four functional categories. Notably, the network was highly enriched for upregulated NBE-MSC-MV proteins (*P* = 1.91 × 10^−80^, Fisher’s exact test), suggesting that stimulating factors from brain extract may target these MSC-MV proteins. We were able to construct a similar protein network for SBE-MSC-MV (Supplementary Fig. S2), which has 1,294 links among 222 genes belonging to the four functional categories involved in tissue repair (Supplementary Table S5). These results also demonstrated the importance of proteomic analysis for investigating the effects of brain extract on the MSC-MV proteome in regulating tissue repair pathways.

## Discussion

A number of clinical trials utilising MSCs from bone marrow, adipose tissue, and umbilical cord are underway, and evidence of the beneficial effects of cellular therapeutics for stroke and other neurodegenerative disorders is accumulating^33^,^34^,^35^. Previous studies reported that at least some of the therapeutic effects of transplanted cells were due to paracrine factors derived from the grafted cells; stem cells release membranous vesicles that can transmit information between cells, thereby reprogramming target cells^36^,^37^. These data prompted us to examine membranous vesicles of the MSCs. In this study, we investigated the proteome and therapeutic effects of MVs derived from MSCs treated with brain extract in a rat model of ischemic stroke.

Our *in vivo* data demonstrated that both NBE-MSC-MVs and SBE-MSC-MVs improved neurological outcomes and markedly reduced ischemic lesions. In agreement with previous findings^38^,^39^, the results of this study clearly support the hypothesis that MSC transplantation or injection promotes endogenous neurogenesis and improves behavioural deficits after ischemic stroke. The NBE-MSC-MVs significantly increased the level of DCX, a reliable marker of neural progenitor cells/neuroblasts, in the lateral ventricles and hippocampal regions of stroke-damaged hemispheres, and there was a comparable increase in DCX-positive cells in the hippocampal region of the ischemic hemisphere in SBE-MSC-MV-treated rats (Supplementary Fig. S3). Although recent studies suggest that non-neurogenic cells with weak DCX-positivity are detectable in various brain regions^40^,^41^, we found that the number of strong DCX-positive cells was significantly elevated compared with that of PBS-treated controls. Additionally, these DCX-positive cells were only detectable in the lateral ventricles and hippocampal regions of stroke-damaged hemispheres at day 7, but were not detected in the cerebral cortex or other brain regions.

We found that NBE-MSC-MVs significantly increased the number of α-SMA-positive cells in the ipsilateral hippocampus area after stroke. α-SMA is the actin isoform that predominates within vascular smooth muscle cells and has an important role in fibrogenesis^42^. Recent studies indicate that neovascularisation (angiogenesis and arteriogenesis) has considerable value for restorative stroke treatment. Previous studies have shown that stem cell transplantation promotes angiogenesis and arteriogenesis in the rat and mouse stroke models^43^,^44^. Sharma *et al*.^45^ reported that the number of α-SMA-positive cells increased in the infarct region up to day 7, and thereafter incorporated into small vessels in a rat pMCAO-induced stroke model. In agreement with recent studies, our results suggest that MSC-MVs treated with brain extract have therapeutic effects on vascular remodelling (angiogenesis and arteriogenesis) after ischemic stroke.

NBE-MSC-MVs greatly attenuated astrocyte activation in the ischemic region. Astrocytes are known to have an important role in maintaining brain homeostasis; these cells become reactive in response to injury, stimulate cell proliferation, and release bioactive substances^46^, which are essential for controlling injury-mediated tissue damage and tissue remodelling. However, astrocyte dysfunction may contribute to disease mechanisms, resulting in global brain damage, such as ischemic stroke^47^. Over activation of reactive astrocytes, which involves inflammation, leads to the formation of glial scars on regenerated brain tissue; this can be a significant barrier to tissue repair and regeneration. The reactive astrocytes around lesions in PBS-treated rats reflect a spontaneous repair mechanism that limits the spread of injury by forming glial scars^48^, and MSC or NBE-MSC-MV treatment can be beneficial by reducing the number of reactive astrocytes and thereby creating a microenvironment that is permissive for axonal sprouting and neurogenesis. Astrocytes are the most abundant cell type in the brain, and recent efforts have focused on the manipulation of astrocyte function in ischemic stroke^49^,^50^,^51^. The results of our study suggest that the mechanism underlying the therapeutic effects of NBE-MSC-MVs on ischemic stroke can encompass multiple actions that involve enhanced neurogenesis, cerebral blood supply, and anti-inflammation therapy in pMCAO rats.

Although the data show positive effects of MSC-MVs on the ischemic rat, no definitive conclusion can be reached due to the insufficient sample size for the significance test. MVs prepared from untreated MSCs have low protein yields (<0.3 mg/3 × 10^8^ cells/48 h; data not shown), which could be a significant drawback for future clinical applications. We hypothesised that stimuli from the ischemic brain extract may increase the amount of MVs while preserving or enhancing the neuroprotective effects of MSCs. Indeed, the amount of MVs from MSC-conditioned medium was greatly increased by treatment with normal or ischemic brain extracts (2.2 mg /3 × 10^8^ cells/48 h for NBE-MSC-MVs and 2.5 mg/3 × 10^8^ cells/48 h for SBE-MSC-MVs; data not shown).

Whereas our study analysed the proteomic profiles of MVs derived from MSCs treated with brain extract and the therapeutic value of MV proteins in a rat pMCAO model, we cannot exclude the possibility that mRNAs and miRNAs in the MV preparation played key roles in MSC-mediated neuroprotection. Recently, Xin *et al*. reported that exposure of MSCs to ischemic brain extract increased exosome levels of miRNAs associated with neurite outgrowth, suggesting that MSCs can produce high levels of therapeutic exosomes in response to injury^37^. In addition, they demonstrated that systemic administration of MSC-derived exosomes to rats with ischemic stroke improved functional outcomes, with therapeutic benefits reflecting those observed after systemically administering MSCs^15^.

We analysed the proteomic profiles of MVs from MSCs treated with normal and stroke-injured brain extract to identify the factors that contribute to improving neurological function after an ischemic stroke. We believe that the therapeutic effects of MVs on complex injuries such as ischemic stroke are likely to result from multiple factors rather than a single factor. Although these factors may include proteins, mRNA, miRNA, lipids, and other molecules, our primary focus was on identifying candidate MV proteins that participate in brain tissue regeneration. The extract-treated MSC-MV proteomes were characterised using MSblender^52^, an algorithm that integrates results from multiple peptide search algorithms via multivariate modelling and can identify substantially more peptides and proteins than a single search algorithm, for a given confidence level. By comparing our results with a literature-based database, we determined that the majority of identified MV proteins were known vesicular proteins. These results indicate the reliability of our MSC-MV proteome sample preparation and mass spectrometry-based proteomic analysis. We also identified MSC-MV proteins upregulated by brain-extract treatment. Interestingly, enolase 2 (*ENO2*), which encodes a neuron-specific enolase (NSE) and was previously reported to be a marker of acute ischemic stroke^53^, was upregulated in extract-treated MSC-MVs.

We measured the functional coherence among the upregulated genes using their association in co-functional networks, and the resultant measures indicated significantly higher functional coherence than would be expected by random chance. This functional modularity suggests that signals from brain extract stimulate a group of functionally coherent MV proteins, thereby activating therapeutic protein modules. The functional modularity also enabled reconstruction of the network of therapeutic effectors in MSC-MVs. We reconstructed a network of brain-extract—treated MSC-MV proteins involved in four well-characterised biological processes related to tissue regeneration, anti-inflammation, angiogenesis, neurogenesis, and apoptosis, using the functional gene network HumanNet. Notably, the network was highly enriched for MSC-MV proteins that were upregulated by brain-extract treatment. This is a strong indication that this network reflects brain extract-induced changes in the regulation of functional modules linked to MSC-MV therapeutic effects. Therefore, this network may contain key biopharmaceutical factors related to MSC-based cell therapy.

Previous studies proposed the beneficial effects of ischemic brain extracts over normal brain extracts^18^,^54^,^55^. Although we anticipated differences in the therapeutic efficacy of these two preparations under the assumption that stroke-injured brain extract contains stronger signals for tissue regeneration, our result showed similar efficacy of MVs from MSCs treated with ischemic brain extract and normal brain extract in behavioural recovery and infarct size reduction. This could be due to the presence of cytotoxic or inhibitory factors in the ischemic brain extracts used for MSC preconditioning. Brain tissue affected by ischemic necrosis undergoes tissue destruction with massive inflammatory responses. Although progressive ischemic damage with marked increases in activated microglial cells and astrocytes in the infarct lesion was documented at 48 h after pMCAO in SD rats^56^, our cytokine array data revealed no statistically significant differences between normal brain extract and stroke-injured brain extract. This implies that stimulation of MSCs with normal brain extract and stroke-injured brain extract may generate MVs with comparable function and similar molecular contents. Indeed, we found that 70% of identified proteins were shared between NBE-MSC-MV and SBE-MSC-MV proteomes.

## Conclusions

Although a number of clinical trials of stem cell transplantation for neurodegenerative diseases such as stroke are underway, and an impressive body of evidence documenting the clinical benefits of such approaches is accumulating, newly introduced approaches utilising MVs/exosomes derived from MSCs represent a novel and safe alternative. In this study, we demonstrated that MVs from brain-extract—treated MSCs exert therapeutic effects in a rat model of ischemia, promoting angiogenesis, neurogenesis, and anti-inflammation to enhance behavioural recovery and reduce infarct size. Therefore, our study supports the hypothesis that the therapeutic efficacy of MSCs is largely mediated by paracrine action of MVs. The use of MVs derived from preconditioned mesenchymal stem cells offers a novel, safer alternative to cellular therapeutics for tissue regeneration.

## Materials and Methods

### Rat model of permanent middle cerebral artery occlusion and preparation of brain extract

All experimental procedures were performed in accordance with protocols approved by the Institutional Animal Care and Use Committee and the Institutional Review Board of Catholic Kwandong University-International St. Mary’s Hospital (Incheon, Korea). The investigators responsible for functional evaluation and molecular and histological studies were blinded to the treatment groups. All animals were housed in a temperature-controlled animal facility with a 12-h light/dark cycle. Male Sprague-Dawley rats weighing 200–250 g were anaesthetised with 3% isoflurane (Hana Pharm, Seoul, Korea) in a mixture of 80% N_2_O and 20% O_2_. Rectal temperature was maintained at 37 °C during the surgical procedure using heating pads. Permanent middle cerebral artery occlusion (pMCAO) was induced as described previously^6^. Briefly, the left common carotid artery and external carotid artery were exposed through a 2 cm midline incision. Then, a 4-0 surgical nylon suture with a slightly enlarged round tip was inserted into the left internal carotid artery and advanced to the Circle of Willis. The thread was left in place until the rats were sacrificed.

To prepare ischemic brain tissue extracts, rats with similar neurological deficits were sacrificed 48 h after pMCAO^18^. To obtain the same quality of stroke-injured brain extract, we performed the experiments on rats with clear hemiplegic symptoms and neurological deficits in behavioural tests at 48 h. A standard block centered at the territory of the MCAo (bregma −1 ∼ +1 mm) was obtained from the ipsilateral region by dissecting on ice from the ipsilateral region from stroke rat. Normal brain tissue extract were collected from the normal rat brain by same method. Subsequently, tissue pieces were homogenised by adding Dulbecco’s modified Eagle’s medium (DMEM; 150 mg/ml). Tissue homogenates were centrifuged twice for 60 min at 100,000 × 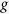 at 4 °C, and then filtered through a 0.2 μm filter, after which protein content was quantified using the Bradford assay. The resulting cell- and MV-free brain extract supernatant was stored at −80 °C.

### Cytokine antibody array

An antibody-based cytokine array system (L Series 90, RayBio^®^ Label-Based Rat Antibody Array 1, RayBiotech, Norcross, GA) was used to detect the levels of cytokines and growth factors in normal and stroke-injured brain extracts. Briefly, normal and stroke-injured brain extract samples were incubated with labelling reagent and washed according to the manufacturer’s instructions. Samples were diluted 1:50 (v/v) in blocking buffer and incubated on pre-blocked arrays overnight at 4 °C. Membranes were then incubated in streptavidin-conjugated peroxidase for 2 h, and exposed to a peroxidase substrate for 5 min before developing on X-ray film. Densitometric analysis was performed on a Kodak ImageStation 4000 M (Eastman Kodak Company, Rochester, NY) with background subtraction from spot edges. Spot data were normalised to a positive control spot on each array.

### Microvesicle isolation from human mesenchymal stem cells

The human MSCs were obtained from adipose tissues of two patients (34- and 41-year old females) undergoing plastic surgery, after written informed consent, in accordance with the Institutional Review Boards (4-2010-0236) of Severance Hospital (Yonsei University Health System, Seoul), were supplied by Yonsei Cell Therapy Center (Yonsei University Health System) and cultured as described previously^57^. The *in vitro* and *in vivo* study was approved by the Institutional Review Board of Yonsei University (approval No. 2011-0087) and Catholic Kwandong University (CKU 01-2014-003). MVs derived from MSC were isolated from conditioned medium using ultracentrifugation as described previously, with minor modifications^10^. Briefly, the primary cultured MSCs (2 × 10^6^ cells/flask) were seeded in 75 cm^2^ culture flasks and cultured in DMEM containing 10% foetal bovine serum for 24 h. After extensive washing with PBS, culture medium was replaced with serum-free, low-glucose DMEM medium containing extract of normal brain tissues or ischemic brain tissues (15 mg/ml). The optimal time point for harvesting microvesicles from cultured MSCs (either untreated or brain-extract—treated) was determined by cell viability based on flow cytometric analysis (Annexin V-PI staining) with the bicinchoninic acid (BCA) assay. We found apoptotic or necrotic bodies are the major contaminating components of microvesicles in conditioned media (data not shown). We therefore harvested microvesicles from cultured MSCs at 48 h for the highest protein contents with minimal apoptotic/necrotic cell death. Conditioned media of untreated MSCs or MSCs treated with normal or ischemic brain extracts were collected at 48 h, centrifuged once at 500 × *g* for 10 min, and then twice at 800 × *g* for 15 min. The supernatants were then concentrated by ultrafiltration using the Minimate TFF capsule system (PALL Corporation, Ann Arbor, MI) with a 100 kDa membrane. To enrich the MVs, the concentrated supernatant was added onto 0.8 and 2.7 M sucrose cushions in 20 mM HEPES/150 mM NaCl buffer (pH 7.2), and subsequently ultracentrifuged twice at 100,000 × *g* for 60 min, which precipitates MVs derived from rat brain^58^,^59^. After centrifugation, the interface between the 0.8 and 2.7 M sucrose cushions was collected and diluted in PBS. For further purification of the MVs, sucrose cushion−enriched MVs were mixed with OptiPrep™ (final concentration = 30%) and then pipetted into the bottom of a 12 ml tube. Next, 3 ml of 20% OptiPrep™ and 2.5 ml of 5% OptiPrep™ were prepared in 20 mM HEPES/150 mM NaCl buffer (pH 7.2) and layered sequentially, and the tubes were then ultracentrifuged at 200,000 × *g* for 3 h. The microvesicles were collected from the interface between 5% and 20% OptiPrep™, diluted with 9 ml of PBS, and then ultracentrifuged at 100,000 × *g* for 1 h. The purified MVs were resuspended in PBS, and the protein concentration of each fraction was determined using refractometry and the Bradford dye assay. All fractions were stored at −80 °C until use.

### *In vivo* experimental protocol

For consistency in data analysis, we excluded rats showing neither hemiplegia nor neurological deficits at 24 h after the pMCAO procedure. Animals were randomised into four groups: PBS alone (*n* = 10), MSC-MVs (*n* = 7), NBE-MSC-MVs (MVs from MSCs treated with normal brain extracts, *n* = 10), and SBE-MSC-MVs (MVs from MSCs treated with stroke-injured brain extracts, *n* = 10). A sham-operated control group (*n* = 5) underwent the same procedure without vascular occlusion. A single injection of MSC-MVs, NBE-MSC-MVs, or SBE-MSC-MVs (0.2 mg/kg of rat body weight) was performed 48 h after pMCAO via the right common carotid artery.

### Behavioural tests and modified neurological severity score (mNSS)

For all animals, a battery of functional tests was performed 24 h before and 24 h after pMCAO and at 1, 3, and 7 days post-MV injection. Before the onset of ischemia, all rats were able to perform the tests and exhibited no abnormalities. All behavioural tests were administered and scored by trained and experienced observers who were blind to the treatment groups of the animals. We assessed the motor weakness of rats subjected to pMCAO with a beam balance test, a torso-twisting test, a prehensile test, a foot fault test, and an open field test^6^. Change in body weight was calculated by subtracting baseline body weight (g) from the body weight measured post-pMCAO.

The modified torso-twisting test was adopted from a previous report^60^. Rats were examined for lateral movements after elevation by their tails up to 10 cm above the surface of the testing area. The frequency of left or right swing was scored for 1 min. The score was 0 for torso twist, left and right swing; 1, asymmetrical twist <30°; and 2, asymmetrical twist >30°. Differential counts for torso-twisting were ipsilateral twist (same as infarct site) and contralateral twist (counter to infarct site). Baseline value of sham control was excluded in torso twisting, as rats without hemiplegic paralysis (as of sham control) display two extremes, either no side swing elevation (score 0) or swing elevation of complete symmetry (score 10~20).

In the beam balance test, rats were placed at the centre of a beam (100 cm × 3 cm × 0.5 cm) and motor performance was graded as a 6-point scale adapted from the previous description^61^. The score was 1, balances with steady posture and paws on top of the beam; 2, grasps side of beam and has shaky movement; 3, one or more paws slip off beam; 4, attempts to balance on the beam but falls off; 5, drapes over the beam but falls off; and 6, falls off the beam with no attempt to balance.

In the prehensile test, a steel bar (2 cm diameter and 100 cm long) placed horizontally 70 cm above a foam rubber pad (7.5 cm thick) was used. After hanging the forepaws of the rat on the bar, the animal was released. Time to falling with rear limb movements was measured. The score 0 was assigned to rats that were able to hang for 5 s and bring rear limbs up, score 1 was assigned to rats hanging for 5 s with no rear limbs up, score 2 was assigned to rats hanging for 3–4 s, and score 3 was assigned to rats hanging for 0–2 s. The foot fault test measures the accuracy of forepaw placement on an equidistant grid (60 cm × 60 cm, 6 cm between grid lines) during 2 min. The open field test consisted of a wooden box (10 cm × 7.5 cm) with walls painted black and the floor painted white, which was divided into twelve squares (2.5 cm × 2.5 cm each). The animals were placed at the centre of the open field arena and tested in a quiet room. The number of lines crossed (at least two paws and nose in a quadrant), rotation (unidirectional circling), and rearing (animal standing upright on its hind legs) were recorded for 2 min.

The neurological severity score (NSS) was a composite of motor, sensory, and reflex test results as described previously^62^. The objective quantifications were based on the torso-twisting test, beam balance test, prehensile traction test, open field test (rotation frequency), and foot fault test. The mNSS score was 0, no deficits; 2, difficulty in fully extending the contralateral forelimb (3 ≤front foot fault <10); 4, unable to extend the contralateral forelimb (front foot fault ≥10); 6, mild circling to the contralateral side (1 ≤rotation or asymmetrical twisting <5); 8, severe circling (rotation or asymmetrical twisting ≥5); and 10, falling to the contralateral side (prehensile traction ≤2).

### Quantification and immunohistochemical assessment of infarct area

Seven days after MV transplantation, rats were anaesthetised with Zoletil (Virbac S.A., France, 25 mg/kg) and perfused with phosphate buffered saline (PBS; pH 7.5). After performing the modified neurological severity score, rats were sacrificed with an overdose of chloral hydrate. Brains were immediately removed and weighed to obtain brain tissue wet weight. The brains were then sectioned into 2-mm-thick coronal slices with the aid of a brain matrix. Coronal brain slices were immediately immersed into 2% 2,3,5,-triphenyltetrazolium chloride (TTC) (Sigma, USA) for 20 min at 37 °C, followed by fixation in a 4% paraformaldehyde solution overnight before analysis. The TTC-stained sections were photographed with a digital camera, and the infarct area of each section was determined with ImageJ software analysis. Indirect lesion area, in which the intact area of the ipsilateral hemisphere was subtracted from the area of the contralateral hemisphere, was calculated. Relative infarct area is presented as the percentage of the indirect lesion versus the contralateral hemisphere.

Each brain was fixed in 4% paraformaldehyde (PFA) for 24 h and washed in PBS. To create paraffin sections, tissues were dehydrated in a graded ethanol series and embedded in paraffin. Each paraffin-embedded brain was sectioned into 4 μm thick layers with a microtome, deparaffinised in xylene for 10 min, and rehydrated in a graded alcohol series. Sections were boiled in 10 mM citric acid for 1 h, followed by the addition of a 5% bovine serum albumin (BSA) solution containing PBS and 0.5% Triton X-100. Then, brain sections were incubated with primary antibodies to doublecortin (DCX; Abcam, 1:100), glial fibrillary acidic protein (GFAP; Millipore, 1:100), and α-smooth muscle actin (αSMA; Abcam, 1:100) for 15–17 h at 4 °C. Sections were incubated overnight with primary antibodies, washed with PBS, and then incubated with fluorescence-tagged secondary antibodies (Alexa-Fluor^®^488 or 594, 1:500, Molecular Probes, Eugene, OR, USA) for 1 h. A fluorescent microscope (Olympus IX71) was used to examine the sections and capture fluorescent images. DCX-positive cells and αSMA positive cells in the hippocampus and the SVZ (cells/mm^2^) were quantified using the MetaMorph Imaging System (Molecular Device, Sunnyvale, CA). The GFAP positive area of the hippocampus image was measured and converted to the volume by multiplying the section thickness (4 *μ*m).

### RT-PCR analysis

Total RNA was extracted using TRIzol reagent. Standard reverse-transcription (RT) was performed using Maxim-RT Premix Kit (Invitrogen). RT-PCR was performed with PCR primers (Bioneer, Daejeon, Korea) under the conditions listed in Supplementary Table S6. The levels of β-actin were used as an internal control.

### Nano-LC-ESI-MS/MS analysis

Briefly, tryptic peptides were applied to a home-made analytical column (75 μm × 11 cm) packed with C18 regular 5 μm sized resin. A linear 45 min gradient was achieved from 97% solvent A (0.1% formic acid in H_2_O) to 60% solvent B (0.1% formic acid in acetonitrile) at a flow rate of 0.3 μl/min. The separated peptide ions were then electrosprayed into the nano-electrospray ionisation (ESI) source. The electrospray voltage was 1.9 kV, and 35% normalised collision energy was used for MS/MS. All MS/MS spectra were acquired by data-dependent scans in which the five most abundant spectra from the full MS scan were selected for fragmentation. The repeat count for dynamic exclusion was set to 1, the repeat duration was 30 s, the dynamic exclusion duration was set to 180 s, the exclusion mass width was 1.5 Da, and the list of dynamic exclusion was 50. Three independent samples of NBE-MSC-MVs were prepared and were subjected to mass spectrometry analysis.

### Mass spectrometry-based proteome analysis

Tryptic peptides produced by in-gel digestion were analysed using a linear trap quadrupole (LTQ) mass spectrometer (Thermo Finnigan, San Jose, CA) coupled with an Eksigent Nano Ultra Performance Liquid Chromatography (uPLC) system (Eksigent Technologies, CA). Three independent samples of NBE-MSC-MVs were prepared and subjected to mass spectrometry. To identify peptides, we first used a free scaffold viewer (http://www.proteomesoftware.com/products/free-viewer/) and MSconverter-3.0.6585 to convert raw spectral data into MGF and mzXML format for use by peptide search algorithms^63^. These MS/MS spectra were searched against the Human Ensembl GRCh37.75^24^ protein database using the MSblender^25^ algorithm, which statistically combines peptide identification scores obtained from multiple search algorithms using multivariate modelling. We ran three separate search algorithms, Comet-2014.01_rev. 0^26^, MSGFDB-v7780^27^, and Myrimatch-2.1.138^28^, against the spectral data. All algorithms were performed using the default parameter settings. Peptide scores from the three search algorithms were integrated with MSblender. We selected a set of proteins for functional proteome analysis by identifying peptides with >1 spectral count in at least one batch, with false discovery rate (FDR) <0.01.

### Gene ontology (GO) analysis and construction of a therapeutic network

We obtained a human Gene Ontology (GO) annotation from NCBI. We manually filtered the identified proteins for annotated genes by GO terms related to angiogenesis, anti-inflammation, neurogenesis, and apoptosis, and generated a list of therapeutic genes. We then connected those genes by links with HumanNet^31^ to generate a therapeutic network composed of 236 therapeutic genes and 1,276 functional associations among them. Visualisation of the network was performed by Cytoscape-3.1.0^25^.

### Database analysis of vesicular proteins

To identify vesicular proteins in the proteome, we used Vesiclepedia^30^, a literature-based database of proteins from exosomes, MVs, and microparticles. We also measured coverage of the identified proteins among the 3,990 MV, microparticle, and exosome proteins of the database.

### Identification of differentially expressed proteins and analysis of their functional coherence based on a human gene network

Differentially expressed proteins (DEPs) between brain-extract—treated and control MSC-MVs were identified using qprot software^64^. Control, untreated MSC-MV proteome data were derived from our previous study^29^. We defined DEPs as those with an FDR threshold of 0.01. To test the functional coherence of our findings, we measured ‘within-group edge counts’ and ‘network neighbours overlap’ among upregulated proteins in the human functional gene network, HumanNet^31^, and a protein-protein interaction network from the Human Protein Reference Database (HPRD)^32^.

### Statistical analysis

Data are expressed as mean ± standard error of mean (SEM) or mean ± standard deviation (SD). Behavioural test and infarct area results were analysed by ANOVA and independent *t*-test using the Statistical Package for Social Sciences (SPSS) version 20. Differences with *P* < 0.05 were considered statistically significant.

## Additional Information

**How to cite this article**: Lee, J. Y. *et al*. Microvesicles from brain-extract—treated mesenchymal stem cells improve neurological functions in a rat model of ischemic stroke. *Sci. Rep.* **6**, 33038; doi: 10.1038/srep33038 (2016).

## Supplementary Material

Supplementary Information
