# Supplementary material for: Microvesicles from brain-extract—treated mesenchymal stem cells improve neurological functions in a rat model of ischemic stroke
Source: Sci Rep. 2016 Sep 9;6:33038. doi: 10.1038/srep33038 (PMC5016792; doi:10.1038/srep33038)
Supplement: Supplementary Information [file srep33038-s1.pdf]

Supplementary Online Information

Microvesicles from brain-extract-treated mesenchymal stem cells improve neurological functions in a rat model of ischemic stroke

Ji Yong Lee, Eiru Kim, Seong-Mi Choi, Dong-Wook Kim, Kwang Pyo Kim, Insuk Lee, and Han-Soo Kim

☐ **Contents**

- **Supplementary Figure S1**
- **Supplementary Figure S2**
- **Supplementary Figure S3**
- **Supplementary Table S1**
- **Supplementary Table S2**
- **Supplementary Table S3**
- **Supplementary Table S4**
- **Supplementary Table S5**
- **Supplementary Table S6**

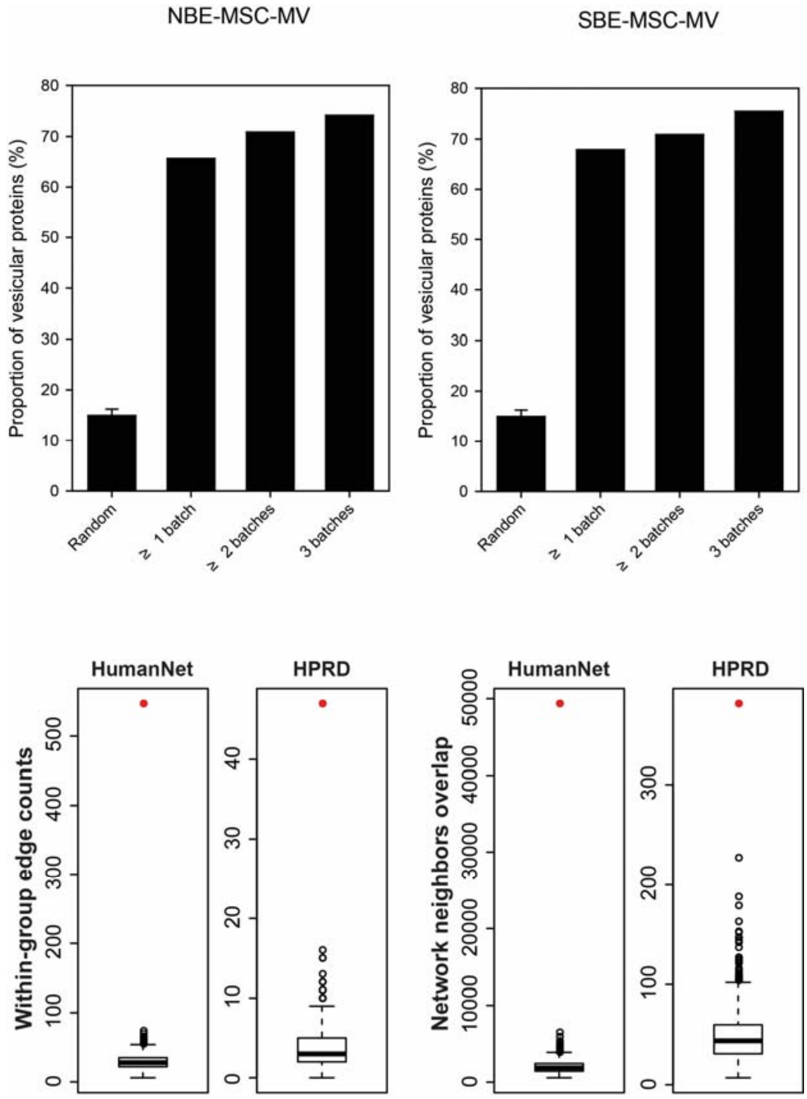

Supplementary Figure S1. **Functional analysis of SBE-MSC-MV proteome.** (A) Both NBE-MSC-MV and SBE-MSC-MV proteomes were enriched for vesicular proteins annotated in the Vesiclepedia database. The degree of enrichment for vesicular proteins was higher for proteins identified in multiple batches. (B) Plots from 'within-group connectivity' and 'sharing network neighbor' analysis of the SBE-MSC-MV proteome, for a functional network, HumanNet, and a protein-protein interaction network, HPRD. Box-and-whisker plots represent 'within-group edge count' and 'network neighbors overlap' scores for 1,000 random gene sets of equal size, and red dots represent scores for the SBE-MSC-MV proteome.

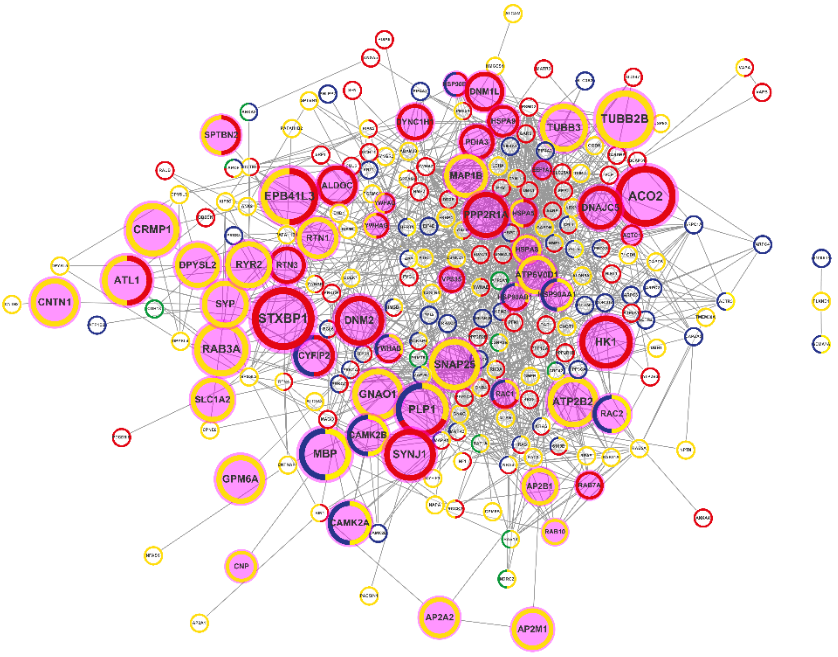

Supplementary Figure S2. A network of SBE-MSC-MV proteins belonging to four functional categories involved in tissue repair. The circumference of each node represents the proportion of proteins from each of the four functional categories: green for angiogenesis, blue for anti-inflammation, yellow for neurogenesis, and red for apoptosis. Upregulated SBE-MSC-MV proteins are shown as purple nodes. The size of the purple nodes represents the fold change.

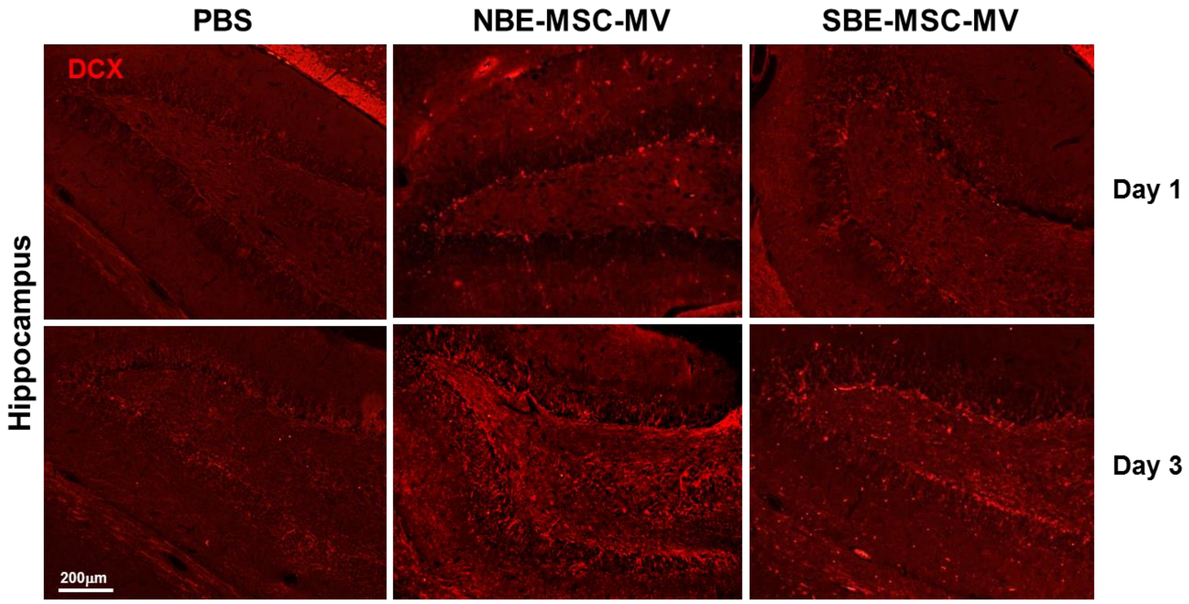

Supplementary Figure S3. S**BE-MSC-MV and NBE-MSC-MV promoted neurogenesis in the hippocampal are in the ischemic rat brain.** Immunostaining of DCX in the hippocampal region of PBS-treated control, NBE-MSC-MV-treated and SBE-MSC-MV-treated rats at Day 1 and Day 3 post-treatment.

Supplementary table S1. A list of identified proteome from NBE-MSC-MV and SBE-MSC-MV by LC-MS/MS upon tissue regeneration using MSblender. Spectral count would not be integer because MSblender distributes 1/N count scores when a spectrum mapped to N peptides

Protein	Gene Symbol	Length of proteins	NBE-MSC-MV batch 1	NBE-MSC-MV batch 2	NBE-MSC-MV batch 3	SBE-MSC-MV batch 1	SBE-MSC-MV batch 2	SBE-MSC-MV batch 3
ENSP00000000233	ARF5	180	8	30	0	12	18	21
ENSP00000000412	M6PR	277	1	1	1	0	1	0
ENSP00000005257	RALA	206	3	7	4	4	7	4
ENSP00000013034	NME1	177	1	4	0	2	2	1
ENSP00000022615	VDAC3	283	1	0	0	0	0	1
ENSP00000050961	ZFHX4	3567	1	0	0	0	0	0
ENSP00000054666	VAMP3	100	15	22	9	13	16	15
ENSP00000078429	GNA11	359	1	1	1	0	0	0
ENSP00000157812	PSMC4	418	1	0	0	0	1	0
ENSP00000164139	PYGM	842	9	9	9	11	20	8
ENSP00000164247	KCNAB2	367	1	0	1	0	0	0
ENSP00000167586	KRT14	472	4	21	18	4	5	3
ENSP00000172853	NEB	6669	1	0	0	0	0	0
ENSP00000193403	ACTN1	892	2	0	0	1	0	0
ENSP00000195649	SNAP91	902	4	1	2	4	0	0
ENSP00000196371	OXCT1	520	1	3	0	0	0	0
ENSP00000198765	CPNE3	537	4	0	0	3	0	0
ENSP00000202028	EPB41L1	779	1	2	0	0	0	1
ENSP00000207457	TEKT2	430	1	0	0	0	0	0
ENSP00000211372	RPS18	152	1	7	0	2	8	1
ENSP00000215095	STX1B	288	34	55	39	22	33	26
ENSP00000215567	TECR	308	1	3	2	1	3	0
ENSP00000215832	MAPK1	360	4	8.5	5	4	5	4
ENSP00000215904	PDXP	296	2	0	0	1	0	0
ENSP00000216121	NIPSNAP1	284	2	3	7	1	5	1
ENSP00000216155	SYNGR1	118	5	3	3	7	2	0
ENSP00000216181	MYH9	1960	2	3	1	0	2	0
ENSP00000216254	ACO2	780	53	41	29	40	61	42
ENSP00000216281	HSP90AA1	732	63.5	85.5	78	68	121	96
ENSP00000216442	ATP6V1D	247	2	7	6	4	9.5	3
ENSP00000216775	CPNE6	557	7	3	1	7.5	3	0
ENSP00000216962	PYGB	843	19	20	21	26	36	24
ENSP00000217133	TUBB1	451	46	94	74	49	77	61
ENSP00000217182	EEF1A2	463	41	47	37.5	44.5	46	29
ENSP00000217420	SLC32A1	525	1	2	1	0	1	0
ENSP00000217426	AHCY	432	2	5	6	4	2	1
ENSP00000217456	APMAP	416	3	0	1	0	0	0
ENSP00000217971	PGRMC1	195	3	4	4	3	3	4
ENSP00000218348	USP11	963	2	0	1	1	1	0
ENSP00000218439	MAGED2	606	1	0	0	1	0	0
ENSP00000218548	ATP12A	1045	22	27	25	18	18	21
ENSP00000218789	ARHGEF7	705	1	0	0	0	0	0
ENSP00000219599	CRYM	314	1	0	0	1	1	2
ENSP00000220325	EHD4	541	1	0	0	0	0	0
ENSP00000220584	FDFT1	417	1	0	0	0	0	0
ENSP00000220931	NCALD	193	3	7	0	3	1	0
ENSP00000221138	PPP2CB	309	5	6	4	4	7	2
ENSP00000221419	HNRNPL	589	1	2	1	0	2	0
ENSP00000221485	SLC17A7	560	8	2	4	6	4	4
ENSP00000222120	RAB3D	219	17.5	20.5	16.5	15	26	24
ENSP00000222256	RAB3A	220	8.5	32.5	28	15.5	34	20
ENSP00000222286	GAPDHS	408	27	36	31	24	39	26

ENSP00000222330	GSK3A	483	2	3	1	0	5	0
ENSP00000222673	OGDH	1023	4	1	0	4	2	1
ENSP00000222812	STX1A	288	19	27	12	8	16	12
ENSP00000223073	RBM28	759	1	0	0	0	0	0
ENSP00000223136	FIS1	152	1	5	0	0	1	0
ENSP00000223836	AK1	210	2	6	0	1	7	3
ENSP00000224784	ACTA2	377	69	141	129	87	120	102
ENSP00000225282	NSF	644	28	46	33	33	45.5	22
ENSP00000225430	RPL19	196	2	2	1	1	5	5
ENSP00000225792	DDX5	614	2	2	2	3	4	0
ENSP00000225899	KRT32	448	1	0	1	0	0	0
ENSP00000226218	VTN	478	1	0	0	0	0	0
ENSP00000226253	ALDOC	364	21.5	36	27	24.5	34	28
ENSP00000226574	NFKB1	969	1	0	0	0	0	0
ENSP00000226760	WFS1	890	3	2	2	1	3	0
ENSP00000226796	GAR1	217	1	0	0	0	0	0
ENSP00000227157	LDHA	241	6	0	1	5	0	2
ENSP00000227378	HSPA8	646	109	189	158	117.5	206	87
ENSP00000228140	RPS13	148	2	6	0	1	2	1
ENSP00000228318	SLC25A3	362	1	3	0	2	1	1
ENSP00000228825	ARPC3	178	2	6	0	2	5	4
ENSP00000229239	GAPDH	335	35	87	65	24	82	72
ENSP00000229264	GNB3	340	6	11	11	9	11	7
ENSP00000229268	USP5	858	2	3	3	3	7	4
ENSP00000229270	TPI1	286	8	19	13	9	17	12
ENSP00000229277	ENO2	434	16	18	14	21	28	14
ENSP00000229319	LDHB	334	11	8	6	15	5	11
ENSP00000229340	RAB35	201	5	8	4.5	7	9	5.5
ENSP00000230461	TMEM30A	361	1	2	1	1	1	0
ENSP00000230588	MEP1A	746	1	0	0	0	0	0
ENSP00000232014	BCL6	706	2	3	2	8	1	1
ENSP00000232447	FLNB	2561	1	1	0	1	0	0
ENSP00000232564	GNB4	340	20	18	18	15	11	7
ENSP00000233114	MDH1	334	5	4	4	4	2	2
ENSP00000233710	ACADL	430	1	0	1	0	0	0
ENSP00000234590	ENO1	434	21	31	20	17.5	32.5	28.5
ENSP00000234981	GSTM1	181	1	5	4	5	4	2
ENSP00000235835	AKR7A2	359	1	1	0	1	0	0
ENSP00000236877	SLC8A2	921	4	4	2	2	0.5	0
ENSP00000236959	ATIC	592	2	1	2	2	0	2
ENSP00000238081	YWHAQ	245	18.5	30.5	26	28.5	23	18
ENSP00000240327	SPOP	374	1	6	5	2	6	11
ENSP00000241001	PAX6	422	1	0	0	0	0	0
ENSP00000241337	GSTM2	218	2	1	1	2	1	0
ENSP00000241704	COPA	1224	1	1	0	3	5	1
ENSP00000242338	CNTFR	372	1	0	1	1	1	0
ENSP00000243052	PDE1B	536	1	0	0	0	0	0
ENSP00000243077	LRP1	4544	7.5	4	3	3	0	2
ENSP00000243706	HAUS3	603	1	0	0	0	0	0
ENSP00000243964	SLC12A5	1116	16	13	18	12	10	9
ENSP00000244295	PSG4	326	1	0	0	0	0	0
ENSP00000244314	IRGC	463	2	5	3	2	4	2
ENSP00000244458	PACSIN1	444	1	4	2	1	2	0
ENSP00000244534	HIST1H1D	221	2	0	0	2	0	1
ENSP00000244751	CPNE5	593	1	0	1	0	1	0
ENSP00000245138	CNOT1	1227	1	0	0	0	0	0
ENSP00000245615	MBOAT7	472	1	0	0	0	0	0
ENSP00000246024	TMX4	349	2	2	1	0	0	0

ENSP00000246069	DSTN	165	1	7	0	3	5	4
ENSP00000246662	KRT9	623	4	124	90	3	28	19.5
ENSP00000246957	TRAP1	704	3.5	4.5	4.5	4	3	5
ENSP00000247207	HSPA2	639	3	8	3	2	5	4
ENSP00000247271	OMG	440	2	1	2	1	1	0
ENSP00000247461	CANX	592	58	34	39	53	43	29
ENSP00000248121	SYNGR3	229	2	3	3	1	1	1
ENSP00000248437	TUBA4A	448	184	331	231	173	278	212
ENSP00000248975	YWHAH	246	3	11	9	7	10	2
ENSP00000249042	TST	297	2	1	3	1	1	1
ENSP00000249071	RAC2	192	16	30.5	8	20	29	21
ENSP00000249364	CALU	315	1	0	0	1	0	0
ENSP00000249700	TMOD2	351	1	0	0	1	0	0
ENSP00000250111	ATP1B2	290	5	6	5	4	5	1
ENSP00000250559	RAP1B	184	3	4	1	3	6	3
ENSP00000251195	CLSPN	1332	1	0	0	0	0	0
ENSP00000251287	HCN2	889	1	0	0	0	0	0
ENSP00000251453	RPS16	146	1	6	0	2	2	1
ENSP00000251535	ALOX12	663	1	0	0	0	0	0
ENSP00000251595	HBA2	142	12	19	6	12	19	14
ENSP00000252242	KRT5	590	3	23	21	3	6	5
ENSP00000252244	KRT1	644	44	231.5	205	34.5	76	62
ENSP00000252245	KRT75	551	1	1.5	3	0	2	1
ENSP00000252486	APOE	317	4	0	0	2	0	1
ENSP00000253413	ATP6V1E1	226	9	21	17	12	16	7
ENSP00000253792	ACLY	1101	1	2	2	1	6	6
ENSP00000253856	ATP6V0A4	840	4	1	1	3	2	2
ENSP00000254035	CKMT2	419	2	3	2	2	1	0
ENSP00000254488	SLC6A11	632	1	0	1	1	0	0
ENSP00000254675	RAN	128	3	7	6	5	6	5
ENSP00000254963	HSPA12B	686	2	0	1	3.5	2	1
ENSP00000254976	SNAP25	206	8	41	30	11	22	15
ENSP00000255283	ATP8A2	1123	2	1	1	0	0	0
ENSP00000255324	RNF17	1623	1	0	0	0	0	0
ENSP00000255448	DCLK1	729	1	2	1	2	2	0
ENSP00000255882	PI4KA	2102	1	0	0	0	0	0
ENSP00000256383	EIF2S1	315	1	0	0	0	2	0
ENSP00000256593	GSTM5	218	1	1	1	2	1	1
ENSP00000256594	GSTM3	225	1	0	0	0	0	0
ENSP00000256637	SORT1	831	1	0	0	0	0	0
ENSP00000256682	ARF3	181	3	19	1	7	15	11
ENSP00000256854	NARS	548	1	4	0	0	1	0
ENSP00000257430	APC	2843	1	0	1	0	1	1
ENSP00000257770	NT5E	574	1	1	1	0	1	0
ENSP00000258091	CCT7	543	7	4	4	8	5	4
ENSP00000258201	FHOD1	1164	1	0	0	0	0	0
ENSP00000258682	CAMK2B	517	12	18	18	8	11	5
ENSP00000258737	RAC1	192	14	22	6	12	12	14
ENSP00000258873	ACSBG1	724	1	1	1	0	1	1
ENSP00000259237	BIN1	409	1	2	2	1	0	0
ENSP00000259238	BIN1	497	1	2	2	2	0	0
ENSP00000259253	UGGT1	1555	2	1	0	3	1	1
ENSP00000259271	GAD2	585	1	2	5	3	8	1
ENSP00000259469	RPL35	123	1	1	1	1	3	1
ENSP00000259727	GMPR	345	1	0	1	0	1	1
ENSP00000259818	TUBB2B	445	142	275.33	204.66	163.5	239.5	189.16
ENSP00000259875	DDR1	876	1	0	0	0	0	0
ENSP00000259891	MOG	295	1	1	1	1	2	0

ENSP00000260570	IFT172	1749	2	2	1	2	0	1
ENSP00000260641	ACTR2	394	3	4	7	6	7	4
ENSP00000260985	IDH1	414	2	3	1	2	3	1
ENSP00000261023	ITGAV	1048	1	1	0	0	0	0
ENSP00000261160	CNTN1	1007	48	40	10	37	44	37
ENSP00000261173	ATP2B1	1220	29	45	28	18	31	22
ENSP00000261183	OSBPL8	889	2	1	1	0	0	0
ENSP00000261192	BCAT1	386	1	1	0	1	0	0
ENSP00000261205	SYT1	422	71	126	109	71	137.5	88
ENSP00000261313	PEBP1	187	2	4	0	1	2	1
ENSP00000261386	GDE1	331	1	1	1	0	0	0
ENSP00000261574	IPO5	1115	1	0	0	0	0	2
ENSP00000261733	ALDH2	517	1	2	3	1	3	0
ENSP00000261735	ERP29	261	3	3	2	3	2	1
ENSP00000261793	CAMK2A	478	11	16	10	7	11	3
ENSP00000261833	CIT	2027	1	0	0	0	0	0
ENSP00000261835	CYP46A1	500	1	4	1	1	0	0
ENSP00000261837	GNB5	395	1	2	2	2	3	1
ENSP00000261845	MAPK6	721	1	2	1	0	1	1
ENSP00000261875	PTPLAD1	362	1	6	1	2	1	4
ENSP00000261890	RAB11A	216	5	14	12	4	12	5
ENSP00000261917	HCN4	1203	1	0	0	0	0	0
ENSP00000261918	SEMA7A	666	1	1	2	1	0	1
ENSP00000261965	TUBGCP3	907	0.5	0	0	0	0	0
ENSP00000262030	ATP5B	529	26	22	19	9	28	15
ENSP00000262189	KMT2C	4911	1	0	0	0	0	0
ENSP00000262225	TMED2	201	2	2	0	0	1	0
ENSP00000262325	AP2B1	937	26	20	16	28	26	22
ENSP00000262384	N4BP1	896	1	0	0	0	0	0
ENSP00000262418	SLC4A1	911	1	0	1	0	0	0
ENSP00000262455	ERP44	406	1	1	0	1	1	0
ENSP00000262461	SLC12A2	1212	1	0	0	0	0	0
ENSP00000262493	GNAO1	354	52	77.5	59	35	55	33
ENSP00000262494	GNAO1	354	24	24	20	15	21	13
ENSP00000262584	RPL8	257	1	1	0	0	0	2
ENSP00000262623	ATP4A	1035	18	33	31	18	29	23
ENSP00000262646	RAB2A	212	4	13	0	2	10	4
ENSP00000262719	PHLPP1	1717	1	0	0	0	0	0
ENSP00000262746	PRDX1	199	1	5	0	1	2	0
ENSP00000263160	SLC17A6	582	1	0	0	1	0	0
ENSP00000263212	PPM1F	454	1	0	0	1	0	0
ENSP00000263233	SYP	313	8	4	6.5	5	4	3
ENSP00000263238	ACTR3	418	1	2	1	0	0	2
ENSP00000263273	NUCB1	461	4	0	0	0	0	0
ENSP00000263354	NAPA	295	2	6	4	4	4	1
ENSP00000263431	PRKCG	697	2	2	3	1	1	3
ENSP00000263519	ATP2B3	1220	3	8	4	1	3	4
ENSP00000263549	PARP12	701	1	0	0	0	0	1
ENSP00000263642	IFIH1	1025	1	0	0	0	0	0
ENSP00000264071	TUBB4A	444	85.33	160.83	130.16	84.5	121.5	98.99
ENSP00000264202	CAPZB	277	1	3	4	1	0	3
ENSP00000264235	GSK3B	420	1	0	1	1	0	0
ENSP00000264335	YWHAE	255	4	6	5	8	4	7
ENSP00000264366	ANK2	3924	6	0	1	3	8	1
ENSP00000264377	ADAM23	832	2	1	2	1	0	0
ENSP00000264441	PCYOX1	293	2	3	3	3	1	1
ENSP00000264449	ATP8A1	1149	10	9	2	6	4	8
ENSP00000264555	PHRF1	1649	1	0	1	1	0	0

ENSP00000264572	RAP1GDS1	516	2	4	4	1	1	2
ENSP00000264638	CNTNAP1	1384	1	6	3	4	3	6
ENSP00000264649	ATP6V0A1	838	27	32	30	21	32	27
ENSP00000264710	RAB10	200	12	22	9	15	20	14
ENSP00000264893	SEPT11	429	4	5.5	11	3	5	3
ENSP00000264908	ANXA3	323	2	0	1	2	0	0
ENSP00000264930	SLC12A7	1083	1	1	2	0	1	1
ENSP00000264932	SDHA	664	1	0	0	0	0	0
ENSP00000265062	RAB7A	207	15	27	7	16	22	13
ENSP00000265112	TARS	723	1	0	0	0	0	0
ENSP00000265113	SLC1A3	542	1	3	2	1	0	0
ENSP00000265175	SEC24B	1268	0.5	0	0	0	0	0
ENSP00000265333	VDAC1	283	7	5	8.5	1	2	2
ENSP00000265462	PRDX5	214	1	4	0	2	1	1
ENSP00000265717	PRKAR2B	418	4	3	3	4	2	3
ENSP00000265732	RBM48	367	1	0	0	0	0	0
ENSP00000265970	PIK3C2A	1686	2	3	1	2	3	1
ENSP00000266027	GNAI2	339	8	10	3	5	5	3
ENSP00000266039	CACNA2D2	1145	1	3	0	5	2	2
ENSP00000266481	DNM1L	699	3	8	3	1	7	5
ENSP00000266991	DHH	396	1	0	0	0	0	0
ENSP00000267484	RTN1	776	12	14	7	9	6	2
ENSP00000267502	RDH12	316	1	0	1	0	0	0
ENSP00000267512	RAB15	208	4	6.5	6.17	6	8.66	3.5
ENSP00000268251	ABAT	500	9	6	8	14	8	6
ENSP00000268613	CDH13	760	3	2	3	1	2	2
ENSP00000268835	PRPSAP2	369	1	0	0	0	0	1
ENSP00000269122	CLTC	1675	132	170.5	144	150.5	161	177
ENSP00000269349	EIF4A3	411	1	2	4	4	2	2
ENSP00000269576	KRT10	584	22.5	99	97.5	18	24	22
ENSP00000269848	PFKL	780	4	7	5	6	9	9
ENSP00000269881	CALR3	384	3	0	0	2	0	2
ENSP00000270776	PGD	483	1	1	3	3	2	0
ENSP00000272065	ACP1	158	1	2	0	0	1	0
ENSP00000272067	ACP1	158	1	1	0	0	1	0
ENSP00000272227	PDIA6	440	4	1	0	3	1	1
ENSP00000272233	RHOB	196	2	5	0	0	0	0
ENSP00000272424	TPRKB	175	1	0	0	0	0	0
ENSP00000272519	RALB	206	1	1	1	0	1	2
ENSP00000272716	SLC4A10	1106	4	6	5	4	2	3
ENSP00000273047	RAB5A	215	7	11.5	10.5	5	11	4
ENSP00000273258	ARL6IP5	188	3	1	0	1	1	0
ENSP00000273398	ATP6V1A	617	27	38	39	32	54	36
ENSP00000273512	NCEH1	440	1	2	1	0	0	1
ENSP00000274695	CDKAL1	579	1	0	0	0	0	0
ENSP00000274938	SCUBE3	993	2	3	1	0	1	0
ENSP00000275364	GNA12	381	9.5	8.5	5.5	7.5	7	4.5
ENSP00000275603	CCT6A	531	3	2	4	4	3	2
ENSP00000276390	ATP6V1B2	511	13	18	17	14	26	17
ENSP00000276651	DPYS	519	5	0	2	5	2	2
ENSP00000277853	CAMK2G	495	1	2	1	1	0	0
ENSP00000277865	GLUD1	558	3	11	6	6	9	5
ENSP00000278193	LIN7C	197	1	0	0	0	0	0
ENSP00000278379	SLC1A2	574	13	16	12	11	7	6
ENSP00000278422	TMX2	296	2	2	0	2	0	0
ENSP00000278572	RPS3	259	2	7	4	3	2	1
ENSP00000278886	NINL	1382	1	0	0	1	0	0
ENSP00000280187	GPM6A	278	10	17	13	6	16	4

ENSP00000280326	CCT5	541	2	1	0	0	3	2
ENSP00000280571	RILPL2	211	1	0	0	0	0	1
ENSP00000280706	LDHAL6A	332	3	5	2	3	2	1
ENSP00000281127	NRXN3	432	2	1	0	0	1	2
ENSP00000281154	SLC25A31	315	7	6	2	1	0	0
ENSP00000281456	SLC25A4	298	2	3	0	0	0	0
ENSP00000281543	GUF1	669	1	0	0	1	0	0.5
ENSP00000281924	TMEM163	289	1	0	0	0	0	0
ENSP00000282050	ATP5A1	553	4	11	15	2	5	5
ENSP00000282382	TMED7-TICAM2	404	2	2	3	1	2	1
ENSP00000282493	PDZD2	2839	2	1	2	3	1	0
ENSP00000282878	RAB3C	227	7	8	6.5	3.5	9	9
ENSP00000283179	HNRNPU	825	1	2	0	0	2	0
ENSP00000283269	CADPS	1314	6	12	6	18	6	12
ENSP00000283351	TRAPPC8	1435	1	0	0	0	0	0
ENSP00000284440	UCHL1	223	1	4	2	0	2	2
ENSP00000284548	OBSCN	6620	3	2	1	2	2	0
ENSP00000284719	OLA1	396	3	5	1	4	2	1
ENSP00000284894	NCAM2	695	2	0	0	1	1	0
ENSP00000285208	RAB6B	208	3	12	3	4	8	3
ENSP00000285379	CA2	260	1	0	0	1	0	0
ENSP00000285930	AKR1B1	316	1	1	1	1	1	1
ENSP00000286091	PDIA4	645	1	0	0	0	0	0
ENSP00000286452	KIF5A	943	2	1	0	3	2	2
ENSP00000286548	GNAQ	359	2	4	3	0	2	0
ENSP00000286788	CCT8	548	2	0	1	3	5	3
ENSP00000287008	PCDH1	1237	1	0	0	0	0	0
ENSP00000287022	UQCRB	111	1	0	0	0	0	0
ENSP00000287226	NPTN	338	5	9	9	6	6	3
ENSP00000288319	ERG	479	1	0	1	0	0	0
ENSP00000288699	DPYSL5	564	3	3	2	4	9	1
ENSP00000289228	ACTR1B	376	1	1	0	4	3	3
ENSP00000289361	BTN3A1	513	1	0	0	0	0	0
ENSP00000289495	PPP1R9A	1296	1	0	0	0	0	0
ENSP00000290101	RAP1GAP	727	1	2	1	0	1	0
ENSP00000290158	KPNB1	876	5	3	4	9	8	5
ENSP00000290216	SCRN2	425	1	0	0	0	0	0
ENSP00000290271	STC1	247	1	0	0	0	0	0
ENSP00000290378	ACTC1	377	17	30	25	16	26	14
ENSP00000290949	ATP6V0D1	351	17	12.5	7	17	8	1
ENSP00000291107	ABR	822	1	2	1	0	3	1
ENSP00000291236	SEPT3	286	1	2	3	2	2	0
ENSP00000292385	DBN1	651	1	3	2	0	1	1
ENSP00000292807	AP2M1	435	5	5	4	8	8	9
ENSP00000292896	HBE1	147	43	49	40	48	55	43
ENSP00000292901	HBD	141	13	27	16	19	21	12
ENSP00000293349	PLEKHH3	790	1	0	0	0	0	0
ENSP00000293590	FMNL3	1028	3	5	6	4	8	4
ENSP00000293748	SYNGAP1	1299	1	0	0	0	0	0
ENSP00000293831	EIF4A1	406	3	4	3.5	1	6	2
ENSP00000293842	RPL26	145	1	2	0	3	0	0
ENSP00000294016	ADCY9	1353	1	0	0	0	1	0
ENSP00000294623	FUBP1	653	1	0	0	0	1	0
ENSP00000294724	AGL	1532	1	0	0	1	0	0
ENSP00000294742	ARPC5	154	1	1	0	1	0	0
ENSP00000295101	KCNJ3	501	1	0	0	0	0	0
ENSP00000295156	VSNL1	191	7	12	1	8	7	6
ENSP00000295266	PDHA2	388	1	2	2	0	1	0

ENSP00000295598	ATP1A1	1023	101	132	90	87	93	71.5
ENSP00000295685	ARPC2	300	2	5	6	3	4	2
ENSP00000295688	CCT3	545	5	7	6	5	12	3
ENSP00000295901	PSMD6	389	2	0	0	1	0	0
ENSP00000295987	SYN1	705	29	74	46	26	62	34
ENSP00000295989	CAND2	1119	1	0	0	0	0	1
ENSP00000296121	KIAA1143	154	1	0	0	0	0	0
ENSP00000296122	PPP1CB	327	3	3	3	1	4	1
ENSP00000296402	CAMK2D	478	11	15	16	8	10	5
ENSP00000296425	PGRMC2	223	1	2	0	0	1	1
ENSP00000296435	CAMP	173	1	0	0	0	0	0
ENSP00000296464	HSPA4L	839	1	1	4	0	7	3
ENSP00000296471	CAMKV	473	3	5	4	2	4	3
ENSP00000296479	GRM2	435	1	0	0	1	1	0
ENSP00000296511	ANXA5	320	7	3	3	3	0	1
ENSP00000296741	ENPP6	440	3	2	2	5	1	1
ENSP00000296755	MAP1B	2468	8	4	5	9	9	10
ENSP00000296869	ACSL6	722	1	3	3	0	5	0
ENSP00000297185	HSPA9	679	4	6	7	5	12	4
ENSP00000297268	COL1A2	1366	2	1	0	0	0	0
ENSP00000297283	PGAM2	253	10	14	14	8	10	7
ENSP00000298049	EEF1A2	463	8	12	13	19.5	10	11
ENSP00000298159	CFL2	166	2	9.5	1	3	6	8
ENSP00000298468	VDAC2	282	2	2	0	0	0	0
ENSP00000298556	HPRT1	218	1	3	3	1	3	0
ENSP00000298649	HK1	916	9	5	9	12	15	10
ENSP00000299138	VPS35	796	8	9	8	9	15	9
ENSP00000299198	CKB	381	31	29	27	39	45	33
ENSP00000299300	CCT2	535	5	5	7	5	8	4
ENSP00000299518	IDH3A	366	19	11	9	15	9	8
ENSP00000299767	HSP90B1	803	32	16	18	31	23	13
ENSP00000300026	PPIB	216	4	7	1	3	3	0
ENSP00000300060	ANPEP	967	6	6	1	0	2	3
ENSP00000300161	YWHAB	246	10	23	17	12	24	9
ENSP00000300231	MAP1A	2803	1	2	3	1	2	0
ENSP00000300249	MAPRE2	327	2	2	2	5	1	0
ENSP00000300283	CKMT1B	417	3	3	2	2	6	4
ENSP00000300289	PDIA3	505	17	13	15	16	11	15
ENSP00000300737	STIM1	685	1	0	0	0	0	0
ENSP00000300935	RAB8A	207	1	1	0	1	1	1
ENSP00000301037	SGK494	410	1	0	0	0	0	0
ENSP00000301071	TUBA1A	451	78	134	92	88	127.5	99
ENSP00000301149	GPD1	349	1	1	2	1	1	2
ENSP00000301387	ATP2A3	1043	9	12	12	5	12	9
ENSP00000302088	RER1	177	1	2	1	0	0	0
ENSP00000302393	LDHAL6B	381	1	1	0	1.5	1	1.5
ENSP00000302397	ATP1A3	1013	117	120	81	90.5	95.5	93.5
ENSP00000302486	MAP2K1	393	3	6	7	6	5	3
ENSP00000302777	TUBB3	378	36.5	100	82	38	70	65
ENSP00000302886	PA2G4	394	1	0	2	0	1	1
ENSP00000302961	HSPA4	840	5	11	6	12	23.5	7
ENSP00000303145	TMED10	219	1	2	0	0	2	2
ENSP00000303909	ABR	859	1	2	1	0	3	1
ENSP00000304283	RAC3	192	1	3	0	3	2	7
ENSP00000304410	C2orf68	166	1	0	0	0	0	0
ENSP00000305152	PLP1	277	54	97	57.5	69	79	56
ENSP00000305263	KRT28	464	2	6	4	1	0	0
ENSP00000305355	PRKCB	673	4	0	2	2	4	6

ENSP00000305449	CNNM3	707	1	0	0	0	0	0
ENSP00000305938	NEGR1	226	3	0	1	1	1	0
ENSP00000305995	PGK2	417	20	13	19	20	20	13
ENSP00000306124	PRKCE	737	1	0	0	0	1	0
ENSP00000306253	ITPR1	2743	5	5	2	5	1	4
ENSP00000306306	TGFBI	683	2	1	2	0	0	0
ENSP00000306330	YWHAG	247	25	31	29	28	23	16
ENSP00000306496	RAB33B	229	2.5	4	2.67	6.5	7.16	3
ENSP00000306822	AVEN	362	1	0	0	0	0	0
ENSP00000307241	PDHB	359	5	6	5	3	0	0
ENSP00000307334	AUH	310	2	3	6	0	3	0
ENSP00000307341	SNAP25	206	13	38	31	13	24	13
ENSP00000307900	GLUL	373	9	6	3	9	8	3
ENSP00000307940	EEF2	858	2	6.5	6	11	15	11.5
ENSP00000308495	KRAS	188	1	1	0	0	1	1
ENSP00000309066	DPP10	789	1	0	1	0	1	0
ENSP00000309431	RP11-683L23.1	444	3	18	15	5	10	12
ENSP00000309438	PFKM	780	5.5	9	6	8.5	19	10
ENSP00000309477	ACO1	889	4	2	1	3	3	1
ENSP00000309503	YWHAZ	245	7	7	6	13	10	5
ENSP00000309539	DPYSL2	572	63	92	75	71	133	98
ENSP00000309629	CFL1	166	1	3	0	3	2	3
ENSP00000310040	EIF3F	357	1	1	0	0	1	0
ENSP00000310129	PSMD2	908	1	0	2	1	3	2
ENSP00000310219	HSPA6	643	3	5	8	3.5	5.5	1
ENSP00000310226	RAB1B	201	4	9	1	5	10	11
ENSP00000310587	NCALD	193	2	7	0	2	1	0
ENSP00000310829	TTC18	1121	1	0	0	0	0	0
ENSP00000310861	KRT2	639	12	54	48	17	8	11
ENSP00000311028	RPS14	151	1	3	0	0	1	1
ENSP00000311186	ATP2A2	997	40.5	71	52	38	56	49
ENSP00000311344	PPP2R1B	667	1	2	1	1	2	1
ENSP00000311489	SPTBN2	2390	3	4	1	5	6	10
ENSP00000313050	GBAS	286	1	3	4	0	1	0
ENSP00000313129	TARDBP	295	2	1	2	0	0	0
ENSP00000313164	DNM2	870	10	7	7	9	17	7
ENSP00000313567	CYFIP2	1253	7	22	6	12	20	16
ENSP00000314458	CDC42	191	2	6	3	1	7	2
ENSP00000314649	ALDH5A1	548	1	1	2	0	2	2
ENSP00000314831	KCTD1	257	1	0	1	1	2	0
ENSP00000315662	IQSEC3	1182	1	0	0	0	0	0
ENSP00000315931	AHCYL2	611	4	6	5	3	4	0
ENSP00000316291	RBBP6	1758	1	1	0	0	0	0
ENSP00000316357	USP9X	2570	4	4	2	2	4	5
ENSP00000316664	IGSF8	613	1	0	0	0	1	1
ENSP00000316861	GPM6B	328	4	11	13	2	6	6
ENSP00000317334	TCP1	556	2	2	3	0	5	2
ENSP00000317379	GLS	669	1	7	3	0	4	3
ENSP00000317788	HNRNPK	463	1	1	1	3	5	1
ENSP00000318472	NCAM1	848	20	24	14	13	15	16
ENSP00000318575	TUBA8	383	16	17	12	18	14	10
ENSP00000318687	HSPH1	858	1	0	1	6	7	1
ENSP00000318697	TUBB6	446	16.83	27.83	26.16	14.5	26	22.49
ENSP00000318845	SYNGR1	191	4	9	11	3	8	3
ENSP00000319281	BASP1	227	7	6	6	3	5	0
ENSP00000319361	AP1B1	919	2	0	0	6	0	0
ENSP00000319578	DDX47	406	2	2	1	2	3	0
ENSP00000319739	RCN2	335	1	0	0	2	0	0

ENSP00000320017	PHYHIP	330	2	3.5	3	1	2	2
ENSP00000320171	PKM	531	43	43	35	42	81	57
ENSP00000320291	OSBPL1A	950	1	1	0	1	1	0
ENSP00000320295	TUBB3	450	5	11	10	6	13	9
ENSP00000320324	NPEPPS	919	2	0	0	2	2	2
ENSP00000320516	EHD1	534	2	4	3	3	4	1
ENSP00000320580	PPP3CA	469	4	5	8	7	4	0
ENSP00000320866	CALR	417	17	8	7	17	5	5
ENSP00000321320	FAM203A	390	1	0	0	0	0	0
ENSP00000321343	ST8SIA5	376	1	0	0	0	0	0
ENSP00000321606	CRMP1	686	8	9	7	5	21	9
ENSP00000321617	ANKZF1	726	1	0	0	0	0	0
ENSP00000321703	TACC1	805	1	0	1	0	0	0
ENSP00000321753	C7orf63	895	1	0	0	0	0	0
ENSP00000322147	RTN4	373	3	4	0	2	0	0
ENSP00000322234	SYNJ1	1573	14	22	2	15	28	21
ENSP00000322421	HBA1	142	12	19	7	13	18	15
ENSP00000322832	ROGDI	287	1	1	1	1	0	1
ENSP00000323032	ZNF318	1117	1	0	0	0	0	0
ENSP00000323339	C2CD3	1963	1	0	0	0	0	0
ENSP00000323856	PLEC	4684	1	0	0	1	0	0
ENSP00000324011	XPNPEP1	642	2	2	4	2	4	1
ENSP00000324105	ENO3	434	2	11	7	9	11	7
ENSP00000324172	ATP2B2	1243	12	13	12	9	15	9
ENSP00000324173	HSPA5	654	39	27	26	24	28	7
ENSP00000324287	ACAP2	778	1	0	0	0	0	0
ENSP00000324549	CYFIP1	1253	0.5	0.5	0	2	1.5	1
ENSP00000324628	NAPG	312	5	2	3	2	0	0
ENSP00000324804	PPP2R1A	589	11	12	12	16	15	8
ENSP00000325002	COPG1	874	1	0	1	0	1	0
ENSP00000325074	PPP2R2A	457	2	3	2	4	6	2
ENSP00000325136	HADHB	474	1	0	0	0	1	0
ENSP00000325452	KIF26A	1743	1	0	0	0	0	0
ENSP00000325660	CNTN1	1018	4	2	0	7	2	1
ENSP00000325817	CYFIP2	1278	1.5	1	0	3	1.5	1.5
ENSP00000325836	DIRAS1	198	1	0	0	0	0	0
ENSP00000325875	HSP90AB1	724	27	40	30.5	34.5	62	46
ENSP00000326381	EIF4A2	407	1	3	1.5	0	2	3
ENSP00000326777	MTHFSD	382	1	0	0	0	0	0
ENSP00000327070	MDH2	338	10	11	7	13	8	5
ENSP00000327116	EHD3	535	1	0	0	0	0	0
ENSP00000327137	ARPC2	300	2	5	5	2	3	2
ENSP00000327145	FLNC	2725	1	0	0	0	0	0
ENSP00000327539	HNRNPH1	472	1	4	5	1	4	1
ENSP00000327589	GLUD2	558	3	7	5	4	7	4
ENSP00000327694	AP2A2	940	13	18	13	12	32	13
ENSP00000327801	P4HB	508	2	0	0	3	1	0
ENSP00000328226	DPP6	803	7	1	1	0	3	0
ENSP00000328336	CEND1	149	1	0	0	0	0	0
ENSP00000328455	LSAMP	345	16	9	12	15	12	4
ENSP00000329568	NLRP7	1009	1	0	0	0	0	0
ENSP00000330054	EEF1A1	462	27	51	43	43	58	36
ENSP00000330101	KRT76	638	2	0	0	2	0	0
ENSP00000330862	OPCML	345	6	6	5	7	7	4
ENSP00000331428	CMTM1	122	1	0	0	0	0	0
ENSP00000331514	ACTG1	375	53	87	70	52.5	85	55
ENSP00000331636	CAPN12	719	1	0	0	1	0	0
ENSP00000331901	EEF1G	437	1	5	3	1	6	1

ENSP00000332287	SYNGR1	233	5	4	3	4	2	0
ENSP00000332818	SV2B	683	2	3	1	0	0	0
ENSP00000333255	VMA21	101	1	0	1	1	0	0
ENSP00000333393	ATL2	566	3	4	3	0	1	2
ENSP00000333547	RAB11B	218	5	15	13	4	12	6
ENSP00000333994	HBB	147	9	15	6	11	15	5
ENSP00000334234	SEPT10	321	1	0	1	0	1	0
ENSP00000334379	C2CD3	2353	1	0	0	0	0	0
ENSP00000335153	HSP90AA1	854	3	0	2	3	3	2
ENSP00000335323	ACACA	2268	2	0	1	0	0	0
ENSP00000336604	KRT13	420	1	5	6	1	2	1
ENSP00000336744	GSTM4	157	1	1	1	0	1	1
ENSP00000336799	TUBA1B	451	70	111	75	76	101.5	67
ENSP00000336831	PLS1	629	3	1	1	2	1	1
ENSP00000336850	RAB6A	208	7.5	17	10.83	6	11.5	6.5
ENSP00000336861	IQCH	1027	1	0	0	0	0	0
ENSP00000336927	ALDOA	364	5	11	11	8	15	11
ENSP00000338082	HBG2	147	3	0	2	5	2	2
ENSP00000338349	NRXN3	1061	3	1	1	2	2	1
ENSP00000338413	UBA1	1058	11	18	5	12	22	14
ENSP00000338461	CYB5R3	301	2	1	1	0	0	0
ENSP00000338568	ATP5C1	297	1	1	1	0	0	0
ENSP00000338718	ATXN2L	1075	1	0	0	1	0	0
ENSP00000338934	EZR	586	2	0	0	0	1	0
ENSP00000338964	GGT7	662	1	1	2	2	3	4
ENSP00000339001	TUBB	444	24	39	27	20	31.5	26
ENSP00000339084	AKR7A2	314	1	1	0	1	0	0
ENSP00000339191	CAV1	178	2	1	0	0	0	0
ENSP00000339729	FSCN1	472	15	10	14	12	10	11
ENSP00000340019	HSPD1	573	3	2	2	2	8	3
ENSP00000340211	CORO1B	489	1	1	0	0	1	0
ENSP00000340454	RAP1GDS1	608	4	4	5	3	2	3
ENSP00000340466	GANAB	966	12	5	3	16	5	4
ENSP00000340554	TTN	27118	2	4	3	2	3	2
ENSP00000340610	POTEH	545	1	0	0	1	0	0
ENSP00000340716	RTN1	208	15.5	24	8	9.5	10	3
ENSP00000340766	SMG7	1137	1	0	0	0	0	0
ENSP00000340815	SLC3A2	529	3	0	0	2	1	1
ENSP00000340903	RTN3	241	8.5	7	5	5.5	8	3
ENSP00000340930	ATP2B4	1170	3	1	0	2	1.5	1
ENSP00000340989	SFN	248	1.5	2.5	3	3.5	4	2
ENSP00000341072	CDC42	191	2	3	0	8	3	2
ENSP00000341138	EPB41L3	1087	20	15	12	18	25	13
ENSP00000341214	HIST1H1T	207	2	1	0	1	0	0
ENSP00000341289	TUBB4B	445	366	628.5	495.5	366.5	478	361
ENSP00000341408	ABHD12	398	1	2	3	0	1	0
ENSP00000341524	SEPT6	434	1	1	2	0	2	1
ENSP00000341848	GOLGB1	3259	2	0	0	0	0	0
ENSP00000341885	RPS2	293	2	2	3	1	0	0
ENSP00000341917	ENG	625	1	0	1	1	0	1
ENSP00000341988	SPATA31D1	1576	1	0	0	0	0	0
ENSP00000342026	PRDX6	224	1	4	3	1	3	1
ENSP00000342056	CS	466	5	10	8	7	7	3
ENSP00000342128	NFASC	1257	3	0	1	2	2	1
ENSP00000342710	KRT77	578	4	18	14	3.5	10	7
ENSP00000342711	LY6H	161	1	1	0	1	0	0
ENSP00000342951	ATP6V0A1	837	40	41	33	28	39	36
ENSP00000342958	NPTN	282	7	6	2	6	3	1

ENSP00000343027	GNAI1	354	3	4	6	1	4	1
ENSP00000343040	HMGB1	215	1	0	2	0	1	0
ENSP00000343158	EPB41L3	1087	1	1	1	1	3	1
ENSP00000343206	SYN1	669	9	13	10	11	15	10
ENSP00000343458	BCAP31	246	2	3	3	1	2	0
ENSP00000343463	MAP3K2	619	1	1	1	0	0	0
ENSP00000343599	ATP2B1	1184	16	28	18	10	18	12
ENSP00000343650	TTC18	361	1	0	0	0	0	0
ENSP00000343690	DPYSL3	684	3	2	3	3	8	3
ENSP00000343990	TRIM14	442	1	0	0	0	0	0
ENSP00000344002	FLNC	2692	1	0	0	0	0	0
ENSP00000344106	RTN3	1013	9	17	12	10	10	9
ENSP00000344403	FSIP2	6996	1	0	0	0	0	0
ENSP00000344677	ATP2B2	1229	8	5.5	4	5	6	3
ENSP00000344868	SEPT7	436	9	20.5	19	13	16	9
ENSP00000345461	CACNB1	523	1	0	0	0	0	0
ENSP00000345656	VAPA	294	1	5	5	4	3	0
ENSP00000345680	DNM1	851	41	61	66	52	89	75
ENSP00000346032	ANXA2	357	6	10	6	5	8	3
ENSP00000346067	RPSA	295	3	5	5	4	2	3
ENSP00000346240	FBXO2	296	1	2	0	1	1	0
ENSP00000346246	AP2A1	955	4	5	4	2	7	6
ENSP00000346328	GNAS	395	2.5	3.5	4.5	3.5	2	1.5
ENSP00000346522	ATL1	553	10	9	8	15	8	10
ENSP00000346545	MBP	73	10	12	2	12	9	4
ENSP00000346550	ANXA6	673	5	1	3	7	5	4
ENSP00000346809	PGAP1	922	2	0	1	1	0	0
ENSP00000347359	ATP6V1H	465	8	7	11	11	8	7
ENSP00000347457	DNM3	869	2.5	2	6	5	6	2
ENSP00000347665	COL18A1	1519	1	0	0	0	0	0
ENSP00000347744	FAM49A	323	1	1	0	0	0	0
ENSP00000347890	DNM2	870	1	1	1	4	5	3
ENSP00000348062	PDHA1	203	6	7	6	4	5	2
ENSP00000348273	MBP	304	12	16	10	13	16	12
ENSP00000348279	RTN3	255	1	4	5	3	2	2
ENSP00000348394	NCDN	729	17	21	18	23	27	17
ENSP00000348463	TMEM236	351	1	0	0	1	0	0
ENSP00000348546	RAB1A	173	1	2	0	1	1	2
ENSP00000348589	CACNA2D1	1103	12	16	0	21	18	18
ENSP00000348693	MYO5A	1828	3	3	4	1	3	2
ENSP00000348702	PLEC	4533	1	0	0	1	0	0
ENSP00000348965	DYNC1H1	4646	59	84	53	85	98	77
ENSP00000349053	GANAB	944	5	0	1	5	2	2
ENSP00000349168	HNRNPH1	449	1	3	2	1	3	1
ENSP00000349238	CCDC88B	1476	9	3	4	8	5	6
ENSP00000349320	CACNA2D1	1091	2	5	0	4	5	6
ENSP00000349534	ATL1	558	1	4	3	6	1	2
ENSP00000349588	ANK2	3957	1	0	0	2	2	0
ENSP00000349658	XPNPEP3	507	1	0	0	0.5	2	1
ENSP00000349748	SFPQ	707	1	0	0	0	0	0
ENSP00000349892	MYCBP2	4640	1	0	0	0	0	0
ENSP00000349903	SYNJ1	1295	1	4	3	5	5	6
ENSP00000350310	ATP2B4	1205	1	4	1	2	2	1
ENSP00000350364	NEGR1	354	5	1	2	1	2	1
ENSP00000350447	SGOL2	1265	1	0	1	1	1	0
ENSP00000350469	AMER2	552	2	2	1	2	1	1
ENSP00000350632	CADPS	1274	2	3	4	9	4	10
ENSP00000350876	DNM3	863	3.5	1	3	5	1	2

ENSP00000350882	SPTAN1	2477	2	1	0	6	4	6
ENSP00000350974	PPP1CA	286	4	8	7	1	6	1
ENSP00000351155	ATL1	558	11	11	9	15	10	9
ENSP00000351483	SLC4A8	1071	1	0	0	0	0	1
ENSP00000351617	IQCH	696	1	0	0	0	0	0
ENSP00000351777	VCP	806	3	3	2	8	6	8
ENSP00000351926	AP2A1	977	6	5	6	3	7	5
ENSP00000352228	PCBP2	331	4	4	3	4	3	1
ENSP00000352398	HK1	917	10	9	10	12	19	13
ENSP00000352522	ATP6V1H	483	2	1	2	3	2	1
ENSP00000352842	PFKM	780	3	0	1	5	3	1
ENSP00000352918	ARPC5	151	1	1	0	1	0	0
ENSP00000353072	ATP2A3	1052	2	0	1	0	1	1
ENSP00000353151	HOXA4	320	2	3	1	3	2	2
ENSP00000353174	RYR2	4973	11	15	8	10	13	7
ENSP00000353433	HK1	905	3	1	1	2	3	1
ENSP00000353785	TPPP	219	3	2	0	4	0	1
ENSP00000353846	DCLK1	740	1	2	1	2	2	0
ENSP00000354111	DNAJC5	198	16	20	12	12	12	6
ENSP00000354357	GSTM3	225	1	0	0	0	0	0
ENSP00000354451	IQGAP3	1631	8	6	4	5	6	4
ENSP00000354468	CYB5R3	334	2	1	1	0	0	0
ENSP00000354490	ATP1A2	1020	37.5	34.5	26	23.5	20.5	24.5
ENSP00000354615	ACOT7	370	1	3	4	2	2	4
ENSP00000354635	AGL	1515	1	0	0	1	0	0
ENSP00000354644	ARHGEF11	1522	3	1	1	1	0	0
ENSP00000354712	L1CAM	1248	1	0	0	0	2	0
ENSP00000354777	TBKBP1	615	1	0	0	0	0	0
ENSP00000354778	CNTNAP2	1331	2	1	1	0	1	0
ENSP00000354791	DCTN1	1278	2	1	1	0	5	4
ENSP00000354947	CAPZA2	286	1	2	2	1	2	1
ENSP00000355146	GPRASP1	1395	1	0	0	0	0	0
ENSP00000355311	OPA1	979	2	0	0	1	0	0
ENSP00000355387	SCAMP5	235	3	2.5	0	0	2	0
ENSP00000355493	ADSS	456	1	0	0	1	0	0
ENSP00000355560	TBCE	527	1	0	0	0	0	0
ENSP00000355720	JMJD4	463	1	0	0	0	0	0
ENSP00000355752	C1orf95	141	1	2	1	1	0	0
ENSP00000356137	NFASC	1071	1	0	0	2	1	1
ENSP00000356141	NFASC	1041	1	0	0	1	0	0
ENSP00000356236	SYT2	419	6	9	9	1	6	3
ENSP00000356237	SYT2	419	7	5	7	2	3	2
ENSP00000356537	GLUL	373	3	1	0	3	1	0
ENSP00000356787	ATP1B1	295	26	25	24	24	19	13
ENSP00000356789	ATP1B1	303	23	26	23	20	21	15
ENSP00000356790	ATP1B1	303	1	1	1	1	2	2
ENSP00000357060	ATP1A4	1029	79.5	91.5	70.5	66.5	59	61.5
ENSP00000357065	IGSF8	613	2	0	0	1	1	1
ENSP00000357106	CADM3	432	1	0	1	0	0	0
ENSP00000357177	ARHGEF11	1562	3	1	1	1	0	0
ENSP00000357244	CCT3	500	3	8	8	6	14	3
ENSP00000357251	SOGA3	947	1	0	0	1	0	2
ENSP00000357564	RAB13	203	2	2	3	1	5	6
ENSP00000357609	STK32C	276	1	0	0	0	0	0
ENSP00000357624	MARCKS	332	10	12	11	13	15	10
ENSP00000357722	S100A8	93	1	0	0	0	0	0
ENSP00000358124	VPS45	538	1	0	0	0	1	0
ENSP00000358141	SV2A	682	14	6	9	10	11	9

ENSP00000358159	ASCC3	2202	1	1	0	0	0	1
ENSP00000358211	HSPA12A	675	11	15	9	14	22	8
ENSP00000358224	PPIAL4C	164	1	3	0	0	2	1
ENSP00000358415	PHGDH	499	1	3	2	1	4	2
ENSP00000358417	PHGDH	533	1	0	1	1	3	2
ENSP00000358508	ATP1A1	992	61	49	39	51	32	25
ENSP00000358628	MAGI3	1125	1	2	1	0	1	0
ENSP00000358630	MAGI3	1150	1	2	2	0	2	1
ENSP00000358656	RHOC	193	1	1	0	1	0	0
ENSP00000358812	PDCD11	1871	1	1	2	1	1	0
ENSP00000358814	AHCYL1	530	2	6	5	2	3	0
ENSP00000358827	GSTM5	237	1	0	1	2	0	1
ENSP00000358846	GSTM2	220	2	4	4	5	4	0
ENSP00000358921	ACTR1A	376	8	3	3	9	4	1
ENSP00000358990	IRAK1	339	1	0	0	0	0	0
ENSP00000358992	MYO6	1253	1	0	0	0	0	0
ENSP00000358994	MYO6	1285	1	0	0	0	0	0
ENSP00000359345	RPL5	297	1	0	0	0	0	1
ENSP00000359539	GOT1	413	2	0	0	2	1	0
ENSP00000359991	PGAM1	254	7	17	14	9	11.5	9
ENSP00000360124	PGM1	580	3	3	6	3	7	2
ENSP00000360467	LGI1	291	2	1	1	0	1	0
ENSP00000360609	HSP90AB1	724	13	9	16	21	23.5	16
ENSP00000360626	KCNG1	513	3	3	2	2	2	2
ENSP00000360671	SLC25A5	298	3	1	0	0	0	0
ENSP00000361057	SURF4	269	1.5	1	0	0	0	1
ENSP00000361071	RPL7A	151	2	1	2	1	0	0
ENSP00000361495	PRPS1	218	1	3	0	2	0	0
ENSP00000361505	PRPS1	251	1	0	0	0	0	0
ENSP00000361816	SPTAN1	2472	1	0	0	3	0	0
ENSP00000361930	YWHAB	246	7	14	13	9	15	7
ENSP00000361965	ADA	363	1	0	0	0	0	0
ENSP00000361990	UBR2	1755	1	0	0	0	0	0
ENSP00000361992	UBR2	1755	1	0	0	0	0	0
ENSP00000362010	ANXA7	488	1	0	0	1	0	0
ENSP00000362014	DNM1	864	1	0	1	0	3	1
ENSP00000362249	AK1	194	1	3	0	0	0	0
ENSP00000362271	AK1	194	2	3	0	1	4	2
ENSP00000362317	CLSPN	1275	1	0	0	0	0	0
ENSP00000362340	NCDN	729	19	27	25	26	34	23
ENSP00000362350	NCDN	712	6	13	8	5	12	6
ENSP00000362396	STXBP1	594	69	108	93	66	116	77
ENSP00000362399	STXBP1	603	20	23	23	18	27	16
ENSP00000362413	PGK1	417	26	41	36	30	47	31
ENSP00000362566	HPCA	193	3	4	0	2	3	3
ENSP00000362638	MARCKSL1	195	2	5	3	3	5	1
ENSP00000362744	RPS4X	263	1	4	5	0	0	0
ENSP00000362946	RAB14	215	5	16	7	6	15	4
ENSP00000362985	PHYHIPL	350	1	2	2	1	1	1
ENSP00000362987	PHYHIPL	376	1	3	0	0	0	0
ENSP00000363057	EPB41L1	701	2	2	0	1	0	1
ENSP00000363216	OGDHL	1010	1	0	0	1	0	0
ENSP00000363362	KIAA1958	738	1	0	0	0	0	0
ENSP00000363500	CLIC4	253	2	4	1	0	3	2
ENSP00000363537	ADAM23	832	1	0	0	1	0	0
ENSP00000363626	LYPLA2	180	1	1	1	1	1	1
ENSP00000363638	LYPLA2	231	1	2	2	1	0	1
ENSP00000363910	OPCML	304	5	5	3	6	5	4

ENSP00000363916	NTM	316	3	2	1	3	1	2
ENSP00000363988	ALDOB	364	1	0	0	2	0	6
ENSP00000364145	PFKFB1	471	1	0	0	0	0	0
ENSP00000364209	MAGED2	606	1	0	0	0	0	0
ENSP00000364239	TRIM14	442	1	0	0	0	0	0
ENSP00000364298	FKBPL	349	2	1	0	0	2	0
ENSP00000364345	EMC1	992	1	2	0	0	2	0
ENSP00000364365	UBR4	5176	1	0	0	0	0	0
ENSP00000364416	UBR4	5204	1	0	0	0	0	0
ENSP00000364691	CROCC	2017	1	0	0	0	0	0
ENSP00000364883	AUH	339	2	3	6	0	2	0
ENSP00000364912	SPEN	3664	1	0	0	0	0	0
ENSP00000364919	DIRAS2	199	1	8	4	1	3	1
ENSP00000364960	RAB18	161	1	7	0	1	3	3
ENSP00000365158	UGGT1	1531	1	1	0	1	0	0
ENSP00000365437	GAD2	585	1	2	5	3	7	1
ENSP00000365439	HNRNPK	464	1	1	1	3	5	1
ENSP00000365572	NME2	197	1	0	0	1	0	0
ENSP00000365687	PTF1A	328	2	0	0	1	0	0
ENSP00000366084	ABCC4	1325	1	0	0	0	0	0
ENSP00000366225	NAPB	298	9	9	7	8	9	3
ENSP00000366260	CNNM3	659	1	0	0	0	0	0
ENSP00000366311	USP11	920	2	0	1	1	1	0
ENSP00000366326	NEBL	1014	1	0	0	0	0	1
ENSP00000366481	UBA1	506	8	9	3	8	11	8
ENSP00000366650	PYGM	754	11	10	13	10	21	9
ENSP00000366672	DTD1	209	1	2	2	1	2	0
ENSP00000367099	BFSP1	540	1	0	0	0	0	0
ENSP00000367220	ACTR2	399	3	6	5	6	8	3
ENSP00000367258	EEF1G	487	1	6	4	1	7	1
ENSP00000367323	KCNAB2	415	1	0	2	0	0	0
ENSP00000367558	USP9X	2554	4	4	2	3	4	5
ENSP00000367562	TMX2	258	2	2	0	2	0	0
ENSP00000367615	APRT	180	1	1	0	0	0	0
ENSP00000367872	GNB1	340	5	7	6	4	3	3
ENSP00000367873	CDKAL1	579	1	0	0	0	0	0
ENSP00000367923	SUCLA2	463	1	1	3	3	1	2
ENSP00000367992	ESD	282	1	2	1	1	2	1
ENSP00000368270	RAP1B	118	1	0	0	0	0	0
ENSP00000368352	CDS2	325	2	2	0	2	0	1
ENSP00000368403	PAX6	422	1	0	0	0	0	0
ENSP00000368566	ACSL6	722	3	3	5	0	5	0
ENSP00000368722	LDHA	332	7	6	6	6	7	5
ENSP00000368752	PRNP	253	1	1	0	0	1	0
ENSP00000369134	PDHA1	428	1	1	3	0	0	0
ENSP00000369315	MYH10	1992	1	0	0	0	0	0
ENSP00000369470	GDI2	148	1	1	0	3	2	1
ENSP00000369475	GDI2	449	7	5	7	14	8	8
ENSP00000369528	GDI2	400	7	6	7	15	8	7
ENSP00000369602	HBG2	137	20	22	20	20	25	24
ENSP00000369654	HBD	147	13	30	17	20	25	14
ENSP00000369703	TUBB2A	445	37.33	94.5	78.5	53	87.5	50.83
ENSP00000370023	HADHA	763	1	0	0	0	0	0
ENSP00000370223	IDH3B	385	2	0	3	1	1	4
ENSP00000370232	IDH3B	383	2	0	3	1	1	4
ENSP00000370388	DNM1L	738	2	9	4.5	2	9	7
ENSP00000370465	PFKP	776	1	6	6	0	7	8
ENSP00000370582	CD99	169	1	0	0	0	0	0

ENSP00000370724	FAM49A	323	1	1	0	0	0	0
ENSP00000370808	SLC25A6	298	2	3	2	1	0	0
ENSP00000371024	PDIA6	445	4	1	0	3	1	1
ENSP00000371084	ATP8A1	1164	4	2	0	2	0	3
ENSP00000371184	HPCAL1	193	1	3	0	2	0	0
ENSP00000371257	CIITA	546	1	1	0	1	0	0
ENSP00000371372	ATP12A	1039	1	0	0	0	0	0
ENSP00000371735	SACS	4579	1	0	0	0	0	0
ENSP00000371798	FSCN1	493	10	9	10	7	6	7
ENSP00000371889	WDR1	466	2	1	2	2	4	1
ENSP00000371890	WDR1	606	4	4	3	4	8	8
ENSP00000371894	AP2M1	433	6	8	9	9	13	10
ENSP00000372515	SEPT5	378	3	5	4	1	3	2
ENSP00000373200	CADM2	404	2	2	0	2	2	0
ENSP00000373620	HSPD1	573	1	1	1	2	5	2
ENSP00000373713	SACM1L	587	1	3	2	2	2	1
ENSP00000373860	ASPDH	283	1	0	2	2	0	0
ENSP00000373918	GARS	739	1	0	2	3	3	0
ENSP00000374292	CCDC57	916	1	0	0	0	0	0
ENSP00000374304	DOK7	608	2	0	0	1	0	0
ENSP00000374408	MROH2A	1674	2	4	2	0	0	0
ENSP00000375391	HSPA1B	641	13	14	9	12	15	7
ENSP00000375668	PPP2R1A	132	3	4	4	5	4	5
ENSP00000375816	ATP2B4	1134	3	2	0	3	1.5	1
ENSP00000375938	TUBA4A	433	25	23	21	14	16	19
ENSP00000376016	EZR	554	1	0	0	0	0	0
ENSP00000376066	ATP1A2	1009	8	11	3	6	3	7
ENSP00000376174	RAN	233	2	9	8	2	7.5	2
ENSP00000376763	CLTC	1639	172	224	184	186	198.5	214
ENSP00000376808	CUL5	780	1	0	2	0	3	1
ENSP00000376855	DPP10	800	1	0	1	0	1	0
ENSP00000376869	ATP2B1	963	8	10	3	4	0	1
ENSP00000376885	CPNE5	301	1	0	0	0	1	0
ENSP00000376892	NME1	152	1	0	0	0	1	0
ENSP00000376932	SYT1	422	5	6	10	9	10	7
ENSP00000377016	FLT4	1298	1	1	0	0	0	1
ENSP00000377195	DBN1	695	1	5	2	0	1	1
ENSP00000377275	GOLGB1	3269	1	0	0	0	0	0
ENSP00000377446	SUCLG1	346	2	3	2	1	2	3
ENSP00000377466	CNP	401	11	16	10	23	20	16
ENSP00000377470	CNP	421	21	23	14	34	29	24
ENSP00000377474	ACLY	1091	1	2	2	1	6	7
ENSP00000377833	ANXA4	321	1	1	0	2	1	0
ENSP00000377925	ACACA	42	1	0	0	0	0	0
ENSP00000377945	MDH1	334	5	3	3	5	2	1
ENSP00000377952	PSMD6	351	1	0	0	0	0	0
ENSP00000377958	CCT4	539	10	6.5	4	11	9	2
ENSP00000378174	SCUBE3	1009	2	6	2	0	1	0
ENSP00000378355	POR	633	2	2	1	0	0	0
ENSP00000378366	FLOT2	483	4	2	0	0	0	0
ENSP00000378479	SOS1	1318	1	0	0	0	0	1
ENSP00000378492	GRM2	872	1	1	0	0	1	0
ENSP00000378656	PFKM	749	0.5	0	1	0.5	2	0
ENSP00000378729	ALDOC	336	22	41	31	22.5	33	22
ENSP00000378872	ATP2A2	1015	6	14	5	7	10	9
ENSP00000378879	ATP2A1	994	5	3	4	5	6	3
ENSP00000379099	SLC1A2	565	13	7	5	7	1	1
ENSP00000379203	ATP6V1C1	382	1	0	1	2	0	3

ENSP00000379281	YWHAZ	245	3	3	1	4	4	1
ENSP00000379385	LDHB	232	2	0	1	2	0	4
ENSP00000379386	LDHB	334	2	0	0	2	1	3
ENSP00000379539	MYH10	1997	1	2	1	0	0	0
ENSP00000379602	VAMP1	118	4	5	2	3	2	4
ENSP00000379626	GNB5	283	1	3	3	2	3	1
ENSP00000379703	SEPT3	350	1	2	3	2	3	1
ENSP00000379769	ACO2	805	20	4	8	11	21	7
ENSP00000379845	ABAT	500	2	1	1	3	0	1
ENSP00000379933	TPI1	249	1	2	2	2	1	2
ENSP00000380067	GAPDH	293	4	14	13.5	5	15	6
ENSP00000380104	HBE1	87	44	50	41	49	55	43
ENSP00000380211	CPNE6	557	2	0	0	1	1	0
ENSP00000380267	ATP2B2	1198	13	22	22	10	18	13
ENSP00000380378	PAFAH1B1	410	5	5	5	3	1.5	3
ENSP00000380427	ARPC4-TTLL3	625	3	8	0	0	2	1
ENSP00000380646	TMEM80	216	1	0	0	0	0	0
ENSP00000380723	HRAS	189	1	2	1	0	2	2
ENSP00000380814	SLC12A6	1091	1	0	0	1	0	0
ENSP00000380899	HBA1	110	4	10	5	8	8	5
ENSP00000380987	CRMP1	572	18	31	25	18	51	21
ENSP00000381293	NSF	744	23	38	30	27	42	14
ENSP00000381412	CAMK2A	489	9	9	7	3	5	3
ENSP00000381456	CCT7	339	1	1	0	0	1	1
ENSP00000381459	NAPB	204	1	1	0	1	0	0
ENSP00000381504	PRPS2	321	1	1	0	2	0	0
ENSP00000381526	DPYSL3	570	5	2	1	4	7	4
ENSP00000381577	USP34	3546	1	0	0	0	0	0
ENSP00000381589	PCDHGA11	935	1	0	0	0	0	0
ENSP00000381803	MAPK1	360	2	3	1	2	2	1
ENSP00000381823	SEC24A	1093	0.5	0	0	1	0	0
ENSP00000382175	MYO5A	585	3	1	2	1	2	2
ENSP00000382179	MYO5A	1852	1	1	3	0	2	2
ENSP00000382380	MAP1A	2805	1	2	2	1	2	0
ENSP00000382412	HMGB1	158	1	3	2	0	1	0
ENSP00000382608	HSPA12B	600	2	0	0	1.5	0	0
ENSP00000382694	ATP6V1E1	196	2	4	10	5	6	3
ENSP00000382696	ATP6V1E1	204	9	22.5	13	8	13	5
ENSP00000382880	VAPA	249	1	0	1	0	0	0
ENSP00000382915	HSPA1A	641	8	11	7.5	7.5	7.5	3
ENSP00000382980	CSNK2B	215	1	0	1	2	1	2
ENSP00000382981	EPB41L3	865	23	18	15	21	26	14
ENSP00000382982	TUBA3C	450	241	465	334	247	393	315
ENSP00000383118	CDC42	191	3	5	1	8	6	2
ENSP00000383170	PHLPP1	1205	1	0	0	0	0	0
ENSP00000383191	COL18A1	1339	1	0	0	0	0	0
ENSP00000383366	EFCAB8	395	1	0	0	0	0	0
ENSP00000383392	NCAM2	837	1	0	0	0	1	0
ENSP00000383934	RAB37	216	2	2.5	0.33	0.5	2.66	0
ENSP00000384014	VSNL1	191	8	14	2	8	8	6
ENSP00000384038	PFKL	827	2.5	2	2	2.5	4	4
ENSP00000384144	HYOU1	999	5	6	2	3	2	10
ENSP00000384194	AP1B1	949	2	4	3	4	5	5
ENSP00000384311	NRXN1	1477	1	1	0	1	0	1
ENSP00000384313	MAST4	2434	1	0	0	0	0	0
ENSP00000384393	DPP6	353	3	0	0	0	0	0
ENSP00000384471	RTN4	986	3	6	0	3	0	0
ENSP00000384675	SOS1	1333	1	0	0	0	0	1

ENSP00000384930	PPM1F	286	1	0	0	1	0	0
ENSP00000385142	NRXN1	1547	1	0	0	0	0	1
ENSP00000385447	SYNGR1	169	9	12	14	10	10	3
ENSP00000385828	TST	297	1	0	0	0	0	0
ENSP00000385844	SACS	3829	1	0	0	0	0	0
ENSP00000385923	NUCB1	461	4	0	0	0	0	0
ENSP00000385958	RPS14	151	1	3	0	0	1	1
ENSP00000386190	C2orf16	1984	2	1	0	1	0	1
ENSP00000386192	DNM2	866	11.5	7	7	11	16	9
ENSP00000386249	SGOL2	247	1	0	1	0	1	0
ENSP00000386350	OLA1	416	3	4	1	4	2	1
ENSP00000386719	MDH1	210	1	1	1	1	0	1
ENSP00000386936	TPRKB	214	1	0	0	0	0	0
ENSP00000387028	PGAP1	592	1	0	0	1	0	0
ENSP00000387176	POTEJ	1038	6	12	5	6	6	9
ENSP00000387246	MAP3K2	619	1	1	1	0	0	0
ENSP00000387270	DCTN1	1139	2	1	1	0	5	4
ENSP00000387286	RAB1A	205	5	11	1	9	15.5	18.5
ENSP00000387506	IDH3A	257	12	9	6	11	7	6
ENSP00000387545	ANXA2	339	1	1	0	1	2	1
ENSP00000387641	CAND2	1236	1	0	0	0	0	1
ENSP00000387694	SLC12A5	1139	16	18	15	10	11	12
ENSP00000387725	SLC12A6	1091	5	1	1	4	1	0
ENSP00000387802	CADM3	234	1	0	1	0	0	0
ENSP00000388169	ARPC4	187	1	1	0	0	0	0
ENSP00000388347	GNAI1	41	1	0	1	1	1	0
ENSP00000388374	P4HB	464	2	0	0	3	1	0
ENSP00000388778	HSPH1	677	1	0	1	3	5	1
ENSP00000388804	CDH13	175	2	1	0	0	1	0
ENSP00000389007	NUCB1	267	1	0	0	0	0	0
ENSP00000389047	PRDX1	171	1	5	0	1	2	0
ENSP00000389372	CLASP1	1494	1	1	0	1	1	0
ENSP00000389435	GNAI1	144	2	3	6	7	0	3
ENSP00000390124	VSNL1	104	1	2	0	2	2	1
ENSP00000390129	VDAC1	184	1	0	3	1	1	1
ENSP00000390367	FDFT1	374	1	0	0	0	0	0
ENSP00000390555	PGAP1	159	1	0	1	0	0	0
ENSP00000390600	SCN10A	1956	3	4	2	2	1	2
ENSP00000390632	MOG	295	1	1	1	1	2	0
ENSP00000390944	QDPR	213	1	5	3	2	4	1
ENSP00000391075	IDH1	157	1	0	0	0	0	0
ENSP00000391243	DDR1	493	1	0	0	0	0	0
ENSP00000391319	RAB5B	174	1	6	3	1	4	3
ENSP00000391429	EHD1	306	2	3	3	2	5	1
ENSP00000391877	SPIB	171	1	0	0	0	0	0
ENSP00000391905	PPP2R1A	395	5	7	10	5	13	3
ENSP00000392028	CHD7	2997	1	0	0	0	0	0
ENSP00000392043	ATP2B1	1220	9	10	3	5	4	1
ENSP00000392208	CNTNAP2	185	1	1	1	0	0	0
ENSP00000392366	EEF1A1	106	4	9	2.5	2	4	2
ENSP00000392494	DDX3X	646	1	1	1	0	1	0
ENSP00000392686	EIF4A2	96	1	0	1	0	0	0
ENSP00000392765	GNB1	165	2	2	1	1	3	3
ENSP00000392878	OGDH	1019	4	3	0	4	2	1
ENSP00000393340	TUBB	372	20	29	9	20	22	18
ENSP00000393391	PLP1	100	6	4	4	8	6	4
ENSP00000393509	CAPZA2	172	1	2	2	2	2	1
ENSP00000393926	HSPA4L	870	1	3	4	1	8	3

ENSP00000394071	GDI1	447	5	9	11	8	12	7
ENSP00000394318	RAP1A	184	2	6	1	4	6	2
ENSP00000394558	YWHAB	74	6	2	4	7	7.5	0
ENSP00000394670	PPIA	120	4	16	2	4	9	9
ENSP00000394729	YWHAB	100	3	7	5	3	8	1
ENSP00000394817	CANX	627	5	1	4	6	4	3
ENSP00000395370	ARF3	144	1	3	0	1	0	2
ENSP00000395521	SLC2A1	249	1	0	0	1	0	0
ENSP00000395616	PRKCSH	525	1	1	1	0	0	0
ENSP00000395628	PAFAH1B1	205	2	1	2	1	0	1
ENSP00000395797	SLC4A10	1088	5	6	5	4	2	3
ENSP00000396061	TUBA4A	153	30	42	25	19	33	34
ENSP00000396127	RAN	234	5	17	16	7	14.5	7
ENSP00000396189	HSP90AA1	413	15	11	9	16	18	11
ENSP00000396268	ERG	387	1	0	0	0	0	0
ENSP00000396632	RAB5A	65	2	2	3	3	1	1
ENSP00000396684	BTN3A1	378	1	0	0	0	0	0
ENSP00000396722	NTM	355	6	4	3	5	4	4
ENSP00000397808	RTN4	203	1	2	0	0	0	0
ENSP00000398054	RAB2A	104	1	8	0	1	5	2
ENSP00000398239	ACTA2	151	17	47	37	21	30	31
ENSP00000398320	GLUL	373	11	8	6	11	10	5
ENSP00000398327	CDC42	136	1	6	1	0	3	5
ENSP00000398370	EIF4A2	408	1	3	1	0	2	3
ENSP00000398393	ACTN4	342	2	0	0	1	0	0
ENSP00000398481	CADPS2	1296	1	2	0	1	0	0
ENSP00000398599	YWHAZ	245	4	5	5	5	7	1
ENSP00000398932	ATP6V1E1	203	2	2	2	4	1	3
ENSP00000398973	CKMT1A	148	1	1	0	2	0	0
ENSP00000398976	ALDOC	149	0.5	1	1	2	1	1
ENSP00000398990	YWHAQ	149	3.5	10.5	6.5	4.5	3	3
ENSP00000399393	BTN3A1	43	1	0	0	0	0	0
ENSP00000399487	ACTB	99	3	4	6	8	6	3
ENSP00000399685	SEPT5	322	2	10	6	1	6	2
ENSP00000399741	GNB1	163	1	1	2	1	1	0
ENSP00000399904	GNB2	251	25	35.5	35	21	24	18
ENSP00000400022	AP1B1	578	12	16	15	17	19	12
ENSP00000400107	RAB14	181	4	17	5	4	15	4
ENSP00000400124	ATP1A1	106	7	3	0	5	1	2
ENSP00000400175	RHOA	193	3	6.5	0	7	2	1
ENSP00000400387	CAMK2B	168	3	3	4	2	1	0
ENSP00000400717	GNA13	377	6.5	3	2.5	6	3.5	1.5
ENSP00000400720	SNAP25	93	9	20	13	4	9	6
ENSP00000400923	MX1	197	1	0	0	0	1	0
ENSP00000401185	GBAS	80	1	1	2	0	1	0
ENSP00000401317	TUBB	444	9	16	12	8	15.5	14
ENSP00000401671	ITPR1	2743	6	5	3	5	1	4
ENSP00000401733	GDI2	263	3	1	2	2	0	2
ENSP00000401802	PSMC6	403	1	1	0	1	0	1
ENSP00000401873	GNB2	296	10	12	11	10	9	5
ENSP00000402033	PDZD2	2839	1	1	0	0	0	0
ENSP00000402051	AP2M1	136	2	0	1	1	0	1
ENSP00000402551	OTUB1	271	4	9	6.5	3.5	7	1
ENSP00000402836	PPIH	144	1	3	0	2	0	0
ENSP00000403175	RPS18	152	1	2	0	1	4	1
ENSP00000403335	PLP1	122	6	13	7	10	9	11
ENSP00000403349	ALDH2	470	2	2	3	1	3	0
ENSP00000403365	PKM	516	12	3	11	10	9	12

ENSP00000403497	PCDH1	1048	1	0	0	0	0	0
ENSP00000403636	SYNGAP1	1343	1	0	0	0	0	0
ENSP00000403778	RAC2	166	5	11	1	7	6	5
ENSP00000404092	SMC6	1091	1	0	1	0	0	0
ENSP00000404100	PAX6	436	1	0	0	0	0	0
ENSP00000404327	PCYOX1	129	2	3	3	3	1	1
ENSP00000404506	FSCN1	132	3	1	0	2	0	1
ENSP00000404740	TUBA4A	119	7	9	5	6	8	6
ENSP00000404801	MAST4	58	1	0	0	0	0	0
ENSP00000405114	ATP6V1B1	248	2	6	4.5	6	9	3
ENSP00000405395	CACNA2D1	309	2	0	0	1	0	0
ENSP00000405417	BCAP31	246	2	4	5	2	2	0
ENSP00000405452	PGK1	281	5	2	2	3	2	3
ENSP00000405729	CMTM1	169	1	0	0	0	0	0
ENSP00000405750	PLP1	277	11	28.5	18	11	13	8
ENSP00000405767	RAB11A	155	1	1	0	1	1	1
ENSP00000405788	TPO	864	1	0	0	0	0	0
ENSP00000405812	SLC4A8	1093	1	0	0	0	0	1
ENSP00000406028	ACTR1A	329	8	3	3	11	5	2
ENSP00000406091	DDR1	876	1	0	0	0	0	0
ENSP00000406131	CAMK2D	513	2	1	2	0	0	0
ENSP00000406133	SLC1A2	199	3	0	0	2	0	0
ENSP00000406288	CHD8	2302	1	0	0	0	0	0
ENSP00000406473	PDHA1	205	6	7	6	4	5	2
ENSP00000406511	NCDN	244	1	0	1	0	1	0
ENSP00000406547	SCAMP5	235	1	0	0	0	0	0
ENSP00000406747	YWHAH	162	1	5	5	4	4	2
ENSP00000407723	MAPRE2	315	2	2	2	6	1	0
ENSP00000407851	NIPSNAP1	221	1	1	3	0	1	0
ENSP00000408402	RHOA	129	1	6	0	2	1	1
ENSP00000408405	KCTD1	865	2	0	1	1	2	0
ENSP00000408644	SMC6	740	1	0	1	0	0	0
ENSP00000408649	MDH2	296	11	11.5	8	12	8	5
ENSP00000408695	PRKCA	672	2	3	4	4	4	5
ENSP00000408838	CALU	315	1	0	0	1	0	0
ENSP00000408858	PFKP	240	1	4	3	0	5	3
ENSP00000409081	AP2M1	433	1	2	2	3	4	1
ENSP00000409221	NTM	36	2	1	1	1	1	0
ENSP00000409374	CACNA2D1	280	1	1	0	0	1	0
ENSP00000409420	GPRASP1	1395	1	0	0	0	0	0
ENSP00000409462	TNK2	143	1	0	0	0	0	0
ENSP00000409493	OBSCN	7968	2	0	0	1	0	0
ENSP00000409667	SYNJ1	1612	4	9	3	5	12	9
ENSP00000409899	LY6H	140	2	5	0	3	2	0
ENSP00000410071	TUBB	444	3	5	3	2	3	2
ENSP00000410073	MDH1	222	2	3	2	4	2	3
ENSP00000410411	KMT2C	1524	1	0	0	0	0	0
ENSP00000410626	PHRF1	1648	1	0	1	1	0	0
ENSP00000410680	PHB	124	1	0	0	0	0	0
ENSP00000410829	TUBB	444	2	10	8	6	9	5
ENSP00000411505	GIGYF2	872	1	2	1	0	0	1
ENSP00000411599	PRNP	249	1	1	0	0	0	0
ENSP00000411612	ARPC2	165	1	0	1	0	0	0
ENSP00000411705	ATP1A2	714	17.5	33	19.5	14	28	23.5
ENSP00000411885	PLEKHH3	616	1	0	0	0	0	0
ENSP00000411916	ABAT	500	8	5	9	14	6	6
ENSP00000411941	RAB5A	201	1	5	5	1	5	2
ENSP00000412284	ARPC4-TTLL3	181	3	10	0	1	4	3

ENSP00000412439	FKBPL	349	2	0	0	0	1	0
ENSP00000412490	POR	418	2	1	1	0	0	0
ENSP00000412492	SNAP91	907	4	1	2	4	0	0
ENSP00000412701	ARF5	150	1	1	0	1	1	0
ENSP00000412883	SACM1L	187	1	3	1	1	1	1
ENSP00000413104	CNP	144	1	1	1	0	1	0
ENSP00000413122	BCL6	650	2	3	2	8	1	1
ENSP00000413135	APOE	216	4	0	0	2	0	1
ENSP00000413221	ARPC3	99	1	4	0	1	3	3
ENSP00000413227	RALA	164	3	4	3	4	5	2
ENSP00000413234	AP2A2	939	1	4	5	2	10	0
ENSP00000413255	CKMT1B	417	3	1	0.5	0	1	1
ENSP00000413294	NEGR1	308	5	2	3	0	2	1
ENSP00000413308	CCT3	276	2	3	1	1	3	1
ENSP00000413507	SEPT7	384	1	1	1	0	0	1
ENSP00000413649	SYNJ1	695	12	13.5	0	10	16	13
ENSP00000414150	ERG	486	1	0	1	0	0	0
ENSP00000414399	AKR1B1	263	1	1	2	1	1	0
ENSP00000414479	TUBB	372	31	37	26	17	23	18
ENSP00000414488	SEPT5	304	1	0	0	0	0	0
ENSP00000414532	FLNB	2422	1	2	0	0	0	0
ENSP00000415031	NFASC	1165	3	0	1	4	2	0
ENSP00000415067	PPP2R1A	534	7	4	1	10	2	0
ENSP00000415131	DNM1L	710	1	0	1	0	1	0
ENSP00000415284	PRNP	217	1	1	0	0	1	0
ENSP00000415370	P2RX5	432	1	0	0	0	0	0
ENSP00000415373	APMAP	289	3	0	1	0	0	0
ENSP00000415446	PRPSAP2	176	1	0	0	0	0	0
ENSP00000416028	PSMD2	778	1	0	2	2	3	2
ENSP00000416293	SLC2A1	492	2	0	0	3	0	0
ENSP00000416320	NTM	346	1	0	0	1	0	1
ENSP00000416330	TGFBI	683	1	1	2	0	0	0
ENSP00000416578	NEB	8525	1	0	0	0	0	0
ENSP00000416638	RPL7A	191	2	3	4	3	0	1
ENSP00000416651	GNB1	107	2	3	2	0	5	4
ENSP00000416745	RPS3	103	1	1	2	1	0	0
ENSP00000416929	MDH2	231	5	1	1	1	1	0
ENSP00000417000	RER1	158	1	2	1	0	0	0
ENSP00000417134	TMEM236	351	1	0	0	1	0	0
ENSP00000417155	RAB7A	134	3	5	1	4	4	5
ENSP00000417189	RAB7A	98	1	2	0	0	2	2
ENSP00000417240	RPSAP58	295	3	5	6	6	2.5	3
ENSP00000417267	PDHB	341	1	0	0	0	0	0
ENSP00000417284	RP11-553A10.1	106	1	0	0	0	0	0
ENSP00000417545	ATP6V1A	184	3	3	4	4	7	11
ENSP00000417606	PARP12	173	1	0	0	0	0	0
ENSP00000417668	RAB7A	160	2	3	0	3	3	0
ENSP00000417827	CADPS	240	1	0	0	3	1	2
ENSP00000417978	RAB7A	150	9	11	4	12	10	9
ENSP00000418235	ARHGEF7	151	1	0	0	0	0	0
ENSP00000418317	PDCD10	212	1	2	0.5	0	0	0
ENSP00000418506	LSAMP	205	1	0	0	1	2	0
ENSP00000418571	NCEH1	408	1	2	1	0	0	1
ENSP00000418700	PLS1	629	3	1	0	2	1	1
ENSP00000418799	TUBAL3	406	7	27	19	8	16	12
ENSP00000418809	CAMKV	470	1	1	0	0	1	0
ENSP00000418955	RAB7A	116	4	12.5	2	0	9.5	5
ENSP00000419000	LSAMP	338	1	0	2	0	1	2

ENSP00000419225	DNM1	851	35	56	60	48.5	82	61
ENSP00000419227	PSMD6	350	1	0	0	1	0	0
ENSP00000419580	PDHB	251	1	1	0	0	0	0
ENSP00000419608	AHCYL2	182	2	0	1	1	1	0
ENSP00000419693	GNB4	340	6	7	4	3	3	3
ENSP00000419764	IPO5	1099	1	0	0	0	1	4
ENSP00000419879	CDS2	445	2	2	0	2	0	1
ENSP00000419904	GPM6B	302	1	1	0	0	1	0
ENSP00000419921	GPM6B	184	5	7	8	2	5	4
ENSP00000420044	CADPS	178	2	2.5	1	2	2	2
ENSP00000420045	DNM1	864	2	6	4	2	3	6
ENSP00000420213	FLNB	2633	1	1	0	0	0	0
ENSP00000420424	PDCD10	146	1	1	0.5	0	0	0
ENSP00000420839	SEPT11	372	3	4	10	3	4	3
ENSP00000420908	SPOP	374	1	6	6	3	6	11
ENSP00000420931	LPHN3	1580	1	0	0	0	0	0
ENSP00000421067	ANK2	1863	2	0	0	2	3	0
ENSP00000421396	PPP2R2B	449	2	1	0	3	2	2
ENSP00000421421	ANXA5	163	1	0	0	0	0	0
ENSP00000421578	GPM6A	267	19	25	17.5	14.5	16	7
ENSP00000421813	CANX	107	3	0	1	3	0	0
ENSP00000422473	TMEM33	247	1	1	1	1	1	0
ENSP00000422542	UCHL1	223	1	4	5	1	4	3
ENSP00000422781	PPP3CA	289	2	1	1	2	1	0
ENSP00000422853	ANK2	966	5	0	1	4	7	1
ENSP00000422896	SEPT11	439	1	0	2	0	0	0
ENSP00000422959	GPM6A	218	1	8	2	2	0	1
ENSP00000422990	PPP3CA	423	2	5	9	3	4	0
ENSP00000423098	HSPA9	62	1	1	1	2	2	0
ENSP00000423224	APC	1135	1	0	0	0	0	0
ENSP00000423264	CKMT2	170	2	2	2	1	1	0
ENSP00000423318	CCT5	520	2	2.5	0	0	2	1
ENSP00000423337	WFS1	890	1	2	2	0	2	1
ENSP00000423416	MAP1B	569	2	1	3	0	2	1
ENSP00000423444	MAP1B	670	7	4	6	9	10	10
ENSP00000423449	SH3D19	767	1	0	0	0	0	0
ENSP00000423497	ENPP6	194	2	1	1	4	1	1
ENSP00000423935	TGFBI	65	1	0	0	0	0	0
ENSP00000423984	GPM6A	271	3	1	2	4	2	0
ENSP00000424063	CANX	592	60	34	39	48	38	28
ENSP00000424103	WFS1	252	2	0	1	1	1	0
ENSP00000424106	ANXA5	260	7	3	3	4	0.5	1
ENSP00000424199	ANXA5	220	1	0	0	0	0	0
ENSP00000424387	TARS	723	1	0	0	0	0	0
ENSP00000424790	NFKB1	968	1	0	0	0	0	0
ENSP00000424987	WDFY3	639	1	0	0	1	0	0
ENSP00000425262	SEPT11	126	1	0	2	1	0	0
ENSP00000425299	EIF2S1	315	1	0	0	0	2	0
ENSP00000425598	HSPA9	95	1	0	0	0	1	0
ENSP00000426052	CANX	78	3	3	2	4	2	2
ENSP00000426096	RAP1GDS1	147	1	0	1	0	0	0
ENSP00000426528	AMER2	671	1	1	1	1	0	1
ENSP00000426589	TGFBI	59	1	0	1	0	0	0
ENSP00000426607	CAMK2A	90	5	5	6	4	5	0
ENSP00000426634	UCHL1	58	1	0	0	0	0	1
ENSP00000426915	SMG7	1145	1	0	0	0	0	0
ENSP00000427099	C5orf28	113	1	3	0	2	1	11
ENSP00000427135	ANXA5	35	1	0	0	0	0	0

ENSP00000427203	SLC1A3	101	3	1	2	1	0	1
ENSP00000427305	HSPA4L	531	1	0	0	1	3	0
ENSP00000427835	ATP6V1H	101	1	1	1	0	0	1
ENSP00000427862	UQCRB	161	1	0	0	0	0	0
ENSP00000427954	DPYSL2	170	5	9	2	5	7	6
ENSP00000427985	DPYSL2	536	79	94	78	87	125	95
ENSP00000428009	CYFIP2	1057	6	22	6	12	18	17.5
ENSP00000428209	UBE2V2	145	1	1	0	1	2	1
ENSP00000428255	ATP6V1H	48	2	1	1	0	0	1
ENSP00000428333	PCDHGA11	750	1	0	0	0	0	0
ENSP00000428381	YWHAZ	75	1	0	0	1	0	0
ENSP00000428450	TACC1	610	1	0	1	0	0	0
ENSP00000428511	SNAP91	877	1	0	0	0	0	0
ENSP00000428775	YWHAZ	125	6	7	5	13	6	4
ENSP00000428845	VDAC3	284	1	0	0	0	0	0
ENSP00000428916	ANXA6	156	1	0	0	2	1	0
ENSP00000429041	YWHAZ	137	2	1	1	2	2	0
ENSP00000429301	PGRMC2	247	1	2	0	0	1	0
ENSP00000429419	UBE2V2	105	1	2	0	1	2	1
ENSP00000429466	NCALD	152	2	4	0	3	0	0
ENSP00000429701	DNM3	639	18	17	16	24	21	20
ENSP00000429947	ATP6V1H	443	6	6	11	11	7	6
ENSP00000429997	SPIDR	209	1	0	0	0	0	0
ENSP00000430055	ENO3	283	1	0	0	4	0	1
ENSP00000430219	YWHAZ	51	5	3	4	3	1	4
ENSP00000430282	ATP6V1C1	382	1	1	2	2	0	4
ENSP00000430636	ENO3	391	15	25	21	30	33	21
ENSP00000430682	ATP6V1B2	199	9	6	9	10	10	10
ENSP00000430758	PPP2CB	124	1	1	2	1	2	1
ENSP00000430817	PPIA	105	2	3	0	2	0	0
ENSP00000430848	ZFHX4	3590	1	0	0	0	0	0
ENSP00000430869	PPP2CA	126	1	0	0	0	0	1
ENSP00000430870	PHYHIP	248	2	2	2	2	2	2
ENSP00000431007	TMEM30A	325	1	2	1	2	1	0
ENSP00000431087	ENO3	434	3	1.5	3	4	3.5	3.5
ENSP00000431117	DPYSL2	536	8	14	14	15	21	14
ENSP00000431146	PPP1CA	253	1	0	0	0	0	0
ENSP00000431267	MBP	32	4	2	0	4	1	0
ENSP00000431364	RAP1GAP	602	1	2	1	0	1	0
ENSP00000431543	CCT3	500	2	3	2	0	4	1
ENSP00000431545	PICALM	148	1	1	0	0	0	0
ENSP00000431589	RAB2A	181	1	0	0	1	1	0
ENSP00000431630	AP2A2	656	12	16	11	10	22	16
ENSP00000431721	RPS18	82	1	3	0	1	3	1
ENSP00000431737	RPS3	135	1	0	1	0	0	0
ENSP00000432083	HSPA8	646	3	2	6	5	7	1
ENSP00000432112	RDX	604	2	0	0	0	1	0
ENSP00000432155	CFL1	149	1	0	0	1	1	0
ENSP00000432249	CD81	209	1	0	0	1	0	0
ENSP00000432542	RPS3	91	1	4	2	0	0	1
ENSP00000432568	SPTBN2	2390	1	0	1	0	1	5
ENSP00000432793	TEKT2	96	1	0	0	0	0	0
ENSP00000432884	HSPA8	312	8	4	8	10	13	6
ENSP00000432988	MBP	98	1	2	2	3	0	0
ENSP00000433034	RPS2	218	1	1	0	0	0	0
ENSP00000433155	RPS3	117	1	0	0	0	0	1
ENSP00000433287	NPEPPS	915	2	0	0	2	3	2
ENSP00000433308	CFL1	79	1	3.5	0	0	0	2

ENSP00000433397	HYOU1	937	2	3	0	1	0	2
ENSP00000433464	RPL8	257	1	2	0	0	0	2
ENSP00000433821	RPS3	243	1	1	1	0	1	1
ENSP00000433910	CFL1	149	5	24	2	8	15	14
ENSP00000434272	ATP1A4	517	2.5	1.5	2.5	0.5	3	2.5
ENSP00000434286	EIF3F	100	1	1	0	0	1	0
ENSP00000434415	HSPA8	171	4	5	3	8	5	3
ENSP00000434466	INTS4	963	1	0	0	0	0	0
ENSP00000434565	HSPA8	137	2	3	1	2	1	3
ENSP00000434570	SOGA3	947	1	0	0	2	0	2
ENSP00000434586	TTN	26926	2	4	2	2	3	2
ENSP00000434599	PPP3CA	132	1	1	0	0	1	0
ENSP00000434744	DNAJC5	167	13	18	10	11	11	6
ENSP00000434851	HSPA8	132	2	2	2	2	4	3
ENSP00000435019	HSPA8	150	2	1	1	2	4	1
ENSP00000435154	HSPA8	187	6	5	0	6	6	6
ENSP00000435195	RAB1B	169	2	4.5	0	1	2.5	4
ENSP00000435306	GANAB	852	9	3	2	15	4	1
ENSP00000435376	CUL5	780	1	0	2	0	3	1
ENSP00000435406	SLC1A2	91	1	2	1	0	0	0
ENSP00000435777	RPS13	151	2	7	0	1	2	1
ENSP00000435786	CMTM1	238	1	0	0	0	0	0
ENSP00000435908	HSPA8	223	1	1	1	1	1	1
ENSP00000436029	SLC1A2	199	10	15	10	9	7	6
ENSP00000436208	STIM1	540	1	0	0	0	0	0
ENSP00000436369	RP11-192H23.4	171	1	0	0	0	0	0
ENSP00000436431	CFL1	159	1	2	1	1	0	0
ENSP00000436603	IQGAP3	1588	8	7	4	5	6	3
ENSP00000436654	DPYS	54	2	0	1	3	0	2
ENSP00000436978	FAM203B	103	1	0	0	0	0	0
ENSP00000437215	RPL35	96	1	1	1	1	2	1
ENSP00000437228	AP2A2	125	1	0	0	0	0	0
ENSP00000437577	ATP1A3	1024	2	2	0	1	1	2
ENSP00000437595	M6PR	148	1	0	0	0	1	0
ENSP00000437611	PITPNM2	1349	1	0	0	0	0	0
ENSP00000437822	PGD	470	1	1	3	3	2	0
ENSP00000437824	CCT7	499	2	1	1	3	0	0
ENSP00000437971	RTN3	185	4.5	4	4	3.5	6	6
ENSP00000438144	MDH1	352	2	1	2	1	0	1
ENSP00000438260	PDIA3	485	19	16	17	16	12	16
ENSP00000438507	RP11-302B13.5	210	2	4	0	2	4	3
ENSP00000438550	PDHA1	397	3	5	6	0	1	1
ENSP00000438687	PIK3C2A	1306	2	2	1	1	1	0
ENSP00000438715	RDH12	316	1	0	1	0	0	0
ENSP00000438778	PDIA6	437	1	0	0	1	0	0
ENSP00000438873	ENO2	434	1	1	1	2	3	0
ENSP00000438964	KPNB1	731	1	0	0	1	0	0
ENSP00000438966	SEMA7A	652	1	1	2	1	0	1
ENSP00000439170	TBCE	578	1	0	0	0	0	0
ENSP00000439189	POTEE	1075	11	15	13	11	10	11
ENSP00000439218	ACOT7	265	2	4	2	3	2	3
ENSP00000439245	PCYT2	389	1	0	1	1	0	0
ENSP00000439312	WNT5B	359	1	1	0	0	0	0
ENSP00000439418	PHEX	652	1	0	0	0	0	0
ENSP00000439528	CADPS	212	1	0	0	0	0	0
ENSP00000439568	PPP1CA	169	1	0	0	1	1	1
ENSP00000439870	BFSP1	526	1	0	0	0	0	0
ENSP00000439874	ATP6V1A	584	10	8	9	11	16	8

ENSP00000439966	RAP1B	184	1	0	0	0	0	1
ENSP00000440001	SLC3A2	247	2	0	0	0	0	1
ENSP00000440045	ATP2A2	1042	3	2	0	0	1	0
ENSP00000440211	OTUB1	286	1	1	1.5	2.5	1	0
ENSP00000440504	KIAA1958	744	1	0	0	0	0	0
ENSP00000440523	ATIC	533	2	0	2	2	0	2
ENSP00000440543	CS	453	1	1	1	1	0	0
ENSP00000440690	VPS45	434	1	0	0	0	1	0
ENSP00000440817	BCAT1	385	1	1	0	1	0	0
ENSP00000440842	MAPK1	316	2	7	3	3	4	3
ENSP00000440874	RTN3	236	17	23	16	14	14	10
ENSP00000441084	INTS4	338	1	0	0	0	0	0
ENSP00000441282	ATP6V0D1	392	2	1	1	2	1	0
ENSP00000441328	OTUB1	241	4	8	6	4	7	1
ENSP00000441767	SLC17A7	548	1	1	1	0	0	0
ENSP00000441770	CLASP1	1255	1	0	0	1	0	0
ENSP00000441819	DPYSL3	196	1	2	2	3	6	2
ENSP00000441847	CCT2	530	6	5	8	6	7	4
ENSP00000441931	ANXA4	237	1	1	0	2	1	0
ENSP00000442091	EPB41L3	865	2	4	2	1	0	1
ENSP00000442174	CCT4	483	1	1	2	2	5	0
ENSP00000442318	CAND1	1230	13	17	1	20	24	26
ENSP00000442348	WNT5B	253	1	0	0	0	0	0
ENSP00000442559	PDE1B	495	1	0	0	0	0	0
ENSP00000442665	HADHB	452	1	0	0	0	1	0
ENSP00000442680	LDHB	102	7	5	4	10	2	3
ENSP00000442730	CCT8	475	2	0	0	1	1	2
ENSP00000442789	HSPA1B	550	2	2	3	3	9	1
ENSP00000442820	AHCY	404	2	6	5	4	2	1
ENSP00000442821	PGRMC1	143	1	1	0	1	0	2
ENSP00000442849	NAPG	230	4	2	3	1	0	0
ENSP00000442864	ANXA7	336	1	0	0	1	0	0
ENSP00000443049	POTEE	394	1	0	0	1	0	0
ENSP00000443061	CCT4	509	10	6	3	9	6	2
ENSP00000443362	LDHA	89	5	0	0	3	0	1
ENSP00000443429	LSAMP	358	3	1	1	4	2	1
ENSP00000443449	PGM1	365	3	3	5	3	5	2
ENSP00000443493	PRKCG	710	3	3	6	2	4	5
ENSP00000443515	SLC8A1	937	1	1	0	0	0	1
ENSP00000443682	XPNPEP3	288	1	0	0	0	1	0
ENSP00000443683	ST8SIA5	345	1	0	0	0	0	0
ENSP00000443787	PYGL	813	1	2	1	2	2	1
ENSP00000443798	RYR2	4951	1	6	2	1	3	3
ENSP00000443994	RAB35	185	5	8	5.5	9.5	8	5.5
ENSP00000444003	MTHFSD	382	1	0	0	0	0	0
ENSP00000444042	RAN	198	1	5	5	3	5	2
ENSP00000444074	PFKFB1	406	1	0	0	0	0	0
ENSP00000444089	CAND1	770	2	2	0	3	4	5
ENSP00000444236	SLC3A2	599	1	0	0	1	1	0
ENSP00000444292	LDHA	72	1	0	0	0	0	0
ENSP00000444379	CCT7	443	5	6	5	5	6	6
ENSP00000444501	SEPT7	235	1	1	2	1	2	1
ENSP00000444596	MYCBP2	4640	1	0	0	0	0	0
ENSP00000444676	ATP6V0A1	837	2	3	6	3	1	3
ENSP00000444708	PGK1	389	13	7	6	13	15	5
ENSP00000444830	RAP1B	142	1	0	0	1	1	1
ENSP00000445257	ACTR3	356	2	1	3	1	3	1
ENSP00000445306	ATP1A1	1023	1	0	0	0	0	0

ENSP00000445330	CORO1B	282	1	2	0	0	1	0
ENSP00000445471	CCT2	488	3	0	2	1	2	2
ENSP00000445578	GOT1	392	2	0	0	2	1	0
ENSP00000446091	SLC12A5	186	1	0	1	1	0	0
ENSP00000446130	PCBP2	301	5	8	8	5	6	2
ENSP00000446489	ATP5B	362	23	19	17	9	22	11
ENSP00000446677	ATP5B	284	3	0	1	1	1	3
ENSP00000446748	HSP90B1	48	6	1	2	4	4	4
ENSP00000447006	CNTN1	1018	15	11	2	9	5	10
ENSP00000447040	KRT8	289	2	8	7	3	1	1
ENSP00000447068	PCBP2	202	1	1	1	1	1	0
ENSP00000447096	ATP2B1	162	1	0	0	0	1	0
ENSP00000447488	LIN7A	233	1	0	0	0	0	0
ENSP00000447571	ATP5B	270	4	1	0	0	2	0
ENSP00000447845	DNM1L	172	1	0	0	0	0	0
ENSP00000447860	CNTN1	90	9	3	0	11	3	2
ENSP00000448004	CNTN1	627	10	6	0	6	6	5
ENSP00000448104	NTM	191	2	2	1	2	0	2
ENSP00000448253	PFKM	139	1	0	1	0	1	1
ENSP00000448428	ATP5B	133	3	3	3	2	2	2
ENSP00000448437	PPP1CC	270	2	1	1	0	1	0
ENSP00000448648	TUBA1B	49	6	11	4	6	11	3
ENSP00000448653	CNTN1	627	17	16	3	15	17	11
ENSP00000448725	TUBA1A	112	12	14	8	8	11	8
ENSP00000448767	KRT5	201	1	11	8	1	5	3
ENSP00000449016	TUBA1A	26	4	13	6	6	9	12
ENSP00000449018	ERP29	160	2	1	1	2	1	0
ENSP00000449080	CS	123	1	3	1	2	2	2
ENSP00000449269	PFKM	156	2	6	4	2	7	5
ENSP00000449325	TUBA1B	246	26	23	19	29	28	17
ENSP00000449415	SYT1	185	18	28	22	9	23	20
ENSP00000449426	PFKM	780	8	11	12	17	27	14
ENSP00000449460	TUBA1B	81	3	7	5	2	7	3
ENSP00000449517	CNTN1	57	3	4	1	3	3	2
ENSP00000449748	TUBA1B	75	8	21	11	9	19	12
ENSP00000449762	RAB5B	79	1	1	1	1	1	1
ENSP00000449777	RAB5C	91	3	2	1	1	3	0
ENSP00000450238	OSBPL8	721	2	1	1	0	0	0
ENSP00000450268	TUBA1A	219	9	28	24	12	15	17
ENSP00000450297	ATP5B	518	1	2	1	0	1	0
ENSP00000450353	RP11-162P23.2	261	1	0	1	0	0	0
ENSP00000450407	ATP2A2	46	4	2	3	5	1	4
ENSP00000450412	CNTN1	62	3	0	0	2	1	1
ENSP00000450538	TUBB3	189	4	10	11	3	6	9
ENSP00000450674	DYNC1H1	180	5	4	1	6	3	3
ENSP00000450683	NRXN3	1392	3	2	1	2	2	2
ENSP00000450712	HSP90AA1	174	11	10	14.5	9	20	9
ENSP00000450822	TUBB3	118	1	5	7	2	4	5
ENSP00000450992	SV2B	683	3	2	1	0	0	0
ENSP00000451069	CYP46A1	96	1	1	1	1	0	0
ENSP00000451156	PSMC6	262	1	1	0	0	0	1
ENSP00000451276	TUBB3	55	2	4.5	2.5	4	3.5	2.5
ENSP00000451378	TUBB3	148	7	13	10	6	13	8
ENSP00000451426	CKB	179	1	1	3	3	5	4
ENSP00000451560	TUBB3	797	6	11	8.5	4	11.5	8.5
ENSP00000451564	ATP6V1D	153	2	5	5	3	4	2
ENSP00000451611	CKB	128	5	1	1	8	2	0
ENSP00000451617	TUBB3	378	43	101.5	86.5	49.5	59.5	43

ENSP00000451622	LRP1	139	1	1	2	1	0	1
ENSP00000451648	NRXN3	1061	1	0	0	0	0	1
ENSP00000451686	CKB	90	5	4	1	3	3	3
ENSP00000451754	NRXN3	175	1	1	0	1	0	1
ENSP00000451870	TRAJ56	21	1	2	4	7	12	13
ENSP00000452001	TUBB3	51	5	8	5	3	8	5
ENSP00000452166	TUBB3	46	9	18	10	9	11	18
ENSP00000452192	ATP6V1D	179	1	1	1	1	2	1
ENSP00000452506	ATL1	99	1	0	0	0	0	0
ENSP00000452754	IDH3A	109	2	2	1	1	2	1
ENSP00000452921	ANXA2	176	4	5	4	4	4	0
ENSP00000453084	IDH3A	331	4	4	2	3	3	3
ENSP00000453111	RAB8B	48	1	2	0.5	2	0	0.5
ENSP00000453222	IDH3A	134	1	1	0	0	0	1
ENSP00000453233	CYFIP1	151	1	1	0	1	1	0
ENSP00000453352	CPNE6	226	1	0	0	0	0	0
ENSP00000453480	IDH3A	46	1	1	1	1	1	1
ENSP00000453544	C15orf57	203	1	0	0	0	0	0
ENSP00000453566	ANXA2	65	1	0	1	0	1	0
ENSP00000453663	ANXA2	149	1	1	0	2	1	0
ENSP00000453747	IDH3A	175	6	0	1	3	0	0
ENSP00000453925	ANXA2	79	3	5	4	3	4	4
ENSP00000454365	SEMA7A	80	1	0	1	0	0	1
ENSP00000454435	CDH13	713	2	2	3	1	2	2
ENSP00000454453	CDIPT	213	1	3	1	1	0	0
ENSP00000454499	ALDOA	140	1	4	4	3.5	3	1
ENSP00000454611	CNOT1	439	2	0	0	0	0	0
ENSP00000454728	GNAO1	128	37.5	58	42	25	41	25
ENSP00000454755	GDE1	102	1	0	0	0	0	0
ENSP00000454861	CTC-554D6.1	94	1	0	0	0	0	0
ENSP00000454963	ABAT	515	2	1	2	5	1	0
ENSP00000454971	GNAO1	94	1	0	0	0	0	1
ENSP00000455313	SCAMP5	243	3	2	0	0	2	0
ENSP00000455447	TRAPPC2L	59	1	0	0	0	0	0
ENSP00000455455	ALDOA	278	1	6	3	2	3	3
ENSP00000455502	ABAT	117	1	2	1	0	2	2
ENSP00000455507	OBSCN	8923	3	1	2	2	2	0
ENSP00000455671	NPTN	152	2	5	6	0	1	1
ENSP00000455724	ALDOA	364	5	12	10	11	16	18
ENSP00000455749	APRT	148	1	1	0	0	0	0
ENSP00000455774	GNAO1	83	9	9	9	6	9	7
ENSP00000455899	STX1B	277	33	60	42	25	36	27
ENSP00000455901	PKM	531	46	57	48	51	86	64
ENSP00000456033	NPTN	308	1	0	0	3	1	0
ENSP00000456098	ALDOA	130	4	2	3	2	3	7
ENSP00000456162	ATXN2L	401	1	0	0	1	0	0
ENSP00000456180	IQSEC3	1182	1	0	0	0	0	0
ENSP00000456259	SMG1	3551	1	0	0	0	0	0
ENSP00000456270	RPL7A	191	2	1	0	1	0	1
ENSP00000456295	GDE1	129	1	0	0	0	0	0
ENSP00000456349	NPTN	278	1	2	0	2	0	0
ENSP00000456638	RAB11A	198	1	4	2	2	3	2
ENSP00000456695	ATP6V0D1	115	1	0	0	0	1	0
ENSP00000456984	PKM	151	8	4	3	6	10	5
ENSP00000457028	NPTN	394	3	4	3	2	2	0
ENSP00000457067	STX1B	204	1	4	7	5	5.5	6
ENSP00000457238	GNAO1	110	5	5	8	6	8	5
ENSP00000457253	PKM	485	8	5	5	7	9	16

ENSP00000457420	PKM	82	6	13	11	8	10	8
ENSP00000457514	ALDOA	204	0.5	3	2	0	3	2
ENSP00000457540	RAB5C	91	4	3	3	1	5	2
ENSP00000457610	TUBB8	410	7	8	4	8	11	5
ENSP00000457766	CDIPT	157	1	5	0	2	2	0
ENSP00000457794	MTHFSD	89	1	0	0	0	0	0
ENSP00000457980	PRKCB	130	2	1	2	1	3	4
ENSP00000458035	ATP2A1	113	3	2	0	2	2	0
ENSP00000458085	FHOD1	157	1	0	0	0	0	0
ENSP00000458149	CAMP	170	1	0	0	0	0	0
ENSP00000458162	ACTG1	375	1	5	1	0	3	1
ENSP00000458221	NSF	739	7	14	9	9	17	3
ENSP00000458238	PI4KA	2044	1	0	0	0	0	0
ENSP00000458310	NLRP7	1065	1	0	0	0	0	0
ENSP00000458474	HSPA12A	675	2	2	3	8	7	1
ENSP00000459093	HYOU1	653	3	3	2	2	2	8
ENSP00000459119	ACTG1	214	120	187	158	123	160	110
ENSP00000459145	ATP1B2	166	1	2	1	0	1	0
ENSP00000459354	TRAP1	215	1	1.5	2	2	1	1
ENSP00000459386	GNAI2	339	15	24	14	13	15	10
ENSP00000459444	STX1A	251	6	6	2	2	3	2
ENSP00000459531	STX1A	288	3	2	1	1	1	2
ENSP00000459646	NSF	136	8	14	8	11	12	10
ENSP00000459836	PCYT2	286	1	0	1	1	0	0
ENSP00000459838	PPP2R1B	667	2	4	2	2	5	2
ENSP00000459841	LPHN3	1299	1	0	0	0	0	0
ENSP00000459911	MDH2	338	4	2	4	6	2	0
ENSP00000459932	NCAM1	858	1	1	1	1	1	1
ENSP00000460126	CRYM	241	1	0	0	1	1	2
ENSP00000460130	HGS	110	1	0	0	0	0	0
ENSP00000460464	ACTG1	214	16	13	10	16	12	4
ENSP00000460499	ATP1B2	172	4	4	4	4	4	1
ENSP00000460598	PKMYT1	215	1	1	0	0	5	0
ENSP00000460712	YWHAE	107	1	1	0	1	0	3
ENSP00000460810	MDH2	296	2	0	1	1	1	2
ENSP00000460932	ATP2B3	1154	25	42	25	15	26	23
ENSP00000460989	DDX5	614	2	2	3	3	4	0
ENSP00000461407	ACTG1	241	10	16	10	11	7	7
ENSP00000461619	MBOAT7	123	1	0	0	0	0	0
ENSP00000461762	YWHAE	233	5	7	7	12	5	5
ENSP00000461857	KIF5C	957	2	1	0	3	4	2
ENSP00000461904	CRYM	140	1	0	0	0	0	0
ENSP00000462223	MBP	304	25	37	14	28	28	18
ENSP00000462421	RAC3	130	3	5	0	3	6	6
ENSP00000462548	RIOK3	516	2	1	0	1	0	0
ENSP00000462550	TRAPPC8	926	1	0	0	0	0	0
ENSP00000462571	EIF4A2	109	1	0	1	0	0	1
ENSP00000462597	AQP4	301	2	0	0	1	0	0
ENSP00000462780	MBP	60	1	4	0	3	4	4
ENSP00000462783	EPB41L3	147	5	1	1	3	1	0
ENSP00000462904	EIF4A1	257	4	4	2	2	6	4
ENSP00000462938	RPL19	194	2	1	1	1	2	1
ENSP00000463016	SV2A	742	15	9	11	15	12	9
ENSP00000463018	NAPG	167	1	0	0	0	0	0
ENSP00000463066	KPNB1	690	1	3	3	0	7	3
ENSP00000463118	ALDOC	122	1	2	3	4	4	2
ENSP00000463328	ATP6V0D1	232	17	14	9	16	10	2
ENSP00000463393	SEC22B	215	2	4	4	0	3	0

ENSP00000463503	FLOT2	428	4	2	0	1	0	0
ENSP00000463916	VPS45	570	1	0	0	0	0	0
ENSP00000463985	RPL19	193	1	1	0	1	1	0
ENSP00000464042	KPNB1	148	1	0	1	3	1	1
ENSP00000464083	AC242842.1	164	1	6	0	0	0	0
ENSP00000464093	SCRN2	180	1	0	0	0	0	0
ENSP00000464261	KCTD1	260	2	0	0	1	1	0
ENSP00000464496	NAPG	18	1	0	0	1	0	0
ENSP00000465076	ROGDI	217	1	2	1	1	0	1
ENSP00000465105	CALR	247	2	0	0	2	0	2
ENSP00000465113	NCAM1	160	1	0	0	0	0	0
ENSP00000465154	NCAM1	364	11	11	7	11	8	10
ENSP00000465200	CLTC	222	3	2	2	5	3	2
ENSP00000465477	ATP5A1	503	4	11	14	2.5	5	6
ENSP00000465478	ACACA	235	1	0	0	0	0	0
ENSP00000465489	PRKCSH	525	1	0	1	0	0	0
ENSP00000465623	ALDOC	135	7	10	6	3	5	3
ENSP00000465819	FLOT2	249	2	0	0	1	0	0
ENSP00000465820	VAMP3	72	3	8	3	7	6	8
ENSP00000465869	NCAM1	135	1	1	1	1	1	0
ENSP00000466290	ATP6V0A1	93	5	1	0	1	0	1
ENSP00000466295	NCAM1	357	2	3	2	2	2	4
ENSP00000466324	YWHAE	115	1	0	2	1	1	0
ENSP00000466328	ATP6V0A1	130	4	4	2	4	1	5
ENSP00000466406	ENO1	195	13	14	14	13	14	16
ENSP00000466696	NCAM1	733	22	25	13	14	18	18
ENSP00000467037	ATP5A1	531	1	3	4	0	2	1
ENSP00000467172	TBCB	169	1	0	0	0	0	0
ENSP00000467255	GAPDHS	179	2	2	7	1	5	3
ENSP00000467270	ENO3	28	1	0	1	1	1	1
ENSP00000467373	ATP6V0A1	198	9	3	1	6	4	1
ENSP00000467399	VAMP2	113	16	31	12	15	26	27
ENSP00000467451	NCAM1	133	1	1	0	0	1	0
ENSP00000467501	RAB8A	207	1	1	0	0	1	0
ENSP00000467515	NCAM1	725	2	2	2	1	2	4
ENSP00000468198	CNP	164	1	0	0	1	1	0
ENSP00000468417	DIRAS1	198	1	0	0	0	0	0
ENSP00000468420	HSPA12A	136	1	3	2	1	4	2
ENSP00000468571	TUBB6	116	7	11	5	6	5	3
ENSP00000468777	TUBB6	338	1	10	9	4	8	6
ENSP00000468854	ATP2B3	1220	2	2	1	1	0	3
ENSP00000468904	MAPRE2	127	1	0	0	0	0	0
ENSP00000468923	AC040977.1	174	1	0	0	0	0	0
ENSP00000468946	CLTCL1	1640	11	6	6	7	5	5
ENSP00000468957	RPS16	50	1	3	0	1	1	1
ENSP00000469129	ATP1A3	178	47	62	48	49.5	43	45
ENSP00000469550	L1CAM	1248	1	0	0	0	2	0
ENSP00000469660	TUBB4A	107	2	5	6	2	7	3
ENSP00000469966	TPI1	167	2	0	1	1	1	1
ENSP00000470087	AC006486.9	167	1	1	0	0	2	0
ENSP00000470102	ACTG1	375	38	99	86	46	87	74
ENSP00000470233	CAPN12	179	1	0	0	1	0	0
ENSP00000470256	DKFZP434E1119	150	1	0	0	0	0	0
ENSP00000470338	SLC17A7	493	8	3	3	5	4	3
ENSP00000470388	USP5	835	2	3.5	5	4	7	4
ENSP00000470571	SYP	313	18	14	11	13	10	7
ENSP00000470627	TUBB4A	157	6	6	9	9	3	5
ENSP00000470642	PGAM4	254	4	7	8	4	8	7

ENSP00000470907	ENO2	103	3	2	0	1	4	3
ENSP00000470986	PGK1	389	33	54	45	36	51	40
ENSP00000471186	GDI1	447	3	1	1	2	1	1
ENSP00000471425	IRGC	275	2	5	3	2	4	2
ENSP00000471490	ENO2	434	4	9	6	12	12	5
ENSP00000471503	TUBB4A	103	1	5	3	1	4	2
ENSP00000471510	CAPN12	93	1	0	0	0	0	0
ENSP00000471581	ATP1A3	983	192.5	293.5	203	154.5	222	184.5
ENSP00000471921	SPIB	262	1	0	0	0	0	0
ENSP00000472079	USP5	858	1	1	1	1	3	1
ENSP00000472219	ZNF446	399	1	0	0	1	0	0
ENSP00000472335	RAB3A	115	7	15.5	7	12	13.5	11
ENSP00000472375	TUBB4A	167	4	2	1	3	0	0
ENSP00000472379	GNB3	232	4	5	5	5	2	2
ENSP00000472481	TUBB4A	155	18	24	22	11	24	24
ENSP00000472697	TECR	153	1	0	0	0	1	0
ENSP00000472957	TECR	150	1	0	0	0	0	0
ENSP00000472985	RPS5	204	1	4	1	0	5	3
ENSP00000473036	PNMAL2	635	1	0	0	0	0	0
ENSP00000473053	ACTG1	198	7	6	11	8	8	7
ENSP00000473186	SYP	313	4	0	1	3	0	2
ENSP00000473334	ACTR1A	332	5	1	1	3	2	1
ENSP00000473474	SDR42E2	388	0.5	0	1	0	0	0
ENSP00000473821	DNAJC6	516	1	1	0	0	0	0
ENSP00000474569	UBA1	1058	9	17	3	6	15	11
ENSP00000474757	UBA1	1058	7	5	1	4	10	5
ENSP00000475018	AP2B1	913	16	9	7	20	15	11
ENSP00000475260	TPI1	113	2	2	2	1	3	0
ENSP00000475548	ENO2	73	1	1	1	4	2	2
ENSP00000476010	PGRMC2	91	1	1	1	1	1	1
ENSP00000476032	TPI1	167	7	14	11	10	15	8
ENSP00000476090	BASP1	45	7	6	6	3	5	0
ENSP00000476193	ESD	120	1	0	0	0	0	0
ENSP00000476311	PFKP	118	1	0	0	0	2	1
ENSP00000476532	ARHGEF2	83	1	0	0	0	0	0
ENSP00000476641	ATP1A3	1226	2	2	6	1	2	5
ENSP00000476705	ATP1A3	1013	86.5	101	66	71.5	82.5	54.5
ENSP00000476707	GDI2	130	2	1	0	5	2	1
ENSP00000476711	SLC25A4	208	1	1	0	0	0	0
ENSP00000476744	SPIDR	209	1	0	0	0	0	0
ENSP00000477015	ATP1B1	130	15	7	8	11	6	6
ENSP00000477141	FRRS1L	344	2	4	4	1	2	0
ENSP00000477245	EPRS	968	1	0	0	1	0	2
ENSP00000477306	TUBA8	275	16	22	25	23	22	22
ENSP00000001008	FKBP4	459	0	2	2	0	0	0
ENSP00000007722	ITGA3	1066	0	1	0	0	0	0
ENSP00000009589	RPS20	119	0	5	1	0	2	2
ENSP00000012443	PPP5C	499	0	1	0	0	0	0
ENSP00000016946	RGPD5	1765	0	1	0	0	0	0
ENSP00000084795	RPL18	190	0	3	0	1	0	1
ENSP00000156084	OTUD5	571	0	1	0	0	0	0
ENSP00000168216	HSD17B10	261	0	1	1	0	1	0
ENSP00000173527	ISOC1	298	0	1	0	0	0	0
ENSP00000179882	STXBP5	806	0	1	0	1	0	0
ENSP00000188376	SLC25A3	361	0	3	0	2	1	1
ENSP00000202773	RPL6	288	0	1	1	0	1	0
ENSP00000211402	VARS	1264	0	2	1	0	0	1
ENSP00000212015	SIRT1	747	0	1	0	0	0	0

ENSP00000215754	MIF	115	0	1	0	0	0	0
ENSP00000216378	CDKL1	276	0	1	0	0	0	0
ENSP00000217515	TXNL1	289	0	1	0	0	1	0
ENSP00000217901	IDH3G	393	0	1	1	0	0	0
ENSP00000218364	HTATSF1	755	0	1	0	1	0	0
ENSP00000219150	CORO1A	461	0	2	1	1	1	0
ENSP00000219789	CDIPT	213	0	3	0	0	0	0
ENSP00000221403	DHDH	334	0	1	0	0	0	0
ENSP00000221740	CASP14	242	0	1	0	0	0	0
ENSP00000221742	SLC1A6	564	0	1	1	0	0	1
ENSP00000221975	RPS19	71	0	2	0	1	0	0
ENSP00000222247	RPL18A	176	0	2	0	0	1	0
ENSP00000225174	PPIF	207	0	3	0	0	0	0
ENSP00000225655	PFN1	140	0	3	3	0	1	4
ENSP00000225719	CPD	1380	0	1	0	0	0	1
ENSP00000226004	DUSP3	185	0	3	0	0	0	0
ENSP00000228682	GLI1	1106	0	1	0	0	1	0
ENSP00000228929	HIST2H2AC	129	0	2	2	1	2	1
ENSP00000229329	CMAS	434	0	1	0	0	0	0
ENSP00000230085	SNX3	162	0	2	0	0	1	0
ENSP00000231487	SKP1	163	0	1	0	0	0	0
ENSP00000232461	GNAT1	350	0	1	1	1	0	0
ENSP00000232496	TUSC2	110	0	1	0	0	0	0
ENSP00000233121	MAPRE3	281	0	12	1	2	3	3
ENSP00000233468	SF3B14	125	0	1	0	0	0	1
ENSP00000234038	PPP1R7	360	0	1	0	1	1	0
ENSP00000234396	ATP6V1B1	513	0	5	8	0	12	5
ENSP00000234677	SARS	514	0	1	1	0	0	0
ENSP00000236342	DHDDS	333	0	1	0	0	0	0
ENSP00000236957	EEF1B2	225	0	1	1	0	1	0
ENSP00000238018	GDA	471	0	1	0	0	1	0
ENSP00000238875	LGALSL	172	0	1	0	1	1	0
ENSP00000239940	PFN2	140	0	1	1	0	1	0
ENSP00000240139	PPP3CC	512	0	1	1	1	1	0
ENSP00000240185	TARDBP	414	0	1	2	0	0	0
ENSP00000242059	SCRN1	414	0	1	0	0	0	0
ENSP00000242577	DYNLL1	89	0	1	2	1	2	1
ENSP00000243298	RAB9B	201	0	0.5	0	1	0.5	0
ENSP00000243903	ACTR5	607	0	1	1	0	0	0
ENSP00000244040	RAB22A	194	0	1	0	0	1	0
ENSP00000244766	NRN1	142	0	1	0	0	0	1
ENSP00000245304	RAP2A	183	0	3	1	1	0	1
ENSP00000246489	KLC1	637	0	1	0	1	0	0
ENSP00000246548	UBA2	640	0	1	0	0	0	0
ENSP00000246635	KRT13	458	0	1	1	0	0	0
ENSP00000246792	RRAS	218	0	1	1	0	0	2
ENSP00000247665	PHPT1	125	0	1	0	0	1	0
ENSP00000247706	ABHD8	439	0	1	0	0	0	0
ENSP00000247930	ZNF777	831	0	1	0	0	1	1
ENSP00000248996	GNAZ	355	0	2	1	1	1	1
ENSP00000249270	DNAJC2	568	0	2	1	0	1	1
ENSP00000249396	SIRT2	389	0	1	0	0	0	0
ENSP00000250498	DAD1	113	0	1	1	0	1	2
ENSP00000250784	RPS4Y1	263	0	3	3	0	0	0
ENSP00000252027	SBF1	1868	0	1	0	0	0	1
ENSP00000252250	KRT6C	564	0	5	6	2	0	0
ENSP00000252252	KRT6B	564	0	11	5	2	1	0
ENSP00000252512	XPO7	1087	0	1	0	0	0	1

ENSP00000252593	BST2	180	0	1	0	0	0	0
ENSP00000252599	COLGALT1	622	0	1	0	0	0	0
ENSP00000253003	ADRM1	407	0	1	0	0	0	0
ENSP00000253023	UBE2M	183	0	7	0	0	0	0
ENSP00000253054	GMFG	109	0	1	0	1	0	0
ENSP00000253408	GFAP	432	0	1	0.5	0	0	0
ENSP00000253788	RPL27	136	0	2	0	0	3	1
ENSP00000253794	VPS25	176	0	3	0	0	1	1
ENSP00000254442	WDR7	1490	0	1	1	1	3	2
ENSP00000254901	REEP2	252	0	1	0	0	0	0
ENSP00000256010	NTS	170	0	1	0	0	0	0
ENSP00000256078	KRAS	189	0	1	0	1	0	2
ENSP00000256496	ARL8B	186	0	10.5	4	2	8	7
ENSP00000256797	ERN2	974	0	1	0	1	0	0
ENSP00000256876	IL2RA	263	0	1	0	0	0	0
ENSP00000256935	DOCK2	1830	0	1	0	0	1	0
ENSP00000258405	SERPINE2	398	0	1	0	0	0	0
ENSP00000258455	MRPS9	396	0	1	0	0	0	0
ENSP00000258739	KDELR2	212	0	1	0	0	0	0
ENSP00000258962	SRSF1	248	0	1	1	0	0	0
ENSP00000259455	GABBR2	941	0	1	2	1	0	0
ENSP00000259477	ARPC5L	153	0	3	0	0	2	2
ENSP00000260638	RAB1A	129	0	2.5	0	3	2.5	5
ENSP00000260746	ARL3	182	0	4	0	0	1	0
ENSP00000261017	ABI2	475	0	1	0	0	0	0
ENSP00000261182	NAP1L1	391	0	1	1	0	0	1
ENSP00000261263	RAB21	225	0	2	1	1	1	1
ENSP00000261636	ARL1	181	0	2	0	0	0	0
ENSP00000261772	AARS	968	0	3	0	2	7	4
ENSP00000261797	NDST1	882	0	1	0	0	0	0
ENSP00000261839	MYO5C	1742	0	2	1	0	1	2
ENSP00000261937	FLT4	1363	0	1	0	0	0	0
ENSP00000262033	PTGES3	160	0	8	0	1	4	3
ENSP00000262043	PHF3	2039	0	1	0	0	0	0
ENSP00000262102	MTUS1	1270	0	1	0	0	0	0
ENSP00000262113	MYOM2	1465	0	1	0	0	0	0
ENSP00000262187	RHEB	184	0	1	0	0	2	0
ENSP00000262215	ARFGEF1	1849	0	1	0	0	0	0
ENSP00000262306	TCEB2	161	0	2	1	0	2	0
ENSP00000262419	KANSL1	1105	0	1	0	0	0	0
ENSP00000262631	SCN1B	218	0	1	0	0	0	0
ENSP00000262803	UPF1	1118	0	1	0	0	1	0
ENSP00000262811	MAST3	1309	0	1	0	0	0	0
ENSP00000262835	ACSL4	670	0	1	0	0	1	1
ENSP00000262942	ARPC1A	370	0	4	2	1	2	2
ENSP00000262948	MAP2K2	400	0	2	0	0	1	1
ENSP00000262982	CSE1L	971	0	3	1	1	1	1
ENSP00000263025	MAPK3	379	0	2	1	0	0	0
ENSP00000263050	AKAP7	84	0	1	0	0	0	0
ENSP00000263168	CAPZA1	286	0	3	0	1	0	0
ENSP00000263270	AP2S1	142	0	4	0	0	3	1
ENSP00000263441	DNAJC6	900	0	1	0	0	0	0
ENSP00000263655	CNRIP1	164	0	1	0	0	2	2
ENSP00000263672	SPCS2	226	0	1	0	0	0	0
ENSP00000263674	ARHGEF17	2063	0	1	0	0	0	0
ENSP00000263710	CLASP1	1538	0	1	0	0	1	0
ENSP00000263811	DYNC1I2	632	0	2	2	0	1	1
ENSP00000263817	ABCB11	1321	0	1	0	1	0	0

ENSP00000264051	NGEF	710	0	1	1	0	4	2
ENSP00000264052	SP100	879	0	2	0	0	0	0
ENSP00000264059	EFHD1	239	0	2	0	1	0	0
ENSP00000264157	CCNT2	730	0	1	0	0	0	0
ENSP00000264258	RPL31	125	0	4	0	0	3	2
ENSP00000264331	TOP2B	1626	0	1	0	0	0	0
ENSP00000264357	GRIA3	894	0	1	0	0	0	0
ENSP00000264426	GRIA2	883	0	1	0	3	0	0
ENSP00000264485	SLC4A4	1079	0	1	0	0	1	0
ENSP00000264651	KRT24	525	0	1	1	0	0	0
ENSP00000264674	MECOM	1116	0	1	1	0	0	0
ENSP00000264689	UFSP2	469	0	1	0	0	0	0
ENSP00000264758	ADD1	768	0	1	0	0	0	0
ENSP00000264904	USO1	890	0	1	0	0	0	0
ENSP00000264993	CDV3	258	0	1	0	0	0	0
ENSP00000265052	MGLL	313	0	1	1	0	0	0
ENSP00000265073	SUB1	127	0	1	0	0	0	0
ENSP00000265077	VCAN	3396	0	1	0	0	1	0
ENSP00000265100	RPL26L1	145	0	2	0	0	0	0
ENSP00000265107	WDR70	654	0	1	0	0	0	0
ENSP00000265312	FN1	2211	0	1	0	0	1	0
ENSP00000265335	RAD50	1312	0	1	0	0	0	0
ENSP00000265368	SYNE1	8797	0	1	1	0	0	0
ENSP00000265421	POLB	335	0	1	0	0	0	0
ENSP00000265459	NRXN2	1712	0	1	0	0	0	1
ENSP00000265563	PRKAR2A	404	0	2	1	0	3	0
ENSP00000265620	GNAS	379	0	1	0	0	1	0
ENSP00000265662	ABCA2	2436	0	2	2	0	3	2
ENSP00000265709	ANK1	1897	0	1	0	0	1	0
ENSP00000265729	SRI	198	0	1	0	0	0	0
ENSP00000265838	ACAT1	427	0	2	0	0	2	0
ENSP00000265990	BTAF1	1849	0	1	2	0	0	0
ENSP00000266126	EIF2B2	351	0	1	0	0	0	0
ENSP00000266742	NEDD1	660	0	1	0	0	0	0
ENSP00000268446	MYLK4	388	0	1	0	0	0	0
ENSP00000268712	NCOR1	2440	0	1	0	0	0	0
ENSP00000269080	ABCA8	1581	0	1	0	0	0	1
ENSP00000269095	MPP2	552	0	1	1	0	0	0
ENSP00000269214	ESCO1	840	0	1	0	0	0	0
ENSP00000269554	PIP4K2B	416	0	2	1	0	0	0
ENSP00000269886	SH3GL1	368	0	2	0	1	2	0
ENSP00000270625	RPS11	158	0	1	0	0	2	1
ENSP00000270708	WRAP73	460	0	1	2	0	0	1
ENSP00000271688	CERS2	380	0	1	0	0	0	0
ENSP00000272134	LEFTY1	366	0	1	0	0	0	0
ENSP00000272217	ARL8A	186	0	3.5	0	0	0	0
ENSP00000272298	CALM2	149	0	2	1	2	2	2
ENSP00000273130	DYNC1LI1	523	0	1	1	0	0	0
ENSP00000273368	TAGLN3	199	0	4	0	1	1	0
ENSP00000274643	MYLK4	388	0	1	0	0	0	0
ENSP00000275730	SLC12A9	914	0	0.5	0	0	0	0
ENSP00000276079	NONO	471	0	1	0	0	0	1
ENSP00000277225	ZNF462	2506	0	1	0	0	0	0
ENSP00000279599	ABI1	496	0	1.5	0	0	1	0
ENSP00000280346	DLAT	647	0	1	1	0	0	0
ENSP00000281243	QDPR	244	0	5	1	1	6	2
ENSP00000282141	CRYGC	174	0	0.5	0	0	0	0
ENSP00000282541	GPD1L	351	0	1	1	0	1	1

ENSP00000282588	ITGA1	1179	0	1	0	0	0	0
ENSP00000283141	SYCP2L	812	0	1	0	0	0	0
ENSP00000283195	RANBP2	3224	0	1	0	0	0	0
ENSP00000283254	SCN3A	2000	0	2	1	0	0	0
ENSP00000283256	SCN2A	2005	0	1	0	0	0	1
ENSP00000283628	UBP1	540	0	2	0	0	2	0
ENSP00000284202	IMPACT	320	0	2	1	0	1	3
ENSP00000284240	THY1	161	0	1	0	0	0	0
ENSP00000284811	TCEB1	112	0	1	0	0	2	3
ENSP00000284981	APP	770	0	2	1	0	1	0
ENSP00000285039	MYO5B	1848	0	1	0	0	0	0
ENSP00000285393	ATP6V0D2	350	0	0.5	0	0	0	0
ENSP00000285735	RHOC	193	0	1	0	0	0	0
ENSP00000286428	VBP1	197	0	1	1	0	0	0
ENSP00000286835	SCAF4	1147	0	1	1	0	0	1
ENSP00000287038	RPL30	115	0	2	2	1	2	0
ENSP00000287380	TBC1D31	1066	0	1	0	0	0	0
ENSP00000287613	ARPC4	78	0	4	0	1	2	2
ENSP00000288221	ERC2	957	0	1	0	0	0	0
ENSP00000289166	FAM46B	425	0	1	0	0	0	0
ENSP00000289316	HIST1H2BD	126	0	1	0	0	2	0
ENSP00000290209	SLC12A6	1099	0	1	0	1	0	0
ENSP00000290299	ATP5O	213	0	1	0	0	0	0
ENSP00000290349	CBR1	277	0	2	1	0	1	0
ENSP00000290573	HK2	917	0	1	0	0	3	0
ENSP00000290921	CTBP1	440	0	1	0	0	0	2
ENSP00000291503	ATP13A1	1086	0	1	0	0	0	0
ENSP00000291700	S100B	92	0	3	2	3	6	5
ENSP00000292304	LMNA	634	0	1	0	0	0	0
ENSP00000293371	DCD	110	0	1	2	0	0	0
ENSP00000294117	GNG3	75	0	1	1	0	0	0
ENSP00000294618	DOCK6	2047	0	1	0	0	0	0
ENSP00000294725	KCNT2	1135	0	1	0	0	0	0
ENSP00000295237	XIRP2	3549	0	1	0	1	0	1
ENSP00000295238	CCNT2	663	0	1	0	0	0	0
ENSP00000295448	GNPDA2	276	0	1	0	0	0	0
ENSP00000295640	RNPEP	650	0	1	2	0	4	0
ENSP00000295822	EIF5A2	153	0	1	0	1	0	1
ENSP00000295897	ALB	609	0	1	0	1	0	0
ENSP00000295937	DNAH12	3092	0	1	1	0	1	0
ENSP00000296255	RPN1	607	0	2	3	0	1	0
ENSP00000296289	TKT	576	0	1	1	0	3	1
ENSP00000296417	H2AFZ	128	0	1	0	0	0	0
ENSP00000296674	RPS23	143	0	2	0	0	0	0
ENSP00000296679	WDR41	459	0	1	0	0	0	0
ENSP00000296742	CAGE1	641	0	1	0	0	0	0
ENSP00000296820	TERT	807	0	1	0	0	0	0
ENSP00000297518	CDK5	260	0	1	1	0	0	0
ENSP00000298097	FBXO33	555	0	1	0	0	0	0
ENSP00000298283	RPL10L	214	0	1	1	0	0	0
ENSP00000298316	ARF6	175	0	4	0	0	2	0
ENSP00000298684	NDRG2	328	0	5	2	0	4	1
ENSP00000298743	GAS1	345	0	1	0	0	0	0
ENSP00000298767	WAPAL	1190	0	1	0	0	0	0
ENSP00000298852	PSMC3	439	0	1	0	0	0	0
ENSP00000299714	SEC11C	99	0	1	0	0	0	0
ENSP00000299736	CENPV	272	0	1	1	0	0	0
ENSP00000299798	SLC9A5	896	0	1	0	0	0	0

ENSP00000300177	GREM1	184	0	2	0	0	0	0
ENSP00000300413	SNRPD1	119	0	1	1	0	0	0
ENSP00000300527	COL6A2	1019	0	1	0	0	0	0
ENSP00000300797	PRRT2	299	0	1	0	0	0	0
ENSP00000301072	TUBA1C	449	0	5	0	0	2	1
ENSP00000301180	DIP2B	1576	0	1	0	0	0	0
ENSP00000301653	KRT16	473	0	4	3	0	0	0
ENSP00000301891	SLC22A11	550	0	1	3	1	0	0
ENSP00000302194	ATP6V1G2	118	0	2	0	0	1	2
ENSP00000302896	RPS9	194	0	3	0	1	1	0
ENSP00000303476	TLN2	2542	0	1	0	0	0	0
ENSP00000304006	PAFAH1B2	175	0	1	1	2	1	0
ENSP00000304133	SCG2	617	0	1	0	0	0	0
ENSP00000304500	ATP9B	1136	0	1	0	0	1	2
ENSP00000304604	MAGI3	1481	0	4	3	1	4	1
ENSP00000304704	CLP1	361	0	1	0	0	0	0
ENSP00000304748	SCN9A	1989	0	1	1	0	0	0
ENSP00000305260	GNB2	340	0	5	5	0	3	2
ENSP00000305556	PCBP1	356	0	2	1	0	3	0
ENSP00000305860	HNRNPD	336	0	1	1	0	0	0
ENSP00000305941	USH2A	5202	0	0.5	0	0	0	0
ENSP00000306010	ARF4	180	0	1	0	0	0	0
ENSP00000306106	JAGN1	183	0	3	0	0	0	0
ENSP00000306670	NUDT16L1	211	0	4	0	1	0	1
ENSP00000306918	PCDHGC4	938	0	1	0	0	0	0
ENSP00000307188	ASL	464	0	1	0	0	0	0
ENSP00000307208	BPTF	2920	0	1	0	0	1	0
ENSP00000307240	KRT74	529	0	1.5	0	2	0	0
ENSP00000307567	QARS	775	0	1	0	0	0	0
ENSP00000307634	AGAP1	857	0	1	0	0	0	0
ENSP00000307674	CANT1	401	0	1	0	0	0	0
ENSP00000307786	CYCS	105	0	1	0	1	0	1
ENSP00000307889	RPL13	211	0	4	1	0	1	0
ENSP00000307939	GCC2	1684	0	1	0	0	1	1
ENSP00000308021	CEP290	2481	0	1	0	0	0	0
ENSP00000308318	PPP1R3B	285	0	1	0	0	0	0
ENSP00000308532	DBN1	649	0	1	0	0	0	0
ENSP00000308685	ADCY5	911	0	1	0	0	0	0
ENSP00000308773	PPIP5K1	1406	0	1	0	0	0	0
ENSP00000308887	WNT5B	359	0	1	0	0	0	0
ENSP00000308908	GPR148	347	0	1	0	0	0	0
ENSP00000308927	SOX5	750	0	1	1	0	0	0
ENSP00000308957	MCTP1	778	0	1	0	0	0	0
ENSP00000309334	RPL15	204	0	2	0	0	0	0
ENSP00000309591	PRKACA	351	0	3	4	3	3	2
ENSP00000309845	HRAS	189	0	2	1	0	2	0
ENSP00000310572	PSMC5	406	0	1	0	0	1	0
ENSP00000310668	NUP93	819	0	1	0	0	0	0
ENSP00000310749	HPCAL1	193	0	2	0	2	0	0
ENSP00000310935	FKBP2	142	0	1	1	0	0	0
ENSP00000311115	ECHDC1	284	0	2	1	0	0	0
ENSP00000311293	RAPH1	644	0	1	0	0	0	0
ENSP00000311449	RAB6A	208	0	5	2	0	3	0
ENSP00000311469	GSTM1	218	0	2	1	3	1	1
ENSP00000311930	ETFB	255	0	1	1	0	0	0
ENSP00000312042	HDLBP	1268	0	1	0	0	0	0
ENSP00000312206	KRT3	629	0	2	0.5	1	1	0
ENSP00000312529	COL6A2	828	0	1	0	0	0	0

ENSP00000313829	KHDRBS1	443	0	2	0	0	0	0
ENSP00000313933	GLTSCR1L	1079	0	1	1	0	1	0
ENSP00000314214	VAMP2	116	0	9	3	1	7	10
ENSP00000314348	DDX10	875	0	1	0	0	0	0
ENSP00000314776	MBLAC2	279	0	1	0	0	0	0
ENSP00000314850	PPM1A	324	0	1	0	0	0	0
ENSP00000315454	BPTF	3046	0	1	0	0	1	0
ENSP00000316696	GRIN1	922	0	1	0	0	0	0
ENSP00000317300	LPCAT4	524	0	1	1	2	0	0
ENSP00000317302	C21orf2	255	0	1	0	0	0	0
ENSP00000317468	CHMP6	201	0	1	1	0	1	0
ENSP00000317636	RECQL5	991	0	1	0	0	0	0
ENSP00000317891	TNIP1	636	0	1	0	0	0	0
ENSP00000318643	EPSTI1	307	0	1	0	0	0	0
ENSP00000318646	RPS15A	130	0	1	0	0	2	1
ENSP00000318803	SYTL2	935	0	1	0	0	0	1
ENSP00000319096	RAP2B	183	0	4	1	2	1	2
ENSP00000319678	ST5	1137	0	1	0	0	0	0
ENSP00000319690	HNRNPC	306	0	1	2	0	2	3
ENSP00000320622	MYLK	1845	0	1	0	0	0	0
ENSP00000320777	ASCC3	176	0	1	0	0	0	0
ENSP00000321071	SEPT4	459	0	1	1	0	1	0
ENSP00000321203	TNIP2	429	0	1	0	0	0	0
ENSP00000321537	LLGL1	1064	0	1	0	0	0	0
ENSP00000321826	STXBP5	1151	0	1	0	0	0	0
ENSP00000322175	AK4	223	0	1	0	0	0	0
ENSP00000322316	COQ7	217	0	1	0	0	0	0
ENSP00000322439	TUFM	455	0	1	0	0	0	0
ENSP00000322938	SCRIB	1630	0	1	0	0	0	0
ENSP00000323687	UBE2O	1292	0	1	2	1	0	1
ENSP00000325127	ERGIC1	159	0	1	0	0	1	0
ENSP00000325269	NRCAM	1183	0	1	0	1	1	0
ENSP00000325369	AP3S1	193	0	2	0	0	1	2
ENSP00000325506	CLVS1	354	0	1	0	1	0	0
ENSP00000325905	SRSF7	238	0	1	0	0	0	0
ENSP00000326247	SAMD9L	1584	0	1	1	0	0	0
ENSP00000326411	CWF19L1	538	0	1	0	0	0	0
ENSP00000326708	OVCH1	1134	0	1	0	0	0	0
ENSP00000327054	LMNB2	620	0	1	0	0	0	0
ENSP00000327209	PDE4DIP	2240	0	3	0	1	2	1
ENSP00000327583	RANBP1	201	0	3	0	0	0	1
ENSP00000328203	TRAIP	469	0	1	0	1	0	0
ENSP00000328690	DDX24	859	0	1	0	0	0	0
ENSP00000329376	CNOT10	717	0	1	0	0	0	0
ENSP00000329471	KDELR1	212	0	1	0	0	0	0
ENSP00000329757	ATP6V0C	155	0	1	0	0	0	0
ENSP00000329867	PPME1	386	0	1	0	1	0	0
ENSP00000330633	CNTN2	1040	0	1	0	1	1	2
ENSP00000330945	TMED9	235	0	2	2	0	0	0
ENSP00000331363	C19orf68	627	0	1	1	0	0	0
ENSP00000331485	RGS7	451	0	2	0	0	1	0
ENSP00000331563	ARL17A	125	0	1	0	0	0	2
ENSP00000331748	RAB12	244	0	1	0	0	0	0
ENSP00000331787	METTL7A	244	0	2	1	0	0	0
ENSP00000331897	IDH2	452	0	5	4	1	3	2
ENSP00000331902	COL4A5	1691	0	1	0	0	0	0
ENSP00000332706	PURA	322	0	1	3	0	3	2
ENSP00000332931	SLC8A1	973	0	1	0	0	0	1

ENSP00000333551	PROSC	275	0	1	1	1	1	0
ENSP00000334448	GNG2	71	0	3	5	2	2	3
ENSP00000334523	KLC1	639	0	1	0	1	0	0
ENSP00000334779	PPIP5K1	1408	0	1	0	0	0	0
ENSP00000334785	TESC	214	0	1	0	0	0	0
ENSP00000334798	KRT26	468	0	2	3	0	0	0
ENSP00000334808	USP16	823	0	1	0	0	0	0
ENSP00000334983	PKM	531	0	3	3	0	6	0
ENSP00000335341	CDC42BPA	1781	0	1	0	0	0	0
ENSP00000335371	TRAPPC11	1133	0	1	0	0	0	0
ENSP00000335397	RTN4RL2	420	0	1	0	0	0	0
ENSP00000336721	LARP1	1019	0	3	0	1	0	4
ENSP00000336725	DDX3Y	660	0	1	1	0	1	0
ENSP00000336741	DHX15	795	0	1	0	0	0	0
ENSP00000337226	CDCA4	241	0	1	0	0	0	0
ENSP00000337838	RTN4	1192	0	1	0	1	0	0
ENSP00000338107	CAGE1	824	0	1	0	0	0	0
ENSP00000338200	FN1	2355	0	1	0	0	1	0
ENSP00000338650	ZC4H2	201	0	1	0	0	0	0
ENSP00000338777	AP3S2	193	0	1	0	0	1	0
ENSP00000339027	RPLP0	317	0	3	0	0	1	0
ENSP00000339063	EEF1A1	462	0	4	1	2	4	5
ENSP00000339095	RPS7	194	0	3	0	0	0	3
ENSP00000339467	RHOG	191	0	2	0	0	2	0
ENSP00000339485	WARS	430	0	1	0	0	1	0
ENSP00000339720	NDUFA4	81	0	1	0	0	0	1
ENSP00000339795	RPL7	248	0	2	0	0	0	0
ENSP00000339850	DPYSL4	572	0	2	4	1	4	0
ENSP00000340138	DLEU2L	55	0	1	0	0	0	0
ENSP00000340305	UBE2V1	170	0	3	0	1	2	0
ENSP00000340944	PTPN11	593	0	1	0	0	0	0
ENSP00000340970	TRMT2B	459	0	1	0	0	0	0
ENSP00000341170	PTN	168	0	1	0	0	0	0
ENSP00000341781	SYNE2	6885	0	1	1	0	0	0
ENSP00000341805	PHF10	498	0	2	0	0	0	0
ENSP00000342434	BAZ1B	1483	0	1	0	0	0	0
ENSP00000342564	ALDH6A1	522	0	1	2	0	0	1
ENSP00000342714	H2AFV	90	0	1	0	0	0	0
ENSP00000342787	RPL28	137	0	2	0	0	1	0
ENSP00000343002	B3GALTL	498	0	0.5	0	0	0	0
ENSP00000343314	FXYD1	92	0	2	1	0	0	0
ENSP00000343317	PPP2R1B	556	0	1	0	0	1	0
ENSP00000343535	USP7	1102	0	1	0	1	0	0
ENSP00000344259	UBE2L3	154	0	1	0	0	0	0
ENSP00000344672	NHSL1	1606	0	1	0	0	0	0
ENSP00000344762	SRSF3	124	0	2	0	0	0	0
ENSP00000344822	S100A13	98	0	2	2	0	0	1
ENSP00000345079	CSRP1	193	0	2	0	2	1	0
ENSP00000345149	AKAP7	81	0	1	0	0	0	0
ENSP00000345361	ARPC5L	153	0	3	0	0	2	2
ENSP00000345689	RAB5C	216	0	4	2	0	3	4
ENSP00000346022	RPL9	192	0	2	0.5	0	0	0
ENSP00000346027	RPL21	160	0	1	0	1	1	0
ENSP00000346037	RPLP1	114	0	1	0	0	1	0
ENSP00000346045	RPS17	135	0	3	0	0	0	0
ENSP00000346088	RPL22	128	0	2	0	0	2	0
ENSP00000346111	NSD1	2427	0	2	0	1	0	0
ENSP00000346173	ETFB	346	0	1	1	0	0	0

ENSP00000346465	RTN4	960	0	2	0	0	0	0
ENSP00000347232	BLM	1417	0	1	1	0	0	0
ENSP00000347271	RPS10	165	0	1	0	0	0	0
ENSP00000347281	VPS13B	863	0	1	0	0	0	0
ENSP00000347555	ABI1	509	0	1.5	0	0	1	0
ENSP00000347596	EFEMP1	493	0	1	0	0	1	0
ENSP00000347900	PC	529	0	1	0	0	1	0
ENSP00000347969	RPL23A	158	0	1	0	1	5	1
ENSP00000348100	ADD1	662	0	1	0	0	0	0
ENSP00000348442	PSMD12	456	0	1	0	0	0	0
ENSP00000348498	NF1	2818	0	2	1	0	1	2
ENSP00000348786	RAP1A	184	0	1	0	0	0	0
ENSP00000348849	RPS26	115	0	3	0	0	0	2
ENSP00000348921	ENSA	140	0	1	0	0	0	0
ENSP00000348933	CCDC25	208	0	2	1	0	0	0
ENSP00000349212	NACA	215	0	1	2	0	2.5	0
ENSP00000349415	RAB18	206	0	8	1	2	5	1
ENSP00000349560	RAB4B	213	0	2	0	1	1	1
ENSP00000349685	VPS13B	3997	0	1	0	0	0	0
ENSP00000349738	TRAPPC11	1086	0	1	0	0	0	0
ENSP00000349856	PRRC2B	2229	0	1	0	0	0	0
ENSP00000350052	POTEF	1075	0	1	0	0	0	0
ENSP00000350187	WDR7	1457	0	1	0	0	0	0
ENSP00000350332	MYBPC2	1141	0	1	1	1	0	0
ENSP00000350757	GMFB	142	0	1	0	1	1	0
ENSP00000350848	HMGN5	282	0	1	0	0	0	0
ENSP00000350885	RIMBP3C	1545	0	1	0	0	0	0
ENSP00000351015	NF1	2839	0	1	0	0	0	1
ENSP00000351207	KIAA1671	1806	0	1	0	0	0	0
ENSP00000351346	VPS13B	4022	0	1	0	0	0	0
ENSP00000351608	PRRT2	340	0	3	1	0	0	0
ENSP00000351635	GNB5	353	0	2	1	2	1	1
ENSP00000351851	NUMA1	2101	0	1	0	0	0	0
ENSP00000351875	ANKRD30B	1392	0	1	2	0	0	0
ENSP00000352088	MYLK	1863	0	1	0	0	0	0
ENSP00000352143	RPL17-C18orf32	190	0	2	1	1	1	0
ENSP00000352667	MBP	186	0	1	0	1	1	0
ENSP00000352948	AK3	157	0	1	0	0	0	0
ENSP00000353124	ARRB1	410	0	1	1	0	1	0
ENSP00000353444	RAB5B	215	0	5	5	0	4	3
ENSP00000353512	PARP9	854	0	1	0	0	0	0
ENSP00000353660	PEA15	130	0	1	0	0	1	0
ENSP00000353701	DPP3	737	0	4	2	2	4	1
ENSP00000353786	VPS29	186	0	1	0	0	2	1
ENSP00000353998	VKORC1L1	176	0	1	0	0	0	0
ENSP00000354228	CACNA2D2	1076	0	1	0	1	1	1
ENSP00000354251	NCKAP1	1134	0	3	0	4	7	5
ENSP00000354310	NSD1	2593	0	2	0	1	0	0
ENSP00000354346	MATR3	847	0	3	2	0	4	0
ENSP00000354394	STAT1	750	0	1	0	0	0	0
ENSP00000354505	COL4A5	1685	0	1	0	0	0	0
ENSP00000354674	SRPK1	671	0	1	1	0	1	0
ENSP00000354739	RPL12	165	0	2	0	0	4	0
ENSP00000354997	CLSTN1	971	0	1	0	0	1	2
ENSP00000355122	VAMP1	117	0	1	1	0	1	2
ENSP00000355316	GRM3	879	0	2	2	1	0	1
ENSP00000355556	GNG4	75	0	1	0	0	0	0
ENSP00000355651	RAB4A	218	0	1	0	1	1	1

ENSP00000355728	CDC42BPA	1754	0	1	0	0	0	0
ENSP00000355743	PHF10	496	0	2	0	0	0	0
ENSP00000355862	PARK2	316	0	1	0	0	0	0
ENSP00000355926	RPS6KC1	1054	0	1	0	0	0	0
ENSP00000355927	RPS6KC1	1066	0	1	0	0	0	0
ENSP00000356038	S100B	94	0	2	2	1	5	4
ENSP00000356154	SCAF8	1337	0	1	1	0	0	1
ENSP00000356367	CRB1	674	0	1	1	0	2	1
ENSP00000356403	KCNT2	1111	0	1	0	0	0	0
ENSP00000356450	STXBP5	1098	0	2	0	1	0	0
ENSP00000356736	GORAB	246	0	1	0	0	0	0
ENSP00000356856	TMCO1	239	0	1	0	0	0	0
ENSP00000356858	MGST3	166	0	1	0	0	0	1
ENSP00000357018	LY9	193	0	1	0	0	0	0
ENSP00000357555	RPS27	84	0	1	1	0	0	0
ENSP00000357616	DPYSL4	412	0	9	6	0	4	5
ENSP00000357642	MKI67	2896	0	1	0	0	0	0
ENSP00000357692	S100A16	103	0	1	0	0	0	0
ENSP00000357791	HRNR	2850	0	3	1	0	0	0
ENSP00000357851	BUB3	326	0	1	1	0	0	0
ENSP00000357858	BUB3	328	0	1	0	0	0	0
ENSP00000357950	CERS2	380	0	1	0	0	0	0
ENSP00000358106	PREP	710	0	1	0	0	5	0
ENSP00000358126	VPS45	570	0	1	0	0	1	0
ENSP00000358142	SV2A	742	0	1	1	3	0	0
ENSP00000358155	HIST2H2AA4	130	0	2	2	2	2	1
ENSP00000358527	TSPAN2	193	0	1	0	0	1	0
ENSP00000358548	NRAS	189	0	2	0	0	1	2
ENSP00000358624	MAGI3	1125	0	3	3	2	3	2
ENSP00000358939	SARS	536	0	2	1	0	0	0
ENSP00000358966	IMPG1	797	0	1	0	0	0	0
ENSP00000359714	PRKACB	264	0	1	1	0	0	0
ENSP00000359718	PRKACB	245	0	2	3	0	0	0
ENSP00000359719	PRKACB	398	0	1	0	0	1	1
ENSP00000359873	HS6ST2	605	0	1	0	0	0	0
ENSP00000360125	PGM1	562	0	1	2	0	3	0
ENSP00000360127	DLEU2L	55	0	1	0	0	0	0
ENSP00000360472	LGI1	557	0	2	1	0	2	0
ENSP00000360666	ABCA2	2435	0	2	2	0	3	3
ENSP00000360689	TNKS2	1166	0	1	0	0	0	0
ENSP00000360742	UBE2V1	170	0	4	0	1	3	0
ENSP00000360937	CMPK1	169	0	1	0	0	2	0
ENSP00000360939	CMPK1	228	0	1	0	0	2	0
ENSP00000361076	RPL7A	266	0	2	3	2	0	1
ENSP00000361283	RPS8	188	0	1	0	0	1	0
ENSP00000361634	SH3GLB2	404	0	1	2	1	0	0
ENSP00000361877	ODF2	638	0	1	0	0	0	0
ENSP00000361940	RPL36A	80	0	1	0	0	0	0
ENSP00000362022	TRMT2B	494	0	1	0	0	0	0
ENSP00000362335	SAR1A	198	0	2	0	0	0	0
ENSP00000362389	HDLBP	973	0	1	0	0	0	0
ENSP00000362576	YARS	528	0	2	0	0	1	0
ENSP00000362664	GIGYF2	1299	0	2	1	0	0	1
ENSP00000362727	DENND1A	1009	0	1	0	0	0	0
ENSP00000362729	RPS4X	122	0	1	1	0	0	0
ENSP00000362738	PSMD1	922	0	1	0	1	1	0
ENSP00000362805	OGT	1036	0	1	0	1	1	0
ENSP00000362820	SRSF3	164	0	1	0	0	0	0

ENSP00000362873	NDUFA8	172	0	1	0	0	0	0
ENSP00000363041	CISD1	108	0	0.5	0	0	1	0
ENSP00000363052	EPB41L1	880	0	1	0	0	0	0
ENSP00000363169	RPS10	172	0	1	0	0	0	0
ENSP00000363613	RAPH1	619	0	1	0	0	0	0
ENSP00000363625	LYPLA2	164	0	1	0	0	0	0
ENSP00000363639	TXN	85	0	1	1	0	0	0
ENSP00000363676	RPL11	178	0	1	0	0	2	1
ENSP00000363752	LUZP1	1076	0	1	0	0	0	0
ENSP00000363818	ZNF462	1449	0	1	0	0	0	0
ENSP00000363905	PARD3	1001	0	1	0	0	0	0
ENSP00000363972	ZC4H2	224	0	1	0	0	0	0
ENSP00000364348	CCDC180	1701	0	1	0	0	0	0
ENSP00000364630	PADI2	437	0	1	0	0	0	0
ENSP00000364664	SLC4A10	1099	0	1	0	0	2	0
ENSP00000364805	HSPA1L	641	0	2	1	0	0	0
ENSP00000364887	DYNC2H1	4307	0	1	0	0	0	0
ENSP00000364943	DDAH2	285	0	2	2	1	0	1
ENSP00000365025	CSNK2B	215	0	1	1	1	0	1
ENSP00000365220	TPP2	1262	0	1	0	0	2	2
ENSP00000365233	TPP2	1249	0	1	0	1	3	2
ENSP00000365536	AF196779.12	292	0	1	0	0	0	0
ENSP00000365566	PRAF2	160	0	1	0	0	0	0
ENSP00000365569	FLOT1	427	0	1	0	1	0	0
ENSP00000365570	PRAF2	178	0	2	0	0	0	0
ENSP00000365683	C10orf67	296	0	1	0	0	0	0
ENSP00000365694	PPP1R10	940	0	1	0	0	0	0
ENSP00000366013	GNB2L1	273	0	3	2	0	1	1
ENSP00000366085	MOG	131	0	1	0	0	1	0
ENSP00000366679	HIST1H2AH	128	0	1	0	0	0	1
ENSP00000366819	UCHL3	230	0	1	0	0	0	0
ENSP00000366886	KLHL21	597	0	1	0	0	0	0
ENSP00000366913	ATP2A2	50	0	1	3	0	1	2
ENSP00000367073	ACOT7	329	0	2	3	0	2	2
ENSP00000367263	AHNAK	5890	0	3	0	0	0	0
ENSP00000367320	TBC1D31	644	0	1	0	0	0	0
ENSP00000367336	RPL23	91	0	3	1	0	3	2
ENSP00000367498	LRRC47	583	0	1	1	1	1	0
ENSP00000367557	RAB4B	121	0	1	0	0	1	0
ENSP00000367590	REEP2	254	0	1	0	0	0	0
ENSP00000367681	FAM213B	231	0	1	0	0	0	0
ENSP00000367806	RPS16	152	0	2	0	1	1	0
ENSP00000368025	WDR87	2873	0	2	0	0	4	1
ENSP00000368308	NRCAM	1236	0	1	0	1	1	0
ENSP00000368565	DNAJC2	621	0	2	0	0	1	1
ENSP00000369038	EEF1E1	174	0	1	0	0	0	0
ENSP00000369066	POSTN	779	0	1	1	0	1	1
ENSP00000369127	DNAJA1	397	0	1	0	0	0	0
ENSP00000369361	KRT80	422	0	3	0	0	0	0
ENSP00000369396	SLC4A11	891	0	1	0	0	0	0
ENSP00000369399	SLC4A11	918	0	1	0	0	0	0
ENSP00000369586	HBE1	147	0	1	0	1	1	1
ENSP00000369741	RPS6	91	0	4	0	1	1	0
ENSP00000369743	RPS6	218	0	2	1	0	0	0
ENSP00000369784	TUBAL3	446	0	4	2	1	1	3
ENSP00000369981	SH3GL2	352	0	5	1	3	5	3
ENSP00000369989	KATNAL1	490	0	1	0	0	0	1
ENSP00000370113	PPP2R2A	447	0	1	2	0	1	0

ENSP00000370462	PFKP	202	0	1	1	0	0	2
ENSP00000370517	PFKP	784	0	8	4	1	4	8.5
ENSP00000371230	AK3	227	0	1	0	0	0	0
ENSP00000371236	GART	1010	0	1	0	0	0	0
ENSP00000371594	GNG7	68	0	1	1	0	2	1
ENSP00000371931	SYNJ1	1526	0	3	0	2	1	3
ENSP00000372067	PRMT8	394	0	1	0	0	1	0
ENSP00000372750	CHRM5	532	0	1	0	0	0	0
ENSP00000374118	SMG1	3662	0	1	0	0	1	0
ENSP00000374443	LYST	3801	0	1	1	1	2	0
ENSP00000375097	SBF1	1867	0	1	0	0	0	1
ENSP00000375918	ACSL3	720	0	1	0	0	0	0
ENSP00000376136	STAT1	712	0	1	0	0	0	0
ENSP00000376176	RAN	216	0	1	0	0	1	1
ENSP00000376214	PKLR	543	0	1	1	0	1	1
ENSP00000376297	DYNLL1	89	0	3	3	1	3	6
ENSP00000376369	CDCA4	241	0	1	0	0	0	0
ENSP00000376399	S100A13	98	0	2	2	0	0	1
ENSP00000376493	MECOM	1051	0	1	1	0	0	0
ENSP00000376506	PDCD10	212	0	1	0	0	0	0
ENSP00000376620	WARS	471	0	1	0	0	1	0
ENSP00000376996	GCC2	247	0	1	0	0	0	1
ENSP00000377298	NUMA1	2115	0	1	0	0	0	0
ENSP00000377403	GRIA2	836	0	2	0	2	0	0
ENSP00000377527	PC	1178	0	2	0	0	3	0
ENSP00000377546	KRT15	456	0	1	0	0	0	0
ENSP00000377616	KRT222	295	0	1	1	0	0	0
ENSP00000377723	GLTSCR1L	1079	0	1	1	2	1	0
ENSP00000377865	RPL23	140	0	1	0	0	0	1
ENSP00000378057	EFEMP1	493	0	1	0	0	1	0
ENSP00000378160	RPL34	117	0	1	0	0	0	0
ENSP00000378279	MATR3	895	0	1	0	0	0	0
ENSP00000378487	VDAC1	283	0	1	0	0	0	0
ENSP00000378514	PPM1A	382	0	1	0	0	0	0
ENSP00000378625	MAPK3	357	0	4	3	0	3	1
ENSP00000378626	MAPK3	311	0	1	1	0	0	0
ENSP00000378628	MAPK3	335	0	1	0	0	0	0
ENSP00000378669	ALDOA	418	0	1	2	2	3	6
ENSP00000378740	ASL	444	0	1	0	0	0	0
ENSP00000379051	PURB	312	0	1	1	0	0	1
ENSP00000379704	SEPT3	358	0	1	0	0	0	0
ENSP00000379729	RPL7	208	0	2	0	0	0	0
ENSP00000379888	RPS8	208	0	1	1	0	2	1
ENSP00000379969	OTUD5	566	0	1	0	0	0	0
ENSP00000380070	GAPDH	335	0	3	1	0	1	0
ENSP00000380158	RPL32	135	0	1	0	0	1	1
ENSP00000380175	RHOG	191	0	2	0	0	1	3
ENSP00000380746	FBXL16	479	0	1	0	0	0	0
ENSP00000380769	S100B	92	0	1	0	2	1	1
ENSP00000380938	CEP290	1539	0	1	0	0	0	0
ENSP00000381047	C21orf2	375	0	1	0	0	0	0
ENSP00000381064	INTS10	710	0	1	0	0	0	0
ENSP00000381167	DYNC2H1	4314	0	1	0	0	0	0
ENSP00000381206	RDH16	317	0	1	0	0	0	0
ENSP00000381402	GPM6B	179	0	4	3	0	0	2
ENSP00000381461	PPME1	400	0	1	0	1	0	0
ENSP00000381604	GSTP1	174	0	2	0	0	0	0
ENSP00000381607	GSTP1	210	0	3	0	0	1	0

ENSP00000381982	FER1L6	1857	0	1	0	0	0	0
ENSP00000382030	RAD51AP2	1159	0	1	0	0	0	0
ENSP00000382174	MYO5A	228	0	1	0	0	0	0
ENSP00000382177	MYO5A	1855	0	1	0	0	0	0
ENSP00000382341	RABGGTA	567	0	1	1	0	0	0
ENSP00000382362	PHB2	299	0	1	0	0	0	0
ENSP00000382857	USP16	822	0	1	0	0	0	0
ENSP00000383125	USP14	483	0	1	0	0	0	0
ENSP00000383702	VAMP1	116	0	1	1	0	1	2
ENSP00000383937	RPS9	139	0	1	0	1	0	0
ENSP00000384062	ATL2	412	0	2	2	0	0	1
ENSP00000384371	BCL6	706	0	1	0	1	0	0
ENSP00000384582	GPR98	6306	0	1	1	0	0	0
ENSP00000384863	XPO1	1071	0	2	0	1	0	0
ENSP00000385018	RPS7	194	0	3	0	0	0	3
ENSP00000385152	KIAA1671	1806	0	1	0	0	0	0
ENSP00000385181	AAK1	863	0	2	0	0	0	0
ENSP00000385286	RPS7	194	0	1	0	0	0	3
ENSP00000385404	ACP1	112	0	1	0	0	0	0
ENSP00000385432	SAR1B	198	0	2	0	0	0	0
ENSP00000385498	PPP1R7	274	0	1	0	1	1	0
ENSP00000385549	DPYSL5	564	0	1	1	3	1	1
ENSP00000385880	PFKP	210	0	7	5	0	8	6
ENSP00000386153	RAP1GDS1	607	0	1	1	0	0	1
ENSP00000386223	RAP1GDS1	558	0	1	0	0	0	0
ENSP00000386452	C17orf104	952	0	1	0	0	0	0
ENSP00000386458	PRPF40A	930	0	1	0	0	0	0
ENSP00000386770	DNAH10	4471	0	1	0	0	0	0
ENSP00000386840	XIRP2	3549	0	2	1	1	0	1
ENSP00000386929	ACTG2	333	0	9	3	3	3	7
ENSP00000386957	KIAA1107	1409	0	1	0	0	0	0
ENSP00000386969	SERPINE2	397	0	1	0	0	0	0
ENSP00000387120	RPL31	79	0	1	0	0	0	0
ENSP00000387123	ALDH7A1	539	0	1	0	0	0	0
ENSP00000387140	HK2	889	0	1	0	0	1	0
ENSP00000387189	TCEB2	113	0	1	0	0	0	0
ENSP00000387279	CYCS	105	0	1	0	1	0	1
ENSP00000387307	RAB6C	254	0	2	0	0	0	2
ENSP00000387348	CBX3	101	0	1	0	0	0	0
ENSP00000387682	NME1	60	0	1	0	0	0	0
ENSP00000387727	NCOR1	537	0	1	0	0	0	0
ENSP00000387904	KRT4	500	0	2	6	2	0	0
ENSP00000387943	DDAH2	226	0	1	1	1	0	1
ENSP00000388009	LEFTY2	332	0	1	0	0	0	0
ENSP00000388353	ATP6V1B1	496	0	4	2	1	3	2
ENSP00000388529	RPL15	204	0	1	0	0	0	0
ENSP00000388538	APP	660	0	1	3	0	3	0
ENSP00000388742	PAFAH1B2	132	0	1	1	1	1	2
ENSP00000388856	HPCAL1	103	0	1	0	0	0	0
ENSP00000388942	SCRN1	434	0	1	0	0	0	0
ENSP00000388990	GLS	114	0	2	0	0	0	0
ENSP00000389033	CAV1	138	0	1	0	0	0	0
ENSP00000389090	PTGES3	124	0	5	0	1	2	2
ENSP00000389103	RPL23A	156	0	1	0	1	5	1
ENSP00000389351	RPSA	300	0	1	1	0	0	0
ENSP00000389602	RNPEP	344	0	1	1	0	3	1
ENSP00000390077	GNB2	232	0	1	5	1	0	1
ENSP00000390090	DNM1L	156	0	1	0	0	1	0

ENSP00000390138	RGS7	393	0	2	0	0	1	0
ENSP00000390148	ATP6V1G2	158	0	2	0	0	2	3
ENSP00000390630	RIMBP3C	1639	0	1	0	0	0	0
ENSP00000390839	RPL11	131	0	1	0	0	0	0
ENSP00000390975	SYNE1	8749	0	1	1	0	0	0
ENSP00000391056	SYT1	419	0	4	5	0	8	3
ENSP00000391160	TAGLN3	199	0	1	0	0	1	0
ENSP00000391316	GPX1	98	0	1	0	0	0	0
ENSP00000391444	ST7	62	0	1	0	0	0	0
ENSP00000392314	AK1	135	0	3	0	0	3	1
ENSP00000392349	RPS18	34	0	2	0	1	0	0
ENSP00000392618	GPR98	1967	0	1	1	0	0	0
ENSP00000392718	POTEI	1075	0	1	2	0	0	0
ENSP00000392735	AP1B1	173	0	2	1	0	0	0
ENSP00000393417	CASP14	242	0	1	0	0	0	0
ENSP00000393485	ATIC	200	0	1	1	0	0	0
ENSP00000393496	ATP5O	74	0	1	0	0	0	0
ENSP00000393527	POR	725	0	1	0	0	0	0
ENSP00000393611	FXYD1	92	0	1	1	0	0	0
ENSP00000394041	PRKAR2A	404	0	2	1	0	3	0
ENSP00000394213	ACTR3	41	0	3	1	0	1	0
ENSP00000394470	GNB2L1	153	0	1	1	0	0	0
ENSP00000394546	NHSL1	1610	0	1	0	0	0	0
ENSP00000394873	MOG	131	0	1	0	0	1	0
ENSP00000395451	PYGB	245	0	6	1	1	6	3
ENSP00000395472	RANBP1	163	0	1	0	0	0	0
ENSP00000395791	RHOC	91	0	1	0	0	0	0
ENSP00000396212	TUBA4A	156	0	1	0	1	2	0
ENSP00000396249	ABI2	480	0	1	0	0	0	0
ENSP00000396475	ARPC2	91	0	1	1	1	1	0
ENSP00000396707	CKMT1B	103	0	1	0	1	1	0
ENSP00000397555	R3HCC1	480	0	1	0	0	0	0
ENSP00000397937	CAMK2B	542	0	4	1	1	1	0
ENSP00000398147	FKBP2	142	0	1	1	0	0	0
ENSP00000398487	ABI2	44	0	1	0	0	0	0
ENSP00000398629	GNG4	75	0	1	0	0	0	0
ENSP00000398812	FKBPL	349	0	1	0	0	1	0
ENSP00000398839	PPP1CB	125	0	1	2	2	1	0
ENSP00000398888	RPL11	174	0	2	0	0	2	1
ENSP00000399058	DNAJC2	188	0	1	1	0	0	0
ENSP00000399119	QARS	282	0	1	0	0	0	0
ENSP00000399177	DHDDS	198	0	1	0	0	0	0
ENSP00000399357	DDAH2	285	0	1	1	0	0	0
ENSP00000399610	RYR3	4865	0	1	0	0	0	0
ENSP00000399698	SAR1A	198	0	2	0	0	0	0
ENSP00000399838	BPGM	259	0	1	0	0	1	0
ENSP00000399862	CNOT10	804	0	1	0	0	0	0
ENSP00000400764	TST	124	0	1	1	1	0	1
ENSP00000400887	PPIP5K1	1433	0	1	0	0	0	0
ENSP00000401559	SYT1	181	0	2	3	0	2	1
ENSP00000401614	UBP1	148	0	2	0	0	2	0
ENSP00000401779	SNX3	130	0	1	0	0	0	0
ENSP00000402024	IL2RA	109	0	1	0	0	0	0
ENSP00000402126	PRPSAP1	104	0	1	0	0	0	0
ENSP00000402425	CD59	128	0	2	0	1	1	1
ENSP00000402430	ATP6V1G2	78	0	1	0	0	0	0
ENSP00000402446	WDR64	914	0	1	1	0	1	1
ENSP00000402478	HSPD1	83	0	1	0	0	1	0

ENSP00000402515	SMG1	3661	0	1	0	0	1	0
ENSP00000402854	DDAH2	188	0	1	1	0	0	0
ENSP00000402996	ARL8B	163	0	1.5	0	0	0	0
ENSP00000403154	DDAH2	285	0	1	0	0	0	0
ENSP00000403172	RPL6	288	0	1	1	0	1	0
ENSP00000404042	TMED4	227	0	1	2	1	0	0
ENSP00000404227	NIPSNAP1	128	0	2	1	0	0	0
ENSP00000404309	SIRT2	237	0	2	0	0	1	0
ENSP00000404375	RPL36A	142	0	2	0	0	0	0
ENSP00000404616	PDHA1	121	0	3	2	1	0	1
ENSP00000404631	CACNA2D2	1144	0	1	0	4	2	1
ENSP00000404864	OLA1	103	0	1	0	0	0	0
ENSP00000404866	ECHDC1	226	0	2	1	0	0	0
ENSP00000404944	EHD1	374	0	2	2	1	0	0
ENSP00000404978	NEDD1	660	0	1	0	0	0	0
ENSP00000405033	TRAPPC4	165	0	1	0	0	0	0
ENSP00000406112	CALM2	65	0	2	1	1	2	1
ENSP00000406372	ATP6V1G2	118	0	1	0	0	0	2
ENSP00000407034	PRDX1	171	0	1	0	0	0	0
ENSP00000407310	PPP1R10	940	0	1	0	0	0	0
ENSP00000407552	GAS7	476	0	1	0	0	0	0
ENSP00000407821	MYH11	1945	0	1	0	0	0	0
ENSP00000407872	RAB18	291	0	2	0	0	1	0
ENSP00000408468	YWHAH	39	0	3	2	0	1	0
ENSP00000408578	ARPC1A	345	0	1	0	0	0	1
ENSP00000408678	SEPT5	301	0	3	2	0	0	0
ENSP00000408796	SAMD9L	1584	0	1	1	0	0	0
ENSP00000409424	RGPD6	56	0	1	0	0	0	0
ENSP00000409510	CSNK2B	215	0	1	1	1	0	0
ENSP00000409811	ATL2	175	0	1	1	0	0	0
ENSP00000409967	FSCN1	120	0	1	0	0	0	0
ENSP00000410208	GORASP2	464	0	1	0	0	0	0
ENSP00000410240	RPL37A	68	0	1	0	0	2	1
ENSP00000410249	XPO7	1088	0	1	0	0	0	0
ENSP00000410465	HMGB1	97	0	1	0	0	0	0
ENSP00000410848	RPSA	263	0	2	2	3	0	0
ENSP00000411121	ACP1	70	0	1	0	0	0	0
ENSP00000411176	RPL15	175	0	1	0	0	0	0
ENSP00000411251	DCTN4	403	0	1	0	0	0	0
ENSP00000411719	MPST	297	0	1	0	0	0	0
ENSP00000412083	ERP29	53	0	2	0	1	1	1
ENSP00000412646	TUBA8	467	0	1	0	0	3	3
ENSP00000412663	GSK3A	483	0	1	0	0	0	0
ENSP00000412976	RPL31	115	0	4	0	0	3	2
ENSP00000413502	RANBP1	145	0	2	0	0	0	1
ENSP00000413600	NAPB	259	0	2	2	2	1	1
ENSP00000413642	CNTN4	1026	0	1	0	1	0	0
ENSP00000414363	EEF1E1	139	0	1	0	0	0	0
ENSP00000414507	PRMT8	385	0	1	0	0	1	0
ENSP00000414568	OLA1	116	0	2	0	2	1	0
ENSP00000414892	PTGES3	139	0	3	0	0	1	1
ENSP00000414918	PPP1CB	138	0	1	1	0	0	0
ENSP00000415033	UBA1	270	0	1	0	1	1	0
ENSP00000415491	PHYHIP	330	0	1.5	0	0	0	0
ENSP00000415949	HK1	290	0	1	1	1	2	3
ENSP00000416110	RPS18	152	0	3	0	0	5	0
ENSP00000416355	FLOT1	238	0	1	0	0	0	0
ENSP00000416479	CYCS	101	0	1	0	0	0	1

ENSP00000416706	ACTBL2	376	0	7	9	3	8	4
ENSP00000417175	VAPB	243	0	3	2	2	4	1
ENSP00000417443	TMED4	186	0	1	1	0	0	0
ENSP00000417445	ERC2	957	0	1	0	0	0	0
ENSP00000417495	TFAP2A	328	0	1	0	0	0	0
ENSP00000417568	CADPS	190	0	4	0	4	0	0
ENSP00000417762	SELT	137	0	1	0	0	0	0
ENSP00000417904	TAGLN3	115	0	1	0	0	0	0
ENSP00000417932	SELT	137	0	1	0	0	0	0
ENSP00000418082	RPL37A	92	0	1	0	0	2	1
ENSP00000418137	DNAH12	2316	0	1	1	0	1	0
ENSP00000418161	RHEB	79	0	1	0	0	1	0
ENSP00000418512	SRI	155	0	1	0	0	0	0
ENSP00000418523	PFN2	91	0	1	2	0	1	0
ENSP00000418537	ADCY5	919	0	1	0	0	0	0
ENSP00000418540	RAB6B	71	0	2	0	1	0	1
ENSP00000418894	PARP9	710	0	1	0	0	0	0
ENSP00000418929	SYNPR	202	0	1	0	0	0	0
ENSP00000419021	ARL6IP5	59	0	1	0	0	0	0
ENSP00000419346	AHCYL2	518	0	1	0	1	0	0
ENSP00000419380	PRPS2	200	0	1	0	0	0	0
ENSP00000419381	RAB6B	195	0	2	0	1	5	0
ENSP00000419740	NSUN4	384	0	1	0	0	0	0
ENSP00000419941	RAB6B	124	0	2	0	0	0	0
ENSP00000420311	RPL23	140	0	1	0	0	0	0
ENSP00000420392	RFC2	52	0	1	0	0	0	0
ENSP00000420417	PFN2	91	0	1	1	0	1	0
ENSP00000420675	TAGLN3	163	0	4	0	2	2	2
ENSP00000420900	RPS23	55	0	1	0	0	0	0
ENSP00000421027	ALB	459	0	1	0	0	0	0
ENSP00000421107	CANX	308	0	1	1	3	1	1
ENSP00000421340	CDV3	188	0	1	0	0	0	0
ENSP00000421351	UBE2V1	89	0	1	0	0	1	0
ENSP00000421458	ENSA	105	0	1	0	0	0	0
ENSP00000421465	DBN1	124	0	3	0	0	0	0
ENSP00000421892	HSPA9	102	0	1	2	0	2	2
ENSP00000422078	SUB1	127	0	1	0	0	0	0
ENSP00000422374	PPP2R2C	430	0	1	1	0	1	0
ENSP00000422768	GNB2L1	269	0	3	3	0	1	1
ENSP00000422784	ALB	604	0	2	0	1	0	0
ENSP00000423144	OXCT1	123	0	1	0	0	0	0
ENSP00000423316	RPL34	117	0	2	0	0	0	0
ENSP00000423348	UCHL1	156	0	1	0	0	1	0
ENSP00000423485	GNPDA1	289	0	1	0	0	0	0
ENSP00000423977	EIF4E	245	0	1	2	0	0	1
ENSP00000424419	UFSP2	112	0	1	0	0	0	0
ENSP00000424494	ATP2B2	1154	0	2	2	2	2	3
ENSP00000424796	RPL9	91	0	2	0	0	0	0
ENSP00000424925	SEPT11	432	0	1	1	0	1	0
ENSP00000425466	SAR1B	170	0	2	0	0	0	0
ENSP00000425561	EIF4E	248	0	1	1	0	0	0
ENSP00000425581	NME1-NME2	152	0	4	0	2	1	1
ENSP00000425732	HNRNPH1	172	0	1	3	0	1	0
ENSP00000425742	CRMP1	570	0	3	0	0	1	0
ENSP00000425841	WDR70	387	0	1	0	0	0	0
ENSP00000425865	RPS23	134	0	1	0	0	0	0
ENSP00000426101	GNB2L1	233	0	1	2	0	1	1
ENSP00000426377	QDPR	162	0	5	3	2	6	3

ENSP00000426470	CTBP1	202	0	1	0	0	0	1
ENSP00000426555	CANX	131	0	1	1	1	0	1
ENSP00000426658	REEP2	191	0	1	0	0	0	0
ENSP00000426715	VCAN	354	0	1	0	0	1	0
ENSP00000426895	UCHL1	207	0	1	2	1	0	1
ENSP00000427388	HNRNPH1	168	0	1	1	0	0	1
ENSP00000427463	SPCS3	180	0	2	0	0	0	0
ENSP00000427499	HINT1	103	0	3	2	0	2	2
ENSP00000427572	EEF1E1	136	0	1	0	0	0	0
ENSP00000427687	WDR1	606	0	1	1	1	2	4
ENSP00000427713	ERGIC1	138	0	1	0	0	1	0
ENSP00000427778	PROSC	152	0	1	0	0	1	0
ENSP00000427886	PROSC	137	0	1	1	1	1	0
ENSP00000428061	LUZP1	273	0	1	0	0	0	0
ENSP00000428093	NCALD	74	0	1	0	1	0	0
ENSP00000428141	ATP6V1E2	226	0	2	1	1	2	1
ENSP00000428487	TNIP1	257	0	1	0	0	0	0
ENSP00000428509	RPS14	151	0	1	0	0	0	0
ENSP00000428589	LARP1	815	0	3	0	0	0	4
ENSP00000428604	NDST1	825	0	1	0	0	0	0
ENSP00000428642	PPP1R3B	285	0	1	0	0	0	0
ENSP00000429279	PPP2R2A	132	0	1	1	1	1	0
ENSP00000429283	DOCK2	1322	0	1	0	0	1	0
ENSP00000429333	RPS20	46	0	2	0	0	0	0
ENSP00000429350	PPP3CA	454	0	1	0	1	1	0
ENSP00000429374	RPS20	142	0	1	0	0	0	0
ENSP00000429415	SKP1	142	0	1	0	0	0	0
ENSP00000429436	POLB	193	0	1	0	0	0	0
ENSP00000429473	HS6ST2	645	0	1	0	0	0	0
ENSP00000429861	CCDC25	86	0	2	1	0	0	0
ENSP00000429906	TCEB1	112	0	2	1	0	3	4
ENSP00000430174	ANK1	1040	0	1	0	0	0	0
ENSP00000430267	DPYSL3	209	0	2	1	1	2	1
ENSP00000430491	UBE2V2	154	0	2	0	0	1	0
ENSP00000430971	TNIP1	635	0	1	0	0	0	0
ENSP00000430993	DCTN4	134	0	1	0	0	0	0
ENSP00000431144	PSMC3	301	0	1	0	0	0	0
ENSP00000431301	THY1	161	0	1	1	0	0	0
ENSP00000431567	PCF11	784	0	1	0	0	0	0
ENSP00000432096	RPS13	116	0	1	0	0	0	0
ENSP00000432153	CFL1	90	0	2.5	0	1	2	1
ENSP00000432362	CD59	130	0	1	0	0	1	1
ENSP00000432458	RPS3	44	0	2	0	0	0	0
ENSP00000433005	LIPT2	73	0	1	0	0	0	0
ENSP00000433109	KCNA2	499	0	1	0	0	0	0
ENSP00000433171	ARRB1	257	0	1	0	0	1	0
ENSP00000433316	HSPA8	410	0	2	2	3	2	0
ENSP00000433351	AMPD3	110	0	1	0	0	0	0
ENSP00000433421	RPS3	231	0	3	3	3	2	1
ENSP00000433480	PRMT1	238	0	1	0	0	1	0
ENSP00000433703	RPL8	167	0	1	0	0	0	0
ENSP00000434023	HRAS	170	0	1	1	0	0	0
ENSP00000434138	VPS51	233	0	1	0	0	0	0
ENSP00000434329	PPP5C	485	0	1	0	0	0	0
ENSP00000434535	RPL8	205	0	1	0	0	0	0
ENSP00000434706	SPCS2	166	0	1	0	0	0	0
ENSP00000435096	RPS25	125	0	3	1	0	0	1
ENSP00000435157	GSTM2	147	0	1	1	1	1	0

ENSP00000435238	SYTL2	910	0	1	0	0	0	1
ENSP00000435289	PAFAH1B2	229	0	1	1	1	1	0
ENSP00000435566	RPS3	36	0	1	1	0	0	1
ENSP00000435818	SEPT6	267	0	0.5	1	1	0	0
ENSP00000436059	AP2A2	378	0	1	0	1	0	1
ENSP00000436066	CLP1	425	0	1	0	0	0	0
ENSP00000436556	RPS3	171	0	1	0	0	0	0
ENSP00000436619	GDA	454	0	1	0	0	1	0
ENSP00000437130	NAP1L4	107	0	1	0	0	0	0
ENSP00000437168	GBP3	290	0	1	0	0	0	0
ENSP00000437189	HSPA8	500	0	5	6	0	6	1
ENSP00000437218	CSRP1	153	0	4	0	2	1	2
ENSP00000437457	DENND1A	794	0	1	0	0	0	0
ENSP00000437542	ARHGAP44	591	0	1	0	0	0	0
ENSP00000437607	GLI1	978	0	1	0	0	1	0
ENSP00000437953	POSTN	749	0	1	1	0	1	1
ENSP00000438063	ARF3	102	0	3	0	0	1	2
ENSP00000438193	ALDH5A1	447	0	1	2	0	2	2
ENSP00000438324	KDM3A	1269	0	1	0	0	0	0
ENSP00000438334	SLC12A4	1054	0	1	1	0	2	0
ENSP00000438457	IDH2	322	0	6	6	1	4	1
ENSP00000438842	RAB6A	71	0	1	0	0	0	0
ENSP00000438959	RPL13	164	0	1	0	0	0	0
ENSP00000439010	DNAJA1	240	0	1	0	0	0	0
ENSP00000439291	GLUD1	391	0	3	1	1	1	0
ENSP00000439321	RAB18	142	0	1	0	0	1	0
ENSP00000439447	TCP1	332	0	2	1	0	5	2
ENSP00000439506	CCNT2	488	0	1	0	0	0	0
ENSP00000440502	DPP3	737	0	4	2	2	4	1
ENSP00000440588	SLC8A2	384	0	1	0	2	0	0
ENSP00000440701	ZNF720	475	0	1	0	0	0	0
ENSP00000440763	LGI1	509	0	2	1	0	2	0
ENSP00000441179	RPL12	132	0	2	0	0	1	0
ENSP00000441364	NONO	382	0	2	0	0	0	0
ENSP00000441872	ACOT7	223	0	1	1	0	0	0
ENSP00000442089	RSL1D1	270	0	1	0	0	0	0
ENSP00000442425	DDX31	160	0	1	0	0	0	0
ENSP00000442699	HTATSF1	755	0	1	0	1	0	0
ENSP00000443014	DUSP3	144	0	3	0	0	2	1
ENSP00000443365	SH3GL2	305	0	5	1	3	8	3
ENSP00000443520	SOX5	753	0	1	1	0	0	0
ENSP00000444060	RAP1B	127	0	1	0	0	0	0
ENSP00000444566	MDM2	84	0	1	0	0	0	0
ENSP00000444850	USO1	947	0	1	0	0	0	0
ENSP00000444923	EPSTI1	80	0	1	0	0	0	0
ENSP00000444978	GSTM3	225	0	1	1	1	0	0
ENSP00000445496	OPCML	354	0	1	2	2	1	1
ENSP00000445788	ENO2	103	0	1	0	1	0	0
ENSP00000445794	CAND1	442	0	2	0	0	0	2
ENSP00000445912	AK4	223	0	1	0	0	0	0
ENSP00000446147	IDH2	400	0	2	2	0	0	1
ENSP00000446179	AP3S1	162	0	1	0	0	1	2
ENSP00000446318	RAP1B	165	0	1	0	0	0	0
ENSP00000446353	ARF3	40	0	4	0	0	1	5
ENSP00000446377	ATP6V0A1	483	0	1	3	0	1	1
ENSP00000446477	CSE1L	754	0	3	1	1	1	1
ENSP00000446519	PFKM	176	0	1	0	0	1	1
ENSP00000446787	NAP1L1	209	0	1	1	0	0	1

ENSP00000447677	AC225613.4	115	0	3	0	0	0	1
ENSP00000448039	NACA	213	0	1	2	0	2.5	0
ENSP00000448040	GNL1	429	0	1	0	0	1	1
ENSP00000448046	RPLP0	247	0	3	0	0	1	0
ENSP00000448079	PCBP2	64	0	1	1	0	1	0
ENSP00000448610	DNM1L	725	0	2	2	0	3.5	0
ENSP00000448708	SLC25A3	361	0	1	0	0	1	0
ENSP00000448850	ARL1	164	0	2	0	0	0	0
ENSP00000448861	SYT1	173	0	1	1	0	0	1
ENSP00000448899	RPL18	133	0	3	0	1	0	1
ENSP00000449622	PFKM	165	0	1	0	0	0	1
ENSP00000449651	KRT5	132	0	1	1	0	0	0
ENSP00000450239	METTL7A	180	0	2	1	0	0	0
ENSP00000450339	RPS26	115	0	1	0	0	0	1
ENSP00000450725	HNRNPC	293	0	1	2	0	2	3
ENSP00000450726	TMED10	153	0	2	0	0	2	1
ENSP00000450831	SYNE2	3541	0	1	1	0	0	0
ENSP00000450885	ATP6V1D	148	0	2	2	1	2	0
ENSP00000450977	DGKA	94	0	1	0	0	0	0
ENSP00000451031	ATP6V1D	72	0	1	0	0	1	0
ENSP00000451062	CALM1	98	0	1	0	1	0	1
ENSP00000451255	NAA30	49	0	3	0	0	0	0
ENSP00000451602	CYP46A1	164	0	1	0	0	0	0
ENSP00000451681	EIF2B2	200	0	1	0	0	0	0
ENSP00000451920	GMFB	150	0	2	0	2	1	0
ENSP00000452006	NDRG2	357	0	7	5	2	5	3
ENSP00000452188	CFL2	149	0	2	0	1	1	0
ENSP00000452413	NDRG2	218	0	1	0	0	0	0
ENSP00000452570	SYNE2	6818	0	1	0	0	0	0
ENSP00000452696	RPS17	59	0	2	0	0	0	0
ENSP00000452763	RPL28	170	0	2	0	0	1	0
ENSP00000453223	AP3S2	205	0	1	0	0	0	0
ENSP00000453285	RPL28	89	0	1	0	0	0	0
ENSP00000453508	TLN2	2542	0	1	0	0	0	0
ENSP00000453745	CHRM5	532	0	1	0	0	0	0
ENSP00000453764	RPS27L	100	0	1	0	0	1	1
ENSP00000453770	ANXA2	146	0	1	1	0	0	0
ENSP00000453890	RABGGTA	424	0	1	0	0	0	0
ENSP00000453982	RAB8B	188	0	1	0	0	1	1
ENSP00000454158	BLM	1286	0	1	1	0	0	0
ENSP00000454580	DNAJA2	134	0	1	0	0	0	0
ENSP00000454752	CORO1A	148	0	3	2	2	2	0
ENSP00000454756	SYNGR3	177	0	1	1	0	0	1
ENSP00000454935	NUP93	132	0	1	0	0	0	0
ENSP00000455009	RPL13	211	0	3	1	0	1	0
ENSP00000455122	COQ7	194	0	1	0	0	0	0
ENSP00000455199	ULK3	41	0	1	0	0	0	0
ENSP00000455341	ARL6IP1	101	0	1	0	0	0	0
ENSP00000455360	AARS	280	0	1	0	1	4	1
ENSP00000455552	CORO1A	461	0	2	1	1	0	0
ENSP00000455563	RAB5C	249	0	1	0	0	1	0
ENSP00000456226	PRRT2	394	0	2	0	0	0	0
ENSP00000456568	PTPLAD1	400	0	6	1	2	1	4
ENSP00000456859	SCRIB	1549	0	1	0	0	0	0
ENSP00000457431	GDE1	108	0	1	1	0	0	0
ENSP00000457433	MYOM2	890	0	1	0	0	0	0
ENSP00000457830	PKM	162	0	2	0	0	2	0
ENSP00000457972	RAB5C	216	0	8.5	5.5	0	7	6

ENSP00000457989	NUTF2	127	0	1	0	0	1	3
ENSP00000458307	GGT6	493	0	1	0	0	0	0
ENSP00000458471	DNAH12	2316	0	1	0	0	0	0
ENSP00000458528	RPS15A	130	0	1	0	0	2	1
ENSP00000458585	MMP12	470	0	1	0	0	0	0
ENSP00000458615	RRAS2	47	0	1	1	0	0	1
ENSP00000458947	RPS9	194	0	2	0	0	1	0
ENSP00000459115	DLAT	294	0	1	0	0	0	0
ENSP00000459260	HYOU1	937	0	1	0	0	0	1
ENSP00000459330	KANSL1	122	0	1	0	0	0	0
ENSP00000459529	RAD17	670	0	1	1	0	0	0
ENSP00000459657	TUSC2	110	0	1	0	0	0	0
ENSP00000459770	GNAI2	303	0	1.5	0	0	0	1
ENSP00000460105	CYB5D1	30	0	1	0	0	0	1
ENSP00000460242	RPS25	125	0	1	0	0	0	0
ENSP00000460363	PFN1	165	0	2	2	0	1	1
ENSP00000460697	RFC2	28	0	1	0	0	0	0
ENSP00000460860	RPS2	155	0	1	0	0	0	0
ENSP00000461098	CHMP6	224	0	1	1	0	1	0
ENSP00000461259	RAD17	584	0	1	1	0	0	0
ENSP00000461423	VPS35	47	0	1	0	0	0	0
ENSP00000461685	RRAS2	166	0	1	1	0	2	2
ENSP00000461688	EIF5A	186	0	1	0	1	1	1
ENSP00000461849	BAZ1B	1483	0	1	0	0	0	1
ENSP00000461910	RPL17	105	0	2	1	1	0	0
ENSP00000461945	RAB31	195	0	2	0	1	3	1
ENSP00000462316	PDE4DIP	2346	0	2	0	0	1	0
ENSP00000462408	NF1	1656	0	1	1	0	1	1
ENSP00000462433	PIK3R5	880	0	1	0	0	0	0
ENSP00000462495	PSMC5	398	0	1	0	0	1	0
ENSP00000462592	CLTC	122	0	1	1	0	0	0
ENSP00000462680	PIK3R5	879	0	1	0	0	0	0
ENSP00000462741	EIF4A1	85	0	1	1	0	0	0
ENSP00000463021	RPL26	96	0	2	0	0	0	0
ENSP00000463057	PPIAL4G	164	0	1	0	0	1	1
ENSP00000463190	KPNB1	248	0	1	0	0	0	0
ENSP00000463329	RPL19	128	0	1	0	0	3	4
ENSP00000463461	SEPT4	225	0	1	1	0	1	0
ENSP00000463801	PSMC5	263	0	1	0	0	0	0
ENSP00000463918	TUBAL3	406	0	4	5	1	3	6
ENSP00000463951	TMCO1	104	0	1	0	0	0	0
ENSP00000464240	GAS7	416	0	1	0	0	0	0
ENSP00000464612	RPL13	126	0	1	0	0	1	0
ENSP00000464617	OSBPL1A	285	0	1	0	0	0	0
ENSP00000464821	NUDT16L1	198	0	3	0	1	0	0
ENSP00000464989	GPX4	260	0	1	0	0	0	0
ENSP00000465313	RPL22	47	0	2	0	0	2	0
ENSP00000465719	ACOT7	279	0	1	0	1	1	2
ENSP00000465798	VPS25	111	0	3	0	0	1	1
ENSP00000465875	NCAM1	228	0	3	2	1	2	1
ENSP00000466010	RPS15	152	0	1	0	0	0	0
ENSP00000466089	KLHL21	230	0	1	0	0	0	0
ENSP00000466692	TRAPPC4	257	0	1	0	0	0	0
ENSP00000466767	ATP6V0A1	195	0	2	0	0	0	1
ENSP00000466807	CNP	120	0	1	0	0	0	2
ENSP00000466812	AP2B1	132	0	1	1	0	2	0
ENSP00000466871	MAPRE2	78	0	1	0	0	0	0
ENSP00000466878	ATP6V0A1	794	0	2	0	0	0	1

ENSP00000467124	KRT16	162	0	1	1	0	0	0
ENSP00000467213	MAP2K6	278	0	1	0	0	0	0
ENSP00000467225	VPS25	99	0	1	0	0	1	1
ENSP00000467231	DOCK6	148	0	1	0	0	0	0
ENSP00000467386	ATP9B	289	0	1	0	0	1	0
ENSP00000467430	DNM2	50	0	2	0	0	1	1
ENSP00000467433	UBA2	262	0	1	0	0	0	0
ENSP00000467437	CANT1	401	0	1	0	0	0	0
ENSP00000467444	ENO3	196	0	2	2	0	0	0
ENSP00000467587	RPL27	136	0	1	0	0	2	1
ENSP00000467686	TRAPPC4	126	0	1	0	0	0	0
ENSP00000467727	FXYD1	92	0	2	0	0	0	0
ENSP00000467830	ATP5A1	111	0	1	1	0	0	0
ENSP00000467900	GPX4	166	0	1	0	0	0	0
ENSP00000467932	KRT9	390	0	13	16	0	3	0
ENSP00000467939	RPL27	80	0	1	0	0	2	1
ENSP00000468165	TXNL1	281	0	1	0	0	1	0
ENSP00000468218	ATP9B	192	0	2	0	0	0	2
ENSP00000468247	SNRPD2	78	0	1	0	0	1	0
ENSP00000468367	CTD-2132N18.3	282	0	1	0	0	1	0
ENSP00000468402	RPL23A	175	0	1	0	1	2	0
ENSP00000468498	UBE2O	807	0	1	2	1	0	0
ENSP00000468604	DUSP3	132	0	2	0	0	0	0
ENSP00000468940	UBE2M	69	0	2	0	0	0	0
ENSP00000469125	VBP1	192	0	1	1	0	1	0
ENSP00000469228	RPS19	76	0	1	0	0	2	0
ENSP00000469263	TECR	131	0	3	2	1	2	0
ENSP00000469558	BCAP31	138	0	1	0	0	1	0
ENSP00000469855	UBE2M	114	0	3	0	0	0	1
ENSP00000469989	RPL36A	106	0	2	0	0	0	0
ENSP00000470176	AP2S1	158	0	2	0	0	1	0
ENSP00000470246	RAB4B	213	0	2	0	1	1	1
ENSP00000470447	RPS11	118	0	1	0	0	1	0
ENSP00000470743	RPL10	214	0	2	1	0	1	0
ENSP00000471222	AC024257.1	150	0	1	0	0	0	0
ENSP00000471261	NAPA	139	0	1	0	1	0	0
ENSP00000471340	AP2S1	144	0	3	0	0	2	0
ENSP00000471438	IDH3G	322	0	2	3	0	1	0
ENSP00000471537	ATP2B3	1159	0	1	1	0	0	0
ENSP00000471584	RPL18A	154	0	4	0	0	1	1
ENSP00000471937	NSUN4	151	0	1	0	0	0	0
ENSP00000472080	AP2S1	92	0	2	0	0	1	1
ENSP00000472168	HSD17B10	261	0	1	0	0	0	0
ENSP00000472182	KDELR1	220	0	1	0	0	0	0
ENSP00000472543	MYH14	2003	0	1	0	0	2	0
ENSP00000472834	PGK1	281	0	1	0	0	1	0
ENSP00000473155	RPL18A	141	0	1	0	0	0	0
ENSP00000473408	TCEB1	65	0	1	0	0	0	0
ENSP00000473435	ARPC4	77	0	2	0	1	1	1
ENSP00000473548	PPIF	145	0	1	0	0	0	0
ENSP00000474028	NCAM1	907	0	1	0	0	0	0
ENSP00000474184	RPS9	194	0	1	0	0	1	0
ENSP00000474603	RAB3A	125	0	3	6	2	5	3
ENSP00000474669	ALDH2	117	0	1	0	0	1	0
ENSP00000475027	RPS10-NUDT3	286	0	1	0	0	0	0
ENSP00000475496	SCAMP1	338	0	3	2	0	0	0
ENSP00000475555	PHB2	299	0	1	0	0	0	0
ENSP00000475829	TPI1	98	0	5	2	0	5	3

ENSP00000476975	DSTN	148	0	3	0	0	0	0
ENSP00000476985	RPS19	76	0	1	0	0	0	0
ENSP00000477075	TKT	576	0	1	1	0	3	1
ENSP00000477164	CDKL3	471	0	3	2	0	4	0
ENSP00000477310	DYNLL2	89	0	2	1	0	1	0
ENSP00000011473	SYPL1	259	0	0	1	0	0	0
ENSP00000023064	SLC7A9	487	0	0	1	0	0	0
ENSP00000046794	LCP2	533	0	0	1	0	0	0
ENSP00000071281	FHL1	323	0	0	1	0	1	0
ENSP00000215473	PCDH11Y	1340	0	0	1	0	0	0
ENSP00000216330	FKBP3	224	0	0	1	0	0	0
ENSP00000216350	PRMT5	576	0	0	2	0	0	0
ENSP00000216455	PSMA3	255	0	0	1	0	0	0
ENSP00000216468	TMED8	325	0	0	1	0	0	0
ENSP00000216840	RABGGTA	567	0	0	1	0	0	0
ENSP00000217131	CTSZ	303	0	0	1	0	0	0
ENSP00000218056	WDR13	485	0	0	1	0	0	0
ENSP00000218343	JADE3	823	0	0	2	0	1	0
ENSP00000219313	PSMD7	324	0	0	1	0	0	0
ENSP00000220616	TG	2768	0	0	1	0	0	0
ENSP00000221476	CKM	381	0	0	1	0	0	0
ENSP00000223029	AIMP2	320	0	0	1	0	0	0
ENSP00000224721	CDH23	3359	0	0	1	1	0	0
ENSP00000225441	RUNDC3A	405	0	0	1	0	1	0
ENSP00000226328	RUFY3	469	0	0	1	1	1	0
ENSP00000229314	GOLT1B	138	0	0	1	1	0	0
ENSP00000230050	RPS12	132	0	0	2	1	0	0
ENSP00000233892	MOB4	193	0	0	1	0	1	0
ENSP00000235453	DENND1B	396	0	0	1	0	0	0
ENSP00000242140	WIPF3	483	0	0	1	0	0	0
ENSP00000242848	ZC3H13	1668	0	0	1	0	0	0
ENSP00000244364	DST	5171	0	0	1	0	0	0
ENSP00000244745	SOX4	474	0	0	1	0	0	0
ENSP00000245544	NUP85	656	0	0	1	0	0	0
ENSP00000246012	CST8	142	0	0	1	0	0	0
ENSP00000247584	HIVEP3	2406	0	0	1	0	0	0
ENSP00000251527	ESYT2	893	0	0	1	0	0	0
ENSP00000252288	GAMT	236	0	0	1	0	0	0
ENSP00000252655	RSPH3	560	0	0	1	0	0	0
ENSP00000252677	BMP15	392	0	0	1	0	0	0
ENSP00000253382	ACSS2	714	0	0	1	0	0	0
ENSP00000254722	SERPINF1	418	0	0	1	0	0	0
ENSP00000254770	LANCL2	450	0	0	2	0	0	1
ENSP00000256001	ACTR3B	418	0	0	1	0	1	0
ENSP00000256196	RRAS2	204	0	0	1	0	2	0
ENSP00000258180	KIAA0513	411	0	0	1	0	0	0
ENSP00000258398	TTLL4	1199	0	0	1	0	0	0
ENSP00000259217	SMPD4	837	0	0	1	0	0	0
ENSP00000259667	HINT2	163	0	0	1	0	0	0
ENSP00000260323	UNC13C	2214	0	0	1	0	0	0
ENSP00000260645	ABCG5	651	0	0	1	0	0	0
ENSP00000261479	PSMA6	246	0	0	1	0	2	0
ENSP00000261700	C14orf166	244	0	0	1	0	1	0
ENSP00000261712	PSMD11	422	0	0	1	1	1	0
ENSP00000261726	CUX2	1486	0	0	1	0	0	0
ENSP00000261745	NAA25	972	0	0	0.5	0	0	0
ENSP00000261892	SLC24A1	1099	0	0	1	0	0	0
ENSP00000261973	RBM25	843	0	0	1	0	0	0

ENSP00000262210	CSPP1	1221	0	0	1	0	0	0
ENSP00000262622	CHST8	424	0	0	1	0	0	0
ENSP00000262640	VAMP7	260	0	0	1	0	0	0
ENSP00000262873	MYH7B	1983	0	0	1	0	0	0
ENSP00000263035	DHTKD1	919	0	0	1	0	2	0
ENSP00000263054	SORCS1	1168	0	0	1	0	0	0
ENSP00000263351	ZNF541	679	0	0	1	0	0	0
ENSP00000264405	SEC31A	969	0	0	1	0	0	0
ENSP00000264424	GUCY1B3	619	0	0	2	0	0	0
ENSP00000265094	FBXW11	542	0	0	1	0	0	0
ENSP00000265334	CDKL3	592	0	0	1	0	1	0
ENSP00000266037	DOCK3	2030	0	0	1	0	0	1
ENSP00000266984	CCDC65	497	0	0	1	0	0	0
ENSP00000267176	SBNO1	1392	0	0	1	0	1	0
ENSP00000267996	TPM1	284	0	0	1	0	0	0
ENSP00000268261	PMM2	246	0	0	1	0	2	0
ENSP00000268699	GAS8	478	0	0	1	0	0	0
ENSP00000268717	COPS3	423	0	0	1	0	1	0
ENSP00000269187	SLC39A6	755	0	0	1	0	0	0
ENSP00000269346	TTYH2	534	0	0	1	0	1	0
ENSP00000272198	PPFIA4	701	0	0	1	1	0	0
ENSP00000273062	CTDSP1	261	0	0	1	0	0	0
ENSP00000273480	RNF7	113	0	0	1	0	0	0
ENSP00000275884	DENND2A	1009	0	0	1	0	0	0
ENSP00000280684	KCNA6	529	0	0	1	0	0	0
ENSP00000280892	EIF4E	237	0	0	1	0	0	1
ENSP00000281282	CGNL1	1302	0	0	1	0	1	1
ENSP00000282891	RAD17	584	0	0	1	0	0	0
ENSP00000283131	SMARCA5	1052	0	0	1	0	0	0
ENSP00000283943	TRIP12	1992	0	0	1	0	0	1
ENSP00000284031	DDAH1	285	0	0	1	1	2	0
ENSP00000284136	SEMA3D	777	0	0	1	0	0	0
ENSP00000285093	ACAA2	397	0	0	1	0	0	0
ENSP00000286485	PSD3	513	0	0	1	0	0	0
ENSP00000286744	ADAMTSL3	1691	0	0	1	0	0	0
ENSP00000287546	VPS8	1428	0	0	1	0	0	0
ENSP00000288197	CACNA2D3	1091	0	0	1	0	2	3
ENSP00000288266	APPL1	709	0	0	1	0	0	0
ENSP00000288666	RPS4Y2	263	0	0	1	0	0	0
ENSP00000289577	TMED4	178	0	0	1	1	0	0
ENSP00000291572	AGPAT3	376	0	0	1	0	0	0
ENSP00000292644	PSMC2	433	0	0	1	0	1	0
ENSP00000293774	KRT4	594	0	0	1	0	0	0
ENSP00000295006	CAPN2	700	0	0	1	0	0	0
ENSP00000295878	KIAA1407	936	0	0	1	0	0	0
ENSP00000295962	ABHD6	337	0	0	1	1	0	0
ENSP00000296577	ABCE1	599	0	0	2	0	1	0
ENSP00000297262	RSPH3	464	0	0	1	0	0	0
ENSP00000297689	NFIL3	462	0	0	1	0	0	0
ENSP00000298687	NDRG2	371	0	0	1	0	0	0
ENSP00000299001	PIWIL4	852	0	0	1	0	0	0
ENSP00000299275	PLEKHA5	1116	0	0	1	0	0	0
ENSP00000300061	SCNN1G	649	0	0	1	0	0	0
ENSP00000300773	OR51B5	312	0	0	1	0	0	0
ENSP00000301336	RILP	401	0	0	1	0	0	0
ENSP00000301776	ASRGL1	308	0	0	1	0	0	0
ENSP00000303058	CEP120	986	0	0	2	1	1	1
ENSP00000303147	MAT2A	395	0	0	1	0	1	0

ENSP00000303366	LMAN2	356	0	0	1	0	0	0
ENSP00000304229	HINT1	126	0	0	1	0	1	2
ENSP00000304401	SLFNL1	407	0	0	1	0	0	0
ENSP00000304701	SH2B2	631	0	0	1	0	0	0
ENSP00000305288	NLGN2	835	0	0	1	0	0	0
ENSP00000305988	ALCAM	583	0	0	2	0	3	1
ENSP00000306157	IL7R	459	0	0	1	0	0	0
ENSP00000306607	OR1L8	309	0	0	1	0	0	0
ENSP00000307387	PDCD6IP	868	0	0	1	0	0	3
ENSP00000307479	ARNT2	717	0	0	1	0	0	0
ENSP00000308540	RP11-1407O15.2	478	0	0	1	0	2	1
ENSP00000308780	WHSC1	647	0	0	1	0	0	0
ENSP00000310440	CHMP2A	222	0	0	1	0	0	0
ENSP00000313516	ZNF786	696	0	0	1	0	0	0
ENSP00000317128	PLXND1	1925	0	0	2	0	0	0
ENSP00000317441	AMPH	653	0	0	2	0	0	0
ENSP00000317985	ROCK2	1388	0	0	2	0	0	2
ENSP00000318297	RUVBL1	456	0	0	1	0	0	0
ENSP00000318318	MPI	423	0	0	1	0	0	0
ENSP00000318716	FAM9B	186	0	0	1	0	0	0
ENSP00000320592	CEP112	211	0	0	1	0	0	0
ENSP00000320949	CNOT1	2376	0	0	2	1	0	0
ENSP00000321583	DIS3L	971	0	0	1	0	0	0
ENSP00000321584	IMPDH2	514	0	0	1	0	0	0
ENSP00000322427	ZNF611	705	0	0	1	0	0	0
ENSP00000322791	KIF1A	1690	0	0	1	1	0	1
ENSP00000324573	FLII	1269	0	0	1	0	0	0
ENSP00000325508	C11orf71	147	0	0	1	0	0	0
ENSP00000325518	TSTD1	109	0	0	2	0	0	0
ENSP00000325593	NFKBIZ	596	0	0	1	0	0	0
ENSP00000325690	CARM1	608	0	0	1	0	1	0
ENSP00000326968	FBXO9	437	0	0	1	0	0	0
ENSP00000327431	HBG1	147	0	0	1	0	1	0
ENSP00000327821	XPOT	962	0	0	1	0	1	1
ENSP00000328213	LCK	539	0	0	1	0	0	0
ENSP00000328307	FAM47B	645	0	0	1	0	0	0
ENSP00000328358	KRT79	535	0	0	0.5	0.5	0	0
ENSP00000329664	ATP2C1	888	0	0	1	0	0	0
ENSP00000330349	DDX41	640	0	0	1	0	0	0
ENSP00000330381	WDR5B	330	0	0	1	0	0	0
ENSP00000331211	RBM43	357	0	0	1	0	0	0
ENSP00000332111	EFCAB13	973	0	0	1	0	0	0
ENSP00000335083	PPP2R2C	447	0	0	1	0	0	0
ENSP00000335084	PPP1CC	323	0	0	1	0	0	0
ENSP00000335321	SF3B1	1304	0	0	1	0	0	0
ENSP00000337190	ACSS2	651	0	0	1	0	0	0
ENSP00000339004	FOXG1	489	0	0	1	0	0	0
ENSP00000339659	TRIM2	771	0	0	1	1	1	0
ENSP00000340089	TLR5	858	0	0	1	0	1	0
ENSP00000340465	SLC20A2	652	0	0	1	0	0	0
ENSP00000340900	MIA3	1907	0	0	1	0	0	0
ENSP00000341653	TPM3	232	0	0	1	0	0	0
ENSP00000341779	PPP1CC	337	0	0	1	0	0	0
ENSP00000341837	SLC24A1	1081	0	0	1	0	0	1
ENSP00000342121	RNF6	685	0	0	1	0	0	0
ENSP00000342681	CD5	495	0	0	1	0	0	0
ENSP00000343633	FOXN2	431	0	0	1	0	0	0
ENSP00000343764	TTN	33423	0	0	1	0	0	0

ENSP00000345358	MCRS1	462	0	0	1	0	0	0
ENSP00000345463	APP	695	0	0	1	0	2	0
ENSP00000345873	SLC26A3	764	0	0	1	0	0	0
ENSP00000345964	SORCS1	1198	0	0	1	0	0	0
ENSP00000346169	SEPT6	429	0	0	1	0	0	0
ENSP00000346435	PDE10A	779	0	0	2	0	0	0
ENSP00000346534	SCN8A	1980	0	0	1	0	0	0
ENSP00000346916	TRIM6-TRIM34	842	0	0	1	0	0	0
ENSP00000347032	PIP4K2C	421	0	0	1	1	1	0
ENSP00000348248	GABBR1	844	0	0	1	0	0	0
ENSP00000348611	PHLPP2	1323	0	0	2	0	0	0
ENSP00000349205	PIK3R4	1358	0	0	1	0	0	0
ENSP00000349351	BICD2	855	0	0	1	0	0	0
ENSP00000349768	DCHS2	2916	0	0	1	0	0	0
ENSP00000350191	ALDH5A1	535	0	0	1	0	0	0
ENSP00000350512	COPS5	334	0	0	1	0	1	0
ENSP00000350928	GAD1	594	0	0	1	1	1	0
ENSP00000351140	MAP3K19	1215	0	0	1	1	0	0
ENSP00000351417	OMA1	486	0	0	1	0	0	0
ENSP00000352121	PIK3CG	1102	0	0	1	0	0	0
ENSP00000352123	IKZF1	477	0	0	1	0	0	0
ENSP00000352438	PCBP2	366	0	0	1	0	0	0
ENSP00000352593	DMBT1	1133	0	0	1	0	0	0
ENSP00000353068	CCSER2	567	0	0	1	0	0	0
ENSP00000353633	CPNE8	552	0	0	1	0	0	0
ENSP00000354074	RPS24	130	0	0	1	0	1	1
ENSP00000354508	DST	7461	0	0	1	0	0	0
ENSP00000354558	MTOR	2549	0	0	1	0	0	0
ENSP00000354919	VEPH1	833	0	0	1	0	0	0
ENSP00000354991	ALPK2	2170	0	0	1	0	0	0
ENSP00000355045	FAM179B	1720	0	0	1	0	0	0
ENSP00000355234	MAG	582	0	0	1	0	1	1
ENSP00000355510	WDR64	1081	0	0	1	0	1	1
ENSP00000355846	TLR5	858	0	0	1	0	1	0
ENSP00000355847	PDE10A	779	0	0	2	0	0	0
ENSP00000355963	LPGAT1	370	0	0	1	0	1	0
ENSP00000355964	LPGAT1	370	0	0	1	0	1	0
ENSP00000356255	RNPEP	611	0	0	1	0	2	1
ENSP00000356348	PCMT1	285	0	0	1	1	0	1
ENSP00000356366	DENND1B	426	0	0	1	0	0	0
ENSP00000356705	DNM3	859	0	0	1	0	1	0
ENSP00000357621	HDAC2	458	0	0	1	0	0	0
ENSP00000357838	OAT	439	0	0	2	0	0	0
ENSP00000358232	PNLIPRP3	467	0	0	1	0	0	0
ENSP00000358694	XPNPEP1	623	0	0	1	0	1	0
ENSP00000359230	HAUS7	368	0	0	1	0	0	0
ENSP00000359977	FBXO9	403	0	0	1	0	0	0
ENSP00000360934	SARDH	368	0	0	1	1	0	1
ENSP00000361214	NRG3	696	0	0	1	0	0	0
ENSP00000361254	FAM213A	229	0	0	1	0	2	0
ENSP00000361259	FAM213A	218	0	0	1	0	2	0
ENSP00000361645	SH3GLB2	395	0	0	1	0	0	0
ENSP00000361665	HIVEP3	2405	0	0	1	0	0	0
ENSP00000362928	SRPK1	547	0	0	1	0	1	0
ENSP00000363155	PACSIN1	402	0	0	1	1	1	0
ENSP00000363509	KIF4A	1127	0	0	1	0	0	0
ENSP00000364119	EIF2S2	333	0	0	1	0	0	0
ENSP00000364286	CAPZB	260	0	0	3	0	1	1

ENSP00000364934	CLIC1	241	0	0	1	0	0	0
ENSP00000365438	DGAT2	345	0	0	1	0	0	0
ENSP00000365919	WDR13	485	0	0	1	0	0	0
ENSP00000366036	TBC1D8	1140	0	0	1	0	0	0
ENSP00000366502	CLSTN1	962	0	0	1	1	0	0
ENSP00000366513	CLSTN1	981	0	0	1	1	0	0
ENSP00000367100	TG	2711	0	0	1	0	0	0
ENSP00000367202	RECK	971	0	0	1	0	0	0
ENSP00000368511	CXorf58	332	0	0	1	0	0	0
ENSP00000368574	ACSL6	712	0	0	1	0	0	0
ENSP00000368927	EIF1AX	144	0	0	1	0	0	0
ENSP00000369042	IPO7	1038	0	0	1	1	4	1
ENSP00000369129	DSP	2871	0	0	1	0	0	0
ENSP00000369223	DCLK1	433	0	0	1	0	0	0
ENSP00000369317	KRT6A	564	0	0	1	0	0	0
ENSP00000369600	FRY	3013	0	0	1	0	0	0
ENSP00000369677	EMILIN1	1016	0	0	1	0	0	0
ENSP00000369768	HSPH1	814	0	0	1	1	0	0
ENSP00000370394	RUFY3	620	0	0	1	1	1	0
ENSP00000371221	MTMR6	621	0	0	1	0	0	0
ENSP00000371293	MTUS1	1216	0	0	1	0	0	0
ENSP00000371314	SPATA6L	291	0	0	2	0	0	0
ENSP00000371377	OR1A2	309	0	0	1	0	0	0
ENSP00000371462	MAP1A	3041	0	0	1	0	0	0
ENSP00000371668	FAM179B	995	0	0	1	0	0	0
ENSP00000373191	LNP1	178	0	0	1	0	0	1
ENSP00000373342	CPNE9	503	0	0	1	0	0	0
ENSP00000373378	SDK2	2153	0	0	1	0	0	0
ENSP00000373697	TRIP12	1722	0	0	1	0	0	1
ENSP00000374260	CCDC149	529	0	0	1	0	2	0
ENSP00000374444	LYST	3801	0	0	1	1	1	0
ENSP00000375717	PTOV1	416	0	0	1	0	0	0
ENSP00000375808	ERCC2	682	0	0	1	0	1	0
ENSP00000376048	MAG	626	0	0	1	0	1	1
ENSP00000376914	ATP2C1	973	0	0	1	0	0	0
ENSP00000377004	SPOP	374	0	0	1	1	0	0
ENSP00000377928	ACACA	2346	0	0	1	0	0	0
ENSP00000378422	VKORC1	160	0	0	1	0	0	0
ENSP00000378658	AIMP2	251	0	0	1	0	0	0
ENSP00000379374	FKBP3	224	0	0	1	0	0	0
ENSP00000380170	ZSCAN29	463	0	0	1	0	0	0
ENSP00000381298	LONRF1	773	0	0	1	0	0	0
ENSP00000381331	HDAC2	582	0	0	1	0	0	0
ENSP00000382382	USP47	1375	0	0	1	0	0	0
ENSP00000383862	CAPZB	272	0	0	3	0	1	1
ENSP00000384328	RALB	227	0	0	1	0	1	1
ENSP00000384408	PARG	491	0	0	1	0	0	0
ENSP00000385509	ROCK2	1145	0	0	1	0	0	1
ENSP00000385873	EFTUD2	937	0	0	1	0	1	0
ENSP00000386353	MAT2A	299	0	0	1	0	1	0
ENSP00000386790	WIPF3	483	0	0	1	0	0	0
ENSP00000387593	TANC2	1990	0	0	1	0	0	0
ENSP00000387875	KIF4B	1234	0	0	1	0	0	0
ENSP00000387892	HLA-DQA1	255	0	0	1	0	0	0
ENSP00000388293	TSTD1	115	0	0	2	0	0	0
ENSP00000388486	FOXN2	246	0	0	1	0	0	0
ENSP00000388703	SLC4A7	1051	0	0	1	0	0	0
ENSP00000389943	DPP3	737	0	0	1	0	0	0

ENSP00000389975	SACM1L	287	0	0	1	0	0	0
ENSP00000390734	AMPH	653	0	0	1	0	0	0
ENSP00000391167	DENND4A	1906	0	0	2	0	0	0
ENSP00000391416	SEPT3	178	0	0	2	0	0	0
ENSP00000391501	GNAQ	170	0	0	2	0	0	0
ENSP00000392781	CAPZB	206	0	0	1	0	0	1
ENSP00000392978	OMA1	183	0	0	1	0	0	0
ENSP00000393228	PSD3	376	0	0	1	0	0	0
ENSP00000393525	IMPDH2	470	0	0	1	0	0	0
ENSP00000394018	VPS8	74	0	0	1	0	0	0
ENSP00000394313	SMPD4	615	0	0	1	0	0	0
ENSP00000397078	SMPD4	313	0	0	1	0	0	0
ENSP00000398010	FRY	150	0	0	1	0	0	0
ENSP00000398081	RC3H2	188	0	0	1	0	0	0
ENSP00000398803	TSTA3	321	0	0	1	0	0	0
ENSP00000400057	ASRGL1	308	0	0	1	0	0	0
ENSP00000400285	KCNAB2	254	0	0	1	0	0	0
ENSP00000401070	ARPC2	89	0	0	1	1	1	0
ENSP00000401507	IKZF1	226	0	0	1	0	0	0
ENSP00000402373	ACTA2	377	0	0	1	0	0	0
ENSP00000402618	PSMD2	749	0	0	1	1	0	1
ENSP00000403084	SARDH	918	0	0	1	1	0	1
ENSP00000404304	ARMCX4	2290	0	0	1	0	0	0
ENSP00000404469	STK25	247	0	0	1	0	0	0
ENSP00000405267	AHCYL2	509	0	0	1	0	1	0
ENSP00000405561	CAMK2G	335	0	0	1	0	0	0
ENSP00000406569	GLCCI1	191	0	0	1	0	0	0
ENSP00000407791	CLIC1	241	0	0	1	0	0	0
ENSP00000407944	SEC31A	1233	0	0	1	0	0	0
ENSP00000408711	ARPC1A	279	0	0	1	0	0	0
ENSP00000408789	LZTR1	54	0	0	1	0	0	0
ENSP00000409418	SLC4A7	463	0	0	1	0	0	0
ENSP00000409456	HNRNPK	379	0	0	1	0	0	0
ENSP00000410862	RUNDC3A	446	0	0	1	0	1	0
ENSP00000411189	DDAH1	192	0	0	1	1	2	0
ENSP00000412035	PIP4K2C	373	0	0	1	0	0	0
ENSP00000412044	GABBR1	899	0	0	2	0	0	0
ENSP00000412606	FAM9B	186	0	0	1	0	0	0
ENSP00000412996	CCT7	98	0	0	1	1	0	0
ENSP00000413133	APC	2843	0	0	1	0	0	0
ENSP00000413311	ABHD12	187	0	0	2	0	0	0
ENSP00000414624	PRPSAP1	385	0	0	1	0	0	0
ENSP00000414906	DCTN4	467	0	0	1	0	0	1
ENSP00000415197	TTLL4	179	0	0	1	0	0	0
ENSP00000415549	RPS24	131	0	0	1	0	1	1
ENSP00000415812	TRIM2	744	0	0	1	1	0	0
ENSP00000416591	POMZP3	154	0	0	1	0	0	0
ENSP00000417254	RUVBL1	267	0	0	1	0	0	0
ENSP00000417689	MGLL	89	0	0	1	0	0	0
ENSP00000418309	C3orf17	238	0	0	1	0	0	0
ENSP00000418458	SPATA6L	392	0	0	2	0	0	0
ENSP00000418461	PCMT1	67	0	0	1	1	0	0
ENSP00000418842	NDUFA6	154	0	0	1	0	0	0
ENSP00000418889	CADPS	345	0	0	1	2	1	0
ENSP00000419236	ALCAM	570	0	0	3	0	3	1
ENSP00000419260	PIK3CG	1102	0	0	1	0	0	0
ENSP00000419294	ATP6V1A	221	0	0	2	0	2	2
ENSP00000419800	NFKBIZ	601	0	0	1	0	0	0

ENSP00000420000	CAMKV	123	0	0	1	0	0	0
ENSP00000420139	CAMKV	94	0	0	1	1	1	0
ENSP00000420315	ABHD6	337	0	0	1	1	0	0
ENSP00000420527	ABCA2	2024	0	0	1	0	3	2
ENSP00000420724	CAMKV	427	0	0	0.5	0	0	0
ENSP00000420842	GUCY1B3	586	0	0	2	0	0	0
ENSP00000421460	DDX41	173	0	0	1	0	0	0
ENSP00000421566	XPNPEP1	666	0	0	1	0	1	0
ENSP00000422191	RPL9	166	0	0	0.5	0	0	0
ENSP00000422316	GNB2L1	274	0	0	1	0	0	0
ENSP00000422482	HSPA4L	839	0	0	1	0	0	0
ENSP00000422810	TMEM33	222	0	0	1	0	1	0
ENSP00000422947	TRIM34	488	0	0	1	0	0	0
ENSP00000423440	ARMCX4	348	0	0	1	0	0	0
ENSP00000425715	CCDC149	529	0	0	1	0	2	0
ENSP00000426720	DPYSL3	92	0	0	1	0	1	0
ENSP00000427194	MAP1B	103	0	0	1	0	0	1
ENSP00000428175	TACC1	138	0	0	1	0	0	0
ENSP00000429483	RPL30	114	0	0	1	0	0	0
ENSP00000429661	HNRNPH1	115	0	0	1	0	0	0
ENSP00000429754	SLC20A2	652	0	0	1	0	0	0
ENSP00000430167	MTUS1	1216	0	0	1	0	0	0
ENSP00000430391	LCP2	140	0	0	1	0	0	0
ENSP00000430443	MPP2	126	0	0	1	0	0	0
ENSP00000430517	ANXA6	641	0	0	1	0	1	0
ENSP00000430774	CTD-2410N18.5	91	0	0	1	1	1	0
ENSP00000431042	DCTN4	86	0	0	1	0	0	0
ENSP00000431150	RBM25	843	0	0	1	0	0	0
ENSP00000431587	TSTA3	268	0	0	1	0	0	0
ENSP00000431883	JAM3	355	0	0	1	0	0	1
ENSP00000433089	TTYH2	513	0	0	1	0	0	0
ENSP00000433206	JAM3	124	0	0	1	0	0	0
ENSP00000433598	PPP2R1B	224	0	0	1	0	1	1
ENSP00000434033	RAP1GAP	617	0	0	1	0	0	0
ENSP00000435235	IPO7	130	0	0	1	1	1	0
ENSP00000435735	TMX4	198	0	0	1	0	0	0
ENSP00000436348	EPB41L2	935	0	0	2	1	3.5	0
ENSP00000436799	FAIM3	125	0	0	1	0	0	0
ENSP00000437383	ACTR2	305	0	0	2	0	0	0
ENSP00000437565	ATP5C1	250	0	0	1	0	0	0
ENSP00000437606	COPS3	403	0	0	1	0	0	0
ENSP00000437959	POSTN	781	0	0	1	0	0	0
ENSP00000439042	OAT	301	0	0	1	0	0	0
ENSP00000439211	ZNF611	705	0	0	1	0	0	0
ENSP00000439837	PLEKHA5	1098	0	0	1	0	0	0
ENSP00000439920	SCN3A	483	0	0	1	0	0	0
ENSP00000440564	ITPR1	721	0	0	1	0	0	0
ENSP00000440611	PLEKHA5	874	0	0	1	0	0	0
ENSP00000440761	PDHA1	359	0	0	1	0	0	0
ENSP00000441762	NUP85	610	0	0	1	0	0	0
ENSP00000442427	DLAT	418	0	0	1	0	0	0
ENSP00000442784	CEP112	955	0	0	1	0	0	0
ENSP00000443510	AGPAT3	376	0	0	1	0	0	0
ENSP00000443925	PSMD7	247	0	0	1	0	0	0
ENSP00000444345	XPOT	228	0	0	1	0	1	1
ENSP00000444354	BUB3	248	0	0	1	0	0	0
ENSP00000444423	TCP1	100	0	0	1	0	1	0
ENSP00000444815	FHL1	280	0	0	1	0	1	0

ENSP00000444984	CCT8	529	0	0	1	1	3	0
ENSP00000445006	KPNB1	660	0	0	1	0	1	0
ENSP00000445087	NXN	126	0	0	1	0	3	1
ENSP00000445546	PSMC2	296	0	0	1	0	1	0
ENSP00000446231	GOLT1B	74	0	0	1	1	0	0
ENSP00000446258	VEPH1	788	0	0	1	0	0	0
ENSP00000446439	KIAA0513	401	0	0	1	0	0	0
ENSP00000446569	CCDC65	373	0	0	1	0	0	0
ENSP00000446740	PFKM	41	0	0	1	0	1	0
ENSP00000447472	ERP29	95	0	0	1	0	0	0
ENSP00000447514	MCRS1	318	0	0	1	0	0	0
ENSP00000447534	CORO1C	480	0	0	1	0	0	0
ENSP00000447895	RAB5B	115	0	0	1	0	1	1
ENSP00000448179	ALDH2	79	0	0	1	0	0	0
ENSP00000448415	SCN8A	972	0	0	1	0	0	0
ENSP00000448847	PCBP2	160	0	0	1	0	0	0
ENSP00000449330	CORO1C	181	0	0	1	0	0	0
ENSP00000449970	HLA-DQA1	191	0	0	1	0	0	0
ENSP00000450281	AL021546.6	168	0	0	1	0	0	0
ENSP00000450528	PSMA6	203	0	0	1	0	2	0
ENSP00000450636	WDR20	520	0	0	1	0	0	0
ENSP00000450834	ATP6V1D	44	0	0	1	0	0.5	1
ENSP00000451376	NRG3	526	0	0	1	0	0	0
ENSP00000451576	GNG2	71	0	0	3	0	2	1
ENSP00000451751	C14orf166	68	0	0	1	0	1	0
ENSP00000453792	ARNT2	706	0	0	1	0	0	0
ENSP00000454243	AC114783.1	121	0	0	1	0	0	0
ENSP00000454591	FAM188B2	356	0	0	1	0	0	0
ENSP00000454905	GAS8	117	0	0	1	0	0	0
ENSP00000455066	PMM2	91	0	0	1	0	1	0
ENSP00000455096	AC114783.1	84	0	0	1	0	0	0
ENSP00000455188	RPL7A	266	0	0	1	0	0	0
ENSP00000455635	CNOT1	2371	0	0	1	0	0	0
ENSP00000456060	SH2B2	243	0	0	1	0	0	0
ENSP00000456313	ADAMTSL3	1682	0	0	1	0	0	0
ENSP00000456649	CNOT1	2150	0	0	2	1	0	0
ENSP00000456842	PCBP2	184	0	0	1	0	0	0
ENSP00000457358	DENND4A	1001	0	0	1	0	0	0
ENSP00000457885	MPI	339	0	0	1	0	0	0
ENSP00000458660	PHLPP2	723	0	0	2	0	0	0
ENSP00000459227	PRKCB	119	0	0	1	0	0	0
ENSP00000461168	NLGN2	835	0	0	1	0	0	0
ENSP00000461238	CHMP6	173	0	0	1	0	0	0
ENSP00000461405	SERPINF1	138	0	0	1	0	0	0
ENSP00000461562	NXN	230	0	0	1	0	3	1
ENSP00000461610	JH8	556	0	0	1	0	0	0
ENSP00000462109	TANC2	1254	0	0	1	0	0	0
ENSP00000463067	EIF4A1	117	0	0	1	2	0	0
ENSP00000463481	MRPS23	61	0	0	1	0	0	0
ENSP00000463831	PIP4K2B	71	0	0	1	0	0	0
ENSP00000464434	PSMD11	187	0	0	1	0	1	0
ENSP00000465019	PFN1	104	0	0	1	0	0	2
ENSP00000465259	ATP5A1	144	0	0	1	0	0	1
ENSP00000465570	TTN	34350	0	0	1	0	0	0
ENSP00000466015	ACAA2	394	0	0	1	0	0	0
ENSP00000466651	PRKACA	343	0	0	1	1	1	1
ENSP00000466842	TARDBP	222	0	0	1	0	0	0
ENSP00000466852	ATP6V0A1	191	0	0	1	1	0	0

ENSP00000467012	CHST8	178	0	0	1	0	0	0
ENSP00000467141	TTN	35991	0	0	1	0	0	0
ENSP00000467724	SLC39A6	273	0	0	1	0	0	0
ENSP00000467805	EFTUD2	972	0	0	1	0	1	0
ENSP00000468169	ATP6V0A1	129	0	0	1	0	0	0
ENSP00000468214	RUNDC3A	400	0	0	1	0	0	0
ENSP00000468246	TPM4	170	0	0	1	0	0	0
ENSP00000469457	ENO2	434	0	0	1	0	2	2
ENSP00000469913	HNRNPL	198	0	0	1	0	0	0
ENSP00000469936	TUBB4A	160	0	0	2	0	0	1
ENSP00000470204	SYP	203	0	0	1	0	1	0
ENSP00000471264	PPFIA4	692	0	0	1	1	0	0
ENSP00000471961	CHMP2A	159	0	0	1	0	0	0
ENSP00000472680	CHMP2A	222	0	0	2	0	0	0
ENSP00000473461	NDUFA6	128	0	0	1	0	0	0
ENSP00000474534	HSPE1-MOB4	261	0	0	1	0	1	1
ENSP00000475352	SSBP4	206	0	0	1	0	0	0
ENSP00000476819	RP11-1407O15.2	478	0	0	1	0	1	1
ENSP00000476998	TKT	623	0	0	1	0	2	0
ENSP00000020673	PSD	1024	0	0	0	1	0	0
ENSP00000023897	GABRA1	456	0	0	0	1	0	0
ENSP00000160373	CTTNBP2	1663	0	0	0	1	0	0
ENSP00000205061	GLG1	1203	0	0	0	1	0	0
ENSP00000205214	AASDH	1098	0	0	0	1	0	0
ENSP00000216286	NID2	1375	0	0	0	1	0	0
ENSP00000216445	C14orf105	296	0	0	0	1	0	0
ENSP00000218867	SGCG	291	0	0	0	1	1	0
ENSP00000220876	STMN2	179	0	0	0	1	0	0
ENSP00000221249	PNPLA6	1327	0	0	0	1	0	0
ENSP00000228567	SYT10	523	0	0	0	1	0	0
ENSP00000229135	IFNG	166	0	0	0	1	0	0
ENSP00000229330	HCFC2	792	0	0	0	1	0	0
ENSP00000232603	MORC1	984	0	0	0	1	0	0
ENSP00000233072	CPS1	1500	0	0	0	1	0	0
ENSP00000236671	CTSD	412	0	0	0	1	0	0
ENSP00000244230	MPHOSPH10	681	0	0	0	1	0	0
ENSP00000245206	GOT2	430	0	0	0	1	1	1
ENSP00000247087	AHDC1	1603	0	0	0	1	0	1
ENSP00000249130	SULT4A1	260	0	0	0	1	0	0
ENSP00000252071	ACTR3C	210	0	0	0	1	1	1
ENSP00000252483	PVRL2	538	0	0	0	1	0	0
ENSP00000252485	PVRL2	479	0	0	0	1	0	0
ENSP00000252773	UNC13A	1703	0	0	0	1	0	0
ENSP00000254658	PER2	404	0	0	0	1	0	0
ENSP00000256652	CD101	1021	0	0	0	1	0	0
ENSP00000256707	KIDINS220	1771	0	0	0	1	0	0
ENSP00000257497	ANXA1	346	0	0	0	1	0	0
ENSP00000258960	NMT1	496	0	0	0	1	1	0
ENSP00000259808	RIPK1	671	0	0	0	1	1	0
ENSP00000260359	NUSAP1	426	0	0	0	1	0	0
ENSP00000261509	PALLD	1106	0	0	0	1	0	0
ENSP00000261729	RASAL1	804	0	0	0	2	1	0
ENSP00000262506	CSNK2A2	350	0	0	0	1	0	0
ENSP00000263461	WDR11	1224	0	0	0	1	0	0
ENSP00000263525	TNR	1358	0	0	0	1	0	0
ENSP00000263663	TAF1B	588	0	0	0	1	0	0
ENSP00000263969	MFN1	741	0	0	0	1	0	0
ENSP00000264203	CAPZB	260	0	0	0	1	0	0

ENSP00000264221	PAICS	425	0	0	0	1	0	0
ENSP00000264436	ADD2	726	0	0	0	1	0	0
ENSP00000265447	ANXA11	505	0	0	0	1	0	0
ENSP00000265634	NPTX2	431	0	0	0	2	0	0
ENSP00000265678	RPS6KA2	733	0	0	0	1	0	0
ENSP00000265724	ABCB1	1280	0	0	0	2	0	0
ENSP00000267079	MAP3K12	859	0	0	0	1	0	0
ENSP00000267568	PTGR2	351	0	0	0	1	0	0
ENSP00000267853	MYZAP	466	0	0	0	1	0	0
ENSP00000268852	MYO19	770	0	0	0	1	0	0
ENSP00000269321	ARHGDIA	204	0	0	0	1	1	0
ENSP00000271452	NUF2	464	0	0	0	1	0	0
ENSP00000272371	OTOF	1997	0	0	0	1	0	0
ENSP00000272849	NRP2	906	0	0	0	1	0	0
ENSP00000273859	ATP10D	1426	0	0	0	1	0	0
ENSP00000274026	CCNA2	432	0	0	0	1	0	0
ENSP00000274546	GABRB2	474	0	0	0	1	0	0
ENSP00000275162	CLVS2	327	0	0	0	1	0	0
ENSP00000275764	STRA8	330	0	0	0	1	0	0
ENSP00000280772	ANK3	4377	0	0	0	1	0	0
ENSP00000280886	DIP2C	1556	0	0	0	2	0	0
ENSP00000281828	FARSB	589	0	0	0	1	0	0
ENSP00000283426	PLEKHG4B	1271	0	0	0	1	0	0
ENSP00000284674	GPR26	337	0	0	0	1	0	0
ENSP00000286067	C10orf12	1247	0	0	0	1	0	0
ENSP00000287394	ATAD2	1390	0	0	0	1	0	0
ENSP00000290795	GPBP1L1	474	0	0	0	1	0	0
ENSP00000294309	TPCN2	752	0	0	0	1	1	0
ENSP00000294981	MAPKAPK2	370	0	0	0	1	0	0
ENSP00000297508	MICALL2	904	0	0	0	1	0	0
ENSP00000299045	TCP11L2	519	0	0	0	1	0	0
ENSP00000299441	DCHS1	3298	0	0	0	1	1	0
ENSP00000299927	ZNF592	1267	0	0	0	1	0	0
ENSP00000300862	HIF3A	667	0	0	0	1	0	0
ENSP00000303554	PTPN9	593	0	0	0	1	0	0
ENSP00000304185	GTSF1	167	0	0	0	1	0	0
ENSP00000304592	FASN	2511	0	0	0	1	0	0
ENSP00000304743	CLASP2	995	0	0	0	1	1	0
ENSP00000305027	SOGA2	1905	0	0	0	1	0	0
ENSP00000306606	ADH1B	375	0	0	0	1	0	0
ENSP00000307129	C8orf74	294	0	0	0	1	0	0
ENSP00000307705	HIST1H1E	219	0	0	0	2	0	0
ENSP00000308820	RIC3	369	0	0	0	1	0	1
ENSP00000309548	PDP2	529	0	0	0	1	0	0
ENSP00000311134	RABL6	729	0	0	0	1	0	0
ENSP00000312769	PPIE	301	0	0	0	1	1	0
ENSP00000312860	TOM1L2	462	0	0	0	1	0	0
ENSP00000313214	TMUB2	301	0	0	0	1	0	0
ENSP00000314036	AGPAT4	378	0	0	0	1	0	0
ENSP00000314837	KLC2	622	0	0	0	1	0	0
ENSP00000315328	CELF2	490	0	0	0	1	0	0
ENSP00000316029	TLN1	2541	0	0	0	1	0	0
ENSP00000316809	PITPNA	270	0	0	0	1	0	0
ENSP00000317749	C1orf167	828	0	0	0	1	0	0
ENSP00000318466	WDFY3	3509	0	0	0	1	0	0
ENSP00000319473	PHF8	878	0	0	0	1	0	0
ENSP00000321706	GEMIN4	1058	0	0	0	1	0	0
ENSP00000322706	HMGCS1	520	0	0	0	2	0	1

ENSP00000323557	BRD9	501	0	0	0	1	0	0
ENSP00000324074	ANP32E	268	0	0	0	1	0	0
ENSP00000326804	CUL1	776	0	0	0	1	1	0
ENSP00000329161	SEPT9	568	0	0	0	1	0	0
ENSP00000329419	COPB2	906	0	0	0	1	1	0
ENSP00000330836	GLRX3	335	0	0	0	1	0	0
ENSP00000331817	ALYREF	257	0	0	0	1	0	0
ENSP00000332816	PTK2B	1009	0	0	0	1	0	0
ENSP00000334156	SPTBN1	2155	0	0	0	4	6	1
ENSP00000334910	PDE2A	941	0	0	0	1	2	0
ENSP00000335592	GABRA5	462	0	0	0	1	0	0
ENSP00000335632	CHP1	195	0	0	0	1	1	0
ENSP00000336127	TTC7B	843	0	0	0	1	0	0
ENSP00000340051	PHF8	948	0	0	0	1	0	0
ENSP00000340167	OLA1	238	0	0	0	1	0	0
ENSP00000340210	CD59	128	0	0	0	1	0	1
ENSP00000340328	NYX	481	0	0	0	1	0	0
ENSP00000340688	LPHN1	1474	0	0	0	1	1	0
ENSP00000340888	GSN	731	0	0	0	1	0	0
ENSP00000340889	PRSS3	261	0	0	0	3	0	0
ENSP00000343021	ALKBH2	261	0	0	0	1	0	0
ENSP00000343087	CCDC157	752	0	0	0	1	0	0
ENSP00000343147	PPP3CB	496	0	0	0	1	0	0
ENSP00000343966	PSPC1	523	0	0	0	1	0	1
ENSP00000344055	AP3D1	1153	0	0	0	1	0	0
ENSP00000344129	ZNF648	568	0	0	0	1	0	0
ENSP00000344658	SNCG	126	0	0	0	1	0	0
ENSP00000345137	OTOF	1230	0	0	0	1	0	0
ENSP00000345923	BCOR	1721	0	0	0	1	0	0
ENSP00000346139	TBC1D20	403	0	0	0	1	0	0
ENSP00000346791	TTC3	2025	0	0	0	1	0	0
ENSP00000347224	GPBP1L1	474	0	0	0	1	0	0
ENSP00000347380	SYNCRIP	562	0	0	0	1	2	1
ENSP00000347416	AP3D1	1215	0	0	0	1	0	0
ENSP00000347866	PHACTR3	518	0	0	0	1	0	0
ENSP00000348877	GPI	558	0	0	0	1	0	0
ENSP00000349259	SPTBN1	2364	0	0	0	4	6	2
ENSP00000349420	GPM6B	265	0	0	0	1	0	1
ENSP00000349564	TTC7B	860	0	0	0	1	0	0
ENSP00000349594	ELAVL4	383	0	0	0	1	0	0
ENSP00000349887	ASNA1	348	0	0	0	1	0	0
ENSP00000350012	ACSL3	720	0	0	0	1	2	0
ENSP00000351047	ICA1L	482	0	0	0	1	0	0
ENSP00000352219	BCS1L	419	0	0	0	1	1	0
ENSP00000353002	PHACTR3	556	0	0	0	1	0	0
ENSP00000353114	AHNAK2	5795	0	0	0	1	0	0
ENSP00000353129	KCTD8	473	0	0	0	1	0	0
ENSP00000353397	TP53TG3C	102	0	0	0	1	0	0
ENSP00000353408	MSN	577	0	0	0	1	0	0
ENSP00000353467	FLNA	2639	0	0	0	1	0	1
ENSP00000354109	RGS22	1264	0	0	0	2	0	0
ENSP00000354335	MPEG1	716	0	0	0	1	0	0
ENSP00000354481	BRINP2	783	0	0	0	1	0	0
ENSP00000354536	ASTN1	1294	0	0	0	1	0	0
ENSP00000354597	DENND4B	1496	0	0	0	1	0	0
ENSP00000355013	CTR9	1173	0	0	0	1	0	0
ENSP00000355124	KRT19	400	0	0	0	1	0	0
ENSP00000355348	NCKAP1	1128	0	0	0	1	0	1

ENSP00000355513	KMO	182	0	0	0	1	0	0
ENSP00000355517	KMO	486	0	0	0	1	0	0
ENSP00000355523	RGS7	487	0	0	0	1	0	0
ENSP00000355872	AGPAT4	159	0	0	0	1	0	0
ENSP00000355890	EPRS	1512	0	0	0	1	1	2
ENSP00000356097	FAM72A	149	0	0	0	1	0	0
ENSP00000356211	TMEM183A	376	0	0	0	2	0	1
ENSP00000356459	ADGB	112	0	0	0	1	0	0
ENSP00000356918	STX7	261	0	0	0	1	0	0
ENSP00000357048	COPA	1233	0	0	0	2	0	0
ENSP00000357571	TTC40	1530	0	0	0	1	0	0
ENSP00000357633	GLRX3	335	0	0	0	1	0	0
ENSP00000357635	DENND4B	1218	0	0	0	1	0	0
ENSP00000358060	TARS2	718	0	0	0	1	0	0
ENSP00000358115	ANP32E	220	0	0	0	1	0	0
ENSP00000358400	MDN1	5596	0	0	0	1	0	0
ENSP00000358482	CD101	1021	0	0	0	1	0	0
ENSP00000358633	G6PD	561	0	0	0	2	0	0
ENSP00000358695	CYB5R4	521	0	0	0	0.5	0	0
ENSP00000358696	PLXNA3	1871	0	0	0	1	0	0
ENSP00000358842	GSTM2	179	0	0	0	1	1	0
ENSP00000358865	INA	499	0	0	0	1	0	0
ENSP00000359184	AGL	1532	0	0	0	1	0	0
ENSP00000360312	BSND	320	0	0	0	1	0	0
ENSP00000360718	RAB3B	219	0	0	0	2	0	0
ENSP00000360740	RABL6	614	0	0	0	1	0	0
ENSP00000360886	ELAVL4	385	0	0	0	1	0	0
ENSP00000361305	ANXA11	505	0	0	0	1	0	0
ENSP00000361464	DLG5	1579	0	0	0	1	0	0
ENSP00000362016	MACF1	4434	0	0	0	1	1	0
ENSP00000362327	PPA1	178	0	0	0	1	0	0
ENSP00000362671	RPL35	96	0	0	0	1	0	1
ENSP00000362912	GSN	207	0	0	0	1	0	0
ENSP00000363123	AHDC1	1603	0	0	0	1	0	1
ENSP00000364590	CUL4A	659	0	0	0	1	0	0
ENSP00000365281	BIN1	424	0	0	0	1	0	0
ENSP00000365529	GPR158	1215	0	0	0	1	0	0
ENSP00000365633	PDE2A	685	0	0	0	1	2	0
ENSP00000367465	NYX	481	0	0	0	1	0	0
ENSP00000367716	BCOR	1703	0	0	0	1	0	0
ENSP00000367727	PANK4	773	0	0	0	1	0	0
ENSP00000368460	PDK3	406	0	0	0	1	0	0
ENSP00000368790	MTRF1	445	0	0	0	1	0	0
ENSP00000369769	HSPH1	817	0	0	0	2	1	1
ENSP00000369773	RIPK1	671	0	0	0	1	0	0
ENSP00000369942	GCOM1	445	0	0	0	1	0	0
ENSP00000370194	GMDS	372	0	0	0	1	0	0
ENSP00000370907	DIP2C	656	0	0	0	1	0	0
ENSP00000371267	YWHAQ	245	0	0	0	2	0	0
ENSP00000371294	CTNS	400	0	0	0	1	1	0
ENSP00000371310	USP7	1086	0	0	0	1	0	0
ENSP00000372025	MBP	197	0	0	0	1	0	0
ENSP00000373215	CADPS	1353	0	0	0	2	0	1
ENSP00000373637	SMYD3	428	0	0	0	1	0	0
ENSP00000374183	CAMSAP1	1602	0	0	0	1	0	0
ENSP00000374592	ZC3H12D	527	0	0	0	1	0	0
ENSP00000375712	SMYD3	147	0	0	0	1	0	0
ENSP00000376792	VTI1A	217	0	0	0	1	0	0

ENSP00000377480	MAGEA3	314	0	0	0	1	0	0
ENSP00000377531	GABRB2	512	0	0	0	1	0	0
ENSP00000377641	KLC2	333	0	0	0	1	0	0
ENSP00000378306	PPP3CB	525	0	0	0	1	0	0
ENSP00000378887	DHRS7B	325	0	0	0	1	0	0
ENSP00000380602	CNTN4	1026	0	0	0	1	0	0
ENSP00000380878	PPP3CC	521	0	0	0	1	0	0
ENSP00000381036	ADGB	1667	0	0	0	1	0	0
ENSP00000381225	CBS	551	0	0	0	1	0	0
ENSP00000381339	GNAT3	354	0	0	0	1	0	0
ENSP00000382633	STMN1	149	0	0	0	1	0	0
ENSP00000384634	EIF3L	466	0	0	0	1	1	1
ENSP00000384892	RIMS2	1349	0	0	0	1	0	1
ENSP00000385357	CCDC157	752	0	0	0	1	0	0
ENSP00000385589	SCARF2	865	0	0	0	1	0	0
ENSP00000386104	CPE	476	0	0	0	1	0	0
ENSP00000386616	ZC3H12D	527	0	0	0	1	0	0
ENSP00000387255	XIRP2	3327	0	0	0	1	0	1
ENSP00000387382	ICA1L	119	0	0	0	1	0	0
ENSP00000388530	CELF2	490	0	0	0	1	0	0
ENSP00000389062	CLYBL	89	0	0	0	1	0	1
ENSP00000390590	DDN	711	0	0	0	1	0	0
ENSP00000391007	BCS1L	76	0	0	0	1	1	0
ENSP00000391705	MAPRE3	218	0	0	0	1	0	2
ENSP00000392061	DNAH14	4515	0	0	0	1	0	0
ENSP00000392541	ALB	197	0	0	0	1	0	0
ENSP00000392683	FLOT1	192	0	0	0	1	0	0
ENSP00000393017	FAM200B	657	0	0	0	0.5	0	0.5
ENSP00000393379	KIF5C	957	0	0	0	1	0	0
ENSP00000393810	HBD	104	0	0	0	1	1	0
ENSP00000393822	EIF3L	183	0	0	0	1	1	0
ENSP00000394100	GOT2	387	0	0	0	2	1	1
ENSP00000394504	MDH1	119	0	0	0	1	0	0
ENSP00000394690	G6PD	256	0	0	0	1	0	0
ENSP00000394770	CMYA5	4069	0	0	0	1	0	0
ENSP00000394993	GGT7	202	0	0	0	1	2	2
ENSP00000395041	ASTN1	1216	0	0	0	1	0	0
ENSP00000396376	PPP1R7	282	0	0	0	1	0	0
ENSP00000397782	SYNCRIP	185	0	0	0	1	2	1
ENSP00000398015	LDHB	136	0	0	0	2	0	2
ENSP00000399273	ETFA	284	0	0	0	1	0	0
ENSP00000399729	OGT	156	0	0	0	1	0	0
ENSP00000399771	RUFY3	506	0	0	0	1	0	0
ENSP00000400031	MTRF1	468	0	0	0	1	0	0
ENSP00000401014	NUSAP1	378	0	0	0	1	0	0
ENSP00000401249	PRSS3	177	0	0	0	1	0	0
ENSP00000403793	CYFIP2	952	0	0	0	1	1	0
ENSP00000403943	TTC3	1179	0	0	0	1	0	0
ENSP00000404590	TMOD2	315	0	0	0	1	0	0
ENSP00000404618	ICA1L	119	0	0	0	1	0	0
ENSP00000407466	RBM39	39	0	0	0	1	0	0
ENSP00000408456	TTC3	780	0	0	0	1	0	0
ENSP00000408759	KRT19	196	0	0	0	1	0	0
ENSP00000409472	DNAH14	3507	0	0	0	1	0	0
ENSP00000410083	VEZT	779	0	0	0	1	0	0
ENSP00000411465	CTNS	292	0	0	0	1	0	0
ENSP00000412357	ADD2	226	0	0	0	1	0	0
ENSP00000412707	SYNJ1	178	0	0	0	1	0	1

ENSP00000413999	CKMT1B	241	0	0	0	1	0	1
ENSP00000414132	ALDH7A1	502	0	0	0	1	0	0
ENSP00000414220	SULT4A1	75	0	0	0	1	0	0
ENSP00000415441	GABRA1	456	0	0	0	1	0	0
ENSP00000415537	SARDH	750	0	0	0	1	0	0
ENSP00000415802	DDX39B	325	0	0	0	1	0	0
ENSP00000415840	RP11-295K3.1	379	0	0	0	1	0	0
ENSP00000416892	EIF3L	564	0	0	0	1	2	1
ENSP00000417282	MORC1	963	0	0	0	1	0	0
ENSP00000418448	PDHB	350	0	0	0	1	0	0
ENSP00000418994	SYNPR	285	0	0	0	1	0	0
ENSP00000419845	BRD9	544	0	0	0	1	0	0
ENSP00000419974	CLASP2	1514	0	0	0	1	0	0
ENSP00000420174	ERVFRD-1	538	0	0	0	1	0	0
ENSP00000420254	ARF4	127	0	0	0	1	1	0
ENSP00000420617	MFN1	741	0	0	0	1	0	0
ENSP00000421096	PAICS	425	0	0	0	1	0	0
ENSP00000422524	NFASC	486	0	0	0	1	0	0
ENSP00000423799	ANK2	1114	0	0	0	1	0	0
ENSP00000424341	CANX	144	0	0	0	1	1	0
ENSP00000425217	GRIA2	124	0	0	0	1	0	0
ENSP00000425236	ANK3	1868	0	0	0	1	0	0
ENSP00000425932	PANK4	473	0	0	0	1	0	0
ENSP00000426591	ANXA3	55	0	0	0	1	0	0
ENSP00000428837	PTK2B	194	0	0	0	1	0	0
ENSP00000429382	RGS22	1083	0	0	0	2	0	0
ENSP00000429562	UNC13A	1703	0	0	0	1	0	0
ENSP00000430058	YWHAZ	246	0	0	0	1	0	0
ENSP00000430572	ANXA6	129	0	0	0	1	0	0
ENSP00000431675	RABL6	471	0	0	0	1	0	0
ENSP00000432280	TBC1D20	403	0	0	0	1	0	0
ENSP00000433475	PPIE	213	0	0	0	1	1	0
ENSP00000434003	C14orf105	295	0	0	0	1	0	0
ENSP00000434481	CCT3	144	0	0	0	1	0	0
ENSP00000435022	INSC	520	0	0	0	1	0	0
ENSP00000438090	PRKCG	548	0	0	0	1	0	0
ENSP00000438488	CCT6A	500	0	0	0	1	0	0
ENSP00000438604	DDAH1	285	0	0	0	1	0	0
ENSP00000438691	SYT10	342	0	0	0	1	0	0
ENSP00000438733	ACO1	790	0	0	0	1	0	1
ENSP00000439332	CBS	463	0	0	0	1	0	0
ENSP00000439381	CLGN	610	0	0	0	1	0	0
ENSP00000441848	KCNJ2	427	0	0	0	1	0	0
ENSP00000442232	SGCG	291	0	0	0	1	1	0
ENSP00000442942	HCFC2	412	0	0	0	1	0	0
ENSP00000442981	TLN1	2429	0	0	0	1	0	0
ENSP00000443009	RAP1A	184	0	0	0	1	1	0
ENSP00000443235	RNF10	124	0	0	0	1	0	0
ENSP00000443871	RIC3	320	0	0	0	1	0	1
ENSP00000444095	ABCB1	1216	0	0	0	2	0	0
ENSP00000446485	GTSF1	167	0	0	0	1	0	0
ENSP00000446500	HLA-DOA	61	0	0	0	1	0	0
ENSP00000447257	DQ-A1	254	0	0	0	1	0	0
ENSP00000447278	KRT18	391	0	0	0	1	0	0
ENSP00000447907	DYNLL1	89	0	0	0	1	0	1
ENSP00000447970	HLA-DQA1	253	0	0	0	1	0	0
ENSP00000448042	PIP4K2C	171	0	0	0	2	1	0
ENSP00000448629	TCP11L2	234	0	0	0	1	0	0

ENSP00000449038	MAP3K12	892	0	0	0	1	0	0
ENSP00000449285	AL662890.2	42	0	0	0	1	0	0
ENSP00000449395	HLA-DOA	61	0	0	0	1	0	0
ENSP00000450215	HSP90B1	147	0	0	0	1	0	0
ENSP00000450425	CALM1	76	0	0	0	1	0	0
ENSP00000450480	ATP10A	594	0	0	0	1	0	0
ENSP00000450625	ACTN1	207	0	0	0	1	0	0
ENSP00000451752	SPTB	2328	0	0	0	1	0	1
ENSP00000452777	ETFA	289	0	0	0	1	0	0
ENSP00000452964	CCDC175	828	0	0	0	1	0	0
ENSP00000453017	ETFA	239	0	0	0	1	0	0
ENSP00000453403	NUSAP1	441	0	0	0	1	0	0
ENSP00000453483	CPNE6	105	0	0	0	1	0	0
ENSP00000454096	ANXA2	175	0	0	0	1	0	0
ENSP00000454199	CYFIP1	1253	0	0	0	1.5	0	0
ENSP00000454218	GLG1	698	0	0	0	1	0	0
ENSP00000455773	PLEC	4525	0	0	0	1	0	0
ENSP00000458300	P4HB	148	0	0	0	2	0	0
ENSP00000458570	PCDHGA4	931	0	0	0	1	0	0
ENSP00000462387	MAPKAPK2	400	0	0	0	1	0	0
ENSP00000462844	HYOU1	224	0	0	0	1	1	0
ENSP00000462960	ARHGDIA	193	0	0	0	1	1	0
ENSP00000465871	ZNF585B	137	0	0	0	1	0	0
ENSP00000465939	MAPRE2	134	0	0	0	1	0	0
ENSP00000466971	TMUB2	321	0	0	0	1	0	0
ENSP00000468424	NMT1	496	0	0	0	1	1	0
ENSP00000468488	MYO19	186	0	0	0	1	0	0
ENSP00000468585	DHRS7B	310	0	0	0	1	0	0
ENSP00000468668	SEPT9	422	0	0	0	1	0	0
ENSP00000468882	HNRNPH2	449	0	0	0	1	0	0
ENSP00000469988	G6PD	561	0	0	0	1	0	0
ENSP00000470631	PLXNA3	1871	0	0	0	1	0	0
ENSP00000470900	GSPT2	628	0	0	0	0.5	0	0
ENSP00000471208	G6PD	515	0	0	0	2	0	0
ENSP00000471265	EEF2	85	0	0	0	2	0	0
ENSP00000471378	ZNF414	120	0	0	0	1	0	0
ENSP00000472141	CALM3	149	0	0	0	1	1	0
ENSP00000472231	RPS16	129	0	0	0	1	1	0
ENSP00000472251	PPFIA4	191	0	0	0	1	0	0
ENSP00000473093	MAGEA3	314	0	0	0	1	0	0
ENSP00000473211	PNPLA6	1365	0	0	0	1	0	0
ENSP00000473512	HSPH1	115	0	0	0	1	1	0
ENSP00000473564	AASDH	789	0	0	0	1	0	0
ENSP00000476547	CSNK2A1	74	0	0	0	1	0	0
ENSP00000476596	CLCN5	64	0	0	0	1	0	0
ENSP00000007735	KRT33A	404	0	0	0	0	3	4
ENSP00000040738	BOD1L1	3051	0	0	0	0	1	0
ENSP00000080059	HDAC7	991	0	0	0	0	1	0
ENSP00000083182	APPBP2	585	0	0	0	0	1	0
ENSP00000165698	REEP1	201	0	0	0	0	1	0
ENSP00000173898	TRO	1431	0	0	0	0	1	0
ENSP00000215885	PLA2G3	509	0	0	0	0	2	0
ENSP00000216554	EIF5	431	0	0	0	0	1	0
ENSP00000216877	PTPRA	793	0	0	0	0	1	0
ENSP00000217244	CSNK2A1	391	0	0	0	0	1	2
ENSP00000224756	CCSER2	834	0	0	0	0	1	0
ENSP00000225831	CCL2	99	0	0	0	0	1	0
ENSP00000228740	LTA4H	611	0	0	0	0	2	0

ENSP00000233084	DDX1	740	0	0	0	0	2	0
ENSP00000233242	APOB	4563	0	0	0	0	1	0
ENSP00000236698	STAG1	1221	0	0	0	0	1	0
ENSP00000239882	ELF1	619	0	0	0	0	2	0
ENSP00000246639	KRT35	425	0	0	0	0	3	0
ENSP00000248846	TUBGCP6	1819	0	0	0	0	1	1
ENSP00000251076	DMXL2	3036	0	0	0	0	4	2
ENSP00000251091	SAMD4A	630	0	0	0	0	1	0
ENSP00000251472	MAST1	1570	0	0	0	0	1	0
ENSP00000251645	KRT31	416	0	0	0	0	3	3
ENSP00000251646	KRT33B	404	0	0	0	0	1	0
ENSP00000251722	USP40	1235	0	0	0	0	1	0
ENSP00000252594	NSUN5	429	0	0	0	0	1	0
ENSP00000253815	UBE2D2	118	0	0	0	0	1	0
ENSP00000254029	WDR44	913	0	0	0	0	1	0
ENSP00000255305	XPO4	1151	0	0	0	0	1	1
ENSP00000256433	IER3IP1	82	0	0	0	0	1	1
ENSP00000256578	AMPD2	879	0	0	0	0	1	0
ENSP00000257863	AMHR2	573	0	0	0	0	1	0
ENSP00000257901	KRT85	507	0	0	0	0	13	8
ENSP00000257974	KRT82	513	0	0	0	0	4	0
ENSP00000258106	EMX1	290	0	0	0	0	1	0
ENSP00000258187	NDRG4	371	0	0	0	0	1	1
ENSP00000258214	CCDC102A	550	0	0	0	0	1	0
ENSP00000258729	IGF2BP3	579	0	0	0	0	0.5	0
ENSP00000258770	TBRG4	631	0	0	0	0	1	0
ENSP00000260665	LRPPRC	1394	0	0	0	0	1	0
ENSP00000260988	CRYGB	175	0	0	0	0	1	0
ENSP00000261443	CSDE1	767	0	0	0	0	1	0
ENSP00000261588	KIAA0556	1618	0	0	0	0	1	0
ENSP00000261643	COX10	443	0	0	0	0	1	0
ENSP00000261646	SREBF1	1147	0	0	0	0	1	0
ENSP00000261716	TAOK1	1001	0	0	0	0	1	0
ENSP00000262027	MARS	900	0	0	0	0	2	0
ENSP00000262346	ANKRD13C	506	0	0	0	0	1	2
ENSP00000262445	MAP2K4	399	0	0	0	0	1	0
ENSP00000262577	ZC3H3	948	0	0	0	0	1	0
ENSP00000262605	TTPAL	342	0	0	0	0	1	0
ENSP00000262650	ITCH	903	0	0	0	0	1	0
ENSP00000262710	ACIN1	1341	0	0	0	0	1	0
ENSP00000262825	CSF2RB	903	0	0	0	0	1	0
ENSP00000262961	ZFR2	939	0	0	0	0	1	0
ENSP00000262963	PTPRS	1928	0	0	0	0	1	0
ENSP00000263269	GRIN2D	1336	0	0	0	0	1	0
ENSP00000263367	HNRNPUL1	767	0	0	0	0	1	0
ENSP00000263697	DNAJC8	253	0	0	0	0	1	1
ENSP00000263773	FNBP4	1017	0	0	0	0	1	0
ENSP00000263800	LTK	864	0	0	0	0	1	0
ENSP00000264028	ARCN1	511	0	0	0	0	1	0
ENSP00000264639	PSMD3	534	0	0	0	0	1	0
ENSP00000264832	ICAM1	532	0	0	0	0	1	0
ENSP00000264896	SCARB2	478	0	0	0	0	1	0
ENSP00000265245	LSG1	658	0	0	0	0	1	0
ENSP00000265388	TNPO3	923	0	0	0	0	1	0
ENSP00000265395	HIBADH	336	0	0	0	0	1	0
ENSP00000265529	KIF9	790	0	0	0	0	1	0
ENSP00000265602	AHI1	1196	0	0	0	0	1	0
ENSP00000266679	CPSF6	588	0	0	0	0	1	1

ENSP00000267119	KRT71	523	0	0	0	0	1	0
ENSP00000267803	DUOXA1	483	0	0	0	0	1	0
ENSP00000267884	SRP14	136	0	0	0	0	1	1
ENSP00000268057	BBS4	519	0	0	0	0	1	0
ENSP00000268082	CCDC33	367	0	0	0	0	1	0
ENSP00000269500	FBXO15	434	0	0	0	0	1	0
ENSP00000270747	ARHGEF19	802	0	0	0	0	1	0
ENSP00000271643	ADAMTSL4	1074	0	0	0	0	1	0
ENSP00000272317	RPS27A	156	0	0	0	0	2	1
ENSP00000273450	ALDH1L1	912	0	0	0	0	1	0
ENSP00000280333	DOCK1	1865	0	0	0	0	1	0
ENSP00000281268	MUC15	311	0	0	0	0	1	0
ENSP00000281416	MFSD6	791	0	0	0	0	1	0
ENSP00000281844	RABGAP1L	378	0	0	0	0	1	0
ENSP00000286621	ADK	362	0	0	0	0	1	0
ENSP00000288602	BRAF	766	0	0	0	0	1	0
ENSP00000288828	WIPI2	454	0	0	0	0	1	0
ENSP00000289734	ANK1	1880	0	0	0	0	1	0
ENSP00000289893	MACF1	5938	0	0	0	0	1	0
ENSP00000290100	EPB41	775	0	0	0	0	1	0
ENSP00000290607	STARD9	4700	0	0	0	0	1	1
ENSP00000291552	U2AF1	240	0	0	0	0	1	0
ENSP00000292432	HK3	923	0	0	0	0	1	0
ENSP00000293525	KRT86	486	0	0	0	0	20	10
ENSP00000293670	KRT83	493	0	0	0	0	6	3
ENSP00000295920	GMPS	594	0	0	0	0	2	0
ENSP00000296701	FBXO38	943	0	0	0	0	1	0
ENSP00000296862	GPR111	708	0	0	0	0	1	0
ENSP00000296953	CREBRF	639	0	0	0	0	1	0
ENSP00000297142	NEUROD6	337	0	0	0	0	1	0
ENSP00000297261	SHH	462	0	0	0	0	1	0
ENSP00000297591	KIAA1429	1812	0	0	0	0	1	0
ENSP00000297875	SYTL5	730	0	0	0	0	1	0
ENSP00000298249	REEP3	240	0	0	0	0	1	0
ENSP00000298912	CLMN	1002	0	0	0	0	1	0
ENSP00000298919	PDZRN4	776	0	0	0	0	1	0
ENSP00000299980	AP1G1	822	0	0	0	0	1	0
ENSP00000300131	NAB2	525	0	0	0	0	1	0
ENSP00000300456	SLC27A4	643	0	0	0	0	1	0
ENSP00000301764	DDB1	1140	0	0	0	0	2	1
ENSP00000302895	ARAP2	1704	0	0	0	0	1	0
ENSP00000303192	RGL1	803	0	0	0	0	1	0
ENSP00000303315	JUNB	347	0	0	0	0	1	0
ENSP00000303507	BCR	1271	0	0	0	0	1	0
ENSP00000303908	TWF2	349	0	0	0	0	1	0
ENSP00000304350	PRPF8	2335	0	0	0	0	1	0
ENSP00000304355	CCDC126	140	0	0	0	0	1	0
ENSP00000304769	ZNF467	595	0	0	0	0	1	0
ENSP00000306299	FAM117B	345	0	0	0	0	1	0
ENSP00000307078	KIF5B	963	0	0	0	0	2	1.5
ENSP00000310585	PCP2	136	0	0	0	0	1	0
ENSP00000311847	CARHSP1	147	0	0	0	0	1	1
ENSP00000312734	RAB8B	207	0	0	0	0	1	0
ENSP00000313995	KLHDC3	382	0	0	0	0	1	0
ENSP00000314075	AKAP1	593	0	0	0	0	1	0
ENSP00000314787	ARHGEF2	985	0	0	0	0	1	1
ENSP00000315325	ARHGEF2	958	0	0	0	0	1	1
ENSP00000316042	HNRNPA0	305	0	0	0	0	1	1

ENSP00000316460	FYB	783	0	0	0	0	1	0
ENSP00000317606	PSAT1	324	0	0	0	0	1	2
ENSP00000318016	KIAA0196	1159	0	0	0	0	1	0
ENSP00000321116	PNPLA1	446	0	0	0	0	1	0
ENSP00000321729	OR51V1	321	0	0	0	0	1	0
ENSP00000321845	SEC24C	1094	0	0	0	0	1	0
ENSP00000321987	TSPEAR	669	0	0	0	0	1	0
ENSP00000322478	AHI1	1053	0	0	0	0	1	0
ENSP00000322609	C1orf173	1530	0	0	0	0	1	0
ENSP00000323377	EXOC3	745	0	0	0	0	1	0
ENSP00000324578	ETS1	441	0	0	0	0	1	0
ENSP00000324740	YES1	543	0	0	0	0	1	0
ENSP00000325240	LASP1	261	0	0	0	0	1	0
ENSP00000326199	INADL	1582	0	0	0	0	1	0
ENSP00000327349	TMCC1	474	0	0	0	0	1	0
ENSP00000327732	C7orf61	206	0	0	0	0	1	0
ENSP00000328800	WBP2NL	309	0	0	0	0	0.5	0
ENSP00000329165	KRT36	467	0	0	0	0	2	2
ENSP00000329896	FAM120C	895	0	0	0	0	1	0
ENSP00000330696	P2RX7	425	0	0	0	0	1	0
ENSP00000330720	KRTAP11-1	163	0	0	0	0	1	0
ENSP00000331019	RPS27L	84	0	0	0	0	1	0
ENSP00000333229	APOL4	347	0	0	0	0	1	0
ENSP00000333504	HNRNPA1	307	0	0	0	0	1	0
ENSP00000334767	CCDC84	332	0	0	0	0	1	0
ENSP00000334879	SOWAHB	793	0	0	0	0	1	0
ENSP00000335557	SLX4IP	408	0	0	0	0	1	0
ENSP00000336719	ZNF226	803	0	0	0	0	1	0
ENSP00000337168	EPB41L1	881	0	0	0	0	1	0
ENSP00000337446	STARD3	445	0	0	0	0	2	0
ENSP00000338260	APOL4	350	0	0	0	0	1	0
ENSP00000338477	HNRNPF	415	0	0	0	0	1	0
ENSP00000338481	EPB41L2	1005	0	0	0	0	1	1
ENSP00000339913	CXCL12	89	0	0	0	0	1	0
ENSP00000340079	RAB28	204	0	0	0	0	0.5	0
ENSP00000340689	GLS	598	0	0	0	0	1	0
ENSP00000340797	TRIM3	744	0	0	0	0	1	0
ENSP00000341285	HNRNPA1L2	320	0	0	0	0	1	0
ENSP00000341730	RPL10	214	0	0	0	0	1	0
ENSP00000342665	WWP1	704	0	0	0	0	1	0
ENSP00000343041	METTL17	456	0	0	0	0	1	1
ENSP00000343446	MASTL	840	0	0	0	0	1	0
ENSP00000343646	RBL1	1014	0	0	0	0	1	0
ENSP00000344832	CAP1	474	0	0	0	0	1	0
ENSP00000345156	RPL14	215	0	0	0	0	1	0
ENSP00000345259	EPB41	864	0	0	0	0	1	0
ENSP00000345530	NEDD4	1247	0	0	0	0	1	0
ENSP00000345822	SREBF1	1071	0	0	0	0	1	0
ENSP00000346265	SYT5	386	0	0	0	0	1	3
ENSP00000346294	S100A4	101	0	0	0	0	1	0
ENSP00000346516	KMT2A	3931	0	0	0	0	1	0
ENSP00000347293	LTK	803	0	0	0	0	1	0
ENSP00000347648	ZNF512	567	0	0	0	0	1	0
ENSP00000347767	NEK5	708	0	0	0	0	1	0
ENSP00000347792	SYNJ2	1496	0	0	0	0	1	2
ENSP00000348349	MYO9A	2548	0	0	0	0	2	0
ENSP00000349545	OSBPL1A	568	0	0	0	0	1	0
ENSP00000349863	SRPK2	688	0	0	0	0	1	1

ENSP00000350219	IPO5	1097	0	0	0	0	1	1
ENSP00000350657	SYTL5	730	0	0	0	0	1	0
ENSP00000351329	CSDE1	798	0	0	0	0	1	0
ENSP00000351524	HELZ	1942	0	0	0	0	1	0
ENSP00000351539	ZBTB18	531	0	0	0	0	1	0
ENSP00000352535	BCR	1227	0	0	0	0	1	0
ENSP00000354125	EIF3B	814	0	0	0	0	2	0
ENSP00000354271	STRBP	658	0	0	0	0	1	0
ENSP00000354482	SP8	490	0	0	0	0	1	0
ENSP00000354837	ARHGEF2	986	0	0	0	0	1	1
ENSP00000354957	SMC5	1101	0	0	0	0	1	0
ENSP00000355185	DCLRE1A	1040	0	0	0	0	1	0
ENSP00000355518	FH	510	0	0	0	0	1	0
ENSP00000355568	IRF2BP2	587	0	0	0	0	1	0
ENSP00000356071	SERAC1	654	0	0	0	0	1	0
ENSP00000356661	RABGAP1L	253	0	0	0	0	1	0
ENSP00000357075	TAGLN2	199	0	0	0	0	1	0
ENSP00000357077	TAGLN2	199	0	0	0	0	1	0
ENSP00000357219	TTC24	582	0	0	0	0	1	0
ENSP00000357220	TTC24	582	0	0	0	0	1	0
ENSP00000357703	S100A4	101	0	0	0	0	1	0
ENSP00000358079	INPP5F	375	0	0	0	0	1	0
ENSP00000358777	ATP6AP1	470	0	0	0	0	1	0
ENSP00000358810	STRIP1	837	0	0	0	0	1	0
ENSP00000358811	STRIP1	742	0	0	0	0	1	0
ENSP00000358844	GSTM2	193	0	0	0	0	1	0
ENSP00000360021	GNG12	72	0	0	0	0	1	0
ENSP00000360200	INADL	1801	0	0	0	0	1	0
ENSP00000360776	B4GALT5	388	0	0	0	0	1	0
ENSP00000360887	WDR44	824	0	0	0	0	1	0
ENSP00000361281	BEST4	473	0	0	0	0	1	0
ENSP00000361408	ZCCHC24	228	0	0	0	0	1	1
ENSP00000361496	PRPS1	168	0	0	0	0	1	0
ENSP00000361819	ADK	345	0	0	0	0	1	0
ENSP00000361878	CAP1	475	0	0	0	0	1	1
ENSP00000362333	SAR1A	121	0	0	0	0	1	0
ENSP00000362336	SAR1A	50	0	0	0	0	1	0
ENSP00000362931	SRPK1	655	0	0	0	0	1	1
ENSP00000363551	CXCL12	93	0	0	0	0	1	0
ENSP00000364162	TRO	706	0	0	0	0	1	0
ENSP00000364517	IQSEC2	1478	0	0	0	0	1	0
ENSP00000364649	SDHB	280	0	0	0	0	1	0
ENSP00000364801	HSPA1B	641	0	0	0	0	3	0
ENSP00000365309	ABI1	476	0	0	0	0	1	0
ENSP00000365634	KIAA1217	1465	0	0	0	0	1	0
ENSP00000366783	HECTD4	4246	0	0	0	0	1	0
ENSP00000366857	DZANK1	247	0	0	0	0	1	0
ENSP00000367001	DPP6	865	0	0	0	0	1	0
ENSP00000367460	HNRNPUL1	752	0	0	0	0	1	0
ENSP00000367894	PRRG1	218	0	0	0	0	1	0
ENSP00000368646	PRDX4	271	0	0	0	0	1	0
ENSP00000369117	AMHR2	478	0	0	0	0	1	0
ENSP00000369136	NWD1	1432	0	0	0	0	1	0
ENSP00000369349	KRT81	505	0	0	0	0	2	1
ENSP00000369456	ITPA	194	0	0	0	0	1	2
ENSP00000369757	RPS6	249	0	0	0	0	1	0
ENSP00000369979	CPXM1	734	0	0	0	0	1	3
ENSP00000370569	RPL21	160	0	0	0	0	1	0

ENSP00000370745	DDX1	740	0	0	0	0	2	0
ENSP00000371046	DDX17	652	0	0	0	0	1	0
ENSP00000371099	EIF3L	516	0	0	0	0	1	0
ENSP00000371243	TEX33	280	0	0	0	0	1	0
ENSP00000372445	METTL17	478	0	0	0	0	1	1
ENSP00000372689	STAG1	1258	0	0	0	0	1	0
ENSP00000373657	C17orf85	620	0	0	0	0	1	0
ENSP00000374574	DDX42	938	0	0	0	0	1	0
ENSP00000374630	SPTBN1	690	0	0	0	0	1	0
ENSP00000375730	RPL13A	203	0	0	0	0	1	0
ENSP00000376071	FAM117B	589	0	0	0	0	1	0
ENSP00000376371	SLC25A12	571	0	0	0	0	1	0
ENSP00000376436	ETS1	485	0	0	0	0	1	0
ENSP00000377219	DNM1	851	0	0	0	0	3	0
ENSP00000377500	PHLDB2	1237	0	0	0	0	1	1
ENSP00000377548	RBM14	156	0	0	0	0	1	0
ENSP00000377555	KRT36	417	0	0	0	0	1	1
ENSP00000377558	KRT35	455	0	0	0	0	2	0
ENSP00000377934	KIF7	1343	0	0	0	0	1	0
ENSP00000378341	PRKCE	219	0	0	0	0	1	0
ENSP00000378577	CLDN3	220	0	0	0	0	1	0
ENSP00000379029	KIF21A	1675	0	0	0	0	1	0
ENSP00000379042	ERO1L	468	0	0	0	0	1	0
ENSP00000379147	VAPB	99	0	0	0	0	1	1
ENSP00000379712	IQSEC2	1488	0	0	0	0	1	0
ENSP00000379801	AMPD3	767	0	0	0	0	1	1
ENSP00000379946	GLTSCR1	1560	0	0	0	0	1	0
ENSP00000380206	EIF3B	814	0	0	0	0	2	0
ENSP00000380373	JADE3	823	0	0	0	0	1	0
ENSP00000380560	KIF5C	725	0	0	0	0	2	1
ENSP00000380727	TMEM213	106	0	0	0	0	1	0
ENSP00000381247	EFCAB9	197	0	0	0	0	1	0
ENSP00000381494	KRTAP2-2	123	0	0	0	0	1	1
ENSP00000381727	GPR111	642	0	0	0	0	1	0
ENSP00000381992	SEPT7	438	0	0	0	0	2	0
ENSP00000383086	CSNK2A1	385	0	0	0	0	1	2
ENSP00000383115	COLEC12	742	0	0	0	0	1	0
ENSP00000383159	PCBP3	361	0	0	0	0	1	0
ENSP00000383163	PCBP3	345	0	0	0	0	1	0
ENSP00000383234	MAPK4	587	0	0	0	0	1	0
ENSP00000383279	UMODL1	1374	0	0	0	0	1	0
ENSP00000383444	XPO4	1151	0	0	0	0	1	1
ENSP00000383915	MAPRE3	266	0	0	0	0	1	0
ENSP00000384296	ACY1	408	0	0	0	0	1	0
ENSP00000384672	MBD5	1727	0	0	0	0	1	0
ENSP00000384774	HK1	921	0	0	0	0	1	0
ENSP00000385276	TENM3	2699	0	0	0	0	1	0
ENSP00000385715	MAPRE3	266	0	0	0	0	1	0
ENSP00000386049	MBD5	1494	0	0	0	0	1	0
ENSP00000386147	UMODL1	1318	0	0	0	0	1	0
ENSP00000386229	RGS14	566	0	0	0	0	1	0
ENSP00000386355	CCDC126	140	0	0	0	0	1	0
ENSP00000386522	DYNC1I2	630	0	0	0	0	1	0
ENSP00000386672	RAB1A	173	0	0	0	0	2	3
ENSP00000386794	ARHGEF4	1014	0	0	0	0	1	0
ENSP00000386883	CNRIP1	128	0	0	0	0	1	1
ENSP00000387691	HSPA1L	641	0	0	0	0	1	0
ENSP00000388082	CNTFR	256	0	0	0	0	1	0

ENSP00000388975	RALA	101	0	0	0	0	1	0
ENSP00000389441	UGGT1	131	0	0	0	0	1	0
ENSP00000389598	WBP2NL	273	0	0	0	0	0.5	0
ENSP00000389640	SLC39A10	126	0	0	0	0	0.5	0
ENSP00000390020	HLA-DOB	273	0	0	0	0	1	0
ENSP00000390121	DDX42	938	0	0	0	0	1	0
ENSP00000390265	IDH1	414	0	0	0	0	1	0
ENSP00000390715	PPP1CB	169	0	0	0	0	1	0
ENSP00000391211	PSMC2	433	0	0	0	0	1	0
ENSP00000391446	RPL14	124	0	0	0	0	1	0
ENSP00000391868	PNPLA1	533	0	0	0	0	1	0
ENSP00000393081	NSUN5	391	0	0	0	0	1	0
ENSP00000393087	HSPA1B	641	0	0	0	0	0.5	0
ENSP00000393265	ZNF226	803	0	0	0	0	1	0
ENSP00000393553	PTPRA	813	0	0	0	0	1	0
ENSP00000394170	AP3S2	124	0	0	0	0	1	0
ENSP00000394290	GNL1	607	0	0	0	0	1	0
ENSP00000394580	COMMD3	163	0	0	0	0	1	0
ENSP00000395438	AP2M1	252	0	0	0	0	1	3
ENSP00000395881	ALDH1L1	801	0	0	0	0	3	0
ENSP00000396000	AMPD3	608	0	0	0	0	1	1
ENSP00000396273	EHD1	162	0	0	0	0	3	0
ENSP00000398390	KIAA1429	1147	0	0	0	0	1	0
ENSP00000399162	MYO9A	2619	0	0	0	0	1	0
ENSP00000400048	VPS29	87	0	0	0	0	1	0
ENSP00000400330	PSMD2	203	0	0	0	0	1	0
ENSP00000400376	ERAP2	960	0	0	0	0	1	0
ENSP00000400433	HNRNPF	415	0	0	0	0	1	0
ENSP00000400625	DHTKD1	160	0	0	0	0	1	0
ENSP00000400766	DDX17	181	0	0	0	0	1	0
ENSP00000400855	DMXL2	2400	0	0	0	0	1	1
ENSP00000401328	PRSS48	328	0	0	0	0	1	1
ENSP00000402041	EPB41L2	747	0	0	0	0	1	0
ENSP00000402913	SEC24C	975	0	0	0	0	1	0
ENSP00000403448	NIPSNAP1	69	0	0	0	0	1	0
ENSP00000403637	LRPPRC	359	0	0	0	0	1	0
ENSP00000404269	SUSD1	281	0	0	0	0	1	0
ENSP00000404379	HECTD4	3996	0	0	0	0	1	0
ENSP00000405012	WDR87	2912	0	0	0	0	1	0
ENSP00000405354	MOB4	204	0	0	0	0	1	0
ENSP00000406640	TEX33	139	0	0	0	0	1	0
ENSP00000406656	WDR64	685	0	0	0	0	1	0
ENSP00000407919	NARS	299	0	0	0	0	1	0
ENSP00000408264	LSG1	285	0	0	0	0	1	0
ENSP00000410402	MAP2K4	410	0	0	0	0	1	0
ENSP00000410728	HNRNPU	241	0	0	0	0	1	0
ENSP00000410833	GBE1	702	0	0	0	0	1	0
ENSP00000411308	CTDSP1	195	0	0	0	0	1	0
ENSP00000411904	CPNE4	557	0	0	0	0	1	0
ENSP00000412720	TTPAL	279	0	0	0	0	1	0
ENSP00000413282	ITPA	177	0	0	0	0	1	1
ENSP00000413298	COPS3	345	0	0	0	0	1	0
ENSP00000413736	PRDX4	161	0	0	0	0	1	0
ENSP00000414650	RBM14	129	0	0	0	0	1	0
ENSP00000414803	LASP1	91	0	0	0	0	1	1
ENSP00000414909	C1orf167	1473	0	0	0	0	1	0
ENSP00000415256	TSPAN2	187	0	0	0	0	1	0
ENSP00000416832	CCT8	323	0	0	0	0	2	0

ENSP00000417056	ACY1	373	0	0	0	0	1	0
ENSP00000417279	CACNA2D3	997	0	0	0	0	1	0
ENSP00000417497	PHLDB2	519	0	0	0	0	1	1
ENSP00000417614	CAMKV	434	0	0	0	0	1	0
ENSP00000417967	TBRG4	118	0	0	0	0	1	0
ENSP00000418228	CACNA2D3	519	0	0	0	0	1	3
ENSP00000418447	PPP2CA	309	0	0	0	0	1	0
ENSP00000419101	CACNA2D3	1091	0	0	0	0	1	0
ENSP00000419526	RAB6B	83	0	0	0	0	1	1
ENSP00000419638	GBE1	661	0	0	0	0	1	0
ENSP00000420353	PRRG1	114	0	0	0	0	1	0
ENSP00000421011	ANK2	1611	0	0	0	0	1	0
ENSP00000421175	ERAP2	694	0	0	0	0	1	0
ENSP00000421320	TENM3	170	0	0	0	0	1	0
ENSP00000421678	CCSER1	70	0	0	0	0	1	0
ENSP00000422188	TTC23L	361	0	0	0	0	1	0
ENSP00000422329	RGS14	437	0	0	0	0	1	0
ENSP00000422455	NEDD4	910	0	0	0	0	1	0
ENSP00000423828	APC	1304	0	0	0	0	1	1
ENSP00000424595	PRPS1L1	318	0	0	0	0	1	0
ENSP00000424827	NEDD4	1319	0	0	0	0	1	0
ENSP00000424853	CPNE4	575	0	0	0	0	1	0
ENSP00000425242	TTC23L	287	0	0	0	0	1	0
ENSP00000425367	SAR1B	144	0	0	0	0	1	0
ENSP00000426312	MAP1B	122	0	0	0	0	1	0
ENSP00000426344	SEPT11	425	0	0	0	0	1	0
ENSP00000426410	FBXO38	943	0	0	0	0	1	0
ENSP00000426586	UBE2D3	118	0	0	0	0	1	0
ENSP00000426597	FYB	174	0	0	0	0	1	0
ENSP00000426923	CCT5	486	0	0	0	0	1	1
ENSP00000428955	NWD1	1358	0	0	0	0	1	0
ENSP00000429255	NCALD	136	0	0	0	0	1	0
ENSP00000429676	KIAA0196	1011	0	0	0	0	1	0
ENSP00000430555	PPP3CC	134	0	0	0	0	1	0
ENSP00000431229	ERCC2	405	0	0	0	0	1	0
ENSP00000431983	MUC15	361	0	0	0	0	1	0
ENSP00000434585	NPEPPS	163	0	0	0	0	1	0
ENSP00000434682	GDA	176	0	0	0	0	1	0
ENSP00000435199	NDUFV1	133	0	0	0	0	1	0
ENSP00000435481	AP003068.23	252	0	0	0	0	1	0
ENSP00000436971	RPS3	243	0	0	0	0	1	0
ENSP00000437164	AMPD2	761	0	0	0	0	1	0
ENSP00000437213	HLA-DOB	154	0	0	0	0	1	0
ENSP00000438061	HNRNPF	415	0	0	0	0	1	0
ENSP00000438247	PCMT1	192	0	0	0	0	1	1
ENSP00000438819	TAOK1	853	0	0	0	0	1	0
ENSP00000439869	STARD3	445	0	0	0	0	2	0
ENSP00000439990	PDZRN4	778	0	0	0	0	1	0
ENSP00000440240	KRT85	295	0	0	0	0	1	0
ENSP00000440660	DDB1	109	0	0	0	0	1	0
ENSP00000441006	GLI1	1065	0	0	0	0	1	0
ENSP00000441345	TCP1	221	0	0	0	0	1	1
ENSP00000441858	DMXL2	3037	0	0	0	0	2	1
ENSP00000441887	NCAM2	424	0	0	0	0	1	0
ENSP00000442461	NPEPPS	839	0	0	0	0	1	1
ENSP00000442508	PSMD3	356	0	0	0	0	1	0
ENSP00000443139	AKAP1	903	0	0	0	0	1	0
ENSP00000443821	OGDH	974	0	0	0	0	1	0

ENSP00000443831	REEP1	180	0	0	0	0	1	0
ENSP00000444439	SLC3A2	175	0	0	0	0	1	0
ENSP00000444650	DDB1	267	0	0	0	0	1	1
ENSP00000445345	SLC39A3	323	0	0	0	0	1	0
ENSP00000446168	MARS	546	0	0	0	0	2	0
ENSP00000446373	KRTAP2-1	124	0	0	0	0	1	1
ENSP00000446768	GPD1	326	0	0	0	0	1	2
ENSP00000446779	CS	400	0	0	0	0	1	0
ENSP00000447058	VPS29	182	0	0	0	0	1	1
ENSP00000447764	NACA	126	0	0	0	0	1	0
ENSP00000448177	PFKM	749	0	0	0	0	1	0
ENSP00000448532	HDAC7	974	0	0	0	0	1	0
ENSP00000449554	RAB5B	100	0	0	0	0	1	0
ENSP00000449783	KRT5	196	0	0	0	0	1	1
ENSP00000450691	NDRG2	252	0	0	0	0	1	0
ENSP00000451422	ATP6V1D	86	0	0	0	0	1	0
ENSP00000452237	KRT86	143	0	0	0	0	3	3
ENSP00000452492	ATP6V1D	59	0	0	0	0	1	0
ENSP00000452535	SAMD4A	718	0	0	0	0	1	0
ENSP00000452573	HNRNPC	117	0	0	0	0	1	1
ENSP00000452817	CCDC33	333	0	0	0	0	1	0
ENSP00000453267	DMXL2	1183	0	0	0	0	2	1
ENSP00000453569	DUOXA1	483	0	0	0	0	1	0
ENSP00000454446	MYO9A	1301	0	0	0	0	1	0
ENSP00000455284	CARHSP1	147	0	0	0	0	1	1
ENSP00000455421	NDRG4	154	0	0	0	0	1	0
ENSP00000456124	EPPK1	5088	0	0	0	0	2	0
ENSP00000456302	PMM2	155	0	0	0	0	1	0
ENSP00000457268	RPL4	170	0	0	0	0	1	0
ENSP00000457496	LPCAT2	188	0	0	0	0	1	0
ENSP00000457827	LOXL1	431	0	0	0	0	1	0
ENSP00000458114	TTC25	672	0	0	0	0	1	0
ENSP00000458243	KRTAP1-4	121	0	0	0	0	1	0
ENSP00000458934	RP1-18F17.1	436	0	0	0	0	2	1
ENSP00000459509	KRT33B	404	0	0	0	0	1	3
ENSP00000460739	KRT31	416	0	0	0	0	2	1
ENSP00000460741	P4HB	188	0	0	0	0	1	0
ENSP00000461346	CCDC84	332	0	0	0	0	1	0
ENSP00000462374	MBP	159	0	0	0	0	1	0
ENSP00000462512	COX10	168	0	0	0	0	1	0
ENSP00000463779	KSR1	130	0	0	0	0	1	0
ENSP00000464066	EIF4A1	214	0	0	0	0	1	0
ENSP00000464200	KPNB1	60	0	0	0	0	1	0
ENSP00000464265	UBBP4	229	0	0	0	0	1	1
ENSP00000464512	HELZ	1943	0	0	0	0	1	0
ENSP00000464813	RPL27	136	0	0	0	0	1	0
ENSP00000464874	ENO3	148	0	0	0	0	1	0
ENSP00000465304	SYT5	67	0	0	0	0	3	1
ENSP00000465680	ICAM1	180	0	0	0	0	1	0
ENSP00000466544	NCAM1	848	0	0	0	0	1	0
ENSP00000466558	TXNL1	138	0	0	0	0	1	0
ENSP00000466574	PGD	160	0	0	0	0	1	0
ENSP00000466914	DNM2	137	0	0	0	0	1	0
ENSP00000467398	PTPRS	1501	0	0	0	0	1	0
ENSP00000467963	HSPA12A	119	0	0	0	0	2	0
ENSP00000468484	H3F3B	151	0	0	0	0	1	0
ENSP00000469468	LSM4	139	0	0	0	0	1	0
ENSP00000469662	ARCN1	552	0	0	0	0	1	0

ENSP00000469896	PPP2R2D	422	0	0	0	0	1	0
ENSP00000470037	RPL13A	210	0	0	0	0	1	0
ENSP00000470142	UPF1	1129	0	0	0	0	1	0
ENSP00000470701	FAM120C	895	0	0	0	0	1	0
ENSP00000471000	ZNF616	781	0	0	0	0	1	1
ENSP00000471185	RPS5	200	0	0	0	0	1	3
ENSP00000471310	ADCK4	134	0	0	0	0	1	0
ENSP00000471621	RPS19	102	0	0	0	0	2	0
ENSP00000472264	UBA52	128	0	0	0	0	2	1
ENSP00000472265	ATP6AP1	470	0	0	0	0	1	0
ENSP00000472460	ENO2	391	0	0	0	0	3	1
ENSP00000472546	PLA2G4C	551	0	0	0	0	1	0
ENSP00000474440	DNAJC8	106	0	0	0	0	1	1
ENSP00000475565	UBA2	54	0	0	0	0	1	0
ENSP00000475950	LSM4	125	0	0	0	0	1	0
ENSP00000476832	GDI2	125	0	0	0	0	1	0
ENSP00000477496	TSPEAR	669	0	0	0	0	1	0
ENSP00000025399	STRAP	363	0	0	0	0	0	1
ENSP00000164640	PDZD4	769	0	0	0	0	0	1
ENSP00000205402	DLD	509	0	0	0	0	0	1
ENSP00000216605	MTHFD1	935	0	0	0	0	0	1
ENSP00000219169	NUTF2	127	0	0	0	0	0	1
ENSP00000225726	CCDC47	483	0	0	0	0	0	1
ENSP00000229922	CAP2	477	0	0	0	0	0	1
ENSP00000231721	SEMA3G	782	0	0	0	0	0	1
ENSP00000232905	EIF1B	113	0	0	0	0	0	1
ENSP00000237530	RPN2	631	0	0	0	0	0	1
ENSP00000237858	GLRX	106	0	0	0	0	0	1
ENSP00000238112	CPSF3	684	0	0	0	0	0	1
ENSP00000242315	KIAA1045	400	0	0	0	0	0	2
ENSP00000250448	FOXA1	472	0	0	0	0	0	1
ENSP00000251636	DHX29	1369	0	0	0	0	0	1
ENSP00000252322	EFCAB4B	395	0	0	0	0	0	1
ENSP00000252725	ARPC1B	372	0	0	0	0	0	1
ENSP00000255226	SLC14A2	920	0	0	0	0	0	1
ENSP00000256178	LYVE1	322	0	0	0	0	0	1
ENSP00000257017	RAB33A	237	0	0	0	0	0	1
ENSP00000258646	RCBTB1	531	0	0	0	0	0	1
ENSP00000260762	EXOC6	804	0	0	0	0	0	1
ENSP00000260983	HECW2	1572	0	0	0	0	0	1
ENSP00000261609	HERC2	4834	0	0	0	0	0	1
ENSP00000261632	IFT88	824	0	0	0	0	0	1
ENSP00000261776	VAC14	782	0	0	0	0	0	1
ENSP00000262320	AXIN1	862	0	0	0	0	0	1
ENSP00000262360	CNTLN	1405	0	0	0	0	0	1
ENSP00000262428	COTL1	142	0	0	0	0	0	1
ENSP00000262525	ZNF629	869	0	0	0	0	0	1
ENSP00000263368	BLVRB	206	0	0	0	0	0	1
ENSP00000263430	LILRB5	491	0	0	0	0	0	1
ENSP00000263867	CAPG	348	0	0	0	0	0	1
ENSP00000264158	RAB3GAP1	981	0	0	0	0	0	1
ENSP00000264414	CUL3	768	0	0	0	0	0	1
ENSP00000264613	CP	1065	0	0	0	0	0	1
ENSP00000264657	STAT3	770	0	0	0	0	0	1
ENSP00000265198	IPCEF1	437	0	0	0	0	0	1
ENSP00000265748	ANLN	1124	0	0	0	0	0	1
ENSP00000265840	ELMOD1	334	0	0	0	0	0	1
ENSP00000267396	REM2	340	0	0	0	0	0	1

ENSP00000267890	TTBK2	1244	0	0	0	0	0	1
ENSP00000272238	ATP6V1C2	427	0	0	0	0	0	1
ENSP00000273654	CCDC39	755	0	0	0	0	0	1
ENSP00000273794	VWA5B2	1024	0	0	0	0	0	1
ENSP00000276461	ERLIN2	339	0	0	0	0	0	1
ENSP00000277549	CACNA1B	1533	0	0	0	0	0	1
ENSP00000277551	CACNA1B	2237	0	0	0	0	0	1
ENSP00000278520	CCDC82	544	0	0	0	0	0	1
ENSP00000280758	BTBD11	1104	0	0	0	0	0	1
ENSP00000281405	WDR35	1170	0	0	0	0	0	1
ENSP00000282516	NIPBL	2804	0	0	0	0	0	1
ENSP00000284049	CHD1	1710	0	0	0	0	0	1
ENSP00000286627	KCNMA1	1178	0	0	0	0	0	1
ENSP00000286713	STOM	288	0	0	0	0	0	1
ENSP00000288048	C1orf158	194	0	0	0	0	0	1
ENSP00000289352	HIST1H4H	103	0	0	0	0	0	1
ENSP00000290178	PAXBP1	815	0	0	0	0	0	1
ENSP00000291906	PKN3	889	0	0	0	0	0	1
ENSP00000296412	ADH5	374	0	0	0	0	0	1
ENSP00000297186	RSPH10B2	870	0	0	0	0	0	1
ENSP00000297540	PHAX	394	0	0	0	0	0	1
ENSP00000298315	EML5	1931	0	0	0	0	0	1
ENSP00000298693	ARHGEF40	1471	0	0	0	0	0	1
ENSP00000299106	JAM3	310	0	0	0	0	0	1
ENSP00000300141	DPP8	882	0	0	0	0	0	1
ENSP00000300481	TRPM2	1449	0	0	0	0	0	2
ENSP00000300482	TRPM2	1503	0	0	0	0	0	1
ENSP00000301030	ANKRD11	2663	0	0	0	0	0	1
ENSP00000301624	TNRC6C	1690	0	0	0	0	0	1
ENSP00000301924	TRIM35	493	0	0	0	0	0	1
ENSP00000302237	GNAS	625	0	0	0	0	0	1
ENSP00000302873	GPS1	491	0	0	0	0	0	1
ENSP00000303153	COL22A1	1626	0	0	0	0	0	1
ENSP00000303659	RGPD3	1766	0	0	0	0	0	1
ENSP00000304408	COL3A1	1466	0	0	0	0	0	1
ENSP00000305161	TRIM56	755	0	0	0	0	0	1
ENSP00000305790	SF3B3	1217	0	0	0	0	0	1
ENSP00000306351	ZNF182	639	0	0	0	0	0	1
ENSP00000306983	YEATS2	1422	0	0	0	0	0	1
ENSP00000307156	LAMB2	1798	0	0	0	0	0	1
ENSP00000307272	RPTOR	1335	0	0	0	0	0	2
ENSP00000308268	CARNS1	827	0	0	0	0	0	1
ENSP00000308450	CDC20	499	0	0	0	0	0	1
ENSP00000309432	FAM134C	466	0	0	0	0	0	1
ENSP00000309560	C17orf66	570	0	0	0	0	0	1
ENSP00000309830	RPL38	70	0	0	0	0	0	2
ENSP00000310227	CKAP5	1972	0	0	0	0	0	1
ENSP00000310649	BRSK1	778	0	0	0	0	0	1
ENSP00000310723	DDX23	820	0	0	0	0	0	1
ENSP00000313007	PABPC1	636	0	0	0	0	0	1
ENSP00000313490	PFAS	1338	0	0	0	0	0	1
ENSP00000313569	GPS1	471	0	0	0	0	0	1
ENSP00000317955	EEA1	1411	0	0	0	0	0	1
ENSP00000320006	MPDZ	2070	0	0	0	0	0	1
ENSP00000320853	BRSK1	473	0	0	0	0	0	1
ENSP00000321519	C19orf18	215	0	0	0	0	0	1
ENSP00000324729	SAV1	383	0	0	0	0	0	1
ENSP00000325628	GRAMD1B	738	0	0	0	0	0	1

ENSP00000326547	USP54	744	0	0	0	0	0	1
ENSP00000326693	SLC25A42	318	0	0	0	0	0	1
ENSP00000328524	DENND5A	1287	0	0	0	0	0	1
ENSP00000329943	LRRC36	754	0	0	0	0	0	1
ENSP00000330199	SLC6A17	727	0	0	0	0	0	1
ENSP00000330219	SYN3	580	0	0	0	0	0	2
ENSP00000330484	AMY1B	511	0	0	0	0	0	1
ENSP00000330877	TEX40	200	0	0	0	0	0	1
ENSP00000331487	NPLOC4	608	0	0	0	0	0	1
ENSP00000332049	CD86	329	0	0	0	0	0	1
ENSP00000334090	AL603965.1	161	0	0	0	0	0	0.5
ENSP00000334714	IGFN1	3708	0	0	0	0	0	1
ENSP00000336739	CDC14A	594	0	0	0	0	0	1
ENSP00000336783	TNRC6C	1726	0	0	0	0	0	1
ENSP00000337724	ZNF202	648	0	0	0	0	0	1
ENSP00000338766	NPHP3	1330	0	0	0	0	0	1
ENSP00000339057	GPRASP2	838	0	0	0	0	0	1
ENSP00000339064	RPL32	153	0	0	0	0	0	1
ENSP00000339157	CPM	443	0	0	0	0	0	1
ENSP00000339208	DPP8	898	0	0	0	0	0	1
ENSP00000339247	CSNK2A1	391	0	0	0	0	0	1
ENSP00000339389	OTUD6A	288	0	0	0	0	0	1
ENSP00000340179	KIF24	1234	0	0	0	0	0	1
ENSP00000340278	PARK7	189	0	0	0	0	0	1
ENSP00000341466	KIF18B	855	0	0	0	0	0	1
ENSP00000341905	RASAL3	1011	0	0	0	0	0	1
ENSP00000342143	PDZK1	519	0	0	0	0	0	1
ENSP00000342278	OAS2	719	0	0	0	0	0	1
ENSP00000342487	CACUL1	303	0	0	0	0	0	1
ENSP00000343683	SNCA	112	0	0	0	0	0	1
ENSP00000344572	FIBP	364	0	0	0	0	0	1
ENSP00000345398	ACLY	1091	0	0	0	0	0	1
ENSP00000346001	RPL3	403	0	0	0	0	0	1
ENSP00000346120	DDX21	783	0	0	0	0	0	1
ENSP00000346321	KCNMA1	1239	0	0	0	0	0	1
ENSP00000346901	FMO1	532	0	0	0	0	0	1
ENSP00000346921	AK2	239	0	0	0	0	0	1
ENSP00000347379	OCLN	522	0	0	0	0	0	1
ENSP00000348020	MLH3	1453	0	0	0	0	0	1
ENSP00000348831	NUDT10	164	0	0	0	0	0	1
ENSP00000348889	ANXA6	667	0	0	0	0	0	1
ENSP00000349359	SASH3	380	0	0	0	0	0	1
ENSP00000349811	FKBP5	457	0	0	0	0	0	1
ENSP00000350392	C17orf102	167	0	0	0	0	0	1
ENSP00000351894	NCOA6	2063	0	0	0	0	0	1
ENSP00000352019	CCT6A	486	0	0	0	0	0	2
ENSP00000352920	ZMYM1	1142	0	0	0	0	0	1
ENSP00000354851	KIF21A	1661	0	0	0	0	0	1
ENSP00000354878	KIF21A	1674	0	0	0	0	0	1
ENSP00000355010	IGSF1	1336	0	0	0	0	0	1
ENSP00000355565	RBM34	425	0	0	0	0	0	1
ENSP00000355644	ACTA1	289	0	0	0	0	0	2
ENSP00000355739	ADCK3	647	0	0	0	0	0	1
ENSP00000355740	ADCK3	595	0	0	0	0	0	1
ENSP00000356129	DSTYK	884	0	0	0	0	0	1
ENSP00000356146	SCAF8	1271	0	0	0	0	0	1
ENSP00000356189	IPCEF1	438	0	0	0	0	0	1
ENSP00000356593	VTA1	249	0	0	0	0	0	1

ENSP00000356602	VTA1	307	0	0	0	0	0	1
ENSP00000357112	AIM2	343	0	0	0	0	0	1
ENSP00000357206	NES	1621	0	0	0	0	0	1
ENSP00000357285	LAMTOR2	150	0	0	0	0	0	1
ENSP00000357618	DPYSL4	412	0	0	0	0	0	1
ENSP00000358853	SH3BGRL2	107	0	0	0	0	0	2
ENSP00000359097	AMY1B	511	0	0	0	0	0	1
ENSP00000359947	IGSF1	1327	0	0	0	0	0	1
ENSP00000360598	EXOC6	153	0	0	0	0	0	1
ENSP00000361107	BMPR1A	532	0	0	0	0	0	1
ENSP00000361540	CDC20	499	0	0	0	0	0	1
ENSP00000361884	CAP1	474	0	0	0	0	0	1
ENSP00000362427	ZMYM1	1142	0	0	0	0	0	1
ENSP00000362724	RPN2	615	0	0	0	0	0	1
ENSP00000363512	ALOX5	674	0	0	0	0	0	1
ENSP00000363879	NPLOC4	617	0	0	0	0	0	1
ENSP00000364265	FOXE1	373	0	0	0	0	0	1
ENSP00000365383	C1orf158	156	0	0	0	0	0	1
ENSP00000365480	SYP	40	0	0	0	0	0	1
ENSP00000365773	PSAT1	370	0	0	0	0	0	1
ENSP00000366364	NEBL	236	0	0	0	0	0	1
ENSP00000366488	PRKACG	351	0	0	0	0	0	1
ENSP00000366708	PARK7	189	0	0	0	0	0	1
ENSP00000367034	HIST1H4C	103	0	0	0	0	0	1
ENSP00000367552	RCBTB1	531	0	0	0	0	0	1
ENSP00000368464	KIF24	1368	0	0	0	0	0	1
ENSP00000369375	TEK	1124	0	0	0	0	0	1
ENSP00000369991	KATNAL1	490	0	0	0	0	0	1
ENSP00000370196	SBF1	1893	0	0	0	0	0	1
ENSP00000370493	KIF2A	686	0	0	0	0	0	1
ENSP00000370526	ARSE	589	0	0	0	0	0	1
ENSP00000371548	LGI2	545	0	0	0	0	0	0.5
ENSP00000372005	DNAJC19	116	0	0	0	0	0	1
ENSP00000372191	SIRT3	399	0	0	0	0	0	1
ENSP00000372316	POLN	900	0	0	0	0	0	1
ENSP00000372683	ZBTB22	634	0	0	0	0	0	1
ENSP00000373586	C2orf54	447	0	0	0	0	0	1
ENSP00000373998	CCDC64B	508	0	0	0	0	0	1
ENSP00000375413	AC011841.1	149	0	0	0	0	0	1
ENSP00000375638	TFPT	244	0	0	0	0	0	1
ENSP00000376800	MTPN	118	0	0	0	0	0	2
ENSP00000377047	PTPRZ1	2315	0	0	0	0	0	1
ENSP00000377428	WDR52	1854	0	0	0	0	0	1
ENSP00000377549	FABP6	177	0	0	0	0	0	1
ENSP00000377570	KRT34	436	0	0	0	0	0	1
ENSP00000377969	GTF2F1	517	0	0	0	0	0	1
ENSP00000378336	MAP2K2	303	0	0	0	0	0	1
ENSP00000379654	KIAA0430	1742	0	0	0	0	0	1
ENSP00000379995	ATP6V1H	483	0	0	0	0	0	1
ENSP00000380068	GAPDH	335	0	0	0	0	0	1
ENSP00000380460	PLAA	795	0	0	0	0	0	1
ENSP00000381026	TRPM2	1553	0	0	0	0	0	2
ENSP00000381214	RGPD2	1756	0	0	0	0	0	1
ENSP00000381693	ZSWIM8	1842	0	0	0	0	0	1
ENSP00000381970	GIN1	522	0	0	0	0	0	1
ENSP00000382732	ITPA	153	0	0	0	0	0	1
ENSP00000384312	TRIOBP	2365	0	0	0	0	0	1
ENSP00000384766	RSPH10B2	870	0	0	0	0	0	1

ENSP00000385433	FABP6	128	0	0	0	0	0	1
ENSP00000385762	RPL3	272	0	0	0	0	0	1
ENSP00000386525	CUL3	744	0	0	0	0	0	1
ENSP00000386965	CAPG	348	0	0	0	0	0	1
ENSP00000387688	RPL35A	94	0	0	0	0	0	1
ENSP00000388325	LAMB2	1798	0	0	0	0	0	1
ENSP00000389631	ARPC1B	372	0	0	0	0	0	1
ENSP00000390088	TNK2	143	0	0	0	0	0	1
ENSP00000390262	EPB41L1	119	0	0	0	0	0	1
ENSP00000390802	WDR35	873	0	0	0	0	0	1
ENSP00000390987	KIT	972	0	0	0	0	0	1
ENSP00000391437	CPSF6	478	0	0	0	0	0	1
ENSP00000392270	STRAP	350	0	0	0	0	0	1
ENSP00000392408	ZZZ3	36	0	0	0	0	0	1
ENSP00000393779	ZZZ3	57	0	0	0	0	0	1
ENSP00000394485	PDZK1	519	0	0	0	0	0	1
ENSP00000394874	C2orf54	108	0	0	0	0	0	1
ENSP00000396320	SCN4A	1836	0	0	0	0	0	1
ENSP00000397002	TEX22	150	0	0	0	0	0	1
ENSP00000397156	CCDC82	544	0	0	0	0	0	1
ENSP00000398058	RPL35A	55	0	0	0	0	0	1
ENSP00000398189	UCHL3	228	0	0	0	0	0	1
ENSP00000398316	HK1	114	0	0	0	0	0	1
ENSP00000398688	VWA5B2	1242	0	0	0	0	0	1
ENSP00000398779	PPP2R2B	443	0	0	0	0	0	1
ENSP00000400000	RBM34	408	0	0	0	0	0	1
ENSP00000400435	SLC35E1	344	0	0	0	0	0	1
ENSP00000401010	CAPZB	301	0	0	0	0	0	1
ENSP00000401519	CARNS1	827	0	0	0	0	0	1
ENSP00000402343	ZNF469	3925	0	0	0	0	0	1
ENSP00000402592	AP2M1	160	0	0	0	0	0	1
ENSP00000404186	TRIM56	305	0	0	0	0	0	1
ENSP00000404710	USP54	153	0	0	0	0	0	1
ENSP00000404979	ANLN	169	0	0	0	0	0	1
ENSP00000406982	FMO1	208	0	0	0	0	0	1
ENSP00000407748	CCT8	107	0	0	0	0	0	1
ENSP00000410474	CTBP2	445	0	0	0	0	0	1
ENSP00000412388	C9orf172	976	0	0	0	0	0	1
ENSP00000412393	RPL32	133	0	0	0	0	0	1
ENSP00000414225	LILRB5	622	0	0	0	0	0	1
ENSP00000416968	DCTN4	460	0	0	0	0	0	1
ENSP00000417016	DLD	486	0	0	0	0	0	1
ENSP00000417371	WDR52	991	0	0	0	0	0	1
ENSP00000417960	CCDC39	671	0	0	0	0	0	1
ENSP00000418749	ZNF76	101	0	0	0	0	0	1
ENSP00000418773	CP	946	0	0	0	0	0	2
ENSP00000418957	CPSF3	647	0	0	0	0	0	1
ENSP00000418988	CD86	247	0	0	0	0	0	1
ENSP00000419100	PLCH1	1014	0	0	0	0	0	1
ENSP00000419191	DNAJC19	91	0	0	0	0	0	1
ENSP00000419744	CPSF3	136	0	0	0	0	0	1
ENSP00000420545	CP	852	0	0	0	0	0	2
ENSP00000421170	MYO10	1397	0	0	0	0	0	1
ENSP00000421309	MYO10	830	0	0	0	0	0	1
ENSP00000424245	CAMK2D	512	0	0	0	0	0	1
ENSP00000424740	CANX	170	0	0	0	0	0	1
ENSP00000424760	GNB2L1	147	0	0	0	0	0	1
ENSP00000426401	POLN	524	0	0	0	0	0	1

ENSP00000426676	EPN3	17	0	0	0	0	0	1
ENSP00000428069	ATP6V1B2	56	0	0	0	0	0	3
ENSP00000428816	PPP2CA	145	0	0	0	0	0	1
ENSP00000428948	PABPC1	165	0	0	0	0	0	1
ENSP00000429229	ERLIN2	197	0	0	0	0	0	1
ENSP00000430447	PLAA	290	0	0	0	0	0	1
ENSP00000430686	TEK	976	0	0	0	0	0	1
ENSP00000431223	ZNF202	126	0	0	0	0	0	1
ENSP00000431329	CACUL1	303	0	0	0	0	0	1
ENSP00000431457	FIBP	137	0	0	0	0	0	1
ENSP00000431988	EPB41L2	706	0	0	0	0	0	1
ENSP00000432472	BRK1	75	0	0	0	0	0	1
ENSP00000432549	DENND5A	630	0	0	0	0	0	1
ENSP00000432768	CKAP5	2032	0	0	0	0	0	1
ENSP00000433232	ELMOD1	328	0	0	0	0	0	1
ENSP00000434750	OPCML	338	0	0	0	0	0	1
ENSP00000435130	GRHL3	52	0	0	0	0	0	1
ENSP00000436500	GRAMD1B	738	0	0	0	0	0	1
ENSP00000436792	CSRP1	193	0	0	0	0	0	1
ENSP00000437216	SIRT3	257	0	0	0	0	0	1
ENSP00000437394	GPRASP2	838	0	0	0	0	0	1
ENSP00000438198	ARSE	544	0	0	0	0	0	1
ENSP00000438588	MTHFD1	1020	0	0	0	0	0	1
ENSP00000439693	SLC24A1	1012	0	0	0	0	0	1
ENSP00000440243	UBAP1L	381	0	0	0	0	0	1
ENSP00000441241	LDHA	144	0	0	0	0	0	1
ENSP00000441883	RAB35	152	0	0	0	0	0	1
ENSP00000441942	XPNPEP3	484	0	0	0	0	0	1
ENSP00000442074	DAD1	65	0	0	0	0	0	1
ENSP00000442479	RPTOR	1177	0	0	0	0	0	2
ENSP00000443865	SPI1	35	0	0	0	0	0	1
ENSP00000444138	KIAA1045	400	0	0	0	0	0	2
ENSP00000444250	SLC3A2	47	0	0	0	0	0	1
ENSP00000444306	RAB3GAP1	935	0	0	0	0	0	1
ENSP00000444369	C10orf90	796	0	0	0	0	0	1
ENSP00000444659	BRK1	105	0	0	0	0	0	1
ENSP00000444987	ACTR3	367	0	0	0	0	0	1
ENSP00000445412	FKBP5	278	0	0	0	0	0	1
ENSP00000445764	STOM	237	0	0	0	0	0	1
ENSP00000446358	MPDZ	2084	0	0	0	0	0	1
ENSP00000447030	TMBIM6	200	0	0	0	0	0	1
ENSP00000447113	ZBTB22	634	0	0	0	0	0	1
ENSP00000447455	CPM	198	0	0	0	0	0	1
ENSP00000447788	DNM1L	261	0	0	0	0	0	1
ENSP00000448322	BTBD11	183	0	0	0	0	0	1
ENSP00000449003	AK2	190	0	0	0	0	0	1
ENSP00000449371	CUX1	662	0	0	0	0	0	1
ENSP00000450309	KIAA0430	1742	0	0	0	0	0	1
ENSP00000451130	MLH3	477	0	0	0	0	0	1
ENSP00000451492	SAV1	312	0	0	0	0	0	1
ENSP00000451651	POMT2	193	0	0	0	0	0	1
ENSP00000451966	NDRG2	300	0	0	0	0	0	1
ENSP00000451998	EML5	1977	0	0	0	0	0	1
ENSP00000452794	UBAP1L	117	0	0	0	0	0	1
ENSP00000452974	PIGH	133	0	0	0	0	0	1
ENSP00000453679	RAB8B	92	0	0	0	0	0	2
ENSP00000455103	LRRC36	633	0	0	0	0	0	1
ENSP00000455653	CUX1	662	0	0	0	0	0	1

ENSP00000456267	ABAT	65	0	0	0	0	0	1
ENSP00000456315	GSTM1	102	0	0	0	0	0	1
ENSP00000456330	ABAT	515	0	0	0	0	0	1
ENSP00000458262	PAXBP1	917	0	0	0	0	0	1
ENSP00000458301	PRPSAP2	260	0	0	0	0	0	1
ENSP00000458767	HERC2	4834	0	0	0	0	0	1
ENSP00000459436	OCLN	130	0	0	0	0	0	1
ENSP00000460902	GIN1	522	0	0	0	0	0	1
ENSP00000461221	KRT33A	404	0	0	0	0	0	1
ENSP00000461241	PRPSAP2	143	0	0	0	0	0	1
ENSP00000462265	GPS1	487	0	0	0	0	0	1
ENSP00000462837	RPL38	64	0	0	0	0	0	1
ENSP00000463309	EIF4A1	134	0	0	0	0	0	1
ENSP00000463312	RPL38	67	0	0	0	0	0	2
ENSP00000463895	IMPACT	106	0	0	0	0	0	2
ENSP00000463963	SCN4A	1836	0	0	0	0	0	1
ENSP00000465004	FAM134C	129	0	0	0	0	0	1
ENSP00000465377	KIF18B	833	0	0	0	0	0	0.5
ENSP00000465576	SYT5	382	0	0	0	0	0	3
ENSP00000465953	SLC14A2	920	0	0	0	0	0	1
ENSP00000465992	KIF18B	852	0	0	0	0	0	1
ENSP00000466321	EIF1	121	0	0	0	0	0	1
ENSP00000466656	PIN1	145	0	0	0	0	0	1
ENSP00000466780	PIN1	90	0	0	0	0	0	1
ENSP00000467173	STAT3	84	0	0	0	0	0	1
ENSP00000469091	GTF2F1	433	0	0	0	0	0	1
ENSP00000470057	MCM3	77	0	0	0	0	0	1
ENSP00000470861	FLNA	604	0	0	0	0	0	1
ENSP00000471018	NAPA	137	0	0	0	0	0	1
ENSP00000471403	GTF2F1	381	0	0	0	0	0	1
ENSP00000471468	NUDT10	164	0	0	0	0	0	1
ENSP00000471720	BLVRB	163	0	0	0	0	0	1
ENSP00000471874	RPS11	88	0	0	0	0	0	1
ENSP00000473537	C10orf131	61	0	0	0	0	0	1
ENSP00000474063	AP2B1	63	0	0	0	0	0	1
ENSP00000474567	TEX22	63	0	0	0	0	0	1
ENSP00000474893	C17orf66	340	0	0	0	0	0	1
ENSP00000475098	HNRNPK	77	0	0	0	0	0	1
ENSP00000477059	XKR7	579	0	0	0	0	0	1
ENSP00000477243	TFPT	253	0	0	0	0	0	1

Supplementary table S2. A table of differentially expressed proteins in NBE-MSC-MV.

Protein ID	LogFold Change	FDR	Gene Symbol	Description
ENSP00000261205	5.005	0	SYT1	synaptotagmin I [Source:HGNC Symbol;Acc:11509]
ENSP00000302397	4.869	0	ATP1A3	ATPase, Na+/K+ transporting, alpha 3 polypeptide [Source:HGNC Symbol;Acc:801]
ENSP00000305152	4.644	0	PLP1	proteolipid protein 1 [Source:HGNC Symbol;Acc:9086]
ENSP00000345680	4.225	0	DNM1	dynamin 1 [Source:HGNC Symbol;Acc:2972]
ENSP00000454728	4.088	0	GNAO1	guanine nucleotide binding protein (G protein), alpha activating activity polypeptide O [Source:HGNC Symbol;Acc:4389]
ENSP00000295987	4.075	0	SYN1	synapsin I [Source:HGNC Symbol;Acc:11494]
ENSP00000455899	4.05	0	STX1B	syntaxin 1B [Source:HGNC Symbol;Acc:18539]
ENSP00000419225	4.049	0	DNM1	dynamin 1 [Source:HGNC Symbol;Acc:2972]
ENSP00000362396	4.049	0	STXBP1	syntaxin binding protein 1 [Source:HGNC Symbol;Acc:11444]
ENSP00000469129	4.048	0	ATP1A3	ATPase, Na+/K+ transporting, alpha 3 polypeptide [Source:HGNC Symbol;Acc:801]
ENSP00000215095	4.036	0	STX1B	syntaxin 1B [Source:HGNC Symbol;Acc:18539]
ENSP00000264071	3.926	0	TUBB4A	tubulin, beta 4A class IVa [Source:HGNC Symbol;Acc:20774]
ENSP00000342951	3.817	0	ATP6V0A1	ATPase, H+ transporting, lysosomal V0 subunit a1 [Source:HGNC Symbol;Acc:865]
ENSP00000216254	3.765	0	ACO2	aconitase 2, mitochondrial [Source:HGNC Symbol;Acc:118]
ENSP00000225282	3.698	0	NSF	N-ethylmaleimide-sensitive factor [Source:HGNC Symbol;Acc:8016]
ENSP00000396061	3.654	0	TUBA4A	tubulin, alpha 4a [Source:HGNC Symbol;Acc:12407]
ENSP00000261160	3.638	0	CNTN1	contactin 1 [Source:HGNC Symbol;Acc:2171]
ENSP00000264649	3.63	0	ATP6V0A1	ATPase, H+ transporting, lysosomal V0 subunit a1 [Source:HGNC Symbol;Acc:865]
ENSP00000302777	3.626	0	TUBB3	Tubulin beta-3 chain [Source:UniProtKB/Swiss-Prot;Acc:Q13509]
ENSP00000254976	3.582	0	SNAP25	synaptosomal-associated protein, 25kDa [Source:HGNC Symbol;Acc:11132]
ENSP00000380104	3.553	0	HBE1	hemoglobin, epsilon 1 [Source:HGNC Symbol;Acc:4830]
ENSP00000354490	3.518	0	ATP1A2	ATPase, Na+/K+ transporting, alpha 2 polypeptide [Source:HGNC Symbol;Acc:800]
ENSP00000381293	3.5	0	NSF	N-ethylmaleimide-sensitive factor [Source:HGNC Symbol;Acc:8016]
ENSP00000476705	3.495	0	ATP1A3	ATPase, Na+/K+ transporting, alpha 3 polypeptide [Source:HGNC Symbol;Acc:801]
ENSP00000307341	3.481	0	SNAP25	synaptosomal-associated protein, 25kDa [Source:HGNC Symbol;Acc:11132]
ENSP00000222286	3.464	0	GAPDHS	glyceraldehyde-3-phosphate dehydrogenase, spermatogenic [Source:HGNC Symbol;Acc:24864]
ENSP00000299198	3.449	0	CKB	creatine kinase, brain [Source:HGNC Symbol;Acc:1991]
ENSP00000292896	3.442	0	HBE1	hemoglobin, epsilon 1 [Source:HGNC Symbol;Acc:4830]
ENSP00000462223	3.442	0	MBP	myelin basic protein [Source:HGNC Symbol;Acc:6925]
ENSP00000356787	3.42	0	ATP1B1	ATPase, Na+/K+ transporting, beta 1 polypeptide [Source:HGNC Symbol;Acc:804]
ENSP00000356789	3.364	0	ATP1B1	ATPase, Na+/K+ transporting, beta 1 polypeptide [Source:HGNC Symbol;Acc:804]
ENSP00000449415	3.343	0	SYT1	synaptotagmin I [Source:HGNC Symbol;Acc:11509]
ENSP00000348394	3.333	0.000001	NCDN	neurochondrin [Source:HGNC Symbol;Acc:17597]
ENSP00000362340	3.327	0	NCDN	neurochondrin [Source:HGNC Symbol;Acc:17597]
ENSP00000311186	3.275	0	ATP2A2	ATPase, Ca++ transporting, cardiac muscle, slow twitch 2 [Source:HGNC Symbol;Acc:812]
ENSP00000218548	3.268	0	ATP12A	ATPase, H+/K+ transporting, nongastric, alpha polypeptide [Source:HGNC Symbol;Acc:13816]
ENSP00000222256	3.261	0	RAB3A	RAB3A, member RAS oncogene family [Source:HGNC Symbol;Acc:9777]
ENSP00000318472	3.251	0	NCAM1	neural cell adhesion molecule 1 [Source:HGNC Symbol;Acc:7656]
ENSP00000466696	3.242	0	NCAM1	neural cell adhesion molecule 1 [Source:HGNC Symbol;Acc:7656]
ENSP00000259818	3.231	0	TUBB2B	tubulin, beta 2B class IIb [Source:HGNC Symbol;Acc:30829]
ENSP00000262493	3.223	0	GNAO1	guanine nucleotide binding protein (G protein), alpha activating activity polypeptide O [Source:HGNC Symbol;Acc:4389]
ENSP00000421578	3.209	0	GPM6A	glycoprotein M6A [Source:HGNC Symbol;Acc:4460]
ENSP00000262494	3.204	0	GNAO1	guanine nucleotide binding protein (G protein), alpha activating activity polypeptide O [Source:HGNC Symbol;Acc:4389]
ENSP00000405750	3.185	0	PLP1	proteolipid protein 1 [Source:HGNC Symbol;Acc:9086]
ENSP00000222812	3.165	0	STX1A	syntaxin 1A (brain) [Source:HGNC Symbol;Acc:11433]
ENSP00000362399	3.164	0	STXBP1	syntaxin binding protein 1 [Source:HGNC Symbol;Acc:11444]
ENSP00000292901	3.145	0	HBD	hemoglobin, delta [Source:HGNC Symbol;Acc:4829]
ENSP00000380267	3.131	0	ATP2B2	ATPase, Ca++ transporting, plasma membrane 2 [Source:HGNC Symbol;Acc:815]
ENSP00000451617	3.106	0	TUBB3	Tubulin beta-3 chain [Source:UniProtKB/Swiss-Prot;Acc:Q13509]
ENSP00000382981	3.063	0	EPB41L3	erythrocyte membrane protein band 4.1-like 3 [Source:HGNC Symbol;Acc:3380]
ENSP00000340716	3.051	0.000041	RTN1	reticulon 1 [Source:HGNC Symbol;Acc:10467]
ENSP00000429701	3.043	0	DNM3	dynamin 3 [Source:HGNC Symbol;Acc:29125]
ENSP00000341138	3.039	0	EPB41L3	erythrocyte membrane protein band 4.1-like 3 [Source:HGNC Symbol;Acc:3380]
ENSP00000222120	3.037	0	RAB3D	RAB3D, member RAS oncogene family [Source:HGNC Symbol;Acc:9779]
ENSP00000375938	3.019	0	TUBA4A	tubulin, alpha 4a [Source:HGNC Symbol;Acc:12407]
ENSP00000369602	2.983	0	HBG2	hemoglobin, gamma G [Source:HGNC Symbol;Acc:4832]
ENSP00000253413	2.981	0	ATP6V1E1	ATPase, H+ transporting, lysosomal 31kDa, V1 subunit E1 [Source:HGNC Symbol;Acc:857]
ENSP00000387694	2.968	0	SLC12A5	solute carrier family 12 (potassium/chloride transporter), member 5 [Source:HGNC Symbol;Acc:13818]

ENSP00000243964	2.93	0	SLC12A5	solute carrier family 12 (potassium/chloride transporter), member 5 [Source:HGNC Symbol;Acc:13818]
ENSP00000357060	2.911	0	ATP1A4	ATPase, Na+/K+ transporting, alpha 4 polypeptide [Source:HGNC Symbol;Acc:14073]
ENSP00000400720	2.899	0	SNAP25	synaptosomal-associated protein, 25kDa [Source:HGNC Symbol;Acc:11132]
ENSP00000471581	2.882	0	ATP1A3	ATPase, Na+/K+ transporting, alpha 3 polypeptide [Source:HGNC Symbol;Acc:801]
ENSP00000369654	2.877	0	HBD	hemoglobin, delta [Source:HGNC Symbol;Acc:4829]
ENSP00000278379	2.842	0.000001	SLC1A2	solute carrier family 1 (glial high affinity glutamate transporter), member 2 [Source:HGNC Symbol;Acc:10940]
ENSP00000470571	2.841	0	SYP	synaptophysin [Source:HGNC Symbol;Acc:11506]
ENSP00000280187	2.811	0	GPM6A	glycoprotein M6A [Source:HGNC Symbol;Acc:4460]
ENSP00000354111	2.765	0	DNAJC5	DnaJ (Hsp40) homolog, subfamily C, member 5 [Source:HGNC Symbol;Acc:16235]
ENSP00000380987	2.762	0	CRMP1	collapsin response mediator protein 1 [Source:HGNC Symbol;Acc:2365]
ENSP00000436029	2.76	0.000033	SLC1A2	solute carrier family 1 (glial high affinity glutamate transporter), member 2 [Source:HGNC Symbol;Acc:10940]
ENSP00000328455	2.756	0	LSAMP	limbic system-associated membrane protein [Source:HGNC Symbol;Acc:6705]
ENSP00000258682	2.754	0	CAMK2B	calcium/calmodulin-dependent protein kinase II beta [Source:HGNC Symbol;Acc:1461]
ENSP00000322234	2.743	0.000004	SYNJ1	synaptojanin 1 [Source:HGNC Symbol;Acc:11503]
ENSP00000251595	2.738	0	HBA2	hemoglobin, alpha 2 [Source:HGNC Symbol;Acc:4824]
ENSP00000299518	2.725	0	IDH3A	isocitrate dehydrogenase 3 (NAD+) alpha [Source:HGNC Symbol;Acc:5384]
ENSP00000261793	2.717	0	CAMK2A	calcium/calmodulin-dependent protein kinase II alpha [Source:HGNC Symbol;Acc:1460]
ENSP00000273398	2.714	0	ATP6V1A	ATPase, H+ transporting, lysosomal 70kDa, V1 subunit A [Source:HGNC Symbol;Acc:851]
ENSP00000309539	2.7	0	DPYSL2	dihydropyrimidinase-like 2 [Source:HGNC Symbol;Acc:3014]
ENSP00000448653	2.698	0.000002	CNTN1	contactin 1 [Source:HGNC Symbol;Acc:2171]
ENSP00000324804	2.682	0	PPP2R1A	protein phosphatase 2, regulatory subunit A, alpha [Source:HGNC Symbol;Acc:9302]
ENSP00000324172	2.652	0	ATP2B2	ATPase, Ca++ transporting, plasma membrane 2 [Source:HGNC Symbol;Acc:815]
ENSP00000369703	2.647	0	TUBB2A	tubulin, beta 2A class IIa [Source:HGNC Symbol;Acc:12412]
ENSP00000348273	2.644	0	MBP	myelin basic protein [Source:HGNC Symbol;Acc:6925]
ENSP00000385447	2.644	0.000026	SYNGR1	synaptogyrin 1 [Source:HGNC Symbol;Acc:11498]
ENSP00000382696	2.605	0.000001	ATP6V1E1	ATPase, H+ transporting, lysosomal 31kDa, V1 subunit E1 [Source:HGNC Symbol;Acc:857]
ENSP00000358211	2.598	0	HSPA12A	heat shock 70kDa protein 12A [Source:HGNC Symbol;Acc:19022]
ENSP00000452166	2.595	0.000001	TUBB3	Tubulin beta-3 chain [Source:UniProtKB/Swiss-Prot;Acc:Q13509]
ENSP00000463016	2.584	0	SV2A	synaptic vesicle glycoprotein 2A [Source:HGNC Symbol;Acc:20566]
ENSP00000217133	2.584	0	TUBB1	tubulin, beta 1 class VI [Source:HGNC Symbol;Acc:16257]
ENSP00000343206	2.577	0.000001	SYN1	synapsin I [Source:HGNC Symbol;Acc:11494]
ENSP00000267484	2.572	0.000001	RTN1	reticulon 1 [Source:HGNC Symbol;Acc:10467]
ENSP00000353174	2.554	0.000002	RYR2	ryanodine receptor 2 (cardiac) [Source:HGNC Symbol;Acc:10484]
ENSP00000434744	2.553	0	DNAJC5	DnaJ (Hsp40) homolog, subfamily C, member 5 [Source:HGNC Symbol;Acc:16235]
ENSP00000351155	2.55	0.000021	ATL1	atlastin GTPase 1 [Source:HGNC Symbol;Acc:11231]
ENSP00000458221	2.548	0.000006	NSF	N-ethylmaleimide-sensitive factor [Source:HGNC Symbol;Acc:8016]
ENSP00000322421	2.541	0.000004	HBA1	hemoglobin, alpha 1 [Source:HGNC Symbol;Acc:4823]
ENSP00000341289	2.541	0	TUBB4B	tubulin, beta 4B class IVb [Source:HGNC Symbol;Acc:20771]
ENSP00000301387	2.537	0	ATP2A3	ATPase, Ca++ transporting, ubiquitous [Source:HGNC Symbol;Acc:813]
ENSP00000318697	2.513	0	TUBB6	tubulin, beta 6 class V [Source:HGNC Symbol;Acc:20776]
ENSP00000465154	2.502	0.000024	NCAM1	neural cell adhesion molecule 1 [Source:HGNC Symbol;Acc:7656]
ENSP00000379769	2.493	0	ACO2	aconitase 2, mitochondrial [Source:HGNC Symbol;Acc:118]
ENSP00000309431	2.489	0.000011	RP11-683L23.1	Tubulin beta-8 chain-like protein LOC260334 [Source:UniProtKB/Swiss-Prot;Acc:A6NNZ2]
ENSP00000477015	2.466	0.000004	ATP1B1	ATPase, Na+/K+ transporting, beta 1 polypeptide [Source:HGNC Symbol;Acc:804]
ENSP00000358141	2.465	0.000011	SV2A	synaptic vesicle glycoprotein 2A [Source:HGNC Symbol;Acc:20566]
ENSP00000472335	2.456	0.000005	RAB3A	RAB3A, member RAS oncogene family [Source:HGNC Symbol;Acc:9777]
ENSP00000459646	2.452	0.000004	NSF	N-ethylmaleimide-sensitive factor [Source:HGNC Symbol;Acc:8016]
ENSP00000362413	2.451	0	PGK1	phosphoglycerate kinase 1 [Source:HGNC Symbol;Acc:8896]
ENSP00000451378	2.438	0.000012	TUBB3	Tubulin beta-3 chain [Source:UniProtKB/Swiss-Prot;Acc:Q13509]
ENSP00000347359	2.428	0.000048	ATP6V1H	ATPase, H+ transporting, lysosomal 50/57kDa, V1 subunit H [Source:HGNC Symbol;Acc:18303]
ENSP00000276390	2.426	0	ATP6V1B2	ATPase, H+ transporting, lysosomal 56/58kDa, V1 subunit B2 [Source:HGNC Symbol;Acc:854]
ENSP00000346522	2.419	0.00018	ATL1	atlastin GTPase 1 [Source:HGNC Symbol;Acc:11231]
ENSP00000439874	2.413	0.000038	ATP6V1A	ATPase, H+ transporting, lysosomal 70kDa, V1 subunit A [Source:HGNC Symbol;Acc:851]
ENSP00000411705	2.405	0	ATP1A2	ATPase, Na+/K+ transporting, alpha 2 polypeptide [Source:HGNC Symbol;Acc:800]
ENSP00000352398	2.403	0.000007	HK1	hexokinase 1 [Source:HGNC Symbol;Acc:4922]
ENSP00000262623	2.394	0	ATP4A	ATPase, H+/K+ exchanging, alpha polypeptide [Source:HGNC Symbol;Acc:819]
ENSP00000413649	2.393	0.000036	SYNJ1	synaptojanin 1 [Source:HGNC Symbol;Acc:11503]
ENSP00000447006	2.39	0.000001	CNTN1	contactin 1 [Source:HGNC Symbol;Acc:2171]
ENSP00000316861	2.366	0.00001	GPM6B	glycoprotein M6B [Source:HGNC Symbol;Acc:4461]
ENSP00000381412	2.365	0.000077	CAMK2A	calcium/calmodulin-dependent protein kinase II alpha [Source:HGNC Symbol;Acc:1460]
ENSP00000386192	2.358	0.000012	DNM2	dynamin 2 [Source:HGNC Symbol;Acc:2974]

ENSP00000321606	2.353	0.000311	CRMP1	collapsin response mediator protein 1 [Source:HGNC Symbol;Acc:2365]
ENSP00000320295	2.353	0.000023	TUBB3	Tubulin beta-3 chain [Source:UniProtKB/Swiss-Prot;Acc:Q13509]
ENSP00000398320	2.348	0.00038	GLUL	glutamate-ammonia ligase [Source:HGNC Symbol;Acc:4341]
ENSP00000387506	2.335	0.000024	IDH3A	isocitrate dehydrogenase 3 (NAD+) alpha [Source:HGNC Symbol;Acc:5384]
ENSP00000362350	2.328	0.000079	NCDN	neurochondrin [Source:HGNC Symbol;Acc:17597]
ENSP00000450538	2.321	0.000145	TUBB3	Tubulin beta-3 chain [Source:UniProtKB/Swiss-Prot;Acc:Q13509]
ENSP00000455774	2.319	0.000015	GNAO1	guanine nucleotide binding protein (G protein), alpha activating activity polypeptide O [Source:HGNC Symbol;Acc:4389]
ENSP00000403335	2.319	0.000002	PLP1	proteolipid protein 1 [Source:HGNC Symbol;Acc:9086]
ENSP00000467399	2.317	0	VAMP2	vesicle-associated membrane protein 2 (synaptobrevin 2) [Source:HGNC Symbol;Acc:12643]
ENSP00000283269	2.304	0.000061	CADPS	Ca++-dependent secretion activator [Source:HGNC Symbol;Acc:1426]
ENSP00000472481	2.304	0	TUBB4A	tubulin, beta 4A class IVa [Source:HGNC Symbol;Acc:20774]
ENSP00000379099	2.302	0.000021	SLC1A2	solute carrier family 1 (glial high affinity glutamate transporter), member 2 [Source:HGNC Symbol;Acc:10940]
ENSP00000356236	2.301	0.000105	SYT2	synaptotagmin II [Source:HGNC Symbol;Acc:11510]
ENSP00000366225	2.296	0.000102	NAPB	N-ethylmaleimide-sensitive factor attachment protein, beta [Source:HGNC Symbol;Acc:15751]
ENSP00000449426	2.293	0.000019	PFKM	phosphofructokinase, muscle [Source:HGNC Symbol;Acc:8877]
ENSP00000418799	2.284	0	TUBAL3	tubulin, alpha-like 3 [Source:HGNC Symbol;Acc:23534]
ENSP00000290949	2.269	0	ATP6V0D1	ATPase, H+ transporting, lysosomal 38kDa, V0 subunit d1 [Source:HGNC Symbol;Acc:13724]
ENSP00000346545	2.268	0.000079	MBP	myelin basic protein [Source:HGNC Symbol;Acc:6925]
ENSP00000378872	2.267	0.000617	ATP2A2	ATPase, Ca++ transporting, cardiac muscle, slow twitch 2 [Source:HGNC Symbol;Acc:812]
ENSP00000468571	2.267	0.0002	TUBB6	tubulin, beta 6 class V [Source:HGNC Symbol;Acc:20776]
ENSP00000384014	2.244	0.000431	VSNL1	visinin-like 1 [Source:HGNC Symbol;Acc:12722]
ENSP00000268251	2.243	0.000213	ABAT	4-aminobutyrate aminotransferase [Source:HGNC Symbol;Acc:23]
ENSP00000318845	2.24	0.000182	SYNGR1	synaptogyrin 1 [Source:HGNC Symbol;Acc:11498]
ENSP00000427985	2.21	0	DPYSL2	dihydropyrimidinase-like 2 [Source:HGNC Symbol;Acc:3014]
ENSP00000342056	2.202	0.000223	CS	citrate synthase [Source:HGNC Symbol;Acc:2422]
ENSP00000313164	2.199	0.000134	DNM2	dynamin 2 [Source:HGNC Symbol;Acc:2974]
ENSP00000298649	2.188	0.000265	HK1	hexokinase 1 [Source:HGNC Symbol;Acc:4922]
ENSP00000451560	2.185	0.000572	TUBB3	tubulin, beta 3 class III [Source:HGNC Symbol;Acc:20772]
ENSP00000411916	2.182	0.000426	ABAT	4-aminobutyrate aminotransferase [Source:HGNC Symbol;Acc:23]
ENSP00000378729	2.182	0	ALDOC	aldolase C, fructose-bisphosphate [Source:HGNC Symbol;Acc:418]
ENSP00000248437	2.165	0	TUBA4A	tubulin, alpha 4a [Source:HGNC Symbol;Acc:12407]
ENSP00000376066	2.163	0.000219	ATP1A2	ATPase, Na+/K+ transporting, alpha 2 polypeptide [Source:HGNC Symbol;Acc:800]
ENSP00000404740	2.161	0.00048	TUBA4A	tubulin, alpha 4a [Source:HGNC Symbol;Acc:12407]
ENSP00000440874	2.159	0	RTN3	reticulon 3 [Source:HGNC Symbol;Acc:10469]
ENSP00000465623	2.156	0.000263	ALDOC	aldolase C, fructose-bisphosphate [Source:HGNC Symbol;Acc:418]
ENSP00000429947	2.156	0.000086	ATP6V1H	ATPase, H+ transporting, lysosomal 50/57kDa, V1 subunit H [Source:HGNC Symbol;Acc:18303]
ENSP00000282878	2.139	0.000366	RAB3C	RAB3C, member RAS oncogene family [Source:HGNC Symbol;Acc:30269]
ENSP00000264449	2.138	0.001093	ATP8A1	ATPase, aminophospholipid transporter (APLT), class I, type 8A, member 1 [Source:HGNC Symbol;Acc:13531]
ENSP00000309438	2.138	0.002952	PFKM	phosphofructokinase, muscle [Source:HGNC Symbol;Acc:8877]
ENSP00000463328	2.133	0	ATP6V0D1	ATPase, H+ transporting, lysosomal 38kDa, V0 subunit d1 [Source:HGNC Symbol;Acc:13724]
ENSP00000376932	2.12	0.000109	SYT1	synaptotagmin I [Source:HGNC Symbol;Acc:11509]
ENSP00000277865	2.117	0.000967	GLUD1	glutamate dehydrogenase 1 [Source:HGNC Symbol;Acc:4335]
ENSP00000336850	2.097	0	RAB6A	RAB6A, member RAS oncogene family [Source:HGNC Symbol;Acc:9786]
ENSP00000470627	2.094	0.000318	TUBB4A	tubulin, beta 4A class IVa [Source:HGNC Symbol;Acc:20774]
ENSP00000333994	2.087	0.000027	HBB	hemoglobin, beta [Source:HGNC Symbol;Acc:4827]
ENSP00000382982	2.075	0	TUBA3C	tubulin, alpha 3c [Source:HGNC Symbol;Acc:12408]
ENSP00000295156	2.069	0.002282	VSNL1	visinin-like 1 [Source:HGNC Symbol;Acc:12722]
ENSP00000470986	2.065	0	PGK1	phosphoglycerate kinase 1 [Source:HGNC Symbol;Acc:8896]
ENSP00000450268	2.064	0	TUBA1A	tubulin, alpha 1a [Source:HGNC Symbol;Acc:20766]
ENSP00000419921	2.058	0.001039	GPM6B	glycoprotein M6B [Source:HGNC Symbol;Acc:4461]
ENSP00000263233	2.05	0.007799	SYP	synaptophysin [Source:HGNC Symbol;Acc:11506]
ENSP00000449748	2.049	0	TUBA1B	tubulin, alpha 1b [Source:HGNC Symbol;Acc:18809]
ENSP00000406473	2.045	0.001256	PDHA1	pyruvate dehydrogenase (lipoamide) alpha 1 [Source:HGNC Symbol;Acc:8806]
ENSP00000376174	2.045	0.001258	RAN	RAN, member RAS oncogene family [Source:HGNC Symbol;Acc:9846]
ENSP00000356237	2.043	0.000809	SYT2	synaptotagmin II [Source:HGNC Symbol;Acc:11510]
ENSP00000348062	2.041	0.00284	PDHA1	pyruvate dehydrogenase (lipoamide) alpha 1 [Source:HGNC Symbol;Acc:8806]
ENSP00000380899	2.037	0.000423	HBA1	hemoglobin, alpha 1 [Source:HGNC Symbol;Acc:4823]
ENSP00000324105	2.035	0.001934	ENO3	enolase 3 (beta, muscle) [Source:HGNC Symbol;Acc:3354]
ENSP00000313567	2.033	0.000007	CYFIP2	cytoplasmic FMR1 interacting protein 2 [Source:HGNC Symbol;Acc:13760]
ENSP00000410829	2.028	0.001829	TUBB	tubulin, beta class I [Source:HGNC Symbol;Acc:20778]
ENSP00000471490	2.024	0.001326	ENO2	enolase 2 (gamma, neuronal) [Source:HGNC Symbol;Acc:3353]

ENSP00000340903	2.022	0.000166	RTN3	reticulon 3 [Source:HGNC Symbol;Acc:10469]
ENSP00000457610	2.018	0.001334	TUBB8	tubulin, beta 8 class VIII [Source:HGNC Symbol;Acc:20773]
ENSP00000477306	1.989	0	TUBA8	tubulin, alpha 8 [Source:HGNC Symbol;Acc:12410]
ENSP00000354451	1.984	0.001359	IQGAP3	IQ motif containing GTPase activating protein 3 [Source:HGNC Symbol;Acc:20669]
ENSP00000452001	1.979	0.002354	TUBB3	Tubulin beta-3 chain [Source:UniProtKB/Swiss-Prot;Acc:Q13509]
ENSP00000476090	1.976	0.003964	BASP1	brain abundant, membrane attached signal protein 1 [Source:HGNC Symbol;Acc:957]
ENSP00000430682	1.968	0.000083	ATP6V1B2	ATPase, H+ transporting, lysosomal 56/58kDa, V1 subunit B2 [Source:HGNC Symbol;Acc:854]
ENSP00000226253	1.967	0	ALDOC	aldolase C, fructose-bisphosphate [Source:HGNC Symbol;Acc:418]
ENSP00000399685	1.958	0.00097	SEPT5	septin 5 [Source:HGNC Symbol;Acc:9164]
ENSP00000307900	1.948	0.007091	GLUL	glutamate-ammonia ligase [Source:HGNC Symbol;Acc:4341]
ENSP00000305995	1.93	0	PGK2	phosphoglycerate kinase 2 [Source:HGNC Symbol;Acc:8898]
ENSP00000457238	1.929	0.002006	GNAO1	guanine nucleotide binding protein (G protein), alpha activating activity polypeptide O [Source:HGNC Symbol;Acc:4389]
ENSP00000391905	1.911	0.000459	PPP2R1A	protein phosphatase 2, regulatory subunit A, alpha [Source:HGNC Symbol;Acc:9302]
ENSP00000262030	1.91	0	ATP5B	ATP synthase, H+ transporting, mitochondrial F1 complex, beta polypeptide [Source:HGNC Symbol;Acc:830]
ENSP00000444708	1.904	0.000033	PGK1	phosphoglycerate kinase 1 [Source:HGNC Symbol;Acc:8896]
ENSP00000320580	1.89	0.003994	PPP3CA	protein phosphatase 3, catalytic subunit, alpha isozyme [Source:HGNC Symbol;Acc:9314]
ENSP00000382694	1.879	0.007404	ATP6V1E1	ATPase, H+ transporting, lysosomal 31kDa, V1 subunit E1 [Source:HGNC Symbol;Acc:857]
ENSP00000340466	1.873	0.001984	GANAB	glucosidase, alpha; neutral AB [Source:HGNC Symbol;Acc:4138]
ENSP00000366650	1.872	0.000002	PYGM	phosphorylase, glycogen, muscle [Source:HGNC Symbol;Acc:9726]
ENSP00000339001	1.867	0	TUBB	tubulin, beta class I [Source:HGNC Symbol;Acc:20778]
ENSP00000247461	1.863	0	CANX	calnexin [Source:HGNC Symbol;Acc:1473]
ENSP00000349238	1.859	0.008366	CCDC88B	coiled-coil domain containing 88B [Source:HGNC Symbol;Acc:26757]
ENSP00000307241	1.854	0.007215	PDHB	pyruvate dehydrogenase (lipoamide) beta [Source:HGNC Symbol;Acc:8808]
ENSP00000446130	1.852	0.001408	PCBP2	poly(rC) binding protein 2 [Source:HGNC Symbol;Acc:8648]
ENSP00000054666	1.848	0	VAMP3	vesicle-associated membrane protein 3 [Source:HGNC Symbol;Acc:12644]
ENSP00000427954	1.845	0.002964	DPYSL2	dihydropyrimidinase-like 2 [Source:HGNC Symbol;Acc:3014]
ENSP00000401317	1.839	0.000003	TUBB	tubulin, beta class I [Source:HGNC Symbol;Acc:20778]
ENSP00000327070	1.826	0.000002	MDH2	malate dehydrogenase 2, NAD (mitochondrial) [Source:HGNC Symbol;Acc:6971]
ENSP00000468777	1.822	0.002244	TUBB6	tubulin, beta 6 class V [Source:HGNC Symbol;Acc:20776]
ENSP00000424063	1.821	0	CANX	calnexin [Source:HGNC Symbol;Acc:1473]
ENSP00000409667	1.806	0.009891	SYNJ1	synaptojanin 1 [Source:HGNC Symbol;Acc:11503]
ENSP00000431630	1.798	0	AP2A2	adaptor-related protein complex 2, alpha 2 subunit [Source:HGNC Symbol;Acc:562]
ENSP00000296402	1.787	0	CAMK2D	calcium/calmodulin-dependent protein kinase II delta [Source:HGNC Symbol;Acc:1462]
ENSP00000408649	1.77	0.000126	MDH2	malate dehydrogenase 2, NAD (mitochondrial) [Source:HGNC Symbol;Acc:6971]
ENSP00000436603	1.766	0.001567	IQGAP3	IQ motif containing GTPase activating protein 3 [Source:HGNC Symbol;Acc:20669]
ENSP00000282050	1.759	0.000027	ATP5A1	ATP synthase, H+ transporting, mitochondrial F1 complex, alpha subunit 1, cardiac muscle [Source:HGNC Symbol;Acc:823]
ENSP00000344106	1.756	0.000001	RTN3	reticulon 3 [Source:HGNC Symbol;Acc:10469]
ENSP00000285208	1.736	0.003903	RAB6B	RAB6B, member RAS oncogene family [Source:HGNC Symbol;Acc:14902]
ENSP00000300289	1.725	0	PDIA3	protein disulfide isomerase family A, member 3 [Source:HGNC Symbol;Acc:4606]
ENSP00000450712	1.719	0.000007	HSP90AA1	heat shock protein 90kDa alpha (cytosolic), class A member 1 [Source:HGNC Symbol;Acc:5253]
ENSP00000403778	1.702	0.008924	RAC2	ras-related C3 botulinum toxin substrate 2 (rho family, small GTP binding protein Rac2) [Source:HGNC Symbol;Acc:9802]
ENSP00000438260	1.69	0	PDIA3	protein disulfide isomerase family A, member 3 [Source:HGNC Symbol;Acc:4606]
ENSP00000344868	1.674	0	SEPT7	septin 7 [Source:HGNC Symbol;Acc:1717]
ENSP00000423444	1.673	0.006902	MAP1B	microtubule-associated protein 1B [Source:HGNC Symbol;Acc:6836]
ENSP00000396127	1.66	0.000001	RAN	RAN, member RAS oncogene family [Source:HGNC Symbol;Acc:9846]
ENSP00000296755	1.654	0.003005	MAP1B	microtubule-associated protein 1B [Source:HGNC Symbol;Acc:6836]
ENSP00000446489	1.643	0	ATP5B	ATP synthase, H+ transporting, mitochondrial F1 complex, beta polypeptide [Source:HGNC Symbol;Acc:830]
ENSP00000249071	1.634	0	RAC2	ras-related C3 botulinum toxin substrate 2 (rho family, small GTP binding protein Rac2) [Source:HGNC Symbol;Acc:9802]
ENSP00000327694	1.632	0	AP2A2	adaptor-related protein complex 2, alpha 2 subunit [Source:HGNC Symbol;Acc:562]
ENSP00000402551	1.632	0.007464	OTUB1	OTU domain, ubiquitin aldehyde binding 1 [Source:HGNC Symbol;Acc:23077]
ENSP00000164139	1.622	0.000863	PYGM	phosphorylase, glycogen, muscle [Source:HGNC Symbol;Acc:9726]
ENSP00000273047	1.621	0.000329	RAB5A	RAB5A, member RAS oncogene family [Source:HGNC Symbol;Acc:9783]
ENSP00000431117	1.606	0.000007	DPYSL2	dihydropyrimidinase-like 2 [Source:HGNC Symbol;Acc:3014]
ENSP00000448725	1.603	0.000001	TUBA1A	tubulin, alpha 1a [Source:HGNC Symbol;Acc:20766]
ENSP00000336799	1.574	0	TUBA1B	tubulin, alpha 1b [Source:HGNC Symbol;Acc:18809]
ENSP00000229264	1.548	0.000087	GNB3	guanine nucleotide binding protein (G protein), beta polypeptide 3 [Source:HGNC Symbol;Acc:4400]
ENSP00000324173	1.533	0	HSPA5	heat shock 70kDa protein 5 (glucose-regulated protein, 78kDa) [Source:HGNC Symbol;Acc:5238]
ENSP00000428009	1.479	0.000002	CYFIP2	cytoplasmic FMR1 interacting protein 2 [Source:HGNC Symbol;Acc:13760]
ENSP00000301071	1.479	0	TUBA1A	tubulin, alpha 1a [Source:HGNC Symbol;Acc:20766]

ENSP00000465477	1.472	0.000095	ATP5A1	ATP synthase, H+ transporting, mitochondrial F1 complex, alpha subunit 1, cardiac muscle [Source:HGNC Symbol;Acc:823]
ENSP00000414479	1.457	0	TUBB	tubulin, beta class I [Source:HGNC Symbol;Acc:20778]
ENSP00000348965	1.409	0	DYNC1H1	dynein, cytoplasmic 1, heavy chain 1 [Source:HGNC Symbol;Acc:2961]
ENSP00000262325	1.406	0	AP2B1	adaptor-related protein complex 2, beta 1 subunit [Source:HGNC Symbol;Acc:563]
ENSP00000475018	1.401	0.000111	AP2B1	adaptor-related protein complex 2, beta 1 subunit [Source:HGNC Symbol;Acc:563]
ENSP00000371894	1.395	0.002005	AP2M1	adaptor-related protein complex 2, mu 1 subunit [Source:HGNC Symbol;Acc:564]
ENSP00000297283	1.391	0.000002	PGAM2	phosphoglycerate mutase 2 (muscle) [Source:HGNC Symbol;Acc:8889]
ENSP00000295598	1.384	0	ATP1A1	ATPase, Na+/K+ transporting, alpha 1 polypeptide [Source:HGNC Symbol;Acc:799]
ENSP00000287226	1.361	0.005666	NPTN	neuroplastin [Source:HGNC Symbol;Acc:17867]
ENSP00000264893	1.353	0.002426	SEPT11	septin 11 [Source:HGNC Symbol;Acc:25589]
ENSP00000269122	1.328	0	CLTC	clathrin, heavy chain (Hc) [Source:HGNC Symbol;Acc:2092]
ENSP00000227378	1.322	0	HSPA8	heat shock 70kDa protein 8 [Source:HGNC Symbol;Acc:5241]
ENSP00000400022	1.304	0.000016	AP1B1	adaptor-related protein complex 1, beta 1 subunit [Source:HGNC Symbol;Acc:554]
ENSP00000376763	1.292	0	CLTC	clathrin, heavy chain (Hc) [Source:HGNC Symbol;Acc:2092]
ENSP00000393340	1.285	0	TUBB	tubulin, beta class I [Source:HGNC Symbol;Acc:20778]
ENSP00000362946	1.255	0.001764	RAB14	RAB14, member RAS oncogene family [Source:HGNC Symbol;Acc:16524]
ENSP00000248975	1.255	0.002949	YWHAH	tyrosine 3-monooxygenase/tryptophan 5-monooxygenase activation protein, eta [Source:HGNC Symbol;Acc:12853]
ENSP00000394071	1.233	0.003021	GDI1	GDP dissociation inhibitor 1 [Source:HGNC Symbol;Acc:4226]
ENSP00000300161	1.224	0.000001	YWHAB	tyrosine 3-monooxygenase/tryptophan 5-monooxygenase activation protein, beta [Source:HGNC Symbol;Acc:12849]
ENSP00000229277	1.22	0.000003	ENO2	enolase 2 (gamma, neuronal) [Source:HGNC Symbol;Acc:3353]
ENSP00000449325	1.22	0	TUBA1B	tubulin, alpha 1b [Source:HGNC Symbol;Acc:18809]
ENSP00000258737	1.171	0.000011	RAC1	ras-related C3 botulinum toxin substrate 1 (rho family, small GTP binding protein Rac1) [Source:HGNC Symbol;Acc:9801]
ENSP00000361930	1.155	0.000729	YWHAB	tyrosine 3-monooxygenase/tryptophan 5-monooxygenase activation protein, beta [Source:HGNC Symbol;Acc:12849]
ENSP00000306330	1.142	0	YWHAG	tyrosine 3-monooxygenase/tryptophan 5-monooxygenase activation protein, gamma [Source:HGNC Symbol;Acc:12852]
ENSP00000430636	1.088	0	ENO3	enolase 3 (beta, muscle) [Source:HGNC Symbol;Acc:3354]
ENSP00000359991	1.066	0.002003	PGAM1	phosphoglycerate mutase 1 (brain) [Source:HGNC Symbol;Acc:8888]
ENSP00000216281	1.002	0	HSP90AA1	heat shock protein 90kDa alpha (cytosolic), class A member 1 [Source:HGNC Symbol;Acc:5253]
ENSP00000265062	0.984	0.000028	RAB7A	RAB7A, member RAS oncogene family [Source:HGNC Symbol;Acc:9788]
ENSP00000396189	0.955	0.006064	HSP90AA1	heat shock protein 90kDa alpha (cytosolic), class A member 1 [Source:HGNC Symbol;Acc:5253]
ENSP00000318575	0.938	0.000858	TUBA8	tubulin, alpha 8 [Source:HGNC Symbol;Acc:12410]
ENSP00000216962	0.928	0.000037	PYGB	phosphorylase, glycogen; brain [Source:HGNC Symbol;Acc:9723]
ENSP00000377470	0.911	0.000012	CNP	2',3'-cyclic nucleotide 3' phosphodiesterase [Source:HGNC Symbol;Acc:2158]
ENSP00000399904	0.9	0	GNB2	guanine nucleotide binding protein (G protein), beta polypeptide 2 [Source:HGNC Symbol;Acc:4398]
ENSP00000299767	0.873	0.000014	HSP90B1	heat shock protein 90kDa beta (Grp94), member 1 [Source:HGNC Symbol;Acc:12028]
ENSP00000232564	0.865	0.000069	GNB4	guanine nucleotide binding protein (G protein), beta polypeptide 4 [Source:HGNC Symbol;Acc:20731]
ENSP00000290378	0.86	0.000015	ACTC1	actin, alpha, cardiac muscle 1 [Source:HGNC Symbol;Acc:143]
ENSP00000325875	0.848	0	HSP90AB1	heat shock protein 90kDa alpha (cytosolic), class B member 1 [Source:HGNC Symbol;Acc:5258]
ENSP00000264710	0.819	0.007666	RAB10	RAB10, member RAS oncogene family [Source:HGNC Symbol;Acc:9759]
ENSP00000343599	0.789	0.000469	ATP2B1	ATPase, Ca++ transporting, plasma membrane 1 [Source:HGNC Symbol;Acc:814]
ENSP00000460932	0.692	0.000022	ATP2B3	ATPase, Ca++ transporting, plasma membrane 3 [Source:HGNC Symbol;Acc:816]
ENSP00000358508	0.687	0	ATP1A1	ATPase, Na+/K+ transporting, alpha 1 polypeptide [Source:HGNC Symbol;Acc:799]
ENSP00000398239	0.681	0.000006	ACTA2	actin, alpha 2, smooth muscle, aorta [Source:HGNC Symbol;Acc:130]
ENSP00000238081	0.629	0.001476	YWHAQ	tyrosine 3-monooxygenase/tryptophan 5-monooxygenase activation protein, theta [Source:HGNC Symbol;Acc:12854]
ENSP00000261173	0.617	0.000115	ATP2B1	ATPase, Ca++ transporting, plasma membrane 1 [Source:HGNC Symbol;Acc:814]
ENSP00000217182	0.535	0.000253	EEF1A2	eukaryotic translation elongation factor 1 alpha 2 [Source:HGNC Symbol;Acc:3192]
ENSP00000459119	0.517	0	ACTG1	actin, gamma 1 [Source:HGNC Symbol;Acc:144]
ENSP00000224784	0.392	0.000001	ACTA2	actin, alpha 2, smooth muscle, aorta [Source:HGNC Symbol;Acc:130]

Supplementary table S3. A table of differentially expressed proteins in SBE-MSC-MV.

Protein ID	LogFold Change	FDR	Gene Symbol	Description
ENSP00000261205	5.195	0	SYT1	synaptotagmin I [Source:HGNC Symbol;Acc:11509]
ENSP00000345680	4.781	0	DNM1	dynamin 1 [Source:HGNC Symbol;Acc:2972]
ENSP00000305152	4.754	0	PLP1	proteolipid protein 1 [Source:HGNC Symbol;Acc:9086]
ENSP00000302397	4.746	0	ATP1A3	ATPase, Na+/K+ transporting, alpha 3 polypeptide [Source:HGNC Symbol;Acc:801]
ENSP00000419225	4.271	0	DNM1	dynamin 1 [Source:HGNC Symbol;Acc:2972]
ENSP00000216254	4.162	0	ACO2	aconitase 2, mitochondrial [Source:HGNC Symbol;Acc:118]
ENSP00000295987	4.071	0	SYN1	synapsin I [Source:HGNC Symbol;Acc:11494]
ENSP00000362396	4.046	0	STXBP1	syntaxin binding protein 1 [Source:HGNC Symbol;Acc:11444]
ENSP00000264071	3.976	0	TUBB4A	tubulin, beta 4A class IVa [Source:HGNC Symbol;Acc:20774]
ENSP00000469129	3.961	0	ATP1A3	ATPase, Na+/K+ transporting, alpha 3 polypeptide [Source:HGNC Symbol;Acc:801]
ENSP00000380104	3.813	0	HBE1	hemoglobin, epsilon 1 [Source:HGNC Symbol;Acc:4830]
ENSP00000292896	3.785	0	HBE1	hemoglobin, epsilon 1 [Source:HGNC Symbol;Acc:4830]
ENSP00000342951	3.768	0	ATP6V0A1	ATPase, H+ transporting, lysosomal V0 subunit a1 [Source:HGNC Symbol;Acc:865]
ENSP00000362340	3.764	0	NCDN	neurochondrin [Source:HGNC Symbol;Acc:17597]
ENSP00000299198	3.754	0	CKB	creatine kinase, brain [Source:HGNC Symbol;Acc:1991]
ENSP00000455899	3.752	0	STX1B	syntaxin 1B [Source:HGNC Symbol;Acc:18539]
ENSP00000261160	3.739	0	CNTN1	contactin 1 [Source:HGNC Symbol;Acc:2171]
ENSP00000454728	3.699	0	GNAO1	guanine nucleotide binding protein (G protein), alpha activating activity polypeptide O [Source:HGNC Symbol;Acc:4389]
ENSP00000225282	3.696	0	NSF	N-ethylmaleimide-sensitive factor [Source:HGNC Symbol;Acc:8016]
ENSP00000222286	3.688	0	GAPDHS	glyceraldehyde-3-phosphate dehydrogenase, spermatogenic [Source:HGNC Symbol;Acc:24864]
ENSP00000396061	3.67	0	TUBA4A	tubulin, alpha 4a [Source:HGNC Symbol;Acc:12407]
ENSP00000302777	3.662	0	TUBB3	Tubulin beta-3 chain [Source:UniProtKB/Swiss-Prot;Acc:Q13509]
ENSP00000381293	3.59	0	NSF	N-ethylmaleimide-sensitive factor [Source:HGNC Symbol;Acc:8016]
ENSP00000215095	3.524	0	STX1B	syntaxin 1B [Source:HGNC Symbol;Acc:18539]
ENSP00000462223	3.482	0	MBP	myelin basic protein [Source:HGNC Symbol;Acc:6925]
ENSP00000354490	3.473	0	ATP1A2	ATPase, Na+/K+ transporting, alpha 2 polypeptide [Source:HGNC Symbol;Acc:800]
ENSP00000264649	3.469	0	ATP6V0A1	ATPase, H+ transporting, lysosomal V0 subunit a1 [Source:HGNC Symbol;Acc:865]
ENSP00000429701	3.465	0	DNM3	dynamin 3 [Source:HGNC Symbol;Acc:29125]
ENSP00000382981	3.325	0	EPB41L3	erythrocyte membrane protein band 4.1-like 3 [Source:HGNC Symbol;Acc:3380]
ENSP00000322234	3.324	0	SYNJ1	synaptojanin 1 [Source:HGNC Symbol;Acc:11503]
ENSP00000476705	3.319	0	ATP1A3	ATPase, Na+/K+ transporting, alpha 3 polypeptide [Source:HGNC Symbol;Acc:801]
ENSP00000222256	3.319	0	RAB3A	RAB3A, member RAS oncogene family [Source:HGNC Symbol;Acc:9777]
ENSP00000259818	3.283	0	TUBB2B	tubulin, beta 2B class IIb [Source:HGNC Symbol;Acc:30829]
ENSP00000222120	3.268	0	RAB3D	RAB3D, member RAS oncogene family [Source:HGNC Symbol;Acc:9779]
ENSP00000362399	3.265	0	STXBP1	syntaxin binding protein 1 [Source:HGNC Symbol;Acc:11444]
ENSP00000369602	3.264	0	HBG2	hemoglobin, gamma G [Source:HGNC Symbol;Acc:4832]
ENSP00000348394	3.264	0	NCDN	neurochondrin [Source:HGNC Symbol;Acc:17597]
ENSP00000218548	3.221	0	ATP12A	ATPase, H+/K+ transporting, nongastric, alpha polypeptide [Source:HGNC Symbol;Acc:13816]
ENSP00000311186	3.211	0	ATP2A2	ATPase, Ca++ transporting, cardiac muscle, slow twitch 2 [Source:HGNC Symbol;Acc:812]
ENSP00000356789	3.181	0	ATP1B1	ATPase, Na+/K+ transporting, beta 1 polypeptide [Source:HGNC Symbol;Acc:804]
ENSP00000449415	3.177	0	SYT1	synaptotagmin I [Source:HGNC Symbol;Acc:11509]
ENSP00000356787	3.157	0	ATP1B1	ATPase, Na+/K+ transporting, beta 1 polypeptide [Source:HGNC Symbol;Acc:804]
ENSP00000307341	3.147	0	SNAP25	synaptosomal-associated protein, 25kDa [Source:HGNC Symbol;Acc:11132]
ENSP00000341138	3.121	0	EPB41L3	erythrocyte membrane protein band 4.1-like 3 [Source:HGNC Symbol;Acc:3380]
ENSP00000254976	3.072	0	SNAP25	synaptosomal-associated protein, 25kDa [Source:HGNC Symbol;Acc:11132]
ENSP00000292901	3.054	0	HBD	hemoglobin, delta [Source:HGNC Symbol;Acc:4829]
ENSP00000380987	3.043	0	CRMP1	collapsin response mediator protein 1 [Source:HGNC Symbol;Acc:2365]
ENSP00000262494	3.038	0	GNAO1	guanine nucleotide binding protein (G protein), alpha activating activity polypeptide O [Source:HGNC Symbol;Acc:4389]
ENSP00000449426	3.03	0	PFKM	phosphofructokinase, muscle [Source:HGNC Symbol;Acc:8877]
ENSP00000352398	3.018	0	HK1	hexokinase 1 [Source:HGNC Symbol;Acc:4922]
ENSP00000466696	3.015	0	NCAM1	neural cell adhesion molecule 1 [Source:HGNC Symbol;Acc:7656]
ENSP00000358211	2.984	0	HSPA12A	heat shock 70kDa protein 12A [Source:HGNC Symbol;Acc:19022]
ENSP00000309539	2.98	0	DPYSL2	dihydropyrimidinase-like 2 [Source:HGNC Symbol;Acc:3014]
ENSP00000251595	2.98	0	HBA2	hemoglobin, alpha 2 [Source:HGNC Symbol;Acc:4824]
ENSP00000318472	2.934	0	NCAM1	neural cell adhesion molecule 1 [Source:HGNC Symbol;Acc:7656]
ENSP00000448653	2.893	0	CNTN1	contactin 1 [Source:HGNC Symbol;Acc:2171]
ENSP00000262493	2.889	0	GNAO1	guanine nucleotide binding protein (G protein), alpha activating activity polypeptide O [Source:HGNC Symbol;Acc:4389]
ENSP00000324804	2.887	0	PPP2R1A	protein phosphatase 2, regulatory subunit A, alpha [Source:HGNC Symbol;Acc:9302]
ENSP00000348273	2.882	0	MBP	myelin basic protein [Source:HGNC Symbol;Acc:6925]

ENSP00000273398	2.88	0	ATP6V1A	ATPase, H+ transporting, lysosomal 70kDa, V1 subunit A [Source:HGNC Symbol;Acc:851]
ENSP00000380267	2.879	0	ATP2B2	ATPase, Ca++ transporting, plasma membrane 2 [Source:HGNC Symbol;Acc:815]
ENSP00000369654	2.875	0	HBD	hemoglobin, delta [Source:HGNC Symbol;Acc:4829]
ENSP00000309438	2.855	0.000002	PFKM	phosphofructokinase, muscle [Source:HGNC Symbol;Acc:8877]
ENSP00000375938	2.835	0	TUBA4A	tubulin, alpha 4a [Source:HGNC Symbol;Acc:12407]
ENSP00000298649	2.834	0	HK1	hexokinase 1 [Source:HGNC Symbol;Acc:4922]
ENSP00000379769	2.824	0	ACO2	aconitase 2, mitochondrial [Source:HGNC Symbol;Acc:118]
ENSP00000321606	2.814	0	CRMP1	collapsin response mediator protein 1 [Source:HGNC Symbol;Acc:2365]
ENSP00000222812	2.813	0.000001	STX1A	syntaxin 1A (brain) [Source:HGNC Symbol;Acc:11433]
ENSP00000451617	2.8	0	TUBB3	Tubulin beta-3 chain [Source:UniProtKB/Swiss-Prot;Acc:Q13509]
ENSP00000413649	2.796	0	SYNJ1	synaptojanin 1 [Source:HGNC Symbol;Acc:11503]
ENSP00000322421	2.789	0	HBA1	hemoglobin, alpha 1 [Source:HGNC Symbol;Acc:4823]
ENSP00000283269	2.778	0.000002	CADPS	Ca++-dependent secretion activator [Source:HGNC Symbol;Acc:1426]
ENSP00000421578	2.753	0	GPM6A	glycoprotein M6A [Source:HGNC Symbol;Acc:4460]
ENSP00000471581	2.749	0	ATP1A3	ATPase, Na+/K+ transporting, alpha 3 polypeptide [Source:HGNC Symbol;Acc:801]
ENSP00000452166	2.748	0	TUBB3	Tubulin beta-3 chain [Source:UniProtKB/Swiss-Prot;Acc:Q13509]
ENSP00000351155	2.745	0	ATL1	atlastin GTPase 1 [Source:HGNC Symbol;Acc:11231]
ENSP00000463016	2.735	0.000008	SV2A	synaptic vesicle glycoprotein 2A [Source:HGNC Symbol;Acc:20566]
ENSP00000313164	2.716	0.000005	DNM2	dynamin 2 [Source:HGNC Symbol;Acc:2974]
ENSP00000253413	2.715	0.000002	ATP6V1E1	ATPase, H+ transporting, lysosomal 31kDa, V1 subunit E1 [Source:HGNC Symbol;Acc:857]
ENSP00000357060	2.7	0	ATP1A4	ATPase, Na+/K+ transporting, alpha 4 polypeptide [Source:HGNC Symbol;Acc:14073]
ENSP00000369703	2.698	0	TUBB2A	tubulin, beta 2A class IIa [Source:HGNC Symbol;Acc:12412]
ENSP00000472335	2.695	0.000001	RAB3A	RAB3A, member RAS oncogene family [Source:HGNC Symbol;Acc:9777]
ENSP00000387694	2.691	0	SLC12A5	solute carrier family 12 (potassium/chloride transporter), member 5 [Source:HGNC Symbol;Acc:13818]
ENSP00000343206	2.691	0	SYN1	synapsin I [Source:HGNC Symbol;Acc:11494]
ENSP00000346522	2.69	0.000002	ATL1	atlastin GTPase 1 [Source:HGNC Symbol;Acc:11231]
ENSP00000459646	2.68	0.000001	NSF	N-ethylmaleimide-sensitive factor [Source:HGNC Symbol;Acc:8016]
ENSP00000358141	2.669	0.000003	SV2A	synaptic vesicle glycoprotein 2A [Source:HGNC Symbol;Acc:20566]
ENSP00000386192	2.666	0	DNM2	dynamin 2 [Source:HGNC Symbol;Acc:2974]
ENSP00000439874	2.651	0	ATP6V1A	ATPase, H+ transporting, lysosomal 70kDa, V1 subunit A [Source:HGNC Symbol;Acc:851]
ENSP00000243964	2.645	0.000011	SLC12A5	solute carrier family 12 (potassium/chloride transporter), member 5 [Source:HGNC Symbol;Acc:13818]
ENSP00000324172	2.641	0	ATP2B2	ATPase, Ca++ transporting, plasma membrane 2 [Source:HGNC Symbol;Acc:815]
ENSP00000299518	2.64	0.000007	IDH3A	isocitrate dehydrogenase 3 (NAD+) alpha [Source:HGNC Symbol;Acc:5384]
ENSP00000362413	2.627	0	PGK1	phosphoglycerate kinase 1 [Source:HGNC Symbol;Acc:8896]
ENSP00000276390	2.589	0	ATP6V1B2	ATPase, H+ transporting, lysosomal 56/58kDa, V1 subunit B2 [Source:HGNC Symbol;Acc:854]
ENSP00000217133	2.567	0	TUBB1	tubulin, beta 1 class VI [Source:HGNC Symbol;Acc:16257]
ENSP00000318697	2.566	0	TUBB6	tubulin, beta 6 class V [Source:HGNC Symbol;Acc:20776]
ENSP00000328455	2.565	0.000005	LSAMP	limbic system-associated membrane protein [Source:HGNC Symbol;Acc:6705]
ENSP00000405750	2.563	0.000001	PLP1	proteolipid protein 1 [Source:HGNC Symbol;Acc:9086]
ENSP00000471490	2.545	0.000008	ENO2	enolase 2 (gamma, neuronal) [Source:HGNC Symbol;Acc:3353]
ENSP00000320295	2.534	0.000002	TUBB3	Tubulin beta-3 chain [Source:UniProtKB/Swiss-Prot;Acc:Q13509]
ENSP00000268251	2.533	0.000003	ABAT	4-aminobutyrate aminotransferase [Source:HGNC Symbol;Acc:23]
ENSP00000458221	2.528	0.000006	NSF	N-ethylmaleimide-sensitive factor [Source:HGNC Symbol;Acc:8016]
ENSP00000467399	2.517	0	VAMP2	vesicle-associated membrane protein 2 (synaptobrevin 2) [Source:HGNC Symbol;Acc:12643]
ENSP00000353174	2.516	0.000002	RYR2	ryanodine receptor 2 (cardiac) [Source:HGNC Symbol;Acc:10484]
ENSP00000470571	2.51	0.000003	SYP	synaptophysin [Source:HGNC Symbol;Acc:11506]
ENSP00000403335	2.48	0.00001	PLP1	proteolipid protein 1 [Source:HGNC Symbol;Acc:9086]
ENSP00000451378	2.475	0.000005	TUBB3	Tubulin beta-3 chain [Source:UniProtKB/Swiss-Prot;Acc:Q13509]
ENSP00000280187	2.464	0.00005	GPM6A	glycoprotein M6A [Source:HGNC Symbol;Acc:4460]
ENSP00000427985	2.458	0	DPYSL2	dihydropyrimidinase-like 2 [Source:HGNC Symbol;Acc:3014]
ENSP00000376932	2.447	0.000006	SYT1	synaptotagmin I [Source:HGNC Symbol;Acc:11509]
ENSP00000292807	2.436	0.000053	AP2M1	adaptor-related protein complex 2, mu 1 subunit [Source:HGNC Symbol;Acc:564]
ENSP00000465154	2.428	0.000002	NCAM1	neural cell adhesion molecule 1 [Source:HGNC Symbol;Acc:7656]
ENSP00000301387	2.427	0.000383	ATP2A3	ATPase, Ca++ transporting, ubiquitous [Source:HGNC Symbol;Acc:813]
ENSP00000341289	2.419	0	TUBB4B	tubulin, beta 4B class IVb [Source:HGNC Symbol;Acc:20771]
ENSP00000409667	2.405	0.000176	SYNJ1	synaptojanin 1 [Source:HGNC Symbol;Acc:11503]
ENSP00000313567	2.404	0	CYFIP2	cytoplasmic FMR1 interacting protein 2 [Source:HGNC Symbol;Acc:13760]
ENSP00000378872	2.383	0.000002	ATP2A2	ATPase, Ca++ transporting, cardiac muscle, slow twitch 2 [Source:HGNC Symbol;Acc:812]
ENSP00000398320	2.38	0.00003	GLUL	glutamate-ammonia ligase [Source:HGNC Symbol;Acc:4341]
ENSP00000411916	2.378	0.000048	ABAT	4-aminobutyrate aminotransferase [Source:HGNC Symbol;Acc:23]
ENSP00000387506	2.377	0.000021	IDH3A	isocitrate dehydrogenase 3 (NAD+) alpha [Source:HGNC Symbol;Acc:5384]
ENSP00000411705	2.375	0	ATP1A2	ATPase, Na+/K+ transporting, alpha 2 polypeptide [Source:HGNC Symbol;Acc:800]

ENSP00000346545	2.369	0.000119	MBP	myelin basic protein [Source:HGNC Symbol;Acc:6925]
ENSP00000347359	2.364	0.000002	ATP6V1H	ATPase, H+ transporting, lysosomal 50/57kDa, V1 subunit H [Source:HGNC Symbol;Acc:18303]
ENSP00000262623	2.35	0	ATP4A	ATPase, H+/K+ exchanging, alpha polypeptide [Source:HGNC Symbol;Acc:819]
ENSP00000324105	2.35	0.000005	ENO3	enolase 3 (beta, muscle) [Source:HGNC Symbol;Acc:3354]
ENSP00000362350	2.338	0.000105	NCDN	neurochondrin [Source:HGNC Symbol;Acc:17597]
ENSP00000457610	2.336	0.000019	TUBB8	tubulin, beta 8 class VIII [Source:HGNC Symbol;Acc:20773]
ENSP00000354111	2.314	0.000003	DNAJC5	DnaJ (Hsp40) homolog, subfamily C, member 5 [Source:HGNC Symbol;Acc:16235]
ENSP00000477015	2.302	0.00013	ATP1B1	ATPase, Na+/K+ transporting, beta 1 polypeptide [Source:HGNC Symbol;Acc:804]
ENSP00000429947	2.296	0.00004	ATP6V1H	ATPase, H+ transporting, lysosomal 50/57kDa, V1 subunit H [Source:HGNC Symbol;Acc:18303]
ENSP00000350632	2.291	0.000228	CADPS	Ca++-dependent secretion activator [Source:HGNC Symbol;Acc:1426]
ENSP00000340716	2.288	0.000036	RTN1	reticulon 1 [Source:HGNC Symbol;Acc:10467]
ENSP00000447006	2.281	0.000019	CNTN1	contactin 1 [Source:HGNC Symbol;Acc:2171]
ENSP00000423444	2.279	0.000005	MAP1B	microtubule-associated protein 1B [Source:HGNC Symbol;Acc:6836]
ENSP00000309431	2.279	0.000008	RP11-683L23.1	Tubulin beta-8 chain-like protein LOC260334 [Source:UniProtKB/Swiss-Prot;Acc:A6NNZ2]
ENSP00000430682	2.265	0.000001	ATP6V1B2	ATPase, H+ transporting, lysosomal 56/58kDa, V1 subunit B2 [Source:HGNC Symbol;Acc:854]
ENSP00000385447	2.259	0.00053	SYNGR1	synaptogyrin 1 [Source:HGNC Symbol;Acc:11498]
ENSP00000261793	2.254	0.001023	CAMK2A	calcium/calmodulin-dependent protein kinase II alpha [Source:HGNC Symbol;Acc:1460]
ENSP00000455774	2.252	0.000416	GNAO1	guanine nucleotide binding protein (G protein), alpha activating activity polypeptide O [Source:HGNC Symbol;Acc:4389]
ENSP00000436029	2.251	0.000034	SLC1A2	solute carrier family 1 (glial high affinity glutamate transporter), member 2 [Source:HGNC Symbol;Acc:10940]
ENSP00000434744	2.248	0.000023	DNAJC5	DnaJ (Hsp40) homolog, subfamily C, member 5 [Source:HGNC Symbol;Acc:16235]
ENSP00000295156	2.244	0.000335	VSNL1	visinin-like 1 [Source:HGNC Symbol;Acc:12722]
ENSP00000278379	2.223	0.000002	SLC1A2	solute carrier family 1 (glial high affinity glutamate transporter), member 2 [Source:HGNC Symbol;Acc:10940]
ENSP00000296755	2.222	0.00003	MAP1B	microtubule-associated protein 1B [Source:HGNC Symbol;Acc:6836]
ENSP00000451560	2.221	0.000461	TUBB3	tubulin, beta 3 class III [Source:HGNC Symbol;Acc:20772]
ENSP00000472481	2.217	0	TUBB4A	tubulin, beta 4A class IVa [Source:HGNC Symbol;Acc:20774]
ENSP00000333994	2.21	0.000001	HBB	hemoglobin, beta [Source:HGNC Symbol;Acc:4827]
ENSP00000384014	2.207	0.000453	VSNL1	visinin-like 1 [Source:HGNC Symbol;Acc:12722]
ENSP00000380899	2.192	0.00049	HBA1	hemoglobin, alpha 1 [Source:HGNC Symbol;Acc:4823]
ENSP00000444708	2.19	0.000002	PGK1	phosphoglycerate kinase 1 [Source:HGNC Symbol;Acc:8896]
ENSP00000366225	2.186	0.001517	NAPB	N-ethylmaleimide-sensitive factor attachment protein, beta [Source:HGNC Symbol;Acc:15751]
ENSP00000382696	2.171	0.000021	ATP6V1E1	ATPase, H+ transporting, lysosomal 31kDa, V1 subunit E1 [Source:HGNC Symbol;Acc:857]
ENSP00000410829	2.171	0.001805	TUBB	tubulin, beta class I [Source:HGNC Symbol;Acc:20778]
ENSP00000282878	2.167	0.000499	RAB3C	RAB3C, member RAS oncogene family [Source:HGNC Symbol;Acc:30269]
ENSP00000277865	2.155	0.000779	GLUD1	glutamate dehydrogenase 1 [Source:HGNC Symbol;Acc:4335]
ENSP00000420908	2.148	0.001637	SPOP	speckle-type POZ protein [Source:HGNC Symbol;Acc:11254]
ENSP00000465820	2.144	0.000352	VAMP3	vesicle-associated membrane protein 3 [Source:HGNC Symbol;Acc:12644]
ENSP00000400720	2.14	0.001308	SNAP25	synaptosomal-associated protein, 25kDa [Source:HGNC Symbol;Acc:11132]
ENSP00000449748	2.136	0	TUBA1B	tubulin, alpha 1b [Source:HGNC Symbol;Acc:18809]
ENSP00000366650	2.134	0	PYGM	phosphorylase, glycogen, muscle [Source:HGNC Symbol;Acc:9726]
ENSP00000164139	2.131	0	PYGM	phosphorylase, glycogen, muscle [Source:HGNC Symbol;Acc:9726]
ENSP00000477306	2.126	0	TUBA8	tubulin, alpha 8 [Source:HGNC Symbol;Acc:12410]
ENSP00000248437	2.124	0	TUBA4A	tubulin, alpha 4a [Source:HGNC Symbol;Acc:12407]
ENSP00000240327	2.122	0.000869	SPOP	speckle-type POZ protein [Source:HGNC Symbol;Acc:11254]
ENSP00000340466	2.118	0.000022	GANAB	glucosidase, alpha; neutral AB [Source:HGNC Symbol;Acc:4138]
ENSP00000349238	2.114	0.002092	CCDC88B	coiled-coil domain containing 88B [Source:HGNC Symbol;Acc:26757]
ENSP00000470986	2.101	0	PGK1	phosphoglycerate kinase 1 [Source:HGNC Symbol;Acc:8896]
ENSP00000404740	2.089	0.001047	TUBA4A	tubulin, alpha 4a [Source:HGNC Symbol;Acc:12407]
ENSP00000457238	2.087	0.001227	GNAO1	guanine nucleotide binding protein (G protein), alpha activating activity polypeptide O [Source:HGNC Symbol;Acc:4389]
ENSP00000258682	2.086	0.00002	CAMK2B	calcium/calmodulin-dependent protein kinase II beta [Source:HGNC Symbol;Acc:1461]
ENSP00000226253	2.066	0	ALDOC	aldolase C, fructose-bisphosphate [Source:HGNC Symbol;Acc:418]
ENSP00000382982	2.062	0	TUBA3C	tubulin, alpha 3c [Source:HGNC Symbol;Acc:12408]
ENSP00000427954	2.056	0.005013	DPYSL2	dihydropyrimidinase-like 2 [Source:HGNC Symbol;Acc:3014]
ENSP00000307900	2.053	0.001288	GLUL	glutamate-ammonia ligase [Source:HGNC Symbol;Acc:4341]
ENSP00000264449	2.05	0.002011	ATP8A1	ATPase, aminophospholipid transporter (APLT), class I, type 8A, member 1 [Source:HGNC Symbol;Acc:13531]
ENSP00000431630	2.04	0	AP2A2	adaptor-related protein complex 2, alpha 2 subunit [Source:HGNC Symbol;Acc:562]
ENSP00000305995	2.04	0	PGK2	phosphoglycerate kinase 2 [Source:HGNC Symbol;Acc:8898]
ENSP00000418799	2.036	0	TUBAL3	tubulin, alpha-like 3 [Source:HGNC Symbol;Acc:23534]
ENSP00000234396	2.013	0.005891	ATP6V1B1	ATPase, H+ transporting, lysosomal 56/58kDa, V1 subunit B1 [Source:HGNC Symbol;Acc:853]

ENSP00000378729	2.009	0	ALDOC	aldolase C, fructose-bisphosphate [Source:HGNC Symbol;Acc:418]
ENSP00000452001	2.008	0.007791	TUBB3	Tubulin beta-3 chain [Source:UniProtKB/Swiss-Prot;Acc:Q13509]
ENSP00000314214	2.006	0.002077	VAMP2	vesicle-associated membrane protein 2 (synaptobrevin 2) [Source:HGNC Symbol;Acc:12643]
ENSP00000216442	1.999	0.006891	ATP6V1D	ATPase, H+ transporting, lysosomal 34kDa, V1 subunit D [Source:HGNC Symbol;Acc:13527]
ENSP00000256496	1.998	0.003418	ARL8B	ADP-ribosylation factor-like 8B [Source:HGNC Symbol;Acc:25564]
ENSP00000450538	1.994	0.001403	TUBB3	Tubulin beta-3 chain [Source:UniProtKB/Swiss-Prot;Acc:Q13509]
ENSP00000431117	1.993	0	DPYSL2	dihydropyrimidinase-like 2 [Source:HGNC Symbol;Acc:3014]
ENSP00000330862	1.987	0.003978	OPCML	opioid binding protein/cell adhesion molecule-like [Source:HGNC Symbol;Acc:8143]
ENSP00000342056	1.984	0.001717	CS	citrate synthase [Source:HGNC Symbol;Acc:2422]
ENSP00000327694	1.983	0	AP2A2	adaptor-related protein complex 2, alpha 2 subunit [Source:HGNC Symbol;Acc:562]
ENSP00000393391	1.975	0.003759	PLP1	proteolipid protein 1 [Source:HGNC Symbol;Acc:9086]
ENSP00000470627	1.969	0.005005	TUBB4A	tubulin, beta 4A class IVa [Source:HGNC Symbol;Acc:20774]
ENSP00000376066	1.958	0.008418	ATP1A2	ATPase, Na+/K+ transporting, alpha 2 polypeptide [Source:HGNC Symbol;Acc:800]
ENSP00000391905	1.958	0.000412	PPP2R1A	protein phosphatase 2, regulatory subunit A, alpha [Source:HGNC Symbol;Acc:9302]
ENSP00000370388	1.956	0.004201	DNM1L	dynamin 1-like [Source:HGNC Symbol;Acc:2973]
ENSP00000340903	1.953	0.003668	RTN3	reticulon 3 [Source:HGNC Symbol;Acc:10469]
ENSP00000290949	1.951	0.000088	ATP6V0D1	ATPase, H+ transporting, lysosomal 38kDa, V0 subunit d1 [Source:HGNC Symbol;Acc:13724]
ENSP00000401317	1.949	0.000002	TUBB	tubulin, beta class I [Source:HGNC Symbol;Acc:20778]
ENSP00000448004	1.948	0.002169	CNTN1	contactin 1 [Source:HGNC Symbol;Acc:2171]
ENSP00000249071	1.94	0	RAC2	ras-related C3 botulinum toxin substrate 2 (rho family, small GTP binding protein Rac2) [Source:HGNC Symbol;Acc:9802]
ENSP00000311489	1.939	0.00041	SPTBN2	spectrin, beta, non-erythrocytic 2 [Source:HGNC Symbol;Acc:11276]
ENSP00000267484	1.922	0.006007	RTN1	reticulon 1 [Source:HGNC Symbol;Acc:10467]
ENSP00000363910	1.901	0.005417	OPCML	opioid binding protein/cell adhesion molecule-like [Source:HGNC Symbol;Acc:8143]
ENSP00000447860	1.888	0.00299	CNTN1	contactin 1 [Source:HGNC Symbol;Acc:2171]
ENSP00000458474	1.885	0.009209	HSPA12A	heat shock 70kDa protein 12A [Source:HGNC Symbol;Acc:19022]
ENSP00000463328	1.883	0.000142	ATP6V0D1	ATPase, H+ transporting, lysosomal 38kDa, V0 subunit d1 [Source:HGNC Symbol;Acc:13724]
ENSP00000437971	1.882	0.009973	RTN3	reticulon 3 [Source:HGNC Symbol;Acc:10469]
ENSP00000384144	1.874	0.00918	HYOU1	hypoxia up-regulated 1 [Source:HGNC Symbol;Acc:16931]
ENSP00000428009	1.873	0	CYFIP2	cytoplasmic FMR1 interacting protein 2 [Source:HGNC Symbol;Acc:13760]
ENSP00000293590	1.869	0.007995	FMNL3	formin-like 3 [Source:HGNC Symbol;Acc:23698]
ENSP00000054666	1.868	0	VAMP3	vesicle-associated membrane protein 3 [Source:HGNC Symbol;Acc:12644]
ENSP00000247461	1.867	0	CANX	calnexin [Source:HGNC Symbol;Acc:1473]
ENSP00000450712	1.855	0.000001	HSP90AA1	heat shock protein 90kDa alpha (cytosolic), class A member 1 [Source:HGNC Symbol;Acc:5253]
ENSP00000440874	1.855	0	RTN3	reticulon 3 [Source:HGNC Symbol;Acc:10469]
ENSP00000336850	1.85	0.000024	RAB6A	RAB6A, member RAS oncogene family [Source:HGNC Symbol;Acc:9786]
ENSP00000450268	1.845	0	TUBA1A	tubulin, alpha 1a [Source:HGNC Symbol;Acc:20766]
ENSP00000475018	1.838	0	AP2B1	adaptor-related protein complex 2, beta 1 subunit [Source:HGNC Symbol;Acc:563]
ENSP00000354451	1.823	0.00482	IQGAP3	IQ motif containing GTPase activating protein 3 [Source:HGNC Symbol;Acc:20669]
ENSP00000468777	1.808	0.009526	TUBB6	tubulin, beta 6 class V [Source:HGNC Symbol;Acc:20776]
ENSP00000339001	1.806	0	TUBB	tubulin, beta class I [Source:HGNC Symbol;Acc:20778]
ENSP00000327070	1.805	0.000044	MDH2	malate dehydrogenase 2, NAD (mitochondrial) [Source:HGNC Symbol;Acc:6971]
ENSP00000417545	1.796	0.000674	ATP6V1A	ATPase, H+ transporting, lysosomal 70kDa, V1 subunit A [Source:HGNC Symbol;Acc:851]
ENSP00000405114	1.785	0.003678	ATP6V1B1	ATPase, H+ transporting, lysosomal 56/58kDa, V1 subunit B1 [Source:HGNC Symbol;Acc:853]
ENSP00000403778	1.78	0.006884	RAC2	ras-related C3 botulinum toxin substrate 2 (rho family, small GTP binding protein Rac2) [Source:HGNC Symbol;Acc:9802]
ENSP00000371894	1.764	0.000008	AP2M1	adaptor-related protein complex 2, mu 1 subunit [Source:HGNC Symbol;Acc:564]
ENSP00000427099	1.758	0.005573	C5orf28	chromosome 5 open reading frame 28 [Source:HGNC Symbol;Acc:26139]
ENSP00000348965	1.753	0	DYNC1H1	dynein, cytoplasmic 1, heavy chain 1 [Source:HGNC Symbol;Acc:2961]
ENSP00000424063	1.749	0	CANX	calnexin [Source:HGNC Symbol;Acc:1473]
ENSP00000262030	1.72	0	ATP5B	ATP synthase, H+ transporting, mitochondrial F1 complex, beta polypeptide [Source:HGNC Symbol;Acc:830]
ENSP00000262325	1.705	0	AP2B1	adaptor-related protein complex 2, beta 1 subunit [Source:HGNC Symbol;Acc:563]
ENSP00000300289	1.697	0	PDIA3	protein disulfide isomerase family A, member 3 [Source:HGNC Symbol;Acc:4606]
ENSP00000442318	1.692	0	CAND1	cullin-associated and neddylation-dissociated 1 [Source:HGNC Symbol;Acc:30688]
ENSP00000438260	1.639	0	PDIA3	protein disulfide isomerase family A, member 3 [Source:HGNC Symbol;Acc:4606]
ENSP00000408649	1.636	0.000142	MDH2	malate dehydrogenase 2, NAD (mitochondrial) [Source:HGNC Symbol;Acc:6971]
ENSP00000344106	1.617	0.00012	RTN3	reticulon 3 [Source:HGNC Symbol;Acc:10469]
ENSP00000229264	1.607	0.000245	GNB3	guanine nucleotide binding protein (G protein), beta polypeptide 3 [Source:HGNC Symbol;Acc:4400]
ENSP00000301071	1.6	0	TUBA1A	tubulin, alpha 1a [Source:HGNC Symbol;Acc:20766]
ENSP00000336799	1.585	0	TUBA1B	tubulin, alpha 1b [Source:HGNC Symbol;Acc:18809]
ENSP00000229277	1.576	0	ENO2	enolase 2 (gamma, neuronal) [Source:HGNC Symbol;Acc:3353]

ENSP00000302961	1.554	0	HSPA4	heat shock 70kDa protein 4 [Source:HGNC Symbol;Acc:5237]
ENSP00000435306	1.526	0.003345	GANAB	glucosidase, alpha; neutral AB [Source:HGNC Symbol;Acc:4138]
ENSP00000400022	1.501	0.000001	AP1B1	adaptor-related protein complex 1, beta 1 subunit [Source:HGNC Symbol;Acc:554]
ENSP00000269122	1.485	0	CLTC	clathrin, heavy chain (Hc) [Source:HGNC Symbol;Acc:2092]
ENSP00000344868	1.481	0.000004	SEPT7	septin 7 [Source:HGNC Symbol;Acc:1717]
ENSP00000430636	1.478	0	ENO3	enolase 3 (beta, muscle) [Source:HGNC Symbol;Acc:3354]
ENSP00000387286	1.455	0	RAB1A	RAB1A, member RAS oncogene family [Source:HGNC Symbol;Acc:9758]
ENSP00000297185	1.453	0.004612	HSPA9	heat shock 70kDa protein 9 (mortalin) [Source:HGNC Symbol;Acc:5244]
ENSP00000310226	1.446	0.00037	RAB1B	RAB1B, member RAS oncogene family [Source:HGNC Symbol;Acc:18370]
ENSP00000396127	1.425	0.00127	RAN	RAN, member RAS oncogene family [Source:HGNC Symbol;Acc:9846]
ENSP00000377470	1.408	0	CNP	2',3'-cyclic nucleotide 3' phosphodiesterase [Source:HGNC Symbol;Acc:2158]
ENSP00000394071	1.408	0.000665	GDI1	GDP dissociation inhibitor 1 [Source:HGNC Symbol;Acc:4226]
ENSP00000376763	1.398	0	CLTC	clathrin, heavy chain (Hc) [Source:HGNC Symbol;Acc:2092]
ENSP00000393340	1.396	0	TUBB	tubulin, beta class I [Source:HGNC Symbol;Acc:20778]
ENSP00000448725	1.392	0.000579	TUBA1A	tubulin, alpha 1a [Source:HGNC Symbol;Acc:20766]
ENSP00000449325	1.378	0	TUBA1B	tubulin, alpha 1b [Source:HGNC Symbol;Acc:18809]
ENSP00000446489	1.374	0.000002	ATP5B	ATP synthase, H+ transporting, mitochondrial F1 complex, beta polypeptide [Source:HGNC Symbol;Acc:830]
ENSP00000216962	1.37	0	PYGB	phosphorylase, glycogen; brain [Source:HGNC Symbol;Acc:9723]
ENSP00000269848	1.347	0.00307	PFKL	phosphofructokinase, liver [Source:HGNC Symbol;Acc:8876]
ENSP00000377466	1.345	0	CNP	2',3'-cyclic nucleotide 3' phosphodiesterase [Source:HGNC Symbol;Acc:2158]
ENSP00000216281	1.315	0	HSP90AA1	heat shock protein 90kDa alpha (cytosolic), class A member 1 [Source:HGNC Symbol;Acc:5253]
ENSP00000227378	1.294	0	HSPA8	heat shock 70kDa protein 8 [Source:HGNC Symbol;Acc:5241]
ENSP00000325875	1.292	0	HSP90AB1	heat shock protein 90kDa alpha (cytosolic), class B member 1 [Source:HGNC Symbol;Acc:5258]
ENSP00000396189	1.268	0.000002	HSP90AA1	heat shock protein 90kDa alpha (cytosolic), class A member 1 [Source:HGNC Symbol;Acc:5253]
ENSP00000417978	1.216	0.000537	RAB7A	RAB7A, member RAS oncogene family [Source:HGNC Symbol;Acc:9788]
ENSP00000295598	1.211	0	ATP1A1	ATPase, Na+/K+ transporting, alpha 1 polypeptide [Source:HGNC Symbol;Acc:799]
ENSP00000300161	1.211	0.000012	YWHAB	tyrosine 3-monooxygenase/tryptophan 5-monooxygenase activation protein, beta [Source:HGNC Symbol;Acc:12849]
ENSP00000362946	1.21	0.004672	RAB14	RAB14, member RAS oncogene family [Source:HGNC Symbol;Acc:16524]
ENSP00000258737	1.207	0.000063	RAC1	ras-related C3 botulinum toxin substrate 1 (rho family, small GTP binding protein Rac1) [Source:HGNC Symbol;Acc:9801]
ENSP00000432884	1.173	0.001303	HSPA8	heat shock 70kDa protein 8 [Source:HGNC Symbol;Acc:5241]
ENSP00000324173	1.155	0.000001	HSPA5	heat shock 70kDa protein 5 (glucose-regulated protein, 78kDa) [Source:HGNC Symbol;Acc:5238]
ENSP00000361930	1.14	0.003712	YWHAB	tyrosine 3-monooxygenase/tryptophan 5-monooxygenase activation protein, beta [Source:HGNC Symbol;Acc:12849]
ENSP00000265062	1.094	0.000006	RAB7A	RAB7A, member RAS oncogene family [Source:HGNC Symbol;Acc:9788]
ENSP00000414479	1.048	0.000001	TUBB	tubulin, beta class I [Source:HGNC Symbol;Acc:20778]
ENSP00000360609	1.016	0.000001	HSP90AB1	heat shock protein 90kDa alpha (cytosolic), class B member 1 [Source:HGNC Symbol;Acc:5258]
ENSP00000299138	1.015	0.00487	VPS35	vacuolar protein sorting 35 homolog (S. cerevisiae) [Source:HGNC Symbol;Acc:13487]
ENSP00000264710	1.01	0.000093	RAB10	RAB10, member RAS oncogene family [Source:HGNC Symbol;Acc:9759]
ENSP00000306330	0.948	0	YWHAG	tyrosine 3-monooxygenase/tryptophan 5-monooxygenase activation protein, gamma [Source:HGNC Symbol;Acc:12852]
ENSP00000318575	0.945	0.000502	TUBA8	tubulin, alpha 8 [Source:HGNC Symbol;Acc:12410]
ENSP00000299767	0.92	0.000001	HSP90B1	heat shock protein 90kDa beta (Grp94), member 1 [Source:HGNC Symbol;Acc:12028]
ENSP00000000233	0.793	0.001525	ARF5	ADP-ribosylation factor 5 [Source:HGNC Symbol;Acc:658]
ENSP00000290378	0.663	0.007581	ACTC1	actin, alpha, cardiac muscle 1 [Source:HGNC Symbol;Acc:143]
ENSP00000238081	0.635	0.00327	YWHAQ	tyrosine 3-monooxygenase/tryptophan 5-monooxygenase activation protein, theta [Source:HGNC Symbol;Acc:12854]
ENSP00000217182	0.563	0.000007	EEF1A2	eukaryotic translation elongation factor 1 alpha 2 [Source:HGNC Symbol;Acc:3192]
ENSP00000398239	0.549	0.002059	ACTA2	actin, alpha 2, smooth muscle, aorta [Source:HGNC Symbol;Acc:130]
ENSP00000358508	0.445	0.00772	ATP1A1	ATPase, Na+/K+ transporting, alpha 1 polypeptide [Source:HGNC Symbol;Acc:799]
ENSP00000459119	0.415	0	ACTG1	actin, gamma 1 [Source:HGNC Symbol;Acc:144]
ENSP00000224784	0.362	0.00002	ACTA2	actin, alpha 2, smooth muscle, aorta [Source:HGNC Symbol;Acc:130]

Supplementary Table S4. A list of NBE-MSC-MV proteome included in four categories.[a]

Angiogenesis	Anti-inflammation	Neurogenesis	Apoptosis
ANXA3	ABR	ABR	AARS
APOE	ACTG1	ACAT1	ABR
ATP5A1	ANXA3	ACTG1	ACO2
CAV1	APOE	AP2A1	ACTC1
CDC42	APP	AP2B1	ACTN4
CDH13	ATP1B1	ALCAM	ACTN1
HBB	ATP1B2	BIN1	ALB
RAB13	BCL6	ANK2	ALDOC
RAP1A	CALM2	APOE	BIN1
PRMT5	CAMK2A	APP	SLC25A4
NDRG2	CAMK2B	RHOA	SLC25A6
	CAMK2D	RHOB	ANXA5
	CAMK2G	RHOG	APOE
	CAPZA1	ATIC	RHOA
	CAPZA2	ATP2B2	RHOB
	CAV1	CALM2	ATP2A1
	CD59	CALR	ATP2B3
	CDC42	CAMK2A	BCL6
	CFL1	CAMK2B	CALR
	COL1A2	CAMK2G	CAV1
	DUSP3	CAPZB	CD59
	EIF4E	CDC42	CFL1
	FLNB	CFL1	CSE1L
	GSK3A	CKB	DCTN1
	GSTP1	AP2M1	DYNC1H1
	HMGB1	AP2S1	DNM2
	HPRT1	CNP	DPP6
	HRAS	CNTN1	EEF1A2
	HSP90AA1	CRMP1	EEF2
	HSP90AB1	DBN1	GARS
	HSPD1	DCTN1	SFN
	ITPR1	DPYSL2	PDIA3
	KPNB1	DPYSL3	GSK3A
	KRT1	FKBP4	GSTP1
	MBP	GAPDH	HBA2
	MYH9	GMFB	HBB
	NCAM1	GNAO1	HINT1
	NME1	GNAQ	HK1
	NRAS	GPM6A	HMGB1

PCBP2	GPM6B	HRAS
PLP1	GSK3A	HSPA1B
PPP3CA	GSTP1	HSPA5
PRKACA	HMGB1	HSPA9
PRKACB	HPRT1	HSP90AB1
PRKAR2A	HRAS	HSPD1
PRKAR2B	HSPA5	ITPR1
PRKCA	HSP90AA1	KIF5A
PRKCG	HSP90AB1	KPNB1
MAPK1	KIF5A	KRT8
MAPK3	KIF5C	LRP1
MAP2K1	LDHA	NME1
MAP2K2	LSAMP	NRAS
RAC1	LY6H	OPA1
RAC2	MAP1B	PFN1
RPS19	MBP	PKM
S100A13	MYH9	PLP1
S100B	MYH10	POR
SLC3A2	NCAM1	PPP1CA
SP100	NCAM2	PPP2CB
YWHAB	NME1	PPP2R1A
SEMA7A	NRAS	PPP2R1B
EIF4A3	OGDH	PPP2R2B
ABI1	OXCT1	PRKCA
ARPC4	PAFAH1B1	PRKCB
ARPC3	PLP1	PRKCG
ACTR3	PPP1CC	MAPK1
ACTR2	MAPK1	MAPK3
ARPC2	MAPK3	PRPS1
ARPC1A	MAP2K1	PSMD2
NCKAP1	MAP2K2	PYGL
CORO1A	PRPS1	RAC1
SIRT2	PURA	RPS6
PHLPP2	RAB3A	S100B
PRDX5	RAB5A	SPTAN1
GREM1	RAB13	SPTBN2
CYFIP2	RAC1	STXBP1
	RAC2	UBA1
	RAC3	UBE2V1
	RAP1A	UCHL1
	RTN1	VCP
	RYR2	VDAC1
	S100B	WFS1
	SH3GL1	YWHAB

SH3GL2	YWHAE
SLC1A2	YWHAG
SLC1A3	YWHAH
SNAP25	YWHAZ
SPTAN1	RAB7A
SPTBN2	CUL5
SYP	YARS
UBE2V1	DYNLL1
EZR	SYNJ1
YWHAB	VAPB
YWHAE	VAPA
YWHAG	ROCK2
YWHAH	ARHGEF11
ALDH5A1	PSMD6
USP9X	DNM1L
SEMA7A	PPIF
CNTNAP1	PDIA6
DYNLL1	BCAP31
ADAM23	RTN3
RAB11A	ARL6IP5
NAPA	NCKAP1
UBE2M	YWHAQ
ATP6V0D1	PDCD10
SYNGR3	EPB41L3
DCLK1	TARDBP
LGI1	DDAH2
VAPA	PRDX5
CPNE6	GIGYF2
NRXN3	CYFIP2
ROCK2	ATL1
ARHGEF11	DDX47
ACTR3	VPS35
TUBB3	RTN4
BASP1	CORO1B
DPYSL4	GNB4
NCKAP1	DNAJC5
PGRMC1	OBSCN
CYP46A1	DYNLL2
RAB10	MAGI3
EHD1	
YWHAQ	
RAB35	
RAB18	
SIRT2	

CNOT1
NFASC
PLXND1
EPB41L3
NCDN
ACSL6
CNTNAP2
IFT172
PHGDH
NPTN
TAGLN3
PACSIN1
ATL1
ATP8A2
PHF10
TMEM30A
DPYSL5
RTN4
NDRG2
GNB4
PGAP1
EFHD1
IGSF8
CEP120
CADM2
PTF1A
NEGR1
TUBB2B
POTEG

[a]Number of identified proteinss associated with the GO terms with p-value are 11 for angiogenesis with p value 3.24E-04, 76 for anti-inflammation with p value of 4.46E-04, 156 for neurogenesis with p value of 9.87E-30 and 122 for apoptosis with p value of 2.70E-13, respectively.

Supplementary Table S5. A list of SBE-MSC-MV proteome included in four categories.[b]

Angiogenesis	Anti-inflammation	Neurogenesis	Apoptosis
ANXA3	ACTG1	ACTG1	AARS
APOE	ACTB	ACTB	ACO2
ATP5A1	ANXA3	AP2A1	ACTC1
CDC42	APOE	AP2A2	ALDOC
CDH13	APP	AP2B1	BIN1
CSNK2B	ATP1B1	ALCAM	SLC25A6
HBB	ATP1B2	BIN1	ANXA4
RAB13	BCL6	ANK2	ANXA5
RAP1A	CAMK2A	APOE	APOE
PRMT5	CAMK2B	APP	RHOA
NDRG2	CAMK2D	RHOA	ATP2A1
	CAPZA2	RHOG	ATP2B3
	CDC42	ATIC	BCL6
	CFL1	ATP2B2	CALR
	EIF4A1	CALR	CFL1
	EIF4A2	CAMK2A	DAD1
	EIF4E	CAMK2B	DCTN1
	HMGB1	CAPZB	DDX5
	HPRT1	CDC42	DYNC1H1
	HRAS	CFL1	DNM2
	HSP90AA1	CKB	EEF1A2
	HSP90AB1	AP2M1	EEF2
	HSPD1	CNP	GARS
	ITPR1	CNTN1	SFN
	KPNB1	CRMP1	PDIA3
	KRAS	CSNK2A1	HBB
	KRT1	CSNK2B	HINT1
	MBP	DBN1	HK1
	NCAM1	DCTN1	HMGB1
	NME1	DPYSL2	HRAS
	NRAS	DPYSL3	HSPA5
	PCBP2	FKBP4	HSPA9
	PLP1	G6PD	HSP90AB1
	PPIA	GAPDH	HSPD1
	PPP3CA	GMFB	ITPR1
	PRKACA	GNAO1	KIF5A
	PRKACB	GNAQ	KPNB1
	PRKAR2B	GPM6A	KRAS
	PRKCA	GPM6B	KRT8

	PRKCG	HMGB1	LRP1
	MAPK1	HMGCS1	NF1
	MAPK3	HPRT1	NME1
	MAP2K1	HRAS	NME1-NME2
	PRSS3	HSPA5	NRAS
	RAC1	HSPA8	PFN1
	RAC2	HSP90AA1	PKM
	S100A13	HSP90AB1	PLP1
	S100B	KIF5A	PPP1CA
	SLC3A2	KIF5B	PPP2CB
	HSP90B1	KIF5C	PPP2R1A
	YWHAB	KRAS	PPP2R1B
	SEMA7A	LDHA	PPP2R2B
	EIF4A3	LSAMP	PRKCA
	ARPC4-TTLL3	LY6H	PRKCB
	ARPC4	MAP1B	PRKCG
	ARPC3	MBP	MAPK1
	ACTR3	NCAM1	MAPK3
	ACTR2	NF1	PRPS1
	ARPC2	NME1	PSMD2
	ARPC1A	NRAS	PYGL
	NCKAP1	OGDH	RAC1
	CORO1A	PAFAH1B1	RALB
	PHLPP2	PAFAH1B2	S100B
	PRDX5	PLP1	SPTAN1
	CYFIP2	MAPK1	SPTBN2
	TMED7-TICAM2	MAPK3	STXBP1
		MAP2K1	HSP90B1
		PRPS1	UBA1
		PURA	UCHL1
		RAB3A	VCP
		RAB5A	VDAC1
		RAB13	WFS1
		RAC1	YWHAB
		RAC2	YWHAE
		RAC3	YWHAG
		RAP1A	YWHAH
		RRAS	YWHAZ
		RTN1	RAB7A
		RYR2	CUL5
		S100B	DYNLL1
		SH3GL2	SYNJ1
		SLC1A2	VAPB
		SLC1A3	VAPA

		SNAP25<br>SPTAN1<br>SPTBN1<br>SPTBN2<br>SYP<br>CNTN2<br>YWHAB<br>YWHAE<br>YWHAG<br>YWHAH<br>ALDH5A1<br>USP9X<br>SEMA7A<br>CNTNAP1<br>DYNLL1<br>ADAM23<br>RAB11A<br>NAPA<br>ATP6V0D1<br>SYNGR3<br>DCLK1<br>VAPA<br>CPNE6<br>NRXN3<br>ROCK2<br>ACTR3<br>TUBB3<br>BASP1<br>DPYSL4<br>NCKAP1<br>PGRMC1<br>RAB10<br>EHD1<br>YWHAQ<br>RAB35<br>RAB18<br>CNOT1<br>NFASC<br>PLXND1<br>EPB41L3<br>NCDN<br>CYFIP1<br>ACSL6<br>IFT172	ROCK2<br>MATR3<br>PDCD6IP<br>DNM1L<br>PDIA6<br>BCAP31<br>RTN3<br>GNB2L1<br>NCKAP1<br>YWHAQ<br>EPB41L3<br>TARDBP<br>DDAH2<br>NGEF<br>PRDX5<br>CYFIP2<br>ATL1<br>DDX47<br>VPS35<br>RTN4<br>GNB4<br>DNAJC5<br>OBSCN<br>MAGI3	

		PHGDH	
		NPTN	
		TAGLN3	
		PACSIN1	
		ATL1	
		TMEM30A	
		DPYSL5	
		RTN4	
		NDRG2	
		GNB4	
		MTPN	
		CEP120	
		CADM2	
		NEGR1	
		TUBB2B	
		POTEE	

[b]Number of identified proteinss associated with the GO terms with p-value are 11 for angiogenesis with p value 9.45E-05, 66 for anti-inflammation with p value of 1.09E-03, 143 for neurogenesis with p value of 3.72E-30 and 107 for apoptosis with p value of 2.69E-12, respectively.

Supplementary Table S6. The list of primers used for RT-PCR

Target gene	Primer sequence	Product size (bp)
rat IL-10	Forward 5′-ACCTGGTAGAAGTGATGCCCCAGGCA-3′<br>Reverse 5′-CTATGCAGTTGATGAAGATGTCAA-3′	237
rat PGRN	Forward 5′AGTGTCCAGATGGGCAGTTC-3′<br>Reverse 5′-ATTGGGCATTGGACAGCAGC-3′	650
rat TSG-6	Forward 5′-CAGATGGGCTGTACCTTATC-3′<br>Reverse 5′-TGACTCCAAAGTAGACCTGC-3′	180
rat TNF-α	Forward 5′-CAGATGGGCTGTACCTTATC-3′<br>Reverse 5′-TGACTCCAAAGTAGACCTGC-3′	306
rat β-actin	Forward 5′-TCATGAAGTGTGACGTGGACATC-3′<br>Reverse 5′-TGTTGCATTTGCGGGGACGATG -3′	282
